# Accelerating inhibitor discovery for deubiquitinating enzymes

**DOI:** 10.1038/s41467-023-36246-0

**Published:** 2023-02-08

**Authors:** Wai Cheung Chan, Xiaoxi Liu, Robert S. Magin, Nicholas M. Girardi, Scott B. Ficarro, Wanyi Hu, Maria I. Tarazona Guzman, Cara A. Starnbach, Alejandra Felix, Guillaume Adelmant, Anthony C. Varca, Bin Hu, Ariana S. Bratt, Ethan DaSilva, Nathan J. Schauer, Isabella Jaen Maisonet, Emma K. Dolen, Anthony X. Ayala, Jarrod A. Marto, Sara J. Buhrlage

**Affiliations:** 1 Department of Cancer Biology and the Linde Program in Cancer Chemical Biology, Dana-Farber Cancer Institute, Boston, MA USA; 2 Department of Biological Chemistry and Molecular Pharmacology, Harvard Medical School, Boston, MA USA; 3 Blais Proteomics Center, Dana-Farber Cancer Institute, Boston, MA USA; 4 Center for Emergent Drug Targets, Dana-Farber Cancer Institute, Boston, MA USA; 5 Department of Pathology, Brigham and Women’s Hospital and Harvard Medical School, Boston, MA USA

**Keywords:** Proteomics, Ubiquitylation, Chemical tools, Screening

## Abstract

Deubiquitinating enzymes (DUBs) are an emerging drug target class of ~100 proteases that cleave ubiquitin from protein substrates to regulate many cellular processes. A lack of selective chemical probes impedes pharmacologic interrogation of this important gene family. DUBs engage their cognate ligands through a myriad of interactions. We embrace this structural complexity to tailor a chemical diversification strategy for a DUB-focused covalent library. Pairing our library with activity-based protein profiling as a high-density primary screen, we identify selective hits against 23 endogenous DUBs spanning four subfamilies. Optimization of an azetidine hit yields a probe for the understudied DUB VCPIP1 with nanomolar potency and in-family selectivity. Our success in identifying good chemical starting points as well as structure-activity relationships across the gene family from a modest but purpose-build library challenges current paradigms that emphasize ultrahigh throughput in vitro or virtual screens against an ever-increasing scope of chemical space.

## Abstract (web summary)

Deubiquitinases (DUBs) are key signaling enzymes, many of which lack selective inhibitors. Chan *et al*. pair a DUB-focused covalent library to mass spectrometry activity-based protein profiling, leading to selective hits against 23 endogenous DUBs and a first-in-class VCPIP1 probe with nanomolar potency.

## Introduction

The deubiquitinating enzymes (DUBs) comprise a family of approximately 100 structurally and functionally related enzymes that catalyze the cleavage of ubiquitin and ubiquitin-like (UBL) post-translational marks from substrate proteins. As key members of the ubiquitin-proteasome system (UPS), DUBs are functionally involved in numerous cellular processes^1^. Moreover, DUBs have been linked to human cancer and neurodegenerative disease^2^, suggesting that DUB inhibitors could have a significant therapeutic impact. However, efforts to demonstrate the clinical benefit of DUB inhibition have been hampered by the poor selectivity of early compounds, as well as a limited understanding of DUB biology, especially the scope of unique and shared substrates across the family of DUB enzymes. A dedicated platform to develop selective DUB inhibitors would provide tool compounds (chemical probes) and drug discovery leads to advance both basic and translational understanding of this large protein family. The majority of the annotated DUBs are cysteine proteases, which are further classified into six subfamilies based on sequence homology^2^. A combination of shared and unique structural features surrounding the catalytic site, along with diversity in primary sequence suggest that cysteine protease DUBs can be selectively targeted with small molecule inhibitors. However, as with other emergent drug target-classes past and present, the field has struggled to gain pharmacological traction for the DUBs. Early generation DUB inhibitors have been found in retrospect to be multitargeted^3,4^. Recently, we and others, have succeeded in developing the first selective inhibitors for a small subset of DUBs (USP7, USP9X, USP28, and USP30)^5–8^. While these results establish precedent for selective small molecule inhibition of individual DUBs, the chemical tractability of the broader class remains unclear^9^.

Numerous advances have been reported in technologies for screening focused or diversity libraries against new targets or in more limited cases, functionally related protein families^10–16^. The ability to achieve higher assay throughput has been accompanied by a significant expansion of the small molecule chemical space available for screening^17–19^. Despite this progress the pace of hit discovery for DUBs remains stubbornly slow, with sparse coverage of the gene family, which is inadequate to glean target-class SAR or other insight for inhibitor design. Hit rate notwithstanding, high-throughput biochemical-based screens are often limited to the catalytic domain, which for DUBs may only provide a partial assessment of compound binding and selectivity. As a result, pharmacologic interrogation of DUB biology as well as rigorous validation of specific DUBs as bona fide drug targets remain largely out of reach.

In this work, to accelerate the systematic development of selective inhibitors for the DUB family we created a covalent library diversified to target multiple, discrete regions around the catalytic site. We paired our focused library with activity-based protein profiling (ABPP) to facilitate the assessment of selectivity against endogenous, full-length DUBs. Moreover, this format enabled us to perform simultaneous hit and SAR deconvolution in the primary screen. Our DUB inhibitor discovery platform reported here spans 178 compounds screened against 65 cellular DUBs with hit validation across multiple orthogonal assays. Although modest in size, our rationally designed library and high-density primary screen provided hits against 45 cellular DUBs, including 23 DUBs by a selective compound, along with target-class SAR. These insights drove rapid development of an azetidine hit-compound into a selective 70 nM covalent inhibitor of the understudied DUB, VCPIP1. Our platform highlights the value of multi-site, structure-guided diversification as a powerful approach to rational library design for small molecule hit discovery against recalcitrant protein families.

## Results

### A DUB-focused library × library screening platform

**Fig. 1:**
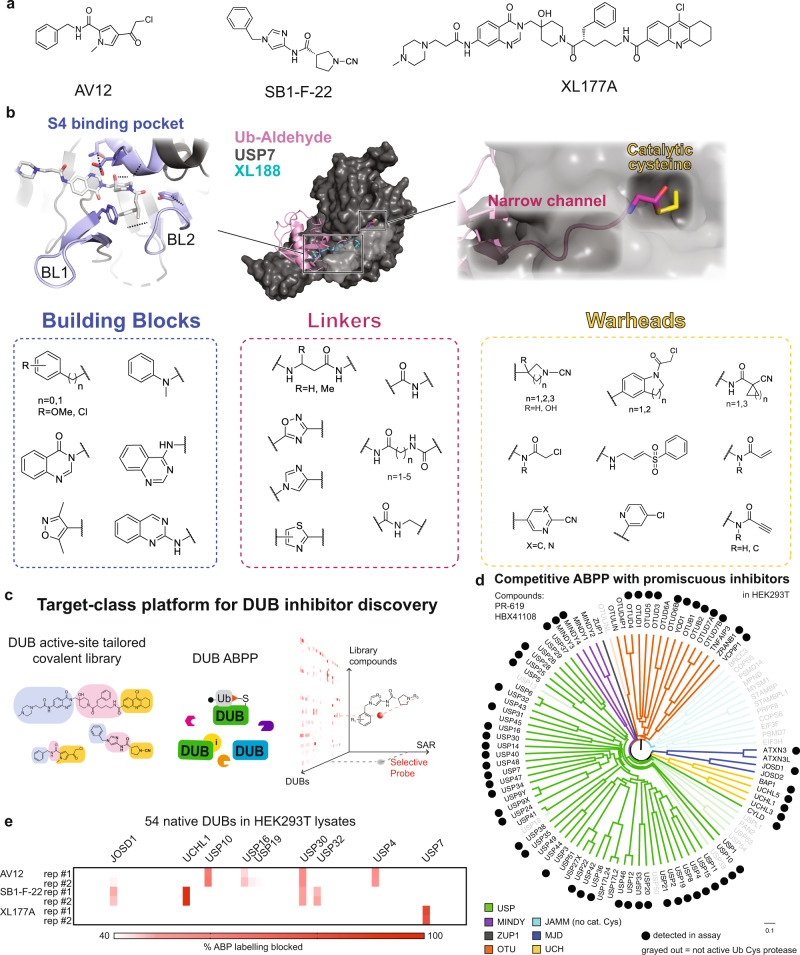
A DUB-tailored inhibitor discovery platform. **a** Structures of DUB inhibitors AV12, SB1-F-22, and XL177A. **b** Library compounds followed a three-piece modular design, where noncovalent building blocks, linkers, and warhead moieties were diversified to target conserved and divergent aspects of the DUB catalytic domain and nearby regions. *Blue*: Noncovalent building blocks were designed to target the blocking loops (BL) 1 and 2, in addition to the P4 site. *Red*: Linkers were designed to traverse a long and narrow channel leading up to the catalytic cysteine residue, occupied by the C-terminus of the substrate ubiquitin in catalysis. In the top panel, an overlay of crystal structures for USP7 with Ub-aldehyde (PDB: 1NBF) and USP7 with XL188 (PDB: 5VS6) are shown, with USP7 in gray, Ub-aldehyde in pink, and XL188 in cyan. *Yellow*: electrophilic warheads were designed to target the invariant catalytic cysteine residue shown in yellow in the top panel. **c** The target-class platform yields high content data to drive DUB hit identification and SAR for compound optimization. **d** Analysis of multitargeted covalent DUB inhibitors (HBX41108 and PR619) confirmed that our DUB-ABPP platform spanned members from all subfamilies of cysteine protease DUBs^3,4^. The dendrogram is colored by DUB subfamily. **e** ABPP analysis using covalent inhibitors depicted in (**a**) in duplicate runs (each shown individually) validated the ability of our platform to read-out selective compound binding activity with deep coverage of the human DUBome.

Our rationally designed library was inspired by diverse DUB inhibitor chemotypes. (Fig. 1a) These include a selective USP7 inhibitor, XL177A developed in our lab^20^, N-cyanopyrrolidines including SB1-F-22 identified in a patent from Mission Therapeutics and validated in-house to modify the active site cysteine of UCHL1^21–23^ (Supplementary Fig. 1), and AV12 (referred to as **1** in the reporting manuscript) which binds multiple DUBs^24^. We analyzed DUB-ligand/-ubiquitin co-structures^25–28^ (Fig. 1b) to identify regions around the catalytic site that may favor compound interaction, as well as possible determinants of selectivity. We then implemented a combinatorial assembly of noncovalent building blocks, linkers, and electrophilic warheads. The noncovalent building blocks incorporated a variety of aromatic and heterocycle moieties to harness interactions with blocking loops 1 and 2 in the leucine-binding pocket S4^27,28^. The linkers were designed to mimic the C-terminal residues of ubiquitin (GG) and traverse a narrow channel leading up to the catalytic cysteine (Fig. 1b)^25^. To capitalize on structural and sequence variation in this channel across DUBs, we diversified length, flexibility, and presentation of hydrogen bond donor/acceptor groups in the linker. We anticipated that interactions between each chemical component and the catalytic diad/triad as well as less-conserved, neighboring regions on each DUB would contribute to overall binding potency and selectivity.

We synthesized most library compounds with a reactive component comprising a ring system elaborated with an electrophile. This design was inspired by our chemotypes and differentiates our library from general electrophile collections which have been largely unsuccessful in yielding DUB ligands. The reactive groups span four general categories: cyano, α,β-unsaturated amide/sulfonamide, chloroacetamide and halogenated aromatics. We diversified within each category with respect to electrophilic functionality as well as pendant ring system (Supplementary Data 1, Synthetic procedures provided in Supplementary Methods). Finally, the library also included a small number (<10) of putatively covalent DUB inhibitors.

We chose activity-based protein profiling (ABPP) coupled with quantitative mass spectrometry as the primary screen platform (Fig. 1c, Supplementary Fig. 2). In general, DUB ABPs consist of ubiquitin protein to confer DUB specificity, an C-terminal electrophile to form a covalent attachment to the DUB catalytic cysteine residue, and an affinity handle at the N-terminus to enable DUB enrichment by capture on streptavidin beads (Supplementary Fig. 2a)^29^. In the experiments in this manuscript, we utilize a 1:1 combination of biotin-Ub-VME and biotin-Ub-PA (Supplementary Fig. 2a). To maximize DUB coverage as well as compound throughput and peptide detection, we used a combination of DUB ABPs along with isobaric TMT multiplexed reagents and our true nanoflow LC columns with integrated electrospray emitters^30,31^. While not typically used at this early stage of inhibitor development, we reasoned that performing this competitive binding assay in a library × library format would accelerate progress on two fronts. First, the assay format maximizes our ability to identify hits against a large number of DUBs in their endogenous, native environment. Second, the integration of ABPP results across our library provides valuable insight for DUB family structure-activity relationships. These SAR data can simultaneously inform medicinal chemistry optimization of hits as well as guide design of new libraries. We tested our chemoproteomic ABPP assay using foundation chemotypes (Fig. 1a) as well as previously characterized multitargeted DUB inhibitors PR-619 and HBX41108^3^. These validation data spanned 54 cellular DUBs, which represents a significant advance in coverage compared with recent reports and recapitulated the DUB binding profiles expected for our selective chemotypes as well as the promiscuous covalent compounds (Fig. 1d, e)^6,32^.

### Primary screen and hit identification

**Fig. 2:**
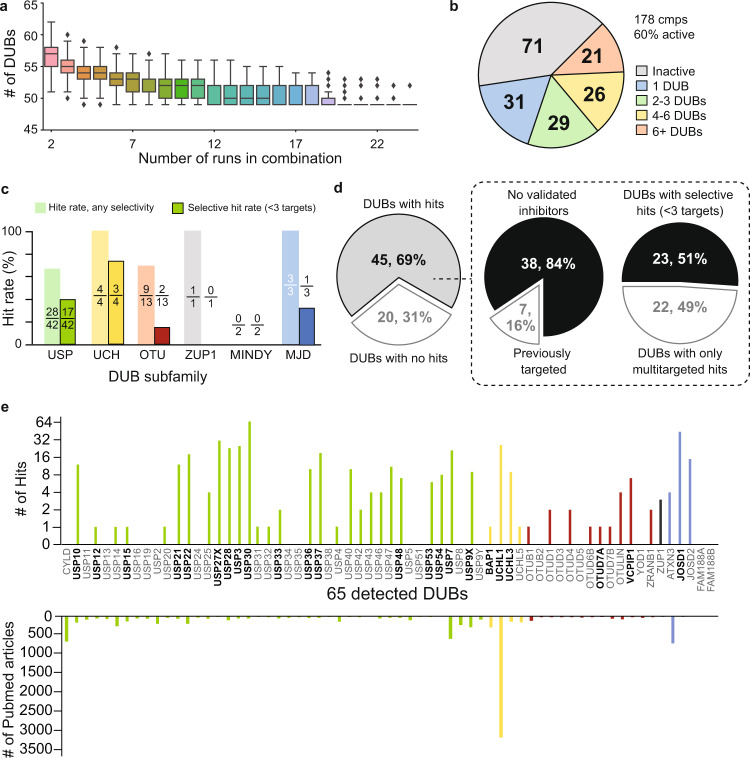
DUB trends in primary screening. **a** *DUB ABPP assay performance* The number of DUBs consistently detected across each unique combination of 2–24 assay runs (*n* = 25 biologically independent experiments) quickly stabilized to 49, with 56 DUBs detected in >80% of runs. Line at center of box shows median number of DUBs detected for the given number of assays considered, box encapsulates the middle two quartiles, while bars represent bounds for the rest of the distribution. “Outlier” points as calculated by an inter-quartile method are shown as diamonds. **b** Most compounds were active and spanned diverse selectivity profiles. **c** Hit rates were roughly consistent across the DUB subfamilies, with no hits identified for the MINDY subfamily. Insets above indicate number of DUBs targeted (numerator) with any selectivity (light color) or selectively with <3 targets (dark color) out of all DUBs in the subfamily (denominator). **d** *left:* Hits were successfully identified for 69% (45 out of 65) of the detected DUBs. *Dashed box, left pie chart:* 84% (38 out of 45) of hits did not have validated inhibitors reported. *Dashed box, right pie chart:* 51% (23 out of 45) of hits were targeted by selective compounds with <3 DUB targets. **e** *DUBs targeted by library compounds*. Individual DUBs showed a range of targetability by our library, within 1 to 65 library compounds hitting each targeted DUB, number of hits per DUB shown in the upper bar chart. Hit compounds were successfully identified against understudied members of the target class, including 11 DUBs with ≤ 20 publications, number of Pubmed articles for each DUB shown in lower bar chart. DUB from different subfamilies were grouped and shown in different colors: USP (green), UCH (yellow), OTU (maroon), ZUP1 (black), MJD (blue), MINDY (no hits, no colored bar). Selectively targeted DUBs are shown in bold.

We next initiated the primary screen of our library, comprising 178 DUB-focused compounds each incubated at a single concentration of 50 µM in cellular protein extracts. We analyzed the entire library across 25 multiplexed chemoproteomic ABPP acquisitions. We first assessed the platform by analyzing ABPP assay performance and then characterizing primary hits against the DUB family. In total, we detected 65 distinct DUBs, representing 75% of the cysteine protease DUB enzymes expressed in HEK293 cells as reported in the Human Protein Atlas^33^. Consistent with our platform validation data, we detected on average 58 DUBs per ABPP run (Supplementary Fig. 3b). Importantly, combinatorial sampling of data across all multiplexed acquisitions indicated that the number of DUBs reproducibly detected stabilized at 49, with 56 DUBs detected in >80% of runs (Fig. 2a). Taken together, results from our analytical validation and primary screen suggest that our DUB-ABPP platform is well-suited for identification of hits against individual DUBs while also providing SAR data across the target class.

To focus our analysis on the most promising compounds we defined a ‘hit compound’ as a compound that blocked ≥50% of ABP labeling for at least a single DUB. More than 60% of library compounds were active, suggesting good fidelity between our chemical diversification strategy and binding activity against the target class. Consistent with first-generation libraries designed for other enzyme classes^34,35^, the number of DUBs targeted by each compound varied broadly (Fig. 2b). Impressively, 60 of the compounds (just over 50% of all hits) displayed excellent selectivity profiles, targeting only 1-3 DUBs. The remaining hit compounds were split among those targeting 4-6 or 6+ DUBs. Other ubiquitin interacting enzymes (e.g. E1, E2, E3 enzymes) were not competitively bound by the library compounds, supporting potential specificity for DUBs (Supplementary Fig. 3a) and further validating our strategy for library design. DUB coverage by our library compounds was broad. Hit compounds spanned 2/3 of all DUBs detected (45 out of 65, 69%) and encompassed five of the six DUB subfamilies (USP, UCH, OTU, MJD, ZUP1) (Fig. 2c). Notably, of the 45 DUBs competitively bound by a hit-compound, 23 were selectively targeted with ≤ 3 other DUB targets. (Fig. 2d). These DUBs span multiple subfamilies (MJD, USP, UCH, OUT) and most (17 out of 23, 74%) have no reported ligands and are largely understudied (Fig. 2d, e). Taken together, these results demonstrate that our focused library and analytical platform work in a coordinated way to drive hit identification broadly across the enzyme family.

**Fig. 3:**
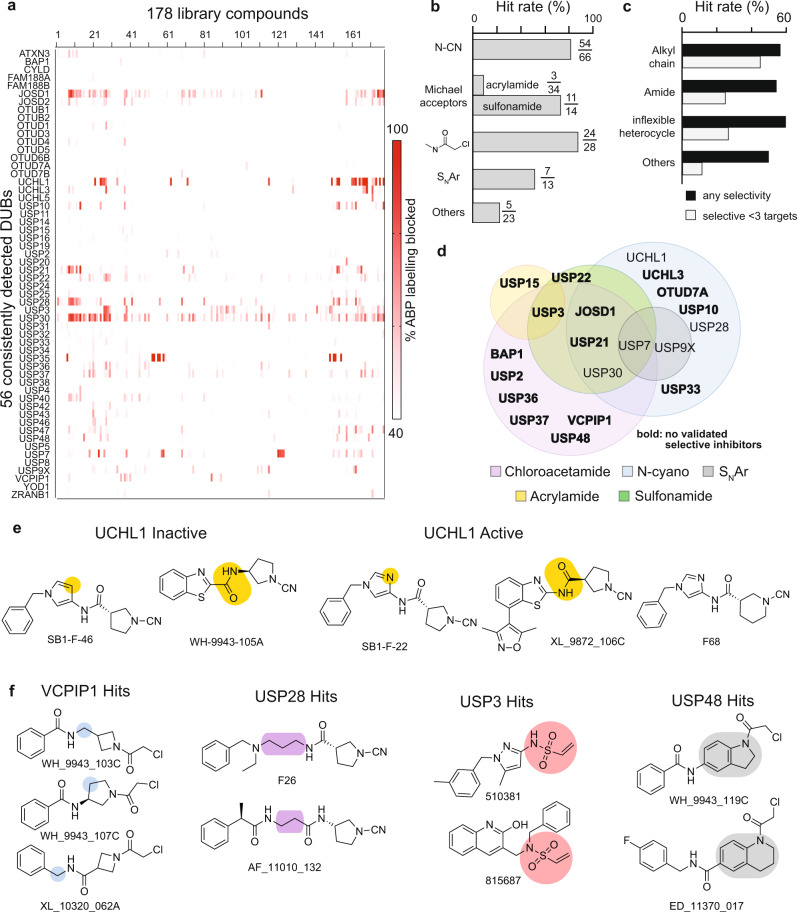
Chemical trends in primary screening. **a** Heatmap representing potency and selectivity data for all 178 library compounds against 56 DUBs detected consistently over 80% of runs. **b** Electrophiles displayed diverse DUB hit rates. Acrylamides exhibited poor activity towards DUB catalytic cysteines. Insets adjacent to each bar indicate the number of hits and the total number of compounds containing each electrophile. **c** Presence and type of linker influenced compound activity and selectivity. **d** Individual warheads showed partially overlapping DUB target profiles. Venn diagram is colored by warhead, legend in figure. Bolded DUBs are hitherto untargeted. **e**, **f** Selected representative examples for multiple chemically similar hits for the same DUB from the screen. In the case of UCHL1, stringent requirements surrounding hydrogen bond acceptor position and amide orientation on the linker are highlighted in yellow. For VCPIP1, compounds were structurally similar except for the methylene group which moved along the backbone of the molecule (in blue). Moieties common across hits are highlighted for USP28 (in purple), USP3 (in red), and USP48 (in gray).

Our ability to reproducibly profile 56 cellular DUBs afforded an opportunity to glean design principles and SAR insights based on chemical features found across hit-compounds (Fig. 3a, Supplementary Data 2). While the hit-compounds comprised all electrophile groups in our library, heterocycle rings elaborated with cyanamides, chloroacetamide or vinyl sulfones were prominent (Fig. 3b). Similarly, all linker groups designed to traverse the narrow channel of DUBs normally occupied by the C-terminus of ubiquitin (Fig. 1b) were exemplified in our hit-compounds, with strong representation of flexible alkyl linkers among selective hits (Fig. 3c). In terms of the target binding profiles, DUBs displayed non-overlapping electrophile preferences (Fig. 3d). In addition, we observed broad chemical diversity in the noncovalent building blocks comprising the hit-compounds.

Selective binders for a given DUB display high structural similarity within all three constituent building blocks, while compounds targeting different DUBs exhibited a combination of overlapping and distinct chemical features (Fig. 3e, f). As a specific example, we observed that selective UCHL1 and USP28 hit-compounds include cyanamides contained within alkyl rings: all USP28 hits contained the cyanamide nitrogen within a 5-membered ring while selective UCHL1 binders contained 4–6-membered rings, with the highest selectivity provided by the 6-membered piperidine warhead. Within the linker region, UCHL1 ligands contained 5-membered heterocycles and required precise placement of a hydrogen bond acceptor within the aromatic ring, while all USP28 hits contained a flexible alkyl linker. We observed that hits targeting VCPIP1 and USP48 contain similar small hydrophobic building blocks and simple amide linkers paired with distinct ring-electrophile warheads: VCPIP1 ligands shared an azetidyl chloroacetamide while USP48 targeting compounds feature a bulky fused indoline or tetrahydroquinoline with the heterocycle nitrogen elaborated into a chloroacetamide reactive group.

Taken together these results highlight the power of quantitative ABPP as a primary screen to simultaneously identify hit-compounds and discern SAR trends. Consistent with our multi-site diversification strategy, we observed that distinct regions of DUB active sites (catalytic and nearby residues, narrow channel leading to the active site, the blocking loops and the P4 pocket) all contribute to selectivity.

### Orthogonal hit validation

**Fig. 4:**
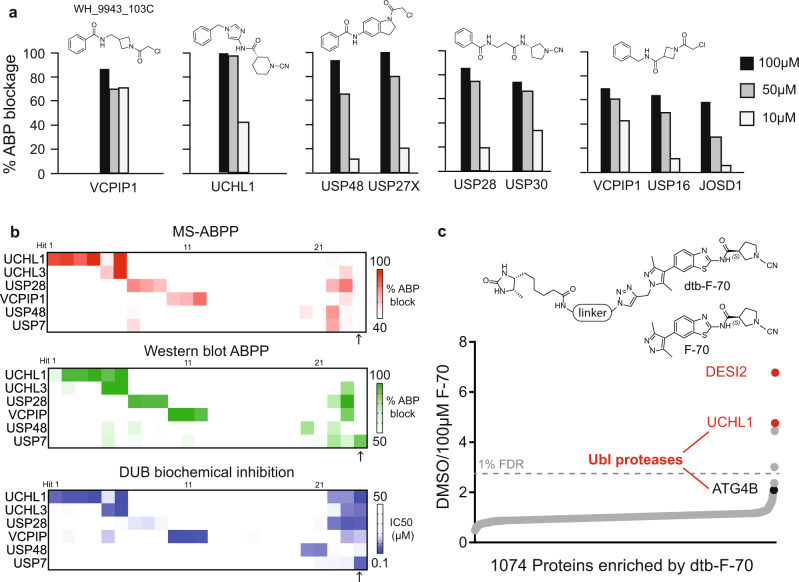
Hit validation. **a** Selected hits were confirmed by dose–response DUB-ABPP over 100, 50, 10 µM. **b** Overall, we observed high confirmation rates and excellent agreement for assays spanning compound binding in mass spectrometry (red), western blot (green), and enzyme inhibition (blue) for library hits targeting UCHL1, UCHL3, USP7, USP28, USP48 and VCPIP1. An outlier (XL-9872-111B) for which we observed poor agreement across assay types is shown on the rightmost column. Data for each DUB is shown in a row (6 DUBs total) and data for each compound is shown in a column (24 compounds total). For individual compound structures and primary screen activity, please refer to Supplementary Data 3. **c** In a competition experiment against a desthiobiotinylated analog, F-70 shows dose–response binding to only 4 proteins among 1074 proteins detected with a 1% FDR cutoff as measured by a two tailed Z-test. Top DUB hits are highlighted in red. Source data are provided as a source data file.

Our primary ABPP screen yielded selective hit-compounds for 23 DUBs, spanning 4 DUB families (MJD, USP, UCH, OTU). Among these, we prioritized compounds targeting 9 different DUBs (UCHL1, UCHL3, USP16, USP27X, USP28, USP30, USP48, JOSD1 and VCPIP1), representing four subfamilies, for validation studies. Compound selection was based on strength of competitive binding along with structural and target diversity. We first re-tested all prioritized hit-compounds in a 3-point dose–response implementation of our ABPP MS assay (Fig. 4a, Supplementary Fig. 3c). In addition, we confirmed competitive ABP-compound binding by western blotting first in cell lysate (Fig. 4b, Supplementary Fig. 4, Supplementary Data 3), then in live cells (Supplementary Fig. 5). Cell viability was unaltered during 24-h compound treatments at 0.25x-4x biochemical IC_50_ for 5 out of 6 compounds selected, each targeting a different DUB (Supplementary Fig. 5). We next biochemically validated inhibition of DUB activity. The potencies of hit compounds varied from sub-micromolar to double-digit micromolar, on par with typical hits from a high-throughput screen (Fig. 4b, Supplementary Tables 2, 3). We next combined data-dependent and targeted LC-MS/MS along with intact protein mass spectrometry to confirm 1:1 ligand binding and covalent modification of the catalytic cysteine for UCHL1, UCHL3, USP7, USP48, USP28, and VCPIP1 ligands (Supplementary Fig. 6, Supplementary Table 4). Overall, we observed good cell permeability, minimal toxicity, high confirmation rates and excellent agreement for assays spanning compound binding in both live cell and lysates, mechanism of action, and enzyme inhibition for library hits targeting UCHL1, UCHL3, USP28, USP48 and VCPIP1 (Fig. 4b).

N-cyanopyrrolidines were strongly represented among hit-compounds, including F-70 which selectively bound UCHL1 with 400nM potency, as previously reported^21,36^. We synthesized a desthiobiotinylated analog (dtb-F-70) and co-incubated it with the native compound in cell extracts followed by multiplexed chemoproteomic analysis. These data confirmed UCHL1 as one of only 6 competitively bound targets throughout the proteome (Fig. 4c, Supplementary Data 4). Interestingly, two off-target enzymes (DESI-2 and ATG4B) were recently reported to deconjugate UBL marks, suggesting that N-cyanopyrrolidines may represent a privileged scaffold for UB- and UBL-targeted proteases^37,38^.

**Fig. 5:**
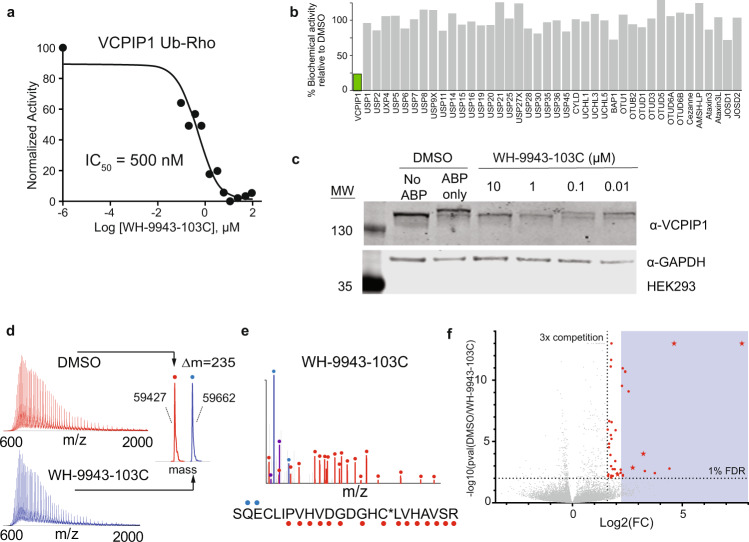
VCPIP1 hit validation. **a** Hit molecule WH-9943-103C inhibited deubiquitination activity of VCPIP1 in Ub-Rho cleavage assay after 6 h incubation (*n* = 1). **b** WH-9943-103C inhibited VCPIP1 (highlighted in green) selectively out of a panel of 41 purified recombinant DUBs after 15 min incubation. **c** WH-9943-103C displayed in-cell target engagement as determined for DUB labeling by ABP then visualized on a Western blot. **d** WH-9943-103C labeled recombinant VCPIP1 with 1:1 stoichiometry as read out by intact protein mass spectrometry. Labeled ion envelope is shown in blue, unlabeled ion envelope from DMSO control is shown in red. **e** CE-MS/MS identified the catalytic cysteine of VCPIP1 to be covalently modified by WH-9943-103C, red and blue glyphs adjacent to the peptide sequence indicate y- and b-type fragment ions, respectively, detected in the MS/MS spectrum. The y-ion series confirms catalytic cysteine modification (C*). **f** A proteome-wide competitive binding survey of WH-9943-103C activity (50µM) indicated off-target modification of 39 (red dots, FDR <1%, >3-fold competition relative to DMSO) out of 24,579 unique cysteines detected (p-values for all detected ratios derived from maximum likelihood estimation and corrected by the Benjamini-Hochberg method). The blue box indicates >4.5-fold competition relative to DMSO, the observed fold competition for VCPIP1. This includes 4 cysteines that were previously characterized as ‘hyper-reactive’ (red stars). Source data are provided as a source data file.

Compounds targeting the understudied DUB VCPIP1, including WH-9943-103C, were among the most potent and selective across our library with sub-micromolar biochemical IC_50_ (Fig. 5a), validated biochemical selectivity with purified DUBs (Fig. 5b), engagement of the native enzyme in cell lysate (Fig. 5c), and validated covalent modification of the catalytic cysteine residue on purified VCPIP1 protein (Fig. 5d, e). WH-9943-103C contains an azetidyl chloroacetamide warhead, which has not been examined prior to this study for proteome-wide reactivity, a primary consideration for covalent inhibitors. Towards this end, we treated cellular protein extracts with either vehicle (DMSO), 50 µM WH-9943-103C, or 50 µM XL177A (negative control for VCPIP1 covalent binding) followed by trypsin digest, TMT stable isotope labeling, and biochemical enrichment of cysteine-containing peptides. We performed quantitative, multiplexed chemoproteomic cysteine profiling using both targeted and data-dependent MS/MS acquisition strategies to characterize the covalent binding of WH-9943-103C on VCPIP1 as well as across the broader set of cysteine residues across proteome, respectively (Supplementary Fig. 7, Supplementary Data 5)^39^. Targeted MS/MS analysis by PRM-LIVE demonstrated a 4.5-fold reduction (corresponding to ~78% target occupancy) in detected signal for the catalytic cysteine VCPIP1 peptide in the WH-9943-103C treated extracts compared to vehicle or XL-177A treatment conditions (Supplementary Fig. 7). In the global experiment we quantified 24,579 unique cysteines. For selectivity assessment, we relaxed the binding occupancy threshold to 66% (3-fold TMT signal reduction for treated compared to DMSO, 1% FDR) and identified 39 potential off-target cysteines for WH-9943-103C (Fig. 5f), including 4 that were previously characterized as ‘hyper-reactive’^14^. Most off-targets (23 of 39) were bound with lower occupancy compared to VCPIP1. These results supported medicinal chemistry optimization of the chemical series. These results confirm that our combinatorial diversification strategy drives selectivity not only within the target class, but also across the broader proteome.

### Optimization of a potent, selective probe for VCPIP1

VCPIP1 belongs to the OTU sub-family and, while not extensively studied, has recently emerged as a potential drug target in botulism intoxication^40^. Based on the demonstrated target-class and proteome-wide selectivity of WH-9943-103C (Fig. 5f), along with the understudied status of its primary DUB target (VCPIP1) we sought to optimize this compound into high quality tool or chemical probe. Given that only six compounds from the azetidyl chloroacetamide chemotype existed our initial library, we synthesized a 20-compound expansion set, incorporating chemical diversity within the warhead, linker, and noncovalent components. Assessment was streamlined by monitoring potency (ubiquitin-rhodamine assay) and target-class selectivity (chemoproteomic DUB ABPP).

**Fig. 6:**
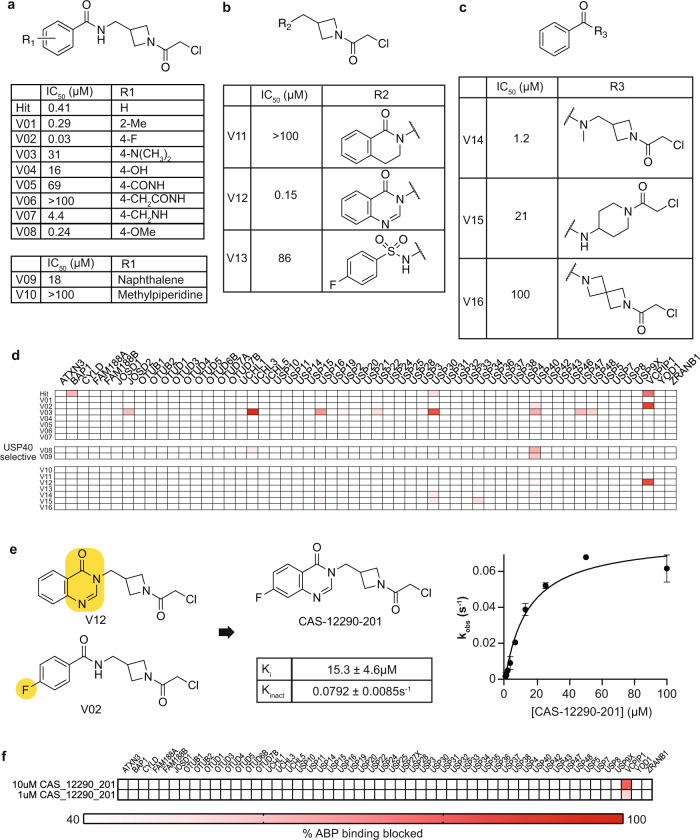
Optimization of a potent and selective VCPIP1 inhibitor. **a**–**c** Structures and VCPIP1 biochemical inhibitory activity of WH-9943-103C analogs. Data for hit WH-9943-103C are duplicated from earlier figures for ease of comparison. **d** DUB-ABPP data for the WH-103C focused library. Compounds with VCPIP1 biochemical IC_50_ values below 250 nM (V02, V08, V12) were screened in ABPP at 10 μM, less potent compounds were screened at 50 μM. **e**, **f** SAR analysis suggested a combination of functionalities on V12 and V02 (yellow highlights) to yield CAS-12290-201. Binding kinetics and DUB-ABPP (10 and 1 µM) demonstrated that CAS-12290-201 to be a potent and selective inhibitor of the understudied DUB VCPIP1, error bars show the 95% confidence interval for parameters as fitted by GraphPad Prism 9.0.1.

Collectively, members of the azetidyl chloroacetamide chemotype hit several DUBs including VCPIP1 (WH-9943-103C), BAP1 (WH-9943-103C), USP16 (V03), and USP40 (V08, V09). There were clear SAR in the azetidyl chloroacetamide series, including selective hits for USP40 and VCPIP1 (Fig. 6a–d). For VCPIP1, small substituents on the benzene ring were well tolerated (V01, V02) while larger functional groups or bulky fused heterocycles abrogated activity (V03, V09). The methylene spacer on the linker was essential for activity, as changes to rigidify this region led to dramatic loss in activity (V15, V16). The two specific changes that drove VCPIP1 potency were addition of halogen substituents to the phenyl ring, such as in the fluorophenyl derivative CAS-11478-188, and integrating the amide into a closed quinazolinone system (V02, V12).

We combined the two productive changes to yield fluoro-quinazolinone compound CAS-12290-201, that inhibited VCPIP1 with an IC_50_ of 70 nM. Kinetic analysis revealed *K*_i_ = 15.3 ±4.6 μM and *k*^inact^ = 0.0792 ± 0.0085 s^−1^ (Fig. 6e). In addition, we confirmed that CAS-12290-201 covalently labeled the catalytic cysteine of VCPIP1 (Supplementary Fig. 8) and selectively bound the native enzyme with little to no activity toward other cellular DUBs (Fig. 6f). Live cell experiments showed little compound toxicity and good target engagement (Supplementary Fig. 8). Together, these data validate CAS-12290-201 as a chemical probe for VCPIP1.

## Discussion

Our results have significant implications for basic DUB biology and drug discovery. Recent trends suggest most new drugs are developed through target-based approaches^16^. While promising in many respects, the low hit-rates for high throughput target-based screens place these technologies, used in combination with ultra-large compound libraries, beyond the reach of all but the largest R&D organizations. In contrast we took a ‘next-generation’ target-class approach to DUB inhibitor development. We eschew agnostic chemical diversity and instead leveraged a structure-guided approach to design a modest-sized library purposely diversified around electrophile and pendant ring system, as well as the linker to build selectivity through multiple sites of DUB-small molecule engagement. As highlighted throughout this report, our use of activity-based protein profiling against endogenous DUBs enabled us to simultaneously deconvolute hits as well as DUB-family SAR in a high-content primary screen. The chemical, biochemical, and chemoproteomic methods we employed are scalable and accessible in a wide range of research environments. In this way our platform democratizes a powerful alternative to the current trend towards massive libraries coupled with ultra-high throughput screening technology.

Efforts in sequencing, genomics, and other systematic molecular analyses continue to highlight DUBs as key nodes in the functional proteome, as well as potential disease vulnerabilities^41^. However, a lack of potent and selective chemical probes represents a significant hurdle in interrogating DUB biology or validating their potential as drug targets. In this respect our results represent a watershed in de-risking DUB inhibitor development. Our combinatorial library diversification strategy yielded selective hits for 23 DUBs spanning 4 DUB families, including examples outside the precedented range of targeted DUBs with foundational chemotypes and for which there are no existing ligands. In fact, for some of the most understudied DUBs our results may motivate development of assays as well as antibodies and other reagents to support comprehensive functional studies. We credit the high hit rate and broad DUB coverage to the success of our rational design strategy; tailoring the screening library to structural elements of the DUB active site, then diversifying against multiple points of target-compound interactions identified in existing inhibitor/DUB co-structures. Additionally, our results provide further evidence that covalent mechanism of action represents a promising path for the development of new selective DUB inhibitors. Our high-content primary screen in which we profiled 178 compounds against 56 DUBs revealed multi-parameter SAR trends which will accelerate future studies. As a powerful example, we leveraged these insights to rapidly optimize a hit for the understudied DUB, VCPIP1, into a potent and selective probe. Importantly, our selective hit-compounds span well-studied DUBs such as UCHL1 as well as some of the most understudied DUBs including USP43, USP53 and OTUD7A. Given the family wide hit diversity, our library provides a multitude of promising chemical starting points and a direct path to tool compounds that can be used to decipher DUB function, ubiquitin substrates, and biochemical pathways. In this way our next-gen target class approach provides a road map for inhibitor development against other understudied proteins or gene-families.

## Methods

### Constructs

UCHL1 (residues 1-223, full length) was cloned into a pGEX6P1 expression vector with an N-terminal GST tag.

UCHL3 (residues 1-230, full length) was cloned into a pET28PP expression vector with an N-terminal 6xHis tag.

USP7 (residues 208–560, catalytic domain) was cloned as described^27^.

USP28 (residues 149-704, catalytic domain) was cloned into a SUMO-pETDUET expression vector with a N-terminal 6xHis-SUMO tag was purchased from Genewiz.

USP30 (residues 65–517, catalytic domain) was cloned into a pET28PP expression vector with an N-terminal 6xHis tag.

OTUD7A (residues1-462, catalytic domain+UBA) in a pOPINK vector with an N-terminal GST tag was purchased from Addgene (#61582).

VCPIP1 (residues 25-561, catalytic domain) in a pOPINK vector with an N-terminal GST tag was purchased from Addgene (#61583).

### Recombinant protein

USP20 (UBI-64-0039-050) and USP27x (UBI-46-0046-050) were ordered from Ubiquigent.

Recombinant USP9x (E-552-052), USP22 (E-608-050), USP15 (E-594-050), and USP48 (E-614-050) were all purchased from R&D Systems, Inc.

### Reagents

Ub-AMC (U-550) and HA-Ub-VS (U-212) were obtained from Boston Biochem.

Bio-Ub-PA (UbiQ-076) and Bio-Ub-VME (UbiQ-054) were obtained from UbiQ Bio.

### Antibodies

USP25 (ab187156) and USP28 (ab188240) antibodies were obtained from abcam. GAPDH (2118s, D4C6R), UCHL1 (13179S), UCHL3 (3525S), USP28 (4217S), USP7 (4833s) antibodies were obtained from Cell Signaling Technology. VCPIP1 (A302-933) and USP48 (A301-190A-M) antibodies were obtained from Bethyl Laboratories.

### Cell culture for ABPP

Cell lines were obtained from ATCC. HEK293T (CRL-3216) cells were cultured in DMEM supplemented with 10% FBS. Cell were maintained in 10 cm tissue-culture treated dishes 37 °C in a 5% CO_2_ incubator. Cells were treated with indicated compounds for the time and amount indicated when relevant.

### DUB activity-based protein profiling primary screening assay

DUB Activity-based protein profiling was performed using conditions modified from those in Schaeur et al., based on work by Lawson *et al*.^20,32^. HEK 293T cells were lysed (50 mM Tris pH 8.0, 150 mM NaCl, 5 mM MgCl_2_, 0.5 mM EDTA, 0.5% NP-40, 10% glycerol, 1 mM TCEP, protease and phosphatase inhibitors) and the lysate was clarified by centrifugation, then diluted to 10 mg/mL. 200 μL aliquots were incubated at the indicated compound concentrations or DMSO for 5 h at RT, final DMSO concentration 0.5%. Afterwards, the treated lysates were incubated with 1 μM each of Biotin-Ub-PA and Biotin-Ub-VME for 90 min at RT. 25 μL magnetic streptavidin sepharose slurry was added to each sample, followed by incubation at RT for 30 min with end-to-end rotation. After immobilizing the beads using a magnetic rack, the supernatant was subjected to an additional streptavidin pulldown as described above, and the pooled beads were washed (3× 0.2% SDS, 3× PBS, 2× ddH_2_O). After the final wash, supernatant was removed, and the resin was flash frozen and stored at −80 °C.

### Sample preparation for mass spectrometry analysis

Streptavidin beads were resuspended in 95 μL 100 mM Tris pH 8.0. Each sample was denatured with 0.1% rapigest, reduced (10 mM dithiothreitol), alkylated (22.5 mM iodoacetamide), and digested with trypsin at 37 °C overnight. The next day, beads were captured using a magnetic rack, and supernatants were acidified with 10% TFA, incubated at 37 °C for 30 min, and centrifuged at 14,100 × *g* for 15 min at 4 °C to remove rapigest. Peptides were then desalted by C18 and dried by vacuum centrifugation.

Dried peptides were reconstituted in 40 μL 50mM pH 8.0 TEAB, and 1/4 unit of TMT reagent was added and reactions incubated at RT for 1 h. TMT reactions were pooled and treated with hydroxylamine according to the manufacturer’s instructions. Peptide mixtures were then dried, reconstituted in 100 mM ammonium bicarbonate and desalted by SP3^42^. Eluted peptides were then analyzed by nanoLC-MS as described in Ficarro et al. with a NanoAcquity UPLC system (Waters, Milford, MA) interfaced to a QExactive HF mass spectrometer (Thermofisher Scientific, San Jose, CA)^30^. TMT labeled peptides were injected onto a precolumn (4 cm POROS 10R2, Applied Biosystems, Framingham, MA), resolved on an analytical column (30 µm I.D. x 50 cm packed with 5 µm Monitor C18) and introduced to the mass spectrometer by ESI (spray voltage = 3.5 kV, flow rate ~30 nL/min). The mass spectrometer was operated in data-dependent mode such that the 15 most abundant ions in each MS scan (*m/z* 300-2000, 120K resolution, target = 3E6, lock mass for 445.120025 enabled) were subjected to MS/MS (m/z 100-2000, 30K resolution, target = 1E5, max fill time = 100 ms). Dynamic exclusion was selected with a repeat count of 1 and an exclusion time of 30 seconds. MS/MS data was extracted to.mgf using mulitplierz scripts and searched against a forward-reverse human NCBI refseq database using Mascot version 2.6.2^43,44^. Search parameters specified fixed cysteine carbamidomethylation, fixed N-terminal and lysine TMT labeling, and variable methionine oxidation. Additional multiplierz scripts were used to filter results to 1% FDR and derive protein-level aggregate reporter ion intensities using peptides mapping uniquely into the genome. Mulitplierz 2.2.0 is available on github as reported^43,44^. Proteins with fewer than two unique peptides were disregarded for quantification due to low signal-to-noise ratio.

% ABP labeling blockage” is calculated by

$$\left(1-\frac{{{{{{\rm{aggregate}}}}}}\, {{{{{\rm{TMT}}}}}}\, {{{{{\rm{reporter}}}}}}\, {{{{{\rm{ion}}}}}}\, {{{{{\rm{intensity}}}}}}\, {{{{{\rm{for}}}}}}\, {{{{{\rm{protein}}}}}}\, {{{{{\rm{in}}}}}}\, {{{{{\rm{condition}}}}}}}{{{{{{\rm{average}}}}}}\, {{{{{\rm{aggregate}}}}}}\, {{{{{\rm{TMT}}}}}}\, {{{{{\rm{reporter}}}}}}\, {{{{{\rm{ion}}}}}}\, {{{{{\rm{intensity}}}}}}\, {{{{{\rm{for}}}}}}\, {{{{{\rm{protein}}}}}}\, {{{{{\rm{in}}}}}}\, {{{{{\rm{DMSO}}}}}}\, {{{{{\rm{controls}}}}}}}\right)\times 100\%$$

### Protein expression

All constructs were overexpressed in E. coli BL21 (DE3). Cells were grown at 37 °C to an OD of 0.9, cooled to 16 °C, induced with 500μM isopropyl -1-thio-D-galactopyranoside (IPTG), incubated overnight at 16 °C, collected by centrifugation, and stored at −80 °C. Cell pellets were sonicated in lysis bufer (25 mM Tris pH 8, 1 M NaCl, and 10 mM BME) supplemented with 10 μg/ml phenylmethanesulfonylfuoride (PMSF) and the resulting lysate was centrifuged at 30,000 × *g* for 40 min. Lysate from His-tagged proteins were mixed with Ni-NTA beads (Qiagen) 2 h, and washed with lysis buffer supplemented with 25 mM imidazole. The bound protein was eluted with lysis buffer supplemented with 300 mM imidazole.

Lysate from GST-tagged proteins were mixed with glutathione beads (company) for 2 h, washed with lysis buffer, and eluted overnight with 3C protease. The samples were then concentrated to 1 ml (30 kDa concentrator; Amicon Ultra, Millipore), and run on a Superdex 200 (GE healthcare) in buffer containing 25 mM HEPES pH 7.5, 200 mM NaCl, and 1mM DTT. Fractions were pooled, concentrated and frozen at −80 °C.

### Biochemical Assays

Enzymes were tested for activity in Ubiquitin-Rhodamine assay in the presence or absence of inhibitors. Enzyme (UCHL1: 2nM; UCHL3: 200pm; USP7: 10nM; USP28: 5nM; USP48: 10nM; VCPIP1: 100nM, JOSD1: 25nM, OTUD7A: 50nM, USP15: 0.1nM, USP9X:0.1nM, USP27X: 125nM, USP20: 1nM, USP21: 2nM) was pre-incubated for 6 h at room temperature with different concentrations of inhibitors or DMSO as a control in 50mM TRIS pH 8, 0.5 mM EDTA, 10 µM ovalbumin, and 5mM TCEP. Ubiquitin-Rhodamine (Boston Biochem) was then added to a final concentration of 500nM. The initial rate of the reaction was measured by collecting fluorescence data at 1-min intervals over 30-min to 1-h period using a Clariostar fluorescence plate reader at excitation and emission wavelength of 345 and 445nm, respectively. The calculated initial rate values were plotted against inhibitor concentrations to determine IC50s. All the experimental data were plotted using GraphPad Prism. All assays for each compound were performed at least twice for each compound.

### *k*_inact_/*K*_i_ determination

*k*_inact_/*K*_i_ determination was carried out as described in Turnbull et al, at the enzyme and inhibitor concentrations listed^45^. Briefly, upon addition of the substrate, fluorescence intensity was monitored kinetically every 30 s over 1 h. Using GraphPad Prism, raw fluorescence data was plotted as a function of time for each concentration. Data was normalized by treating 0 as smallest value and 100 as value>>largest value (set to 100,000). Baseline background fluorescence from no-protein wells was subtracted from each reading. Normalized and baseline corrected kinetic progress curves were fitted to equation (2) *y* = *y*_max_(1−exp(−*k*_obs_.*x*)) for *k*_obs_. *k*_obs_ was then plotted against the inhibitor concentrations and fitted to the equation (3) *y* = *k*_inact_/(1+(*K*_i_/*x*)) for *k*_inact_ and *K*_i_.

### Biochemical selectivity profiling

Selectivity profiling (DUB*profiler*^TM^) was performed by Ubiquigent with a panel of 41 purified DUBs and ubiquitin-rhodamine(110)-glycine as a fluorescent substrate. Single-dose percentage activity inhibition were determined after 15 min compound pre-incubation. Samples were prepared and sent according to protocol (https://www.ubiquigent.com/drug-discovery-screening-platform/dubprofiler/).

### Live cell compound treatment

For experiments shown in Supplementary Fig. 5b, live HEK293 cells were treated at the indicated concentrations overnight. Subsequent lysis and workflow is detailed in the section “DUB ABP Labeling for Western blot target engagement” below.

### DUB ABP labeling for Western blot target engagement

Western blot ABPP target engagement experiments were performed as previously described in Lamberto et al^27^. Briefly, target engagement lysis buffer (50 mM Tris pH 8.0, 150 mM NaCl, 5 mM MgCl_2_, 0.5 mM EDTA, 0.5% NP-40, 10% glycerol, 1 mM TCEP, protease and phosphatase inhibitors) was added to cell pellets on ice. Lysate was cleared by centrifugation and diluted to 2 mg/mL. Where indicated, 30 μL lysate was then incubated with inhibitors or DMSO for the indicated time points. 2 μM Flag-Ub-PA was then added to the lysate and incubated at RT for the indicated time points. Labeling reactions were quenched with 4x LDS sample buffer (Termo Fisher B0007) supplemented with 10% BME, vortexed vigorously, and heated to 95 °C for 5 min. Samples were resolved by SDS-PAGE and analyzed by Western blot with the indicated primary antibodies at 1:1000 dilution.

Where relevant, desitometry was carried out with ImageJ; a rectangular window was defined using the upper ABP-labeled band on the DMSO+ABP lane, and was used for quantifying the ABP-labeled band in all other lanes/conditions. Invert values were obtained with 255-mean, and background taken from the no probe lane was subtracted from each row. Percentage blockage was calculating by dividing the invert of each lane with the invert of the DMSO+ABP condition for % labeled, then subtracting the % labeled value from one.

### Cell viability testing

Cell viability testing was carried out in 96-well plates with 8x replicates of DMSO controls and 4x replicates per compound on each plate. 1E3 live cells/well were treated at 0.25×, 0.5×, 1×, 2×, and 4× biochemical IC50 for target DUB over 24 h. Cell viability was assessed using CellTiter-Glo Luminescent Cell Viability Assay (Promega) per manufacturer instructions on a CLARIOstar Plate Reader (BMG Labtech). Data was plotted using GraphPad Prism 9.0.1.

### Intact MS analysis

five microgram of indicated DUBs were treated with DMSO or a 10-fold molar excess of compound for 1 h. Reactions were then injected onto a self-packed reversed phase column (1/32″ O.D. × 500 μm I.D., 5 cm of POROS 10R2 resin), desalted, and eluted with an HPLC gradient (0-100% B in 4 min, A=0.2M acetic acid in water, B = 0.2 M acetic acid in acetonitrile, flow rate ~30 µL/min) into an LTQ ion trap mass spectrometer (ThermoFisher Scientific, San Jose, CA). Profile mass spectra (*m/z* 300–2000) were deconvoluted using MagTran1.03b2 software^46^.

### CE-MS analysis

To identify sites of covalent modification, treated protein was reduced (10 mM TCEP), alkylated (22.5 mM MMTS), and digested with trypsin overnight at 37 °C. Peptides were desalted using SP3, dried by vacuum centrifugation, and reconstituted in 1% formic acid/50% acetonitrile with 100 mM ammonium acetate^42^. Peptides were then analyzed by CE-MS using a ZipChip CE system and autosampler (908 Devices, Boston, MA) interfaced to a QExactive HF mass spectrometer (ThermoFisher Scientific, San Jose, CA). Peptide solution was loaded for 30 seconds, and the mass spectrometer was operated in data-dependent mode and subjected the 5 most abundant ions in each MS scan (60k resolution, 3E6 target, lock mass enabled) to MS/MS (15k resolution, 1E5 target, 100 ms max inject time). Dynamic exclusion was enabled with a repeat count of 1 and an exclusion time of 6 seconds. MS/MS data was extracted to.mgf using mulitplierz scripts and searched against a forward-reverse human NCBI refseq database using Mascot version 2.6^43,44^. Search parameters specified fixed carbamidomethylation of cysteine, and variable oxidation (methionine) and compound modification. Precursor mass tolerance was set to 10 ppm and product ion tolerance was 25 mmu. Spectral validation was performed using mzStudio^47^.

### Competition with biotinylated inhibitor analog for global off-target profiling

HEK 293T cells were lysed as described above, and the lysate was cleared by centrifugation. Samples were diluted to 10 mg/mL, and 200 μL lysate (2 mg protein total) was incubated with the indicated concentrations of F70 for 4 h at RT, then 2 μM of DTB-F-70 for 4 additional hours. SDS was added to a final concentration of 1.2% and the sample was boiled for 5 min. After cooling to RT, DPBS was added to dilute SDS concentration to a final of 0.2%. 50 μL streptavidin agarose slurry was added to each sample, followed by incubation at RT for 90 min. After streptavidin enrichment, samples were washed (3× 0.2% SDS, 3× PBS, 2× ddH2O). After the final wash, all supernatant was removed and the resin was flash frozen and stored at −80 °C until workup for TMT labeling. See “Sample Prep for Mass Spectrometry Analysis” section in the “Methods" section of the main text for further steps.

### Targeted PRM-LIVE analysis of VCPIP1 catalytic cysteine-containing peptide

The tryptic peptide for VCPIP1 spanning the catalytic cysteine, _**204**_SQECLIPVHVDGDGHCLVHAVSR^**226**^ (*R* = ^15^N-4, ^13^C-6), was synthesized using Fmoc chemistry and HPLC purified. Cysteines were then alkylated with iodoacetamide, and amine terminus was labeled with TMT-131C according to the manufacturer’s instructions. After desalting, the peptide was aliquoted and stored at −80 °C.

Following the enrichment of cysteine peptides from HEK293 cells described above, 200 ng of synthetic VCPIP1 peptide was spiked into 150 μg of enriched cysteine peptides. The peptide mixture subjected to offline high pH fractionation. Briefly, peptide was loaded onto a self-packed analytical column (500μm I.D. fused silica packed with 10 cm of 5μm Xbridge C18) and eluted using an LC gradient (10ul/min flow rate, 5–35% B in 60 min; *A* = 20mM Ammonium Formate pH 10 with 2% acetonitrile, B=acetonitrile). A total of 65 fractions were collected every 1 min on an automated fraction collection platform^48^. Approximately 10% of each fraction were analyzed by MALDI to identify the fraction containing the spiked synthetic VCPIP1 peptide (‘VCPIP1 fraction’). The VCPIP1 fraction was removed and split into two equal aliquots, one for targeted PRM-LIVE MS/MS and the second for data-dependent PASEF MS/MS analysis (described below). The remaining 64 fractions were combined in sets of two according to an n,n+32 scheme, and also analyzed by data-dependent PASEF MS/MS.

Cysteine peptides were analyzed by PASEF on a timsTOF Pro ion mobility mass spectrometer (Bruker, Billerica, MA) coupled to a NanoElute UPLC system (Bruker, Billerica, MA). Peptides were loaded on a trapping column (100 μm × 2 cm, 5 μm C18) and eluted to a self-packed analytical column (75 μm I.D. fused silica packed with 25 cm of 1.9 μm Reprosil C18)^30^ using an LC gradient (2%-35% B in 90 min; A=water with 0.1% formic acid, B=acetonitrile with 0.1% formic acid; spray voltage = 1600V). The mass spectrometer collected ion mobility MS spectra over a mass range of *m/z* 100–1700 and 1/k0 of 0.6 to 1.6, and then performed 10 cycles of PASEF MS/MS with a target intensity of 14.5k and a threshold of 1750. The experiment targeting the VCPIP1 endogenous and synthetic peptides was performed using PRM-LIVE^39^ (+3 to +5 charge states for each peptide, ±0.025 V s/cm^2^, isolation width 0.7 Da, tims stepping enabled). In this experiment, peptides were loaded directly on the analytical column and eluted with an LC gradient (2–17% B in 90 min, 17–25% B in 45 min, 25–37% B in 30 min, 37–80% B in 22.5 min, 80–95% B in 1.5 min; A = water with 0.1% formic acid, B = acetonitrile with 0.1% formic acid; spray voltage = 1600 V).

PRM and DDA PASEF raw data files were searched using MSfragger^49^ against a swiss-prot human protein database with 20386 entries, according to previous methods^39^. The search parameters used trypsin as the protease with 2 missed cleavages allowed, precursor mass tolerance of 20 ppm, and product ion tolerance of 0.05 Da. Modifications included variable methionine oxidation, fixed cysteine carbamidomethylation, and fixed lysine and N-terminal TMT labeling. A false discovery rate of 1% was used as a cutoff for peptide identification.

Data were further processed using in-house scripts to extract TMT reporter ion intensities for each PSM. For the PRM run, PSMs spanning the full-width-half-max of the VCPIP1 peptide elution profile were combined and reporter intensities were compared to determine a relative fold-change between the control (DMSO) and treatment (WH-9943-103C) conditions. Search results for DDA PASEF MS/MS analysis from the other peptide fractions were combined for statistical analysis. We excluded PSMs with a missing value in any of the TMT reporter channels. The remaining PSMs were corrected for TMT isotopic impurities. In cases where multiple spectra matched to the same combination of sequence, modification, and charge state, we selected the PSM with the highest summed intensity across the two DMSO control channels (TMT126 and TMT129). Impurity-corrected reporter intensities less than 0 were replaced with an intensity value of 12, which was empirically determined to be the ~3 times the noise level in the *m/z* region of the TMT reporters. Next, intensity values were median normalized across channels. Pairs of median intensity values for the three replicate channels (TMT126-TMT129, TMT127-TMT130 and TMT128-TMT131) were used as input for a maximum likelihood estimation of parameters to derive an intensity-dependent error model and p-values for DMSO:inhibitor-treated ratio^50^. Finally, we used the Benjamini-Hochberg procedure to adjust the p-values and control the false discovery rate to 1%^51^.

### Pubmed publication analysis

The number of publications per DUB was generated using Pubmed’s search function. A search filter for journal articles was applied, and results were sorted by publication date. Uniprot gene names were used for each search, with the following additions for DUBs which were recently renamed or were commonly referred to with alternative names: FAM188A/MINDY3, FAM188B/MINDY4, FAM63A/MINDY1, FAM63B/MINDY2, VCPIP1/VCIP135, UCHL1/PGP9.5, USP7/HAUSP.

### Reporting summary

Further information on research design is available in the Nature Portfolio Reporting Summary linked to this article.

## Supplementary information

Supplementary Information

Description of Additional Supplementary Files

Supplementary Dataset 1

Supplementary Dataset 2

Supplementary Dataset 3

Supplementary Dataset 4

Supplementary Dataset 5

Reporting Summary

## Data Availability

The raw mass spectrometry data generated in this study has been deposited in the MassIVE public repository under accession code: MSV000088637 [10.25345/C51K2F]. Raw Western blot images, biochemical assay readings, and viability data are provided in the Source Data file. Protein structures used in Fig. 1 can be access through the PDB under the accession codes 5VS6 [10.2210/pdb5vs6/pdb] and 1NBF [10.2210/pdb1nbf/pdb]. Source data are provided with this paper.

## Source data

Source Data
