# Supplementary material for: Accelerating inhibitor discovery for deubiquitinating enzymes
Source: Nat Commun. 2023 Feb 8;14:686. doi: 10.1038/s41467-023-36246-0 (PMC9908924; doi:10.1038/s41467-023-36246-0)
Supplement: Supplementary file 1 — Supplementary Information [file 41467_2023_36246_MOESM1_ESM.pdf]

Accelerating Inhibitor Discovery for Deubiquitinating Enzymes

Wai Cheung Chan [1,2#], Xiaoxi Liu [1,2#], Robert S. Magin [1,2], Nicholas M. Girardi [1,2], Scott B. Ficarro [1,3,4], Wanyi Hu [1,2], Maria I. Tarazona Guzman [1,2], Cara A. Starnbach [1,2], Alejandra Felix [1,2], Guillaume Adelmant[1,3], Anthony C. Varca[1,2], Bin Hu [1,2], Ariana S. Bratt [1,2], Ethan DaSilva [1,2], Nathan J. Schauer [1,2], Isabella Jaen Maisonet [1,2], Emma K. Dolen [1,2], Anthony X. Ayala [1,2], Jarrod A. Marto [1,3,4,5*], Sara J. Buhrlage [1,2,4,5]*.

[1] Department of Cancer Biology and the Linde Program in Cancer Chemical Biology, Dana-Farber Cancer Institute, Boston, MA, USA.

[2] Department of Biological Chemistry and Molecular Pharmacology, Harvard Medical School, Boston, MA, USA.

[3] Blais Proteomics Center, Dana-Farber Cancer Institute, Boston, MA, USA.

[4] Center for Emergent Drug Targets

[5] Department of Pathology, Brigham and Women's Hospital and Harvard Medical School, Boston, MA, USA.

[*] These authors jointly supervised this work

# These authors contributed equally

*Correspondence: jarrod_marto@dfci.harvard.edu; saraj_buhrlage@dfci.harvard.edu

Content

(A) Supplementary Figures

(B) Supplementary Methods

(C) References

Supplementary Figures

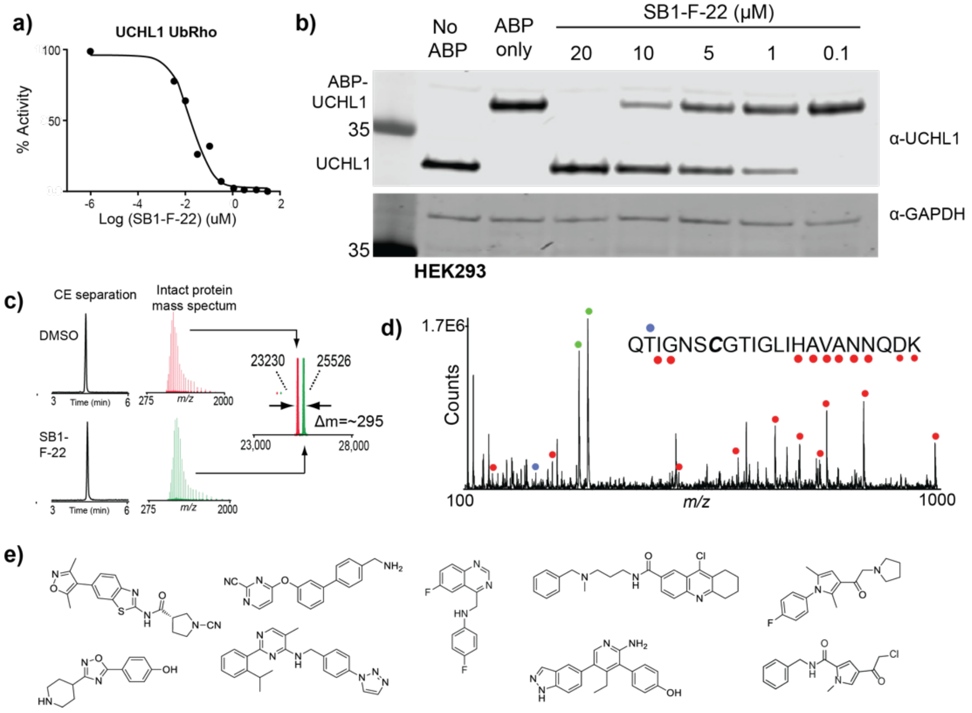

Supplementary Figure 1. Designing the DUB-targeted covalent small molecule library. a) F22 inhibits UCHL1 in a fluorescent Ub-Rho DUB activity assay with an IC_{50} of 100nM. (n=1) b) F22 blocks DUB ABP labelling of UCHL1 in KMS11 cell lysates. c) F22 labels purified UCHL1 with 1:1 stoichiometry. d) F22 labels UCHL1 at the catalytic cysteine residue. Red and blue glyphs adjacent to the peptide sequence indicate y- and b-type fragment ions, respectively, detected in the MS/MS spectrum. The y-ion series along with inhibitor structure-specific fragment ions (green glyphs) confirms catalytic cysteine modification (***C***). e) Structures of existing DUB inhibitors which are taken into consideration in the design of the DUB-targeted covalent small molecule library. Source data are provided as a source data file.

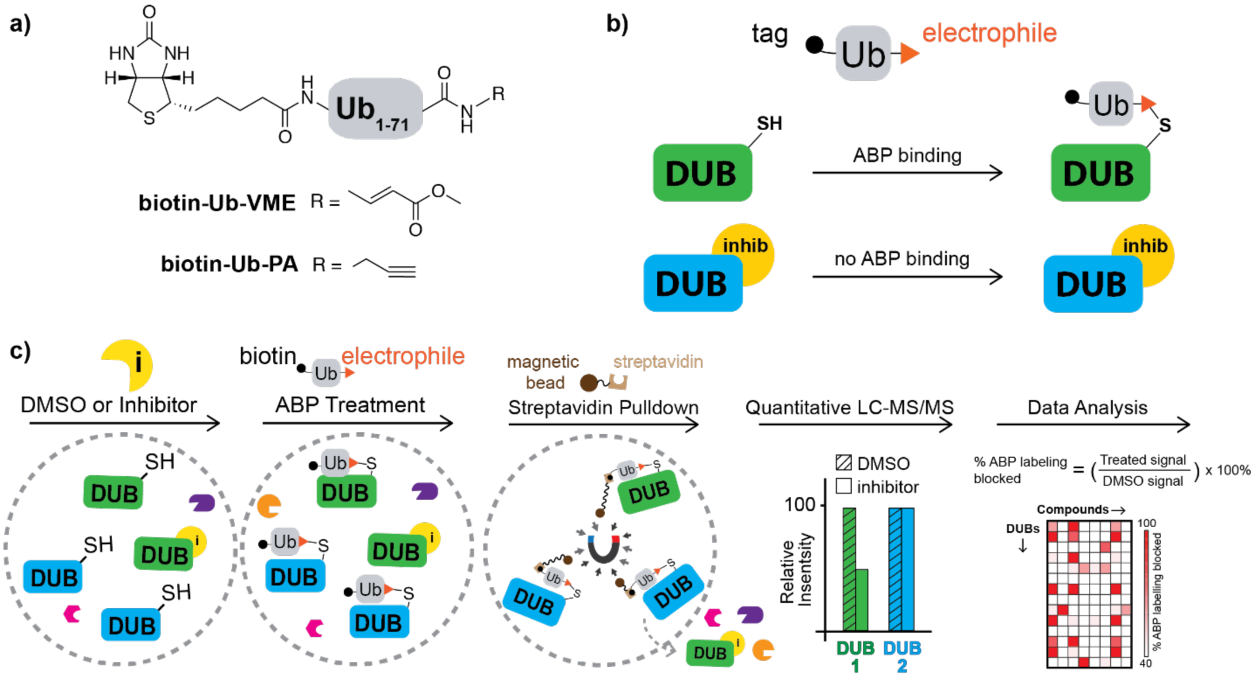

Supplementary Figure 2. Primary Screening assay. a) The ABPs used in this study are a 1:1 mix of biotin-ubiquitin-vinyl methyl ester (biotin-Ub-VME) and biotin-ubiquitin-propargyl amide (biotin-Ub-PA), with structures shown. b) ABPs only react with catalytic cysteine residues on active DUBs, thereby ligating the biotin enrichment tag onto the DUB. Inhibitor-bound DUBs do not react with the ABP and cannot be enriched through streptavidin pulldown. c) Schematic for the MS-ABPP primary screening assay.

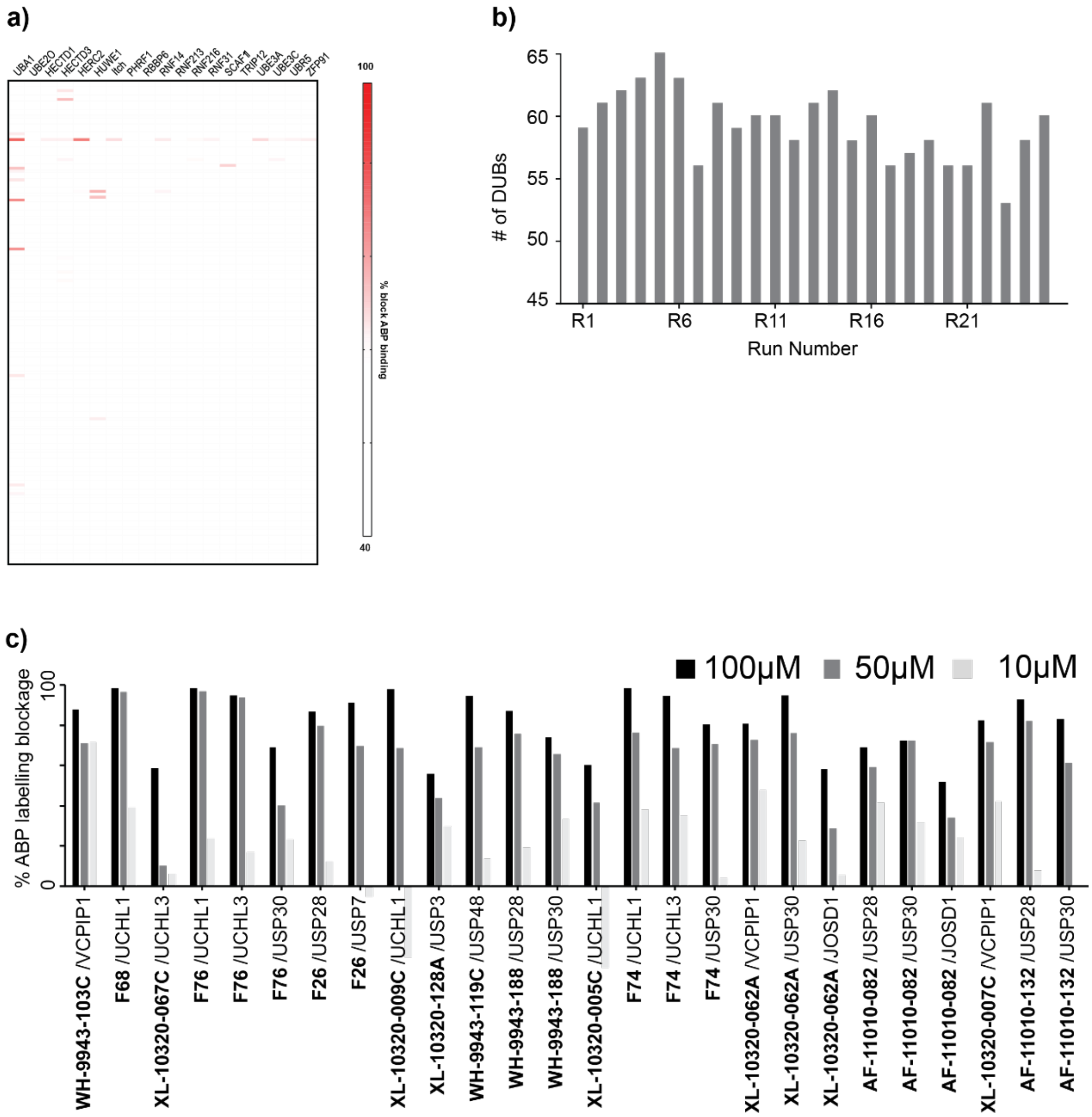

Supplementary Figure 3. Additional Primary Screening data analysis. a) Library compounds showed little activity against E1, E2, E3 enzymes. b) The number of DUBs detected in each 11-plex MS-ABPP run in the primary screening assay. c) Inhibitors were successfully validated over a three-point dose curve in the MS-ABPP primary screening assay.

Supplementary Table 1. Frequency of detection across 25 acquisitions for each DUB detected in primary screening

DUB	Frequency	DUB	Frequency
CYLD	25	USP5	25
USP10	25	USP51	1
USP11	25	USP53	1
USP12	15	USP54	18
USP13	2	USP7	25
USP14	25	USP8	25
USP15	25	USP9X	25
USP16	25	USP9Y	1
USP19	25	BAP1	25
USP2	24	UCHL1	25
USP20	25	UCHL3	25
USP21	25	UCHL5	25
USP22	25	OTUB1	25
USP24	25	OTUB2	25
USP25	25	OTUD1	25
USP27X	21	OTUD3	25
USP28	25	OTUD4	25
USP3	22	OTUD5	25
USP30	25	OTUD6B	25
USP31	25	OTUD7A	25
USP32	25	OTUD7B	25
USP33	25	OTULIN	12
USP34	25	VCPIP1	25
USP35	22	YOD1	25
USP36	25	ZRANB1	25
USP37	25	ZUP1	15
USP38	25	ATXN3	25
USP4	25	JOSD1	25
USP40	25	JOSD2	25
USP42	25	FAM188A	25
USP43	24	FAM188B	25
USP46	23		
USP47	25		
USP48	25		

Supplementary Table 2. Biochemical validation of most potent and selective screen hits, IC50 values shown for biochemistry heatmap in figure 4b in blue. Source data are provided as a Source Data file.

Hit	ABPP Target	Biochemistry IC50 (µM)						
		VCPIP	UCHL1	UCHL3	USP7	USP28	USP48	JOSD1
WH_9943_107C	VCPIP1	1.2	>100	>100	>100	>100	>100	45.8
WH_9943_103C	VCPIP1	2.0	>100	>100	>100	>100	>100	44.1
XL_10320_062A	VCPIP1	3.0	>100	>100	>100	>100	>100	81.0
XL_10320_009C	UCHL1	>100	6.6	>100	>100	62.0	87.2	78.7
XL_10320_005C	UCHL1	33.7	3.5	42.6	>100	>100	>100	>100
F68	UCHL1	>100	3.4	>100	>100	69.1	78.0	38.6
F59	UCHL1/USP10	>100	0.5	>100	>100	>100	>100	>100
XL_10320_67C	UCHL3	14.9	18.1	6.3	>100	>100	>100	>100
F76	UCHL3/1	>100	0.1	1.0	>100	41.2	81.6	31.5
WH_9943_119C	USP48	>100	>100	>100	>100	>100	9.2	29.0
AC19	USP27X	>100	>100	58.4	29.5	40.1	8.6	15.2
F26	USP7/USP28	>100	>100	92.0	66.9	18.5	>100	9.2
WH_9943_188	USP28	>100	>100	>100	>100	17.5	>100	12.3
AF132	USP28	>100	>100	59.0	>100	27.9	>100	3.7
815687	USP3	>100	>100	>100	>100	59.8	>100	75.2
510381	USP3	>100	>100	>100	>100	>100	>100	>100
XL_9872_163	USP3	>100	>100	>100	>100	>100	>100	>100
XL_9872_126A	USP3	41.8	>100	>100	59.4	80.8	>100	43.5
XL_9872_123A	USP3	>100	>100	>100	>100	>100	94.5	48.4
AF_11010_112	USP21/31	>100	>100	>100	>100	>100	>100	>100
F45	USP21	>100	81.0	>100	>100	>100	>100	>100
F46	Multitargeted	>100	9.9	20.1	25.8	8.1	41.5	4.9
AF124	Multitargeted	12.3	10.0	8.6	22.5	4.4	>100	0.9
XL-9872-111B	JOSD1, USP30	14.5	0.3	7.8	5.7	5.3	21.1	2.6

Supplementary Table 3. Biochemical validation of less potent screen hits (n=1). Source data are provided as a source data file.

Compound ID	Target	IC50 (µM)
WH_9943_104B	USP15	>100
XL_9678_185C	USP20	>100
AF-11010-112	USP21	37.4
F45	USP21	27.8
XL_10320_009A	USP22	94.4
XL_10320_028A	USP22	86.5
AC19	USP27X	81.1
AV46	USP27X	35.9
ED27	USP27X	>100
WH_9943_107C	USP27X	>100
bin-01-25	USP9X	>100
Bin-01-36	USP9X	51.4
WH_9943_119C	USP48	7.4

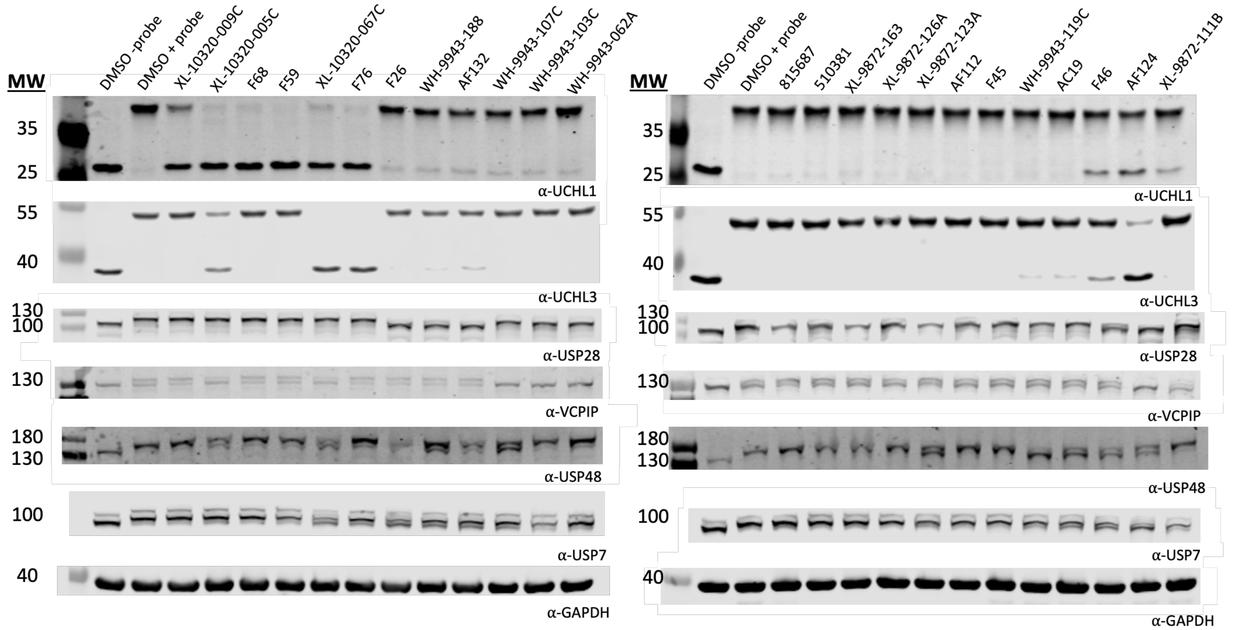

Supplementary Figure 4. Western blot target engagement. Similarly to table 1, in-lysate target engagement was examined by western blot for 24 compounds at 50μM against a set of 6 DUBs. ABP-bound DUBs display a +10kDa shift, while compounds which block probe labelling abolish this shift. Negative and positive controls without probe and with probe only are shown in the first two columns. Loading control against GAPDH is shown in the bottom row. Source data are provided as a Source Data file.

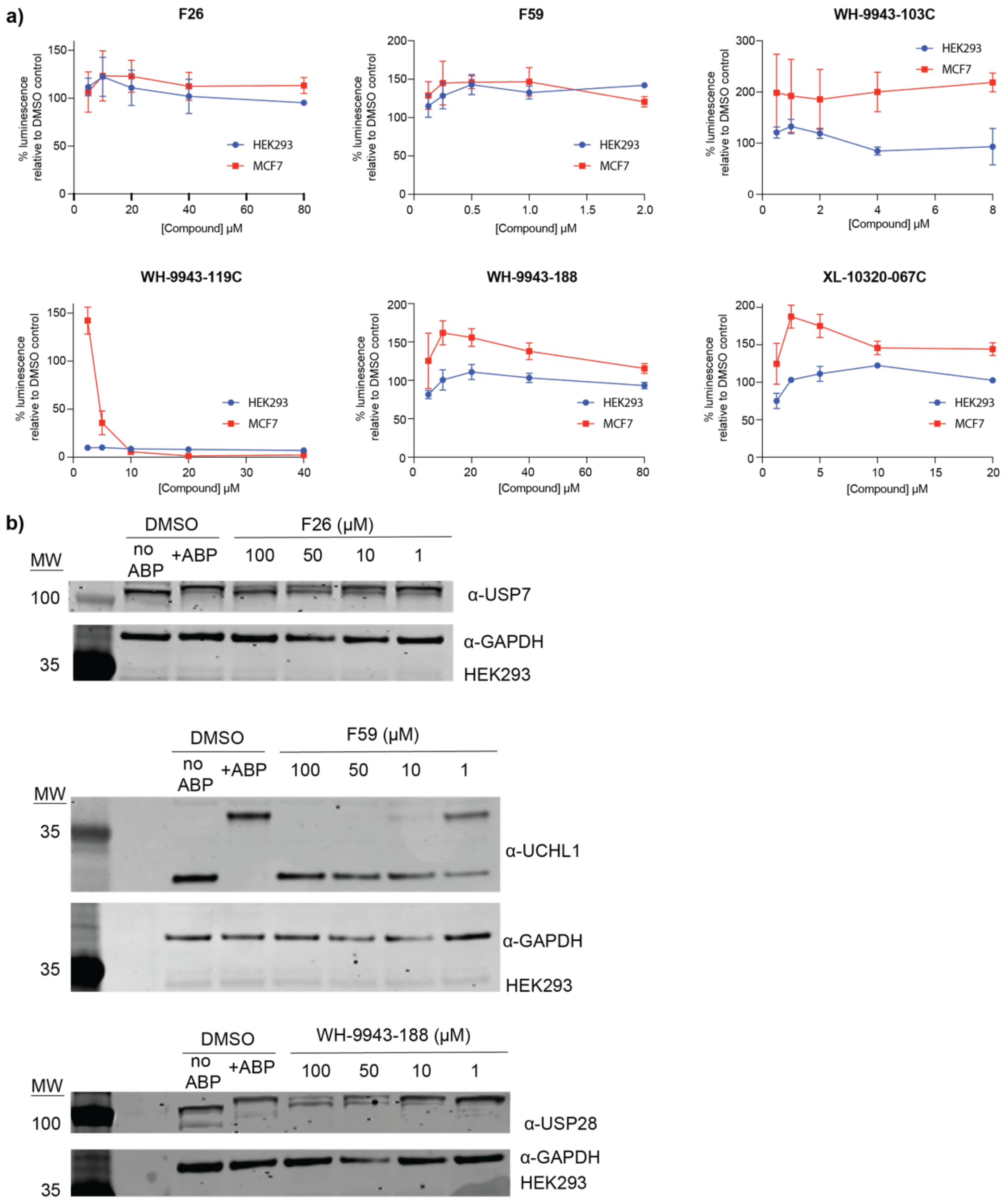

Supplementary Figure 5. Cell viability testing and live cell target engagement. a) Minimal cellular toxicity was observed for a selected panel of six compounds, each targeting a different DUB (n=8 for DMSO controls, n=4 for compound treatment where n is the number of biologically independent experiments) in both HEK293T and MCF7 cells. 1E3 live cells/well were treated at 0.25x, 0.5x, 1x, 2x, and 4x biochemical IC50 for target DUB over 24 hours, after which viability was assessed using CellTiter-Glo. Error bars represent standard deviation. b) Live cell target engagement was confirmed by western blot for 4 compounds, each targeting a different DUB, at the concentrations shown. Data for the 4th compound, WH-9943-103C is shown in main figure 5c. ABP-bound DUBs display a +10kDa shift, while compounds which block probe

labelling abolish this shift. Negative and positive controls without probe and with probe only are shown in the first two columns. Loading control against GAPDH is shown in the bottom row. Source data are provided as a Source Data file.

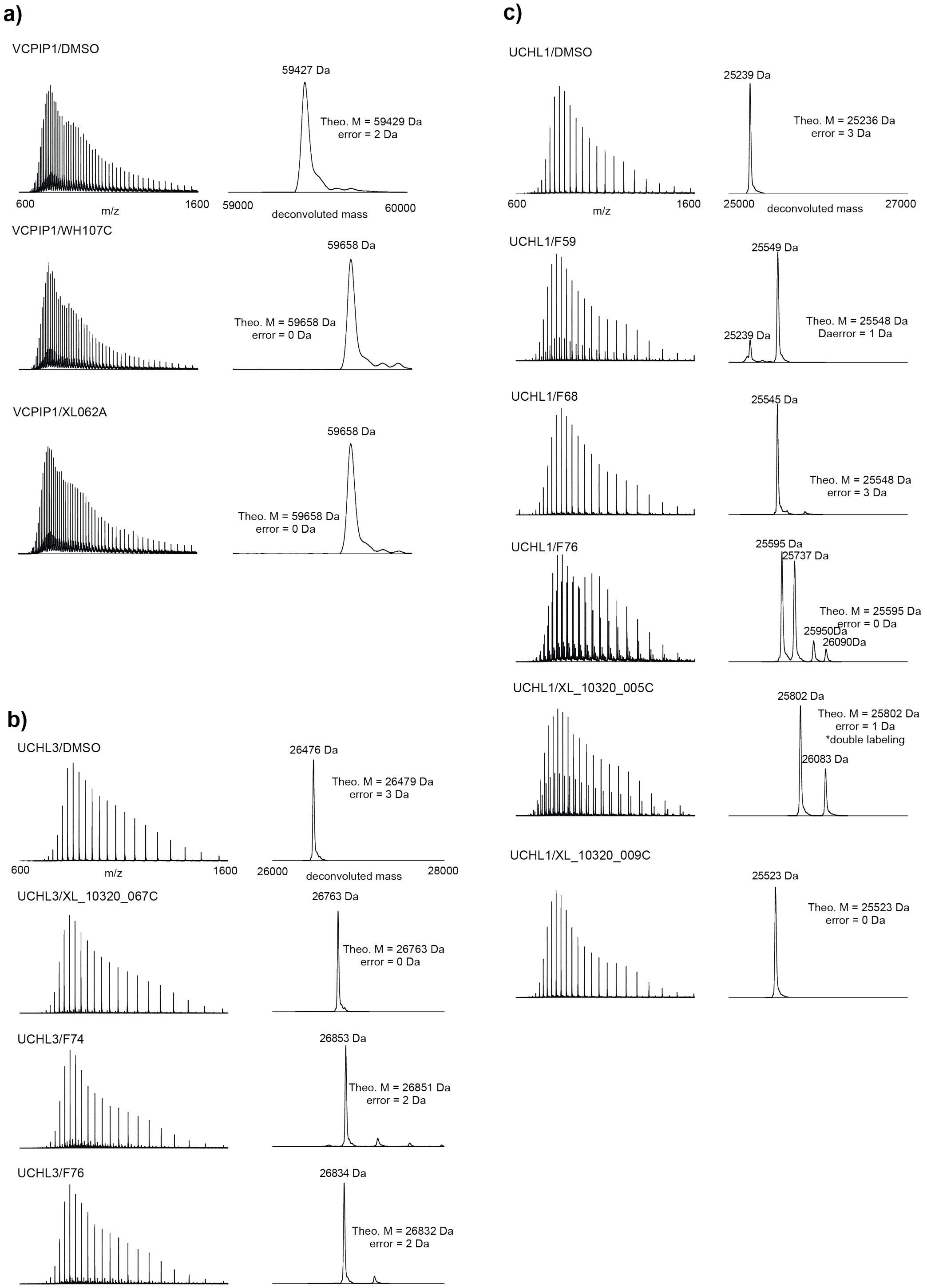

Supplementary Figure 6. Intact protein mass spectrometry validation for selected DUB/compound pairs. The indicated DUBs were incubated with most potent and selective hits at 5-10 fold molar excess, then analyzed by LC-MS. Raw mass spectra shown on the left of deconvoluted mass. Compounds which displayed hyperlabelling were dropped from further consideration. Observed protein masses were consistent with theoretical masses within instrument error.

Supplementary Table 4. Peptide-level CE-MS for selected DUB/compound pairs. DUB-compound complexes were digested with trypsin then analyzed by CE-MS. Cysteines labelled are listed, with catalytic cysteine residues in bold.

DUB	Compound	Cysteine(s) labelled
UCHL1	F59	**C90**
	F68	**C90**
	F76	**C90**, C132
	XL-10320-005C	**C90**, in addition to 5 other Cys sites
	XL-10320-009C	**C90**
UCHL3	XL-10320-067C	**C95**, C209
	F74	**C95**, C209
	F76	**C95**, C209
VCPIP1	WH-9943-107C	**C219** in addition to 9 other Cys sites
	XL-10320-062A	**C219** in addition to 7 other Cys sites
USP28	AF-11010-124	**C171**
	AF-11010-132	**C171** in addition to 2 other Cys sites
	WH-9943-188	**C171** in addition to 3 other Cys sites
USP48	WH-9943-119C	**C98** in addition to 15 other Cys sites

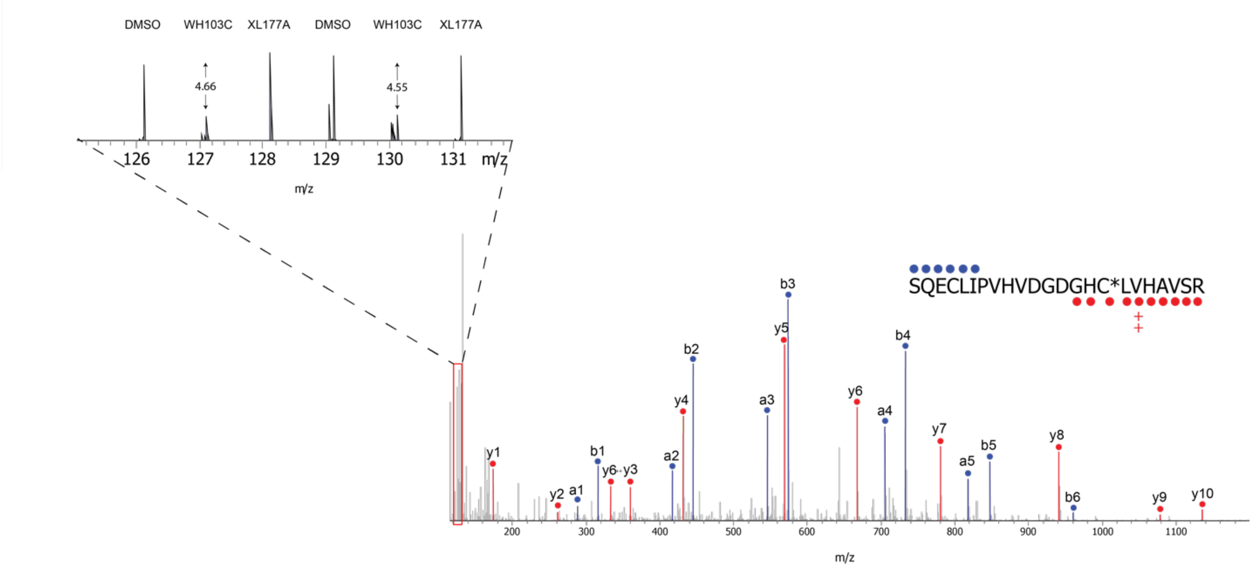

Supplementary Figure 7. Targeted PRM-LIVE experiment for VCPIP1 catalytic cysteine-containing peptide. MS/MS spectrum of the VCPIP1 catalytic cysteine containing peptide. Red and blue glyphs adjacent to the peptide sequence indicate y- and b-type fragment ions, respectively. y-ion series confirms catalytic cysteine modification (C*). *inset:* TMT reporter ion intensities for each condition shown.

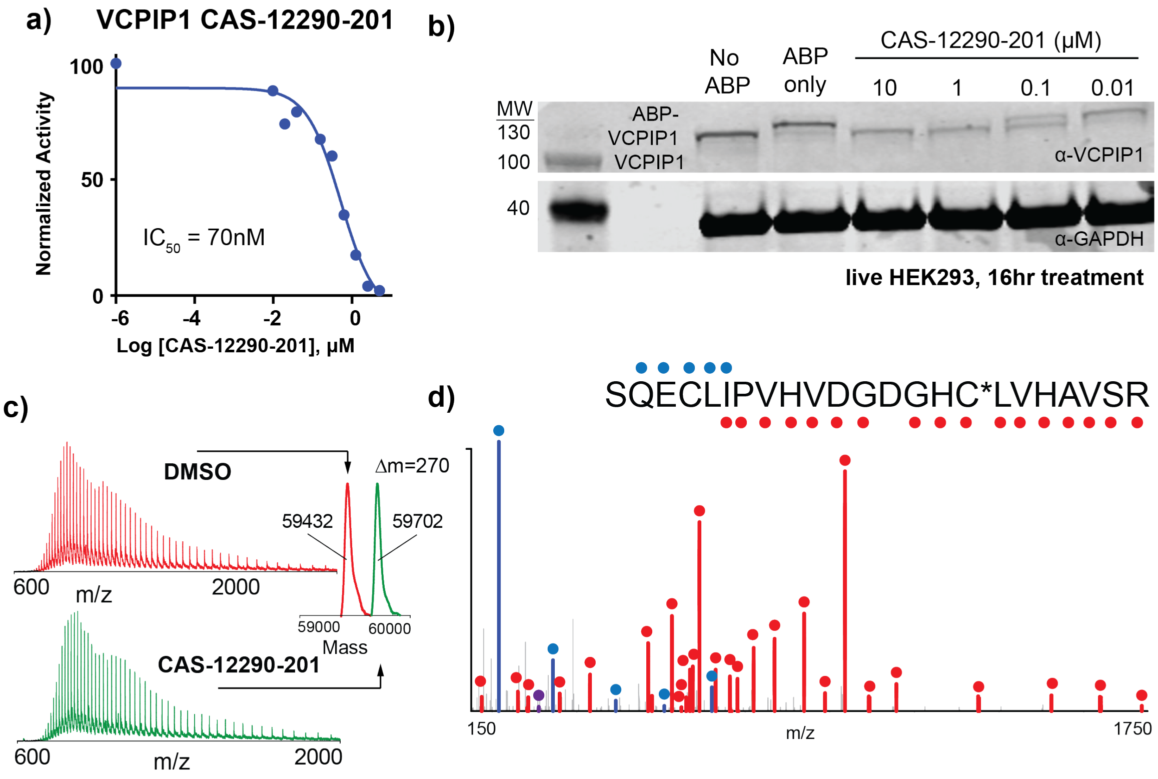

Supplementary Figure 8. Characterization of CAS-12290-201. a) CAS-12290-201 inhibits VCPIP1 catalytic activity at IC_{50} = 70 nM in a ubiquitin-rhodamine fluorescent activity assay. b) CAS-12290-201 displaces ABP binding to VCPIP1 in live 293T cells (16 hours treatment) in a dose-dependent fashion as read out by Western blot. c) CAS-12290-201 labels purified VCPIP1 protein 1:1. d) CAS-12290-201 labels VCPIP1 at the catalytic cysteine residue, C219. Red and blue glyphs adjacent to the peptide sequence indicate y- and b-type fragment ions, respectively, detected in the MS/MS spectrum. The y-ion series confirms catalytic cysteine modification (C*). Source data are provided as a Source Data file.

Supplementary Table 5. Small molecule screening data

Category	Parameter	Description
Assay	Type of assay	Lysate activity-based protein profiling
	Target	Deubiquitinating enzymes
	Primary measurement	TMT reporter ion intensity, summed across peptides for each DUB
	Key reagents	Activity-based probes Biotin-Ub-PA (UbiQ-076) Biotin-Ub-VME (UbiQ-054) were obtained from UbiQ Bio
	Assay protocol	Please see methods section, titled "DUB Activity Based Protein Profiling Primary Screening Assay"
	Additional comments	
Library	Library size	178 compounds
	Library composition	DUB active-site cysteine directed covalent small molecules
	Source	In-house synthesis, a small number of reporter DUB inhibitors
	Additional comments	Please see supplementary methods in supplementary information 1 for synthetic procedure
Screen	Format	1.5mL microcentrifuge tubes, 11-plex TMT experiment
	Concentration(s) tested	50μM for primary screening
	Plate controls	50μM XL177A (USP7 covalent inhibitor) used as a positive control
	Reagent/ compound dispensing system	Manual
	Detection instrument and software	Thermo Q Exactive HF mass spectrometer, Thermo Scientific
	Assay validation/QC	See Figure 2a
	Correction factors	
	Normalization	Signal for each TMT channel normalized by total summed protein signal
	Additional comments	
Post-HTS analysis	Hit criteria	>50% blockage of ABP labelling, as read out by reduction in MS signal from DMSO control to compound-treated condition
	Hit rate	60%
	Additional assay(s)	Dose response MS-ABPP for initial validation, Ub-AMC biochemical DUB fluorescent activity assay, intact protein mass spectrometry for covalent labelling, peptide CE-MS to identify site of modification, Western blot ABPP to confirm target engagement, live cell treatment to eliminate compound toxicity
	Confirmation of hit purity and structure	Please see supplementary methods in supplementary information 1
	Additional comments	

Supplementary Methods

Abbreviation

Et_3N: Triethylamine

HATU: 1-[Bis(dimethylamino)methylene]-1*H*-1,2,3-triazolo[4,5-*b*]pyridinium 3-oxid hexafluorophosphate

EtOAc: Ethyl acetate

DMF: Dimethylformamide

MeOH: Methanol

TFA: Trifluoroacetic acid

HPLC: High performance liquid chromatography

$Pd_2(dba)_3$: Tri(dibenzylideneacetone)dipalladium (0)

Xantphos: 4,5-Bis(diphenylphosphino)9,9-dimethylxanthene

DIAD: Diisopropyl azodicarboxylate

$Pd(dppf)Cl_2$: [1,1'-Bis(diphenylphosphino)ferrocene]dichloropalladium(II)

General Procedure 1:

Step 1: Amines (1.0 eq.), carboxylic acids (1.2 eq.) Et_3N (5.0 eq.) and HATU (1.5 eq.) were added into DMF (3-5mL). The mixture was stirred at room temperature overnight. If necessary, the mixture was diluted with EtOAc (50mL), and washed with brine (30mL×2) to remove excess DMF. Organic layer was dried over anhydrous sodium sulfate (Na_2SO_4), filtered, and concentrated under reduced pressure. The crude material was then purified by flash column chromatography (hexanes/EtOAc/MeOH).

Step 2: Products from last step were dissolved in DCM (2-3mL) and treated with TFA (2-3mL). The mixtures were stirred at room temperature until the tert-butyloxycarbonyl protecting group was cleaved tracking by UPLC-MS. The mixture was concentrated and flushed by flash column chromatography (EtOAc/MeOH/0.5%Et_3N).

Step 3: Products from last step were dissolved in DCM (2-3mL) with Et_3N (2 eq.) at 0°C. Chloroacetyl chloride (1.2 eq.), or acryloyl chloride (1.2 eq.), or cyanogen bromide (1.2eq) was added dropwisely. The mixture was then stirred at 0°C for 1 hour, and directly purified by flash chromatography (hexanes/EtOAc/MeOH) followed by preparative HPLC (MeOH or CH_3CN/H_2O with 0.0425% TFA) to afford the target products.

General Procedure 2:

Step 1: amines (1.0 eq.), epoxides (1.0 eq.) and cesium carbonate (3.0 eq.) were added into anhydrous DMF (10-15mL). The mixture was heated at 60-80 °C overnight, then cooled down to room temperature before dilution with EtOAc (~50mL). The organic layer was washed with brine (~30mL×2). Combined organic layer was dried over anhydrous sodium sulfate (Na_2SO_4), filtered, and concentrated under reduced pressure. The crude material was then purified by flash column chromatography (hexanes/EtOAc/MeOH).

Step 2: Products from last step were dissolved in DCM (2-3mL) and treated with TFA (2-3mL). The mixtures were stirred at room temperature until the tert-butyloxycarbonyl protecting group was cleaved tracking by UPLC-MS. The mixture was concentrated and flushed by flash column chromatography (EtOAc/MeOH/0.5%Et_3N).

Step 3: Products from the last step (1.0 eq.) were dissolved in DCM (2-3mL) with Et_3N (2.0-5.0 eq.) at 0°C. Chloroacetyl chloride (1.2 eq.), or acryloyl chloride (1.2 eq.), or cyanogen bromide (1.2eq) was added dropwisely. The mixture was then stirred at 0°C for 1 hour, and directly purified by flash chromatography (hexanes/EtOAc/MeOH) followed by preparative HPLC (MeOH or CH_3CN/H_2O with 0.0425% TFA) to afford the target products.

General Procedure 3:

Step 1: bromo-substituted benzo[d]thiazol-2-amine (1.0 eq.) carboxylic acids (1.2 eq.), Et3N (5.0-10.0 eq.) and HATU (1.5-2.0 eq.) were added sequentially in anhydrous DMF (5-10mL). The mixture was stirred at room temperature overnight. If necessary, the mixture was diluted with EtOAc (50mL), and washed with brine (30mL×2) to remove excess DMF. Organic layer was dried over anhydrous sodium sulfate (Na_2SO_4), filtered, and concentrated under reduced pressure. The crude material was then purified by flash column chromatography (hexanes/EtOAc/MeOH).

Step 2: The isolated products from step 1 (1.0 eq.) was dissolved in 1,4-dioxane and H_2O (3:1). Into the solution were added boronic acids or boronate ester (3.0 eq.), potassium carbonate (3.0 eq.) and $Pd(PPh_3)_4$ (0.2 eq.). The mixture was degassed by bubbling through N2 for 10min before heating up to 95°C and stirred at this temperature for 2-8 hours. The reaction was then cooled down to room temperature and diluted with EtOAc (50mL). The organic phase was washed with saturated ammonium chloride (30mL×2). Aqueous layer was then extracted with more EtOAc (50mL). Combined organic layers were washed with brine, dried over anhydrous sodium sulfate (Na_2SO_4), filtered, and concentrated under reduced pressure to afford crude material, which was then purified by flash chromatography (hexanes/EtOAc/MeOH).

Step 3: Products from last step were dissolved in DCM (2-3mL) and treated with TFA (2-3mL). The mixtures were stirred at room temperature until the tert-butyloxycarbonyl protecting group was cleaved tracking by UPLC-MS. The mixture was concentrated and flushed by flash column chromatography (EtOAc/MeOH/0.5%Et_3N).

Step 4: Products from the last step (1.0 eq.) were dissolved in DCM (2-3mL) with Et_3N (2.0-5.0 eq.) at 0°C. Cyanogen bromide (1.2eq) was then added. The mixture was then stirred at 0°C for 1 hour, and directly purified by flash chromatography (hexanes/EtOAc/MeOH) followed by preparative HPLC (MeOH or CH_3CN/H_2O with 0.0425% TFA) to afford the target products.

General Procedure 4:

Step 1: The mixture of bromobenzo[d]thiazol-2-amine (1.0 eq.), 3,5-dimethylisoxazole-4-boronic acid (1.3 eq.), sodium carbonate (2.0 eq.) were mixed in 1,4-dioxane, EtOH and H_2O (8:2:1). N_2 was bubbled through the suspension for 10 to 15min, followed by addition of tetrakis(triphenylphosphine palladium (0) (0.1 eq.) The mixture was purged with N_2 for another 5 min before stirring at 95°C overnight under N_2. Then the mixture was concentrated under reduced pressure, diluted with EtOAc, and washed with saturated NH_4Cl. Combined aqueous layer was extracted with EtOAc. Combined organic layer was washed once with brine, dried over anhydrous Na_2SO_4, filtered, and evaporated under reduced pressure to afford crude material, which was then purified by flash chromatography (hexanes/EtOAc/MeOH).

Step 2: The products isolated from last step (1.0 eq.) and (*S*)-1-Boc-pyrrolidine-3-carboxylic acid (1.2 eq.), Et_3N (5.0 eq.) and HATU (1.5 eq.) were added into DCM/DMF. The solution was stirred at room temperature overnight. The crude was then directly purified by flash chromatography (hexanes/EtOAc/MeOH) to afford desired product.

Step 3: Products from last step were dissolved in DCM (2-3mL) and treated with TFA or 4M HCl in 1,4-dioxane (2-3mL). The mixtures were stirred at room temperature until the tert-butyloxycarbonyl protecting group was cleaved tracking by UPLC-MS. The mixture was concentrated and flushed by flash column chromatography (EtOAc/MeOH/0.5%Et_3N).

Step 4: Products from the last step (1.0 eq.) were dissolved in DCM (2-3mL) with Et_3N (2.0-5.0 eq.) at 0°C. Cyanogen bromide (1.2eq) was then added. The mixture was then stirred at 0°C for 1 hour, and directly purified by flash chromatography (hexanes/EtOAc/MeOH) followed by preparative HPLC (MeOH or CH_3CN/H_2O with 0.0425% TFA) to afford the target products.

General Procedure 5:

Step 1: 5-(3,5-dimethylisoxazol-4-yl)benzo[d]thiazol-2-amine, which was synthesized in step 1 of General Procedure 4 (0.075g, 0.3mmol) was added in 3mL anhydrous MeCN. Into the solution was added $CuBr_2$ (0.065g, 0.45mmol) and t-butyl nitrite (0.046g, 0.45mmol) at 0°C. The mixture was then warmed up to room temperature then 65°C, and stirred for 4 hours. The reaction was cooled to room temperature, and diluted with water (30mL). The mixture was acidified with 12M HCl to pH=2 and extracted with EtOAc (30mL×2). The combined organic layer was washed with brine, dried over anhydrous Na_2SO_4, filtered, and concentrated under reduced pressure to afford the crude material. The material was purified by flash chromatography (hexanes/EtOAc/MeOH) to afford a mixture of desired product and chloride-substituted analogue, which did not undergo further purification and used directly in the next step. LC/MS (ESI) *m/z* 264.77; $[M+H]^+$; calcd for $C_{12}H_{10}ClN_2OS^+$: 265.02

Step 2: Products from the last step (0.14g, 0.5mmol), 1-Boc-3-oxopiperazine (0.2g, 1.0mmol), cesium carbonate (0.65g, 2.0mmol), $Pd_2(dba)_3$ (0.046g, 0.05mmol), and Xantphos (0.058g, 0.1mmol) were added into 5mL 1,4-dioxane. The mixture was degassed by bubbling in N_2 for 10-15min before heated at 95°C overnight. Then the mixture was cooled to room temperature

before diluted with EtOAc (30mL). Organic layer was washed with 20% citric acid (20mL×2). Combined aqueous layer was extracted with EtOAc (30mL). Combined organic layer was washed with brine, dried over anhydrous Na_2SO_4, filtered and concentrated under reduced pressure to afford crude material. The crude material was purified by flash chromatography (hexanes/EtOAc/MeOH) to afford desired product (0.12g,) LC/MS (ESI) *m/z* 428.87; $[M+H]^+$; calcd for $C_{21}H_{25}N_4O_4S^+$: 429.16

Step 3: Products from last step were dissolved in DCM (2-3mL) and treated with TFA (2-3mL). The mixtures were stirred at room temperature until the tert-butyloxycarbonyl protecting group was cleaved tracking by UPLC-MS. The mixture was concentrated and flushed by flash column chromatography (EtOAc/MeOH/0.5%Et_3N).

Step 4: Products from the last step (0.04g, 0.1mmol, 1.0 eq.) were dissolved in DCM (3mL) with Et_3N (0.07mL, 0.5mmol, 5.0 eq.) at 0°C. 2-chloroethane-1-sulfonyl chloride (16µL, 0.15mmol, 1.5 eq.), or acryloyl chloride (13µL, 0.15mmol, 1.5 eq.), or cyanogen bromide (3M) (50µL, 0.15mmol, 1.5 eq) was added dropwisely. The mixture was then stirred at 0°C for 1 hour, and directly purified by flash chromatography (hexanes/EtOAc/MeOH) followed by preparative HPLC (MeOH or CH_3CN/H_2O with 0.0425% TFA) to afford the target products.

General Procedure 6:

Step 1: 6-(3,5-dimethylisoxazol-4-yl)benzo[d]thiazol-2-amine, which was synthesized in step 1 of General Procedure 4 (0.25g, 1.0mmol) was added in 10mL anhydrous MeCN. Into the solution was added $CuBr_2$ (0.22g, 1.5mmol) and t-butyl nitrite (0.16g, 1.5mmol) at 0°C. The mixture was then warmed up to room temperature then 65°C, and stirred for 4 hours. The reaction was cooled to room temperature, and diluted with water (30mL). The mixture was acidified with 12M HCl to pH=2 and extracted with EtOAc (30mL×2). The combined organic layer was washed with brine, dried over anhydrous Na2SO4, filtered, and concentrated under reduced pressure to afford the crude material. The material was purified by flash chromatography (hexanes/EtOAc/MeOH) to afford 0.26g mixture of desired product and chloride-substituted analogue, which did not

undergo further purification and used directly in the next step. LC/MS (ESI) *m/z* 308.87; $[M+H]^+$; calcd for $C_{12}H_{10}BrN_2OS^+$: 308.97

Step 2: The product isolated from last step (0.08g, 0.26mmol), 3 or 4-aminophenylboronic acid (0.05g, 0.4mmol), potassium carbonate (0.07g, 0.52mmol), and Pd(dppf)Cl_2 (0.022g, 0.03mmol) were added into 1,4-dioxane/H_2O (4mL, 3:1). The mixture was degassed by bubbling in N_2 for 10-15min before heated at 95°C overnight. Then the mixture was cooled to room temperature before diluted with EtOAc (30mL). Organic layer was washed with saturated ammonium chloride. Combined aqueous layer was extracted with EtOAc (30mL). Combined organic layer was washed with brine, dried over anhydrous Na_2SO_4, filtered and concentrated under reduced pressure to afford crude material. The crude material was purified by flash chromatography (hexanes/EtOAc/MeOH) to afford desired products (4-amino: 0.084g, LC/MS (ESI) *m/z* 322.04; $[M+H]^+$; calcd for $C_{18}H_{16}N_3OS^+$: 322.10; 3-amino:0.073g LC/MS (ESI) *m/z* 322.04; $[M+H]^+$; calcd for $C_{18}H_{16}N_3OS^+$: 322.10)

Step 3a: Products from the last step (4-(6-(3,5-dimethylisoxazol-4-yl)benzo[d]thiazol-2-yl)aniline (0.042g, 0.13mmol, 1.0 eq.)) were dissolved in DCM (3mL) with Et_3N (0.056mL, 0.4mmol, 3.0 eq.) at 0°C. 2-chloroethane-1-sulfonyl chloride (22µL, 0.2mmol, 1.5 eq.), or acryloyl chloride (16µL, 0.2mmol, 1.5 eq) was added dropwisely. The mixture was then stirred at 0°C for 1 hour, and directly purified by flash chromatography (hexanes/EtOAc/MeOH) followed by preparative HPLC (MeOH or CH_3CN/H_2O with 0.0425% TFA) to afford the target products.

Step 3b: Products from the last step (3-(6-(3,5-dimethylisoxazol-4-yl)benzo[d]thiazol-2-yl)aniline (0.036g, 0.11mmol, 1.0 eq.)) were dissolved in DCM (3mL) with Et_3N (0.07mL, 0.55mmol, 5.0 eq.) at 0°C. 2-chloroethane-1-sulfonyl chloride (14µL, 0.12mmol, 1.1 eq.), or acryloyl chloride (11µL, 0.12mmol, 1.1 eq) was added dropwisely. The mixture was then stirred at 0°C for 1 hour, and directly purified by flash chromatography (hexanes/EtOAc/MeOH) followed by preparative HPLC (MeOH or CH_3CN/H_2O with 0.0425% TFA) to afford the target products.

General Procedure 7:

Step 1: bromo-substituted heterocyclic carboxylic acids (1.0 eq.) were added into 5mL anhydrous DCM under N_2. Into the mixture was added benzylamine (1.0 eq.), Et_3N (10.0 eq.) and T3P (5.0

eq.). The reaction mixture was stirred at room temperature overnight, and directly purified by flash chromatography (hexanes/EtOAc/MeOH) followed by preparative HPLC (MeOH or CH_3CN/H_2O with 0.0425% TFA) to afford the target products.

Synthesis of XL_10320_005A, 005B, 005C:

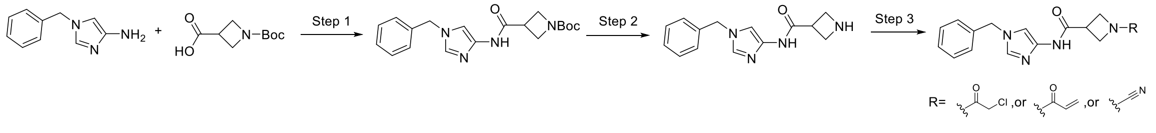

Step 1: The synthesis was preformed according to General Procedure 1 with 1-benzyl-1H-imidazol-4-amine (0.25g, 1.5mmol) and 1-(tert-butoxycarbonyl)azetidine-3-carboxylic acid (0.36g, 1.8mmol). 0.13g desired compound (tert-butyl 3-((1-benzyl-1H-imidazol-4-yl)carbamoyl)azetidine-1-carboxylate) was obtained (24%). LC/MS (ESI) *m/z* 357.07; $[M+H]^+$ calcd for $C_{19}H_{25}N_4O_3^+$: 357.19.

Step 2: The synthesis was performed according to the General Procedure 1 tert-butyl 3-((1-benzyl-1H-imidazol-4-yl)carbamoyl)azetidine-1-carboxylate (0.13g, 0.37mmol). 0.08g N-(1-benzyl-1H-imidazol-4-yl)azetidine-3-carboxamide (84%)

Step 3: The synthesis was performed according to the General Procedure 1 with N-(1-benzyl-1H-imidazol-4-yl)azetidine-3-carboxamide (0.016g, 0.06mmol) and chloroacetyl chloride (10.0 µL, 0.12mmol), or acryloyl chloride (10.0 µL, 0.12mmol), or cyanogen bromide (0.013g, 0.12mmol).

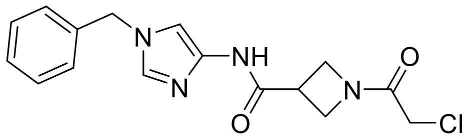

XL_10320_005A (5mg,25%) ^{1}H NMR (500 MHz, DMSO) δ 10.70 (s, 1H), 8.09 (s, 1H), 7.54 – 7.08 (m, 6H), 5.22 (s, 2H), 4.34 (t, *J* = 8.7 Hz, 1H), 4.25 (dd, *J* = 8.5, 5.9 Hz, 1H), 4.17 – 4.08 (d, *J* = 2.4 Hz , 2H), 4.03 (t, *J* = 9.3 Hz, 1H), 3.95 (dd, *J* = 9.6, 5.9 Hz, 1H). (3-H on the azetidine ring may overlap with water peak) LC/MS (ESI) *m/z* 332.87; $[M+H]^+$ calcd for $C_{16}H_{18}ClN_4O_2^+$: 333.11

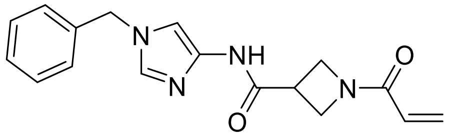

XL_10320_005B (4mg,22%) ^{1}H NMR (500 MHz, DMSO) δ 10.70 (s, 1H), 8.05 (s, 1H), 7.46 – 7.24 (m, 6H), 6.30 (dd, *J* = 17.0, 10.3 Hz, 1H), 6.10 (dd, *J* = 17.0, 2.2 Hz, 1H), 5.67 (dd, *J* = 10.3, 2.2 Hz, 1H), 5.22 (s, 2H), 4.35 (t, *J* = 8.7 Hz, 1H), 4.24 (dd, *J* = 8.4, 5.8 Hz, 1H), 4.05 (t, *J* = 9.4 Hz, 1H), 3.95 (dd, *J* = 9.8, 5.9 Hz, 1H). (3-H on the azetidine ring may overlap with water peak) LC/MS (ESI) *m/z* 310.97; [M+H]$^+$ calcd for $C_{17}H_{19}N_4O_2^+$: 311.15

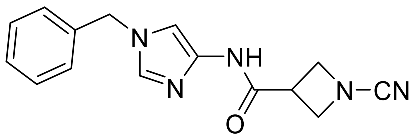

XL_10320_005C (8mg, 47%) 1H NMR (500 MHz, DMSO) δ 10.46 (s, 1H), 7.60 (s, 1H), 7.41 – 7.34 (m, 2H), 7.34 – 7.23 (m, 4H), 5.16 (s, 2H), 4.24 (t, J = 8.1 Hz, 2H), 4.18 (t, J = 6.9 Hz, 2H), 3.62 (ddd, J = 15.2, 8.6, 6.6 Hz, 1H). LC/MS (ESI) m/z 282.08; [M+H]$^+$ calcd for $C_{15}H_{16}N_5O^+$: 282.13

Synthesis of XL_10320_006A, 006B, 006C:

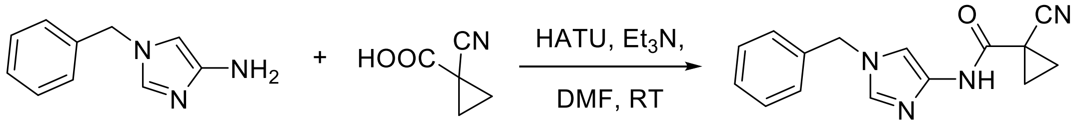

XL_10320_006A

1-benzyl-1H-imidazol-4-amine (0.06g, 0.3mmol), 1-cyanocyclopropane-1-carboxylic acid (0.05g, 0.5mmol) Et_3N (0.48mL, 3.0mmol), and HATU (0.24g, 0.64mmol) were added sequentially to 3mL anhydrous DMF. The reaction mixture was stirred overnight at room temperature, and purified by flash chromatography (hexanes/EtOAc/MeOH) and preparative HPLC (MeOH/H_2O w/ 0.0425% TFA) to afford XL_10320_006A (53mg, 67%) ^{1}H NMR (500 MHz, DMSO) δ 10.59 (s, 1H), 8.17 (s, 1H), 7.39 (m, 2H), 7.36 – 7.30 (m, 4H), 5.25 (s, 2H), 1.73 – 1.59 (m, 4H). LC/MS (ESI) *m/z* 266.97; [M+H]$^+$ calcd for $C_{15}H_{15}N_4O^+$: 267.12

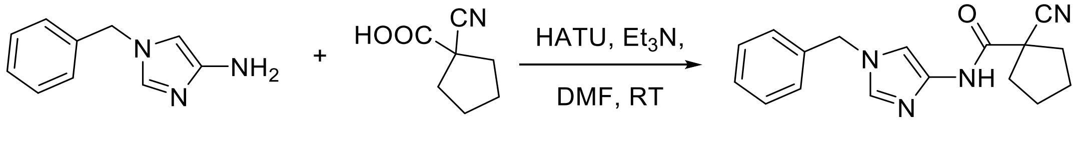

XL_10320_006B

1-benzyl-1H-imidazol-4-amine (0.06g, 0.3mmol), 1-cyanocyclopentane-1-carboxylic acid (0.07g, 0.5mmol) Et_3N (0.48mL, 3.0mmol), and HATU (0.24g, 0.64mmol) were added sequentially to 3mL

anhydrous DMF. The reaction mixture was stirred overnight at room temperature, and purified by flash chromatography (hexanes/EtOAc/MeOH) and preparative HPLC (MeOH/H_2O w/ 0.0425% TFA) to afford XL_10320_006B (61mg, 69%) ^{1}H NMR (500 MHz, DMSO) δ 10.79 (s, 1H), 7.65 (d, *J* = 1.5 Hz, 1H), 7.38 (dd, *J* = 9.9, 4.5 Hz, 2H), 7.31 (ddd, *J* = 13.2, 6.7, 4.1 Hz, 3H), 7.23 (d, *J* = 1.5 Hz, 1H), 5.16 (s, 2H), 2.33 – 2.13 (m, 4H), 1.81 – 1.60 (m, 4H). LC/MS (ESI) *m/z* 295.07 $[M+H]^+$; calcd for $C_{17}H_{19}N_4O^+$: 295.15

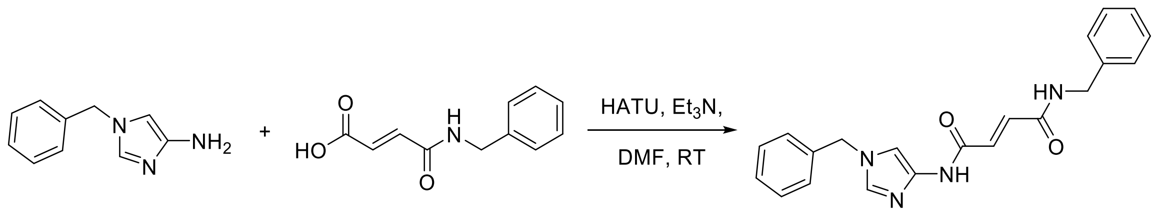

XL_10320_006C

1-benzyl-1H-imidazol-4-amine (0.06g, 0.3mmol), (E)-4-(benzylamino)-4-oxobut-2-enoic acid (0.10g, 0.5mmol) Et_3N (0.48mL, 3.0mmol), and HATU (0.24g, 0.64mmol) were added sequentially to 3mL anhydrous DMF. The reaction mixture was stirred overnight at room temperature, and purified by flash chromatography (hexanes/EtOAc/MeOH) and preparative HPLC (MeOH/H_2O w/ 0.0425% TFA) to afford XL_10320_006C (6mg, 6%) ^{1}H NMR (500 MHz, DMSO) δ 10.88 (s, 1H), 8.93 (t, *J* = 6.0 Hz, 1H), 7.65 (d, *J* = 1.4 Hz, 1H), 7.45 – 7.18 (m, 11H), 7.08 (d, *J* = 15.1 Hz, 1H), 6.95 (d, *J* = 15.1 Hz, 1H), 5.18 (s, 2H), 4.38 (d, *J* = 6.0 Hz, 2H). LC/MS (ESI) *m/z* 360.97; $[M+H]^+$ calcd for $C_{21}H_{21}N_4O_2^+$: 361.17

<u>Synthesis of XL_10320_007A, 007B, 007C:</u>

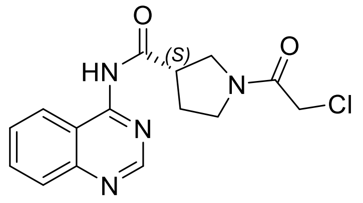

XL_10320_007A (10mg, 11%) 1H NMR (500 MHz, DMSO, mixture of rotamers) δ 8.94 (d, J = 4.8 Hz, 1H), 8.22 (d, J = 8.4 Hz, 1H), 8.01 – 7.89 (m, 2H), 7.73 – 7.64 (m, 1H), 4.34 (d, J = 2.2 Hz, 1H), 3.86 (dd, J = 10.0, 7.7 Hz, 0.6H), 3.79 – 3.63 (m, 2H), 3.63 – 3.50 (m, 2H), 3.40 (dt, J = 11.8, 7.6 Hz,

0.7H), 2.35 – 2.28 (m, 0.6H), 2.28 – 2.18 (m, 1H), 2.17 – 2.06 (m, 0.7H). LC/MS (ESI) m/z 318.97; [M+H]+ calcd for $C_{15}H_{16}ClN_4O_2^+$: 319.10

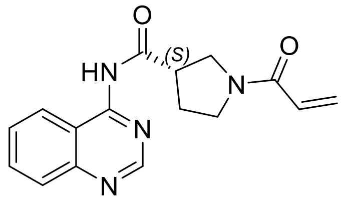

XL_10320_007B (3mg, 3%) 1H NMR (500 MHz, DMSO, mixture of rotamers) δ 8.94 (d, J = 6.0 Hz, 1H), 8.22 (d, J = 8.3 Hz, 1H), 7.94 (m, 2H), 7.69 (m, 1H), 6.62 (ddd, J = 16.8, 12.0, 10.3 Hz, 1H), 6.22 – 6.07 (m, 1H), 5.68 (dt, J = 10.3, 2.4 Hz, 1H), 3.91 (dd, J = 10.3, 7.8 Hz, 0.6H), 3.86 – 3.77 (m, 0.6H), 3.77 – 3.52 (m, 3H), 3.47 – 3.39 (m, 0.6H), 2.26 (m, 1.6H), 2.12 (m, 0.8H) LC/MS (ESI) m/z 296.87; [M+H]+ calcd for $C_{16}H_{17}N_4O_2^+$: 297.13

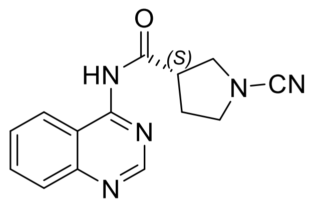

XL_10320_007C (4mg, 5%) 1H NMR (500 MHz, DMSO, mixture of rotamers) δ 8.91 (s, 1H), 8.23 (d, J = 8.3 Hz, 1H), 8.00 – 7.88 (m, 2H), 7.72 – 7.62 (m, 1H), 3.68 (dd, J = 11.2, 10.0 Hz, 1H), 3.65 – 3.59 (m, 2H), 3.53 – 3.40 (m, 2H), 2.30 – 2.12 (m, 2H). LC/MS (ESI) m/z 267.97; [M+H]+ calcd for $C_{14}H_{14}N_5O^+$: 268.12

<u>Synthesis of XL_10320_009A, 009B, 009C</u>:

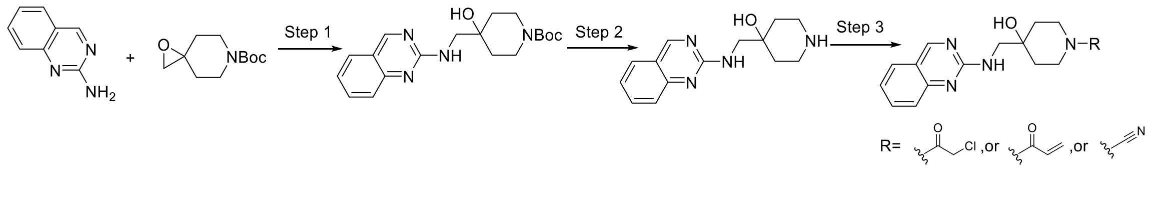

Step 1: The synthesis was performed according to General Procedure 2 with quinazolin-2-amine (0.1g, 0.7mmol) and tert-butyl 1-oxa-6-azaspiro[2.5]octane-6-carboxylate (0.15g, 0.7mmol) with exception of using NaH (60% in mineral oil) (0.03g, 0.75mmol) as base instead of cesium carbonate. 0.16g desired product (tert-butyl 4-hydroxy-4-((quinazolin-2-

ylamino)methyl)piperidine-1-carboxylate) was obtained (64%). LC/MS (ESI) *m/z* 358.97; $[M+H]^+$ calcd for $C_{19}H_{27}N_4O_3^+$: 359.21

Step 2: The synthesis was performed according to the General Procedure 2 tert-butyl 4-hydroxy-4-((quinazolin-2-ylamino)methyl)piperidine-1-carboxylate (0.16g, 0.45mmol) except for using 4N HCl in 1,4-dioxane instead of TFA/DCM. 0.12g 4-((quinazolin-2-ylamino)methyl)piperidin-4-ol was obtained (quant.)

Step 3: The synthesis was performed according to the General Procedure 2 with 4-((quinazolin-2-ylamino)methyl)piperidin-4-ol (0.04g, 0.16mmol), Et_3N (0.13mL, 0.9mmol) and chloroacetyl chloride (14.0µL, 0.18mmol), or acryloyl chloride (15.0µL, 0.18mmol), or cyanogen bromide (0.019g, 0.18mmol).

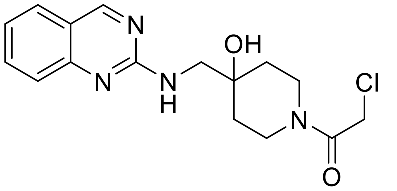

XL_10320_009A (5mg, 9%) ^{1}H NMR (500 MHz, DMSO) δ 9.38 (m, 1H), 8.53 (d, *J* = 234.4 Hz, 1H), 7.88 (d, *J* = 51.8 Hz, 2H), 7.60 (d, *J* = 8.4 Hz, 1H), 7.39 (s, 1H), 4.44 – 4.27 (m, 2H), 4.06 (d, *J* = 13.0 Hz, 1H), 3.63 (d, *J* = 13.5 Hz, 2H), 3.35 (dd, *J* = 18.4, 9.4 Hz, 1H), 3.02 (t, *J* = 11.5 Hz, 1H), 1.55 (dd, *J* = 45.8, 17.5 Hz, 4H). LC/MS (ESI) *m/z* 334.87; $[M+H]^+$ calcd for $C_{16}H_{20}ClN_4O_2^+$: 335.13

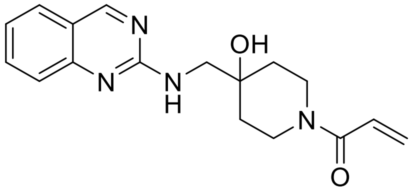

XL_10320_009B (2mg, 1%) 1H NMR (500 MHz, DMSO) δ 9.12 (s, 1H), 7.80 (m, 1H), 7.75 – 7.58 (m, 1H), 7.44 (m, 1H), 7.31 – 7.09 (m, 2H), 6.92 – 6.69 (m, 1H), 6.06 (dd, J = 16.6, 6.3 Hz, 1H), 5.77 – 5.50 (m, 1H), 5.10 (br, 1H), 4.12 (m, 1H), 3.82 (d, J = 11.4 Hz, 1H), 3.45 (m, 2H), 3.04 (m, 1H), 1.53 (m, 4H). LC/MS (ESI) m/z 312.87; $[M+H]^+$ calcd for $C_{15}H_{18}N_5O^+$: 313.17

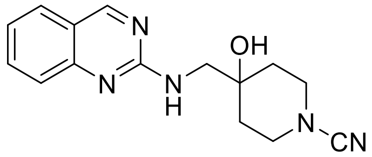

XL_10320_009C (10mg, 8%). 1H NMR (500 MHz, DMSO) δ 9.23 (s, 1H), 7.89 (d, J = 7.9 Hz, 1H), 7.77 (dd, J = 20.4, 12.9 Hz, 1H), 7.53 (t, J = 10.9 Hz, 1H), 7.33 (dd, J = 17.2, 9.7 Hz, 1H), 3.54 (s, 2H), 3.34 – 3.24 (m, 2H), 3.21 (dt, J = 12.7, 4.1 Hz, 2H), 1.77 – 1.64 (m, 2H), 1.60 (d, J = 13.2 Hz, 2H). LC/MS (ESI) m/z 283.87; $[M+H]^+$ calcd for $C_{15}H_{18}N_5O^+$: 284.15

Synthesis of Xl_10320_027A, 027B, 027C

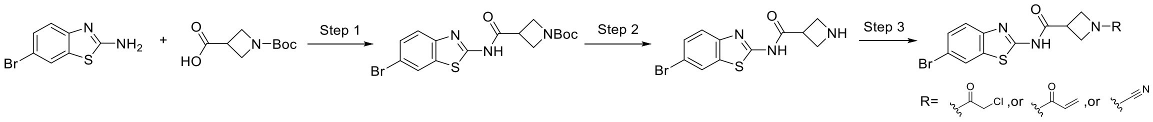

Step 1: The synthesis was preformed according to General Procedure 1 with 6-bromobenzo[d]thiazol-2-amine (0.39g, 1.7mmol) and 1-(tert-butoxycarbonyl)azetidine-3-carboxylic acid (0.42g, 2.1mmol). 0.21g desired compound (tert-butyl 3-((6-bromobenzo[d]thiazol-2-yl)carbamoyl)azetidine-1-carboxylate) was obtained (30%). LC/MS (ESI) *m/z* 356.07 (M+H−*t*-butyl); $[M+H]^+$ calcd for $C_{16}H_{19}BrN_3O_3S^+$: 412.03

Step 2: The synthesis was performed according to the General Procedure 1 with tert-butyl 3-((6-bromobenzo[d]thiazol-2-yl)carbamoyl)azetidine-1-carboxylate (0.21g, 0.5mmol). 0.18g N-(6-bromobenzo[d]thiazol-2-yl)azetidine-3-carboxamide was obtained (quant.)

Step 3: The synthesis was performed according to the General Procedure 1 with N-(4-phenylthiazol-2-yl)azetidine-3-carboxamide (0.06g, 0.17mmol) and chloroacetyl chloride (0.017mL, 0.2mmol), or acryloyl chloride (0.017mL, 0.2mmol), or cyanogen bromide (0.02g, 0.2mmol).

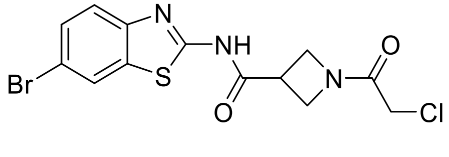

XL_10320_027A (43mg, 65%) ^{1}H NMR (500 MHz, DMSO) δ 12.62 (s, 1H), 8.28 (d, *J* = 1.9 Hz, 1H), 7.69 (d, *J* = 8.6 Hz, 1H), 7.58 (dd, *J* = 8.6, 2.0 Hz, 1H), 4.47 – 4.33 (m, 2H), 4.17 (s, 2H), 4.15 – 4.03

(m, 2H), 3.77 (tt, *J* = 8.9, 5.9 Hz, 1H), 3.18 (d, *J* = 4.7 Hz, 1H). LC/MS (ESI) *m/z* 387.77; $[M+H]^+$ calcd for $C_{13}H_{12}BrClN_3O_2S^+$: 387.95

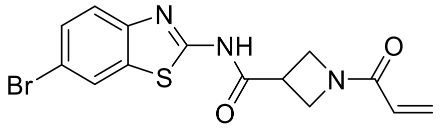

XL_10320_027B (31mg, 50%) ^1H NMR (500 MHz, DMSO) δ 12.62 (s, 1H), 8.28 (d, *J* = 2.0 Hz, 1H), 7.69 (d, *J* = 8.6 Hz, 1H), 7.58 (dd, *J* = 8.6, 2.0 Hz, 1H), 6.33 (dd, *J* = 17.0, 10.3 Hz, 1H), 6.12 (dd, *J* = 17.0, 2.2 Hz, 1H), 5.69 (dd, *J* = 10.3, 2.2 Hz, 1H), 4.48 – 4.33 (m, 2H), 4.17 – 4.03 (m, 2H), 3.76 (tt, *J* = 8.9, 5.8 Hz, 1H). LC/MS (ESI) *m/z* 365.77; $[M+H]^+$ calcd for $C_{14}H_{13}BrN_3O_2S^+$: 365.99

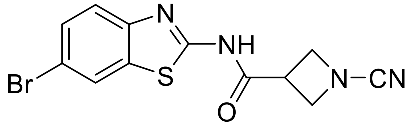

XL_10320_027C (36mg, 63%) ^1H NMR (500 MHz, DMSO) δ 12.56 (s, 1H), 8.27 (t, *J* = 4.2 Hz, 1H), 7.68 (d, *J* = 8.6 Hz, 1H), 7.58 (dd, *J* = 8.6, 2.1 Hz, 1H), 4.34 (dt, *J* = 14.0, 7.8 Hz, 4H), 3.82 (tt, *J* = 8.9, 6.3 Hz, 1H). LC/MS (ESI) *m/z* 336.77; $[M+H]^+$; calcd for $C_{12}H_{10}BrN_4OS^+$: 336.98

Synthesis of XL_10320_028A, 028B, 028C:

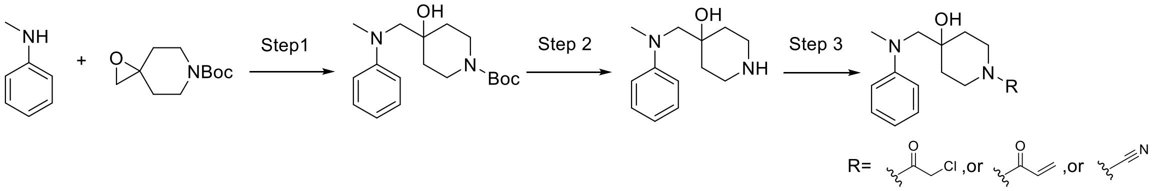

Step 1: The synthesis was performed according to General Procedure 2 with *N*-methylaniline (0.086mL, 0.8mmol) and tert-butyl 1-oxa-6-azaspiro[2.5]octane-6-carboxylate (0.17g, 0.8mmol). LiHMDS (1M) (0.8mL, 0.8mmol) was used instead of cesium carbonate. LiHMDS was dropwisely added to the solution of *N*-Methylaniline in 3mL anhydrous THF at 0°C. The mixture was stirred for 0.5h at 0°C before introducing solution of tert-butyl 1-oxa-6-azaspiro[2.5]octane-6-carboxylate in 2mL THF. The reaction was stirred at room temperature overnight before the general work-up procedure was followed. 0.25g desired product (tert-butyl 4-hydroxy-4-((methyl(phenyl)amino)methyl)piperidine-1-carboxylate) was obtained (96%). LC/MS (ESI) *m/z* 320.97; $[M+H]^+$; calcd for $C_{18}H_{29}N_2O_3^+$: 321.22

Step 2: The synthesis was performed according to the General Procedure 2 tert-butyl 4-hydroxy-4-((methyl(phenyl)amino)methyl)piperidine-1-carboxylate (0.25g, 0.78mmol) except for using 4N HCl in 1,4-dioxane instead of TFA/DCM. 0.15g 4-((methyl(phenyl)amino)methyl)piperidin-4-ol was obtained (quant.)

Step 3: The synthesis was performed according to the General Procedure 2 with 4-((methyl(phenyl)amino)methyl)piperidin-4-ol (0.05g, 0.2mmol), Et_3N (0.14mL, 1.0mmol) and chloroacetyl chloride (19.0µL, 0.24mmol), or acryloyl chloride (20.0µL, 0.24mmol), or cyanogen bromide (0.025g, 0.24mmol).

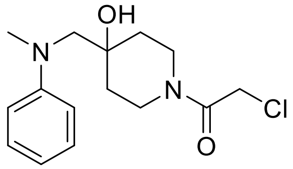

XL_10320_028A (33mg, 55%) ^{1}H NMR (500 MHz, DMSO) δ 7.13 (dd, *J* = 8.7, 7.3 Hz, 2H), 6.78 (d, *J* = 8.2 Hz, 2H), 6.58 (t, *J* = 7.2 Hz, 1H), 4.61 (s, 1H), 4.35 (s, 2H), 4.16 (t, *J* = 15.8 Hz, 1H), 3.63 (d, *J* = 13.3 Hz, 1H), 3.27 (m, 2H), 2.96 (s, 3H), 2.92 – 2.83 (m, 1H), 1.58 (m, 3H), 1.43 (td, *J* = 12.9, 4.6 Hz, 1H). LC/MS (ESI) *m/z* 296.97; $[M+H]^+$ calcd for $C_{15}H_{22}ClN_2O_2^+$: 297.14

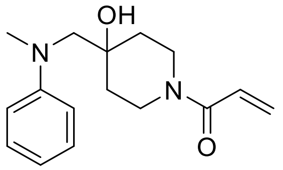

XL_10320_028B (24mg, 44%) ^{1}H NMR (500 MHz, DMSO) δ 7.12 (t, *J* = 7.9 Hz, 2H), 6.80 (m, 3H), 6.57 (t, *J* = 7.2 Hz, 1H), 6.07 (dd, *J* = 16.7, 2.4 Hz, 1H), 5.63 (dd, *J* = 10.4, 2.4 Hz, 1H), 4.23 (d, *J* = 11.0 Hz, 1H), 3.85 (d, *J* = 13.5 Hz, 1H), 3.30 (m, 3H), 2.94 (s, 3H), 2.93 – 2.83 (m, 1H), 1.46 (m, 4H). LC/MS (ESI) *m/z* 274.97; $[M+H]^+$ calcd for $C_{16}H_{23}N_2O_2^+$: 275.18

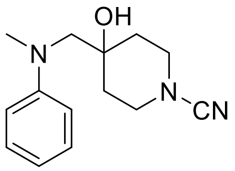

XL_10320_028C (32mg, 65%) ^{1}H NMR (500 MHz, DMSO) δ 7.14 (t, *J* = 8.0 Hz, 2H), 6.80 (d, *J* = 8.1 Hz, 2H), 6.64 – 6.48 (m, 1H), 3.68 (d, *J* = 12.7 Hz, 1H), 3.30 (s, 1H), 3.27 (s, 1H), 3.23 (dd, *J* = 12.3, 2.7 Hz, 1H), 3.17 (dd, *J* = 12.4, 2.8 Hz, 1H), 2.99 – 2.91 (m, 4H), 1.64 (td, *J* = 13.0, 5.1 Hz, 1H), 1.57 – 1.45 (m, 1H), 1.45 – 1.32 (m, 2H). LC/MS (ESI) *m/z* 245.88; [M+H]$^+$ calcd for $C_{14}H_{20}N_3O^+$: 246.16

Synthesis of 029A, 029B, 029C:

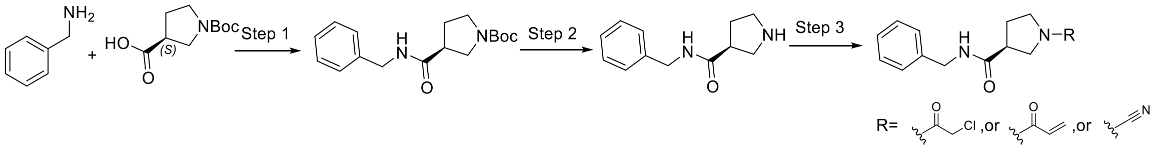

Step 1: The synthesis was preformed according to General Procedure 1 with benzylamine (0.18mL, 1.64mmol) and (*S*)-1-(tert-butoxycarbonyl)pyrrolidine-3-carboxylic acid (0.43g, 1.9mmol). 0.38g desired compound (tert-butyl (*S*)-3-(benzylcarbamoyl)pyrrolidine-1-carboxylate) was obtained (76%). LC/MS (ESI) *m/z* 249.17 (M+H−*t*-butyl); [M+H]$^+$; calcd for $C_{17}H_{25}N_2O_3^+$: 305.19

Step 2: The synthesis was performed according to the General Procedure 1 tert-butyl (*S*)-3-(benzylcarbamoyl)pyrrolidine-1-carboxylate (0.38g, 1.2mmol). 0.30g (*S*)-N-benzylpyrrolidine-3-carboxamide (quant.)

Step 3: The synthesis was performed according to the General Procedure 1 with (S)-N-benzylpyrrolidine-3-carboxamide (0.1g, 0.4mmol) and chloroacetyl chloride (40.0 µL, 0.5mmol), or acryloyl chloride (40.0 µL, 0.5mmol), or cyanogen bromide (0.53g, 0.5mmol).

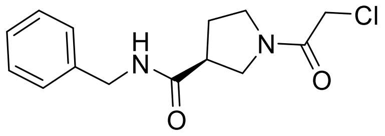

XL_10320_029A (45mg, 40%) ^{1}H NMR (500 MHz, DMSO) δ 8.55 (dt, *J* = 11.8, 5.8 Hz, 1H), 7.38 – 7.28 (m, 2H), 7.25 (m, Hz, 3H), 4.32 – 4.28 (m, 4H), 3.71 (dd, *J* = 10.1, 7.9 Hz, 0.5H), 3.67 – 3.53 (m, 1.5H), 3.54 – 3.40 (m, 1H), 3.42 – 3.36 (m, 0.5H), 3.32 – 3.25 (m, 0.5H), 3.15 – 3.03 (m, 0.5H), 2.99 (p, *J* = 7.7 Hz, 0.5H), 2.22 – 1.85 (m, 2H). (mixture of rotamers) LC/MS (ESI) *m/z* 280.87; [M+H]$^+$ calcd for $C_{14}H_{18}ClN_2O_2^+$: 281.11

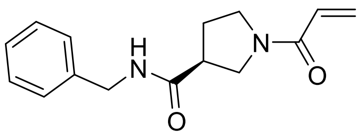

XL_10320_029B (61mg, 59%) ^{1}H NMR (500 MHz, DMSO) δ 8.54 (d, *J* = 5.2 Hz, 1H), 7.37 – 7.30 (m, 2H), 7.25 (d, *J* = 6.8 Hz, 3H), 6.57 (ddd, *J* = 16.7, 10.3, 6.4 Hz, 1H), 6.13 (dt, *J* = 16.8, 2.0 Hz, 1H), 5.78 – 5.52 (m, 1H), 4.29 (d, *J* = 7.3 Hz, 2H), 3.72 – 3.65 (m, 0.5H), 3.65 – 3.57 (m, 1H), 3.53 (m, 1H), 3.43 (dd, *J* = 12.1, 7.2 Hz, 0.5H), 3.38 – 3.30 (m, 0.5H), 3.09 (dt, *J* = 15.3, 7.6 Hz, 0.5H), 2.99 (dt, *J* = 15.1, 7.6 Hz, 0.5H), 2.17 – 2.02 (m, 1.5H), 1.94 (m, 0.5H). (mixture of rotamers) LC/MS (ESI) *m/z* 258.87; $[M+H]^+$ calcd for $C_{15}H_{19}N_2O_2^+$: 259.14

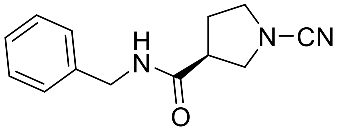

XL_10320_029C (88mg, 96%) ^{1}H NMR (500 MHz, DMSO) δ 8.52 (dt, *J* = 32.7, 5.7 Hz, 1H), 7.38 – 7.29 (m, 2H), 7.29 – 7.21 (m, 3H), 4.39 – 4.19 (m, 2H), 3.55 (dd, *J* = 9.2, 7.9 Hz, 2H), 3.47 – 3.39 (m, 2H), 3.39 – 3.32 (m, 1H), 3.10 – 2.98 (m, 1H), 2.17 – 2.02 (m, 1H), 2.03 – 1.92 (m, 1H). LC/MS (ESI) *m/z* 230.08; $[M+H]^+$ calcd for $C_{13}H_{16}N_3O^+$: 230.13

Synthesis of XL_10320_061A:

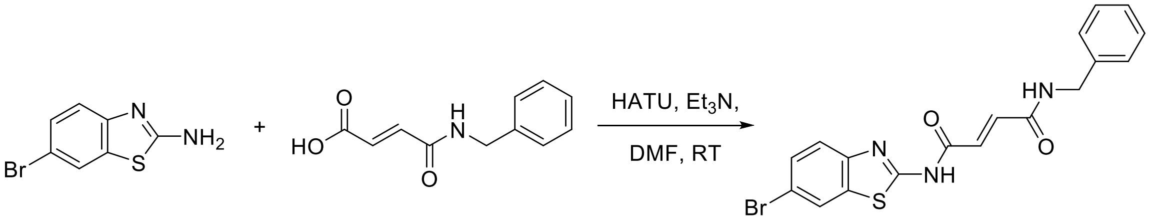

XL_10320_061

6-bromobenzo[d]thiazol-2-amine (0.046g, 0.2mmol), (E)-4-(benzylamino)-4-oxobut-2-enoic acid (0.05g, 0.24mmol) Et_3N (0.28mL, 2.0mmol), and HATU (0.15g, 0.4mmol) were added sequentially to 3mL anhydrous DMF. The reaction mixture was stirred overnight at room temperature, and purified by flash chromatography (hexanes/EtOAc/MeOH) and preparative HPLC (MeOH/H_2O w/ 0.0425% TFA) to afford XL_10320_061A (14mg, 17%): ^{1}H NMR (500 MHz, DMSO) δ 12.91 (s, 1H),

9.10 (t, *J* = 5.9 Hz, 1H), 8.30 (d, *J* = 2.0 Hz, 1H), 7.73 (d, *J* = 8.6 Hz, 1H), 7.60 (dd, *J* = 8.6, 2.1 Hz, 1H), 7.35 (dd, *J* = 10.1, 4.6 Hz, 1H), 7.28 (dd, *J* = 16.4, 7.7 Hz, 2H), 7.25 – 7.15 (m, 2H), 6.54 (s, 2H), 4.42 (d, *J* = 5.9 Hz, 2H). LC/MS (ESI) *m/z* 415.77; $[M+H]^+$ calcd for $C_{18}H_{15}BrN_3O_2S^+$: 416.01

<u>Synthesis of XL_10320_054A, 054B:</u>

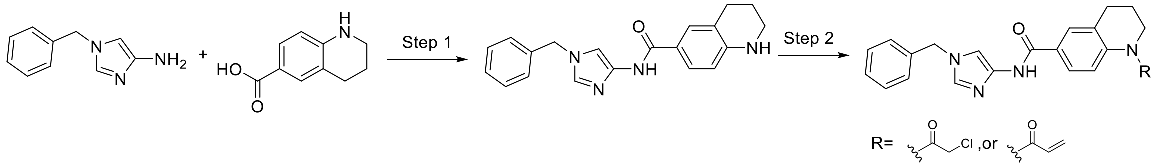

Step 1: The synthesis was preformed according to General Procedure 1 with 1-benzyl-1H-imidazol-4-amine (0.25g, 1.5mmol) and 1,2,3,4-tetrahydroquinoline-6-carboxylic acid (0.38g, 1.8mmol). 0.17g desired compound (*N*-(1-benzyl-1H-imidazol-4-yl)-1,2,3,4-tetrahydroquinoline-6-carboxamide) was obtained (34%). LC/MS (ESI) *m/z* 332.87; $[M+H]^+$ calcd for $C_{20}H_{21}N_4O^+$: 333.17

Step 2: The synthesis was performed according to the General Procedure 1 with N-(1-benzyl-1H-imidazol-4-yl)-1,2,3,4-tetrahydroquinoline-6-carboxamide (0.087g, 0.26mmol) and chloroacetyl chloride (25.0 µL, 0.32mmol), or acryloyl chloride (25.0 µL, 0.32mmol).

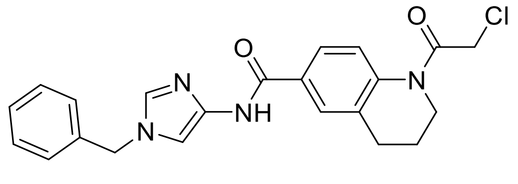

XL_10320_054A (42mg, 39%) ^{1}H NMR (500 MHz, DMSO) δ 10.98 (s, 1H), 8.33 (s, 1H), 7.83 (s, 1H), 7.80 (d, *J* = 8.5 Hz, 1H), 7.75 (s, 1H), 7.51 (s, 1H), 7.45 – 7.33 (m, 5H), 5.30 (s, 2H), 4.62 (s, 2H), 3.74 (dd, *J* = 14.8, 8.6 Hz, 2H), 2.78 (dd, *J* = 22.6, 16.0 Hz, 2H), 2.01 – 1.87 (m, 2H). LC/MS (ESI) *m/z* 409.37; $[M+H]^+$ calcd for $C_{22}H_{22}ClN_4O_2^+$: 409.14

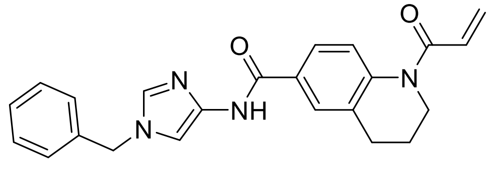

XL_10320_054B (41mg, 41%) ^{1}H NMR (500 MHz, DMSO) δ 10.95 (s, 1H), 8.30 (s, 1H), 7.85 (d, *J* = 1.7 Hz, 1H), 7.80 (dd, *J* = 8.5, 2.0 Hz, 1H), 7.50 (d, *J* = 1.3 Hz, 1H), 7.46 – 7.26 (m, 6H), 6.59 (dd, *J*

= 16.7, 10.3 Hz, 1H), 6.26 (dd, *J* = 16.8, 2.1 Hz, 1H), 5.77 (dd, *J* = 10.3, 2.1 Hz, 1H), 5.30 (s, 2H), 3.77 (t, *J* = 6.4 Hz, 2H), 2.79 (t, *J* = 6.5 Hz, 2H), 1.91 (p, *J* = 6.5 Hz, 2H). LC/MS (ESI) *m/z* 387.37; $[M+H]^+$ calcd for $C_{23}H_{23}N_4O_2^+$: 387.18

Synthesis of XL_10320_062A, 062B, 062C:

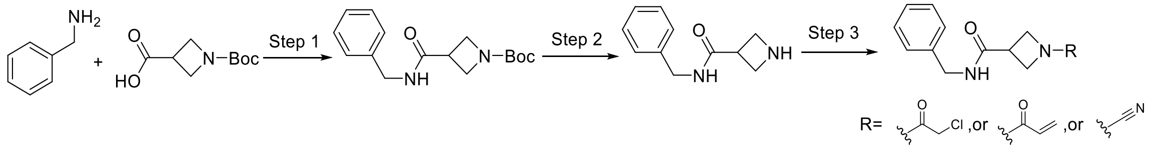

Step 1: The synthesis was preformed according to General Procedure 1 with benzylamine (0.22mL, 2.0mmol) and 1-(tert-butoxycarbonyl)azetidine-3-carboxylic acid (0.6g, 3.0mmol). 0.37g desired compound (tert-butyl 3-(benzylcarbamoyl)azetidine-1-carboxylate) was obtained (64%). LC/MS (ESI) *m/z* 291.17; $[M+H]^+$ calcd for $C_{16}H_{23}N_2O_3^+$: 291.17

Step 2: The synthesis was performed according to the General Procedure 1 tert-butyl 3-(benzylcarbamoyl)azetidine-1-carboxylate (0.37g, 1.2mmol). 0.23g *N*-benzylazetidine-3-carboxamide (quant.)

Step 3: The synthesis was performed according to the General Procedure 1 *N*-benzylazetidine-3-carboxamide (0.08g, 0.4mmol) and chloroacetyl chloride (40.0 µL, 0.5mmol), or acryloyl chloride (40.0 µL, 0.5mmol), or cyanogen bromide (0.53g, 0.5mmol).

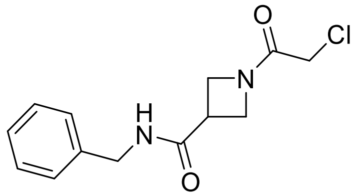

XL_10320_062A (31mg, 29%) ^{1}H NMR (500 MHz, DMSO) δ 8.54 (t, *J* = 5.4 Hz, 1H), 7.38 – 7.29 (m, 2H), 7.29 – 7.20 (m, 3H), 4.32 (dd, *J* = 10.5, 7.3 Hz, 3H), 4.27 – 4.21 (m, 1H), 4.18 – 4.09 (m, 2H), 4.03 (t, *J* = 9.2 Hz, 1H), 3.93 (dd, *J* = 9.4, 6.0 Hz, 1H), 3.45 – 3.38 (m, 1H). ^{13}C NMR (126 MHz, DMSO) δ 171.55, 166.08, 139.60, 128.81, 127.75, 127.35, 53.14, 51.22, 42.76, 40.36, 32.66. LC/MS (ESI) *m/z* 267.17; $[M+H]^+$ calcd for $C_{13}H_{16}ClN_2O_2^+$: 267.09

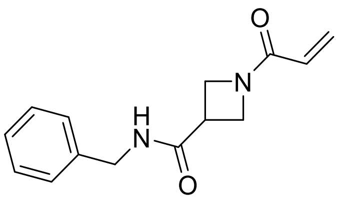

XL_10320_062B (36mg, 37%) ^{1}H NMR (500 MHz, DMSO) δ 8.55 (t, *J* = 5.7 Hz, 1H), 7.38 – 7.29 (m, 2H), 7.29 – 7.20 (m, 3H), 6.31 (dd, *J* = 17.0, 10.3 Hz, 1H), 6.10 (dd, *J* = 17.0, 2.2 Hz, 1H), 5.67 (dd, *J* = 10.3, 2.2 Hz, 1H), 4.39 – 4.28 (m, 3H), 4.23 (dd, *J* = 8.3, 5.9 Hz, 1H), 4.04 (t, *J* = 9.3 Hz, 1H), 3.93 (dd, *J* = 9.7, 5.9 Hz, 1H). (one proton overlaps with HDO peak) LC/MS (ESI) *m/z* 245.28; $[M+H]^+$ calcd for $C_{14}H_{17}N_2O_2^+$: 245.13

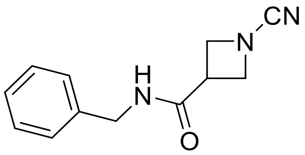

XL_10320_062C (51mg, 56%) ^{1}H NMR (500 MHz, DMSO) δ 8.50 (t, *J* = 5.4 Hz, 1H), 7.37 – 7.29 (m, 2H), 7.29 – 7.21 (m, 3H), 4.30 (d, *J* = 5.9 Hz, 2H), 4.26 (dd, *J* = 8.8, 7.5 Hz, 2H), 4.21 – 4.16 (m, 2H), 3.49 (tt, *J* = 8.9, 6.4 Hz, 1H). LC/MS (ESI) *m/z* 216.18; $[M+H]^+$ calcd for $C_{12}H_{14}N_3O^+$: 216.11

Synthesis of XL_10320_064A, 064B, 064C:

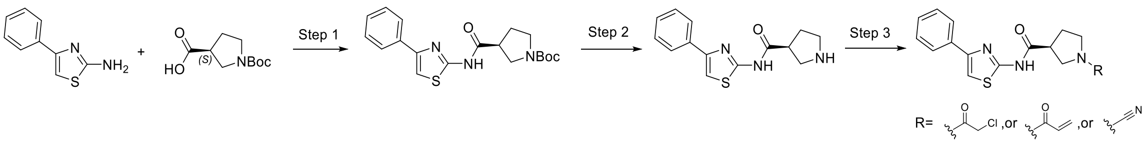

Step 1: The synthesis was preformed according to General Procedure 1 with 4-phenylthiazol-2-amine (0.26g, 1.5mmol) and (S)-1-(tert-butoxycarbonyl)pyrrolidine-3-carboxylic acid (0.38g, 1.8mmol). 0.6g desired compound (tert-butyl (*S*)-3-((4-phenylthiazol-2-yl)carbamoyl)pyrrolidine-1-carboxylate) was obtained (quant.). LC/MS (ESI) *m/z* 274.17 (M+H−Boc); $[M+H]^+$ calcd for $C_{19}H_{24}N_3O_3S^+$: 374.15

Step 2: The synthesis was performed according to the General Procedure 1 with tert-butyl (*S*)-3-((4-phenylthiazol-2-yl)carbamoyl)pyrrolidine-1-carboxylate (0.6g, 1.5mmol). 0.40g (*S*)-N-(4-phenylthiazol-2-yl)pyrrolidine-3-carboxamide (quant.)

Step 3: The synthesis was performed according to the General Procedure 1 with (*S*)-N-(4-phenylthiazol-2-yl)pyrrolidine-3-carboxamide (0.13g, 0.5mmol) and chloroacetyl chloride (0.08mL, 1.0mmol), or acryloyl chloride (0.08mL, 1.0mmol), or cyanogen bromide (0.11g, 1.0mmol).

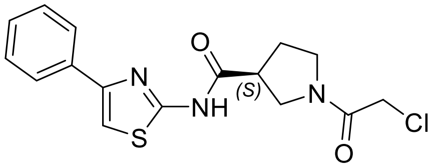

XL_10320_064A (12mg, 7%) ^{1}H NMR (500 MHz, DMSO) δ 12.48 (d, *J* = 7.6 Hz, 1H), 7.90 (d, *J* = 7.4 Hz, 2H), 7.65 (d, *J* = 2.5 Hz, 1H), 7.44 (t, *J* = 7.7 Hz, 2H), 7.33 (t, *J* = 7.3 Hz, 1H), 4.33 (d, *J* = 2.2 Hz, 2H), 3.80 (dd, *J* = 10.4, 7.8 Hz, 1H), 3.74 – 3.60 (m, 2H), 3.53 (ddd, *J* = 17.8, 11.3, 6.5 Hz, 3H), 2.33 – 2.00 (m, 3H).(Conformational isomers were observed. Protons overlaps with HDO peak) LC/MS (ESI) *m/z* 350.17; $[M+H]^+$ calcd for $C_{16}H_{17}ClN_3O_2S^+$: 350.07

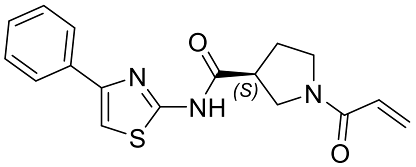

XL_10320_064B (8mg, 5%) ^{1}H NMR (500 MHz, DMSO) δ 12.47 (s, 1H), 7.95 – 7.85 (m, 2H), 7.65 (d, *J* = 1.8 Hz, 1H), 7.44 (t, *J* = 7.7 Hz, 2H), 7.33 (t, *J* = 7.4 Hz, 1H), 6.60 (ddd, *J* = 16.8, 10.3, 3.5 Hz, 1H), 6.15 (dd, *J* = 16.8, 2.4 Hz, 1H), 5.69 (dd, *J* = 10.3, 2.4 Hz, 1H), 3.87 (dd, *J* = 10.3, 7.8 Hz, 1H), 3.72 (ddd, *J* = 12.2, 9.2, 5.4 Hz, 2H), 3.41 (dd, *J* = 13.4, 6.4 Hz, 2H), 3.32 (dt, *J* = 14.0, 7.1 Hz, 1H), 2.33 – 1.99 (m, 3H). (Conformational isomvers were observed) LC/MS (ESI) *m/z* 328.17; $[M+H]^+$ calcd for $C_{17}H_{18}N_3O_2S^+$: 328.11

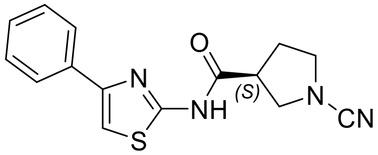

XL_10320_064C (15mg, 10%) ^{1}H NMR (500 MHz, DMSO) δ 12.45 (d, *J* = 25.3 Hz, 1H), 7.97 – 7.84 (m, 2H), 7.65 (d, *J* = 7.8 Hz, 1H), 7.44 (t, *J* = 7.7 Hz, 2H), 7.33 (t, *J* = 7.3 Hz, 1H), 3.64 (dd, *J* = 9.6, 7.8 Hz, 1H), 3.61 – 3.52 (m, 1H), 3.52 – 3.40 (m, 2H), 3.40 – 3.31 (m, 1H), 2.22 (td, *J* = 13.2, 7.4

Hz, 1H), 2.10 (dt, *J* = 19.7, 7.0 Hz, 1H). LC/MS (ESI) *m/z* 298.97; $[M+H]^+$ calcd for $C_{15}H_{15}N_4OS^+$: 299.10

Synthesis of XL_10320_065A, 065B, and 065C:

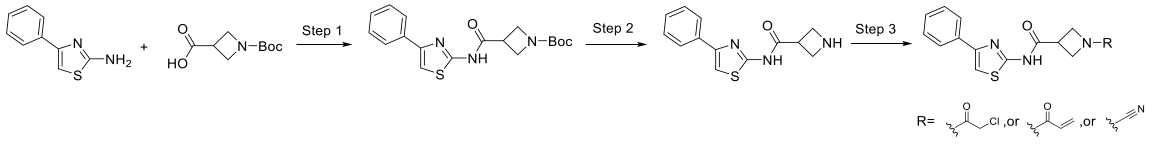

Step 1: The synthesis was preformed according to General Procedure 1 with 4-phenylthiazol-2-amine (0.26g, 1.5mmol) and 1-(tert-butoxycarbonyl)azetidine-3-carboxylic acid (0.36g, 1.8mmol). 0.52g desired compound (tert-butyl 3-((4-phenylthiazol-2-yl)carbamoyl)azetidine-1-carboxylate) was obtained (96%). LC/MS (ESI) *m/z* 260.17 (M+H−Boc); $[M+H]^+$ calcd for $C_{18}H_{22}N_3O_3S^+$: 360.14

Step 2: The synthesis was performed according to the General Procedure 1 with tert-butyl 3-((4-phenylthiazol-2-yl)carbamoyl)azetidine-1-carboxylate (0.52g, 1.5mmol). 0.46g N-(4-phenylthiazol-2-yl)azetidine-3-carboxamide was obtained (quant.)

Step 3: The synthesis was performed according to the General Procedure 1 with N-(4-phenylthiazol-2-yl)azetidine-3-carboxamide (0.15g, 0.6mmol) and chloroacetyl chloride (0.08mL, 1.0mmol), or acryloyl chloride (0.08mL, 1.0mmol), or cyanogen bromide (0.11g, 1.0mmol).

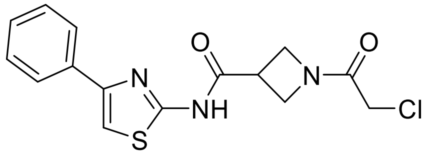

XL_10320_065A (33mg, 16%) ^{1}H NMR (500 MHz, DMSO) δ (ppm): 12.43 (s, 1H), 7.94 – 7.84 (m, 2H), 7.66 (s, 1H), 7.43 (dd, *J* = 10.6, 4.8 Hz, 2H), 7.36 – 7.27 (m, 1H), 4.45 – 4.32 (m, 2H), 4.17 (s, 2H), 4.13 – 4.00 (m, 2H), 3.76 – 3.70 (m, 1H). LC/MS (ESI) *m/z* 336.17; $[M+H]^+$ calcd for $C_{15}H_{15}ClN_3O_2S^+$: 336.06

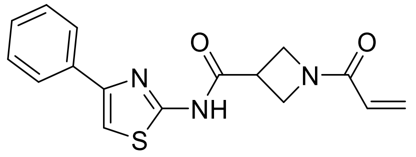

XL_10320_065B (18mg, 10%) ^{1}H NMR (500 MHz, DMSO) δ (ppm): 12.44 (s, 1H), 7.92 – 7.86 (m, 2H), 7.66 (s, 1H), 7.43 (t, *J* = 10.0Hz, 2H), 7.32 (t, *J* = 10.0 Hz, 1H), 6.32 (dd, *J* = 17.0, 10.3 Hz, 1H), 6.11 (dd, *J* = 17.0, 2.2 Hz, 1H), 5.68 (dd, *J* = 10.3, 2.2 Hz, 1H), 4.41 (t, *J* = 10.0 Hz, 1H), 4.35 (dd, *J* = 8.5, 5.8 Hz, 2H), 4.12 (t, *J* = 9.5 Hz, 2H), 4.04 (dd, *J* = 9.9, 5.8 Hz, 2H), 3.72 (tt, *J* = 8.9, 5.7 Hz, 1H). LC/MS (ESI) *m/z* 314.17; $[M+H]^+$ calcd for $C_{16}H_{16}N_3O_2S^+$: 314.10

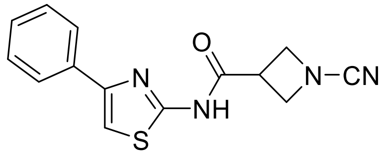

XL_10320_065C (3mg, 2%) ^{1}H NMR (500 MHz, DMSO) δ (ppm): 12.38 (s, 1H), 7.89 (d, *J* = 7.5 Hz, 2H), 7.66 (s, 1H), 7.43 (t, *J* = 7.6 Hz, 2H), 7.32 (t, *J* = 7.4 Hz, 1H), 4.32 (dt, *J* = 14.0, 7.7 Hz, 5H), 3.85 – 3.70 (m, 1H). LC/MS (ESI) *m/z* 285.07; $[M+H]^+$ calcd for $C_{14}H_{13}N_4OS^+$: 285.08

Synthesis of XL_10320_067A, 067B, 067C:

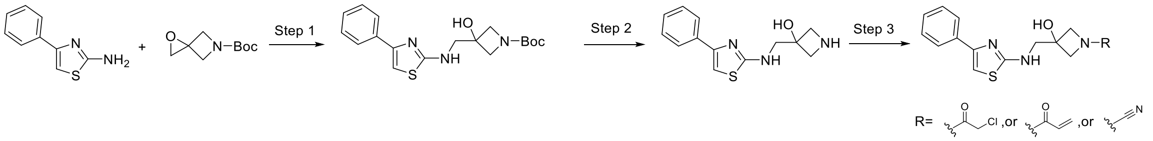

Step 1: The synthesis was performed according to General Procedure 2 with 4-phenylthiazol-2-amine (0.24g, 1.4mmol) and tert-butyl 1-oxa-5-azaspiro[2.3]hexane-5-carboxylate (0.25g, 1.4mmol). 0.17g desired product (tert-butyl 3-hydroxy-3-(((4-phenylthiazol-2-yl)amino)methyl)azetidine-1-carboxylate) was obtained (36%). LC/MS (ESI) *m/z* 361.97; $[M+H]^+$ calcd for $C_{18}H_{24}N_3O_3S^+$: 362.15

Step 2: The synthesis was performed according to the General Procedure 2 with tert-butyl 3-hydroxy-3-(((4-phenylthiazol-2-yl)amino)methyl)azetidine-1-carboxylate (0.12g, 0.3mmol). 0.084g 3-(((4-phenylthiazol-2-yl)amino)methyl)azetidin-3-ol was obtained (quant.)

Step 3: The synthesis was performed according to the General Procedure 2 with 3-(((4-phenylthiazol-2-yl)amino)methyl)azetidin-3-ol (0.028g, 0.1mmol), Et_3N (0.077mL, 0.55mmol) and chloroacetyl chloride (9.2µL, 0.11mmol), or acryloyl chloride (9.2µL, 0.11mmol), or cyanogen bromide (0.012g, 0.11mmol).

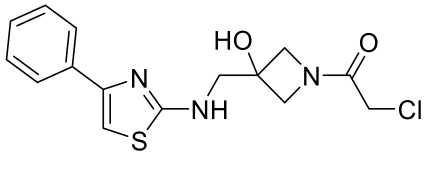

XL_10320_067A (8mg, 24%) ^{1}H NMR (500 MHz, DMSO) δ (ppm): 8.06 (s, 1H), 7.83 – 7.77 (m, 2H), 7.38 (t, *J* = 7.6 Hz, 2H), 7.29 (t, *J* = 7.3 Hz, 1H), 7.08 (s, 1H), 4.27 (d, *J* = 9.2 Hz, 1H), 4.19 – 4.08 (dd, *J* = 15.0, 5.0 Hz, 2H), 4.02 (m, 2H), 3.72 (d, *J* = 10.2 Hz, 1H), 3.61 (s, 2H). LC/MS (ESI) *m/z* 338.07; $[M+H]^+$ calcd for $C_{15}H_{17}ClN_3O_2S^+$: 338.07

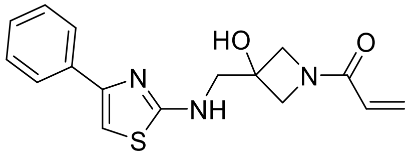

XL_10320_067B (8mg, 25%) ^{1}H NMR (500 MHz, DMSO) δ (ppm): 8.14 (s, 1H), 7.79 (d, *J* = 7.5 Hz, 2H), 7.38 (t, *J* = 7.7 Hz, 2H), 7.29 (t, *J* = 7.3 Hz, 1H), 7.08 (s, 1H), 6.32 (dd, *J* = 16.9, 10.3 Hz, 1H), 6.10 (dd, *J* = 17.0, 1.6 Hz, 1H), 5.65 (dd, *J* = 10.3, 1.6 Hz, 2H), 4.31 (d, *J* = 9.0 Hz, 1H), 4.01 (d, *J* = 9.8 Hz, 2H), 3.74 (d, *J* = 10.4 Hz, 1H), 3.62 (q, *J* = 13.8 Hz, 2H). LC/MS (ESI) *m/z* 315.87; $[M+H]^+$ calcd for $C_{16}H_{18}N_3O_2S^+$: 316.11

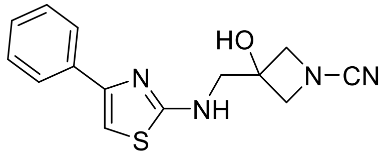

XL_10320_067C (15mg, 53%, mixture of rotamers) ^{1}H NMR (500 MHz, DMSO) δ (ppm): 7.84 – 7.77 (m, 2H), 7.43 – 7.36 (m, 2H), 7.29 (m, 1H), 7.11 – 7.04 (m, 1H), 4.19 (d, *J* = 8.5 Hz, 1H), 4.01 (d, *J* = 8.4 Hz, 1H), 3.83 (d, *J* = 9.7 Hz, 2H), 3.64-3.59 (m, 3H). LC/MS (ESI) *m/z* 286.97; $[M+H]^+$ calcd for $C_{14}H_{15}N_4OS^+$: 287.10

Synthesis of XL_10320_085A, 085B, 085C:

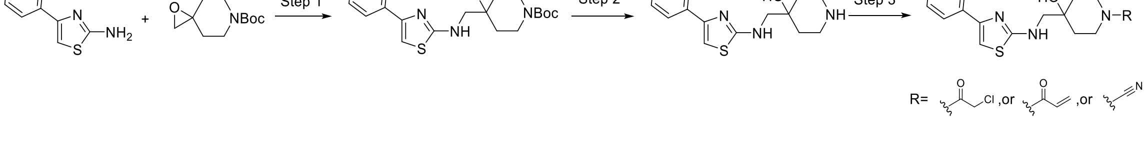

Step 1: The synthesis was performed according to General Procedure 2 with 4-phenylthiazol-2-amine (0.23g, 1.3mmol) and tert-butyl 1-oxa-6-azaspiro[2.5]octane-6-carboxylate (0.28g, 1.3mmol). 0.12g desired product (tert-butyl 4-hydroxy-4-(((4-phenylthiazol-2-yl)amino)methyl)piperidine-1-carboxylate) was obtained (24%). LC/MS (ESI) *m/z* 390.37; $[M+H]^+$ calcd for $C_{20}H_{28}N_3O_3S^+$: 390.18

Step 2: The synthesis was performed according to the General Procedure 2 tert-butyl 4-hydroxy-4-(((4-phenylthiazol-2-yl)amino)methyl)piperidine-1-carboxylate (0.12g, 0.3mmol) except for using 4N HCl in 1,4-dioxane instead of TFA/DCM. 0.08g 4-(((4-phenylthiazol-2-yl)amino)methyl)piperidin-4-ol was obtained (90%)

Step 3: The synthesis was performed according to the General Procedure 2 with 4-(((4-phenylthiazol-2-yl)amino)methyl)piperidin-4-ol (0.015g, 0.05mmol), Et_3N (0.077mL, 0.55mmol) and chloroacetyl chloride (5.0µL, 0.06mmol), or acryloyl chloride (5.0µL, 0.06mmol), or cyanogen bromide (0.007g, 0.06mmol).

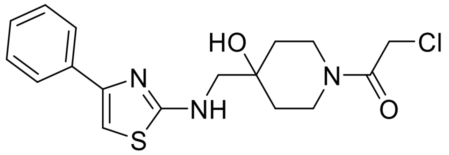

XL_10320_085A (4mg, 22%) 1H NMR (500 MHz, DMSO) δ 7.80 (m, J = 7.4 Hz, 2H), 7.38 (t, J = 7.6 Hz, 2H), 7.28 (t, J = 7.3 Hz, 1H), 7.05 (s, 1H), 4.42 – 4.31 (m, 2H), 4.07 (d, J = 12.9 Hz, 1H), 3.64 (d, J = 13.3 Hz, 1H), 3.42 – 3.28 (m, 3H), 3.02 (t, J = 10.5 Hz, 1H), 1.56 (tdd, J = 31.9, 20.6, 11.0 Hz, 4H). LC/MS (ESI) m/z 365.87; [M+H]+ calcd for $C_{17}H_{21}N_3O_2S^+$: 366.10

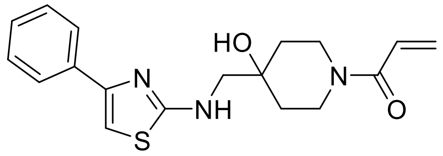

XL_10320_085B (4mg, 23%) 1H NMR (500 MHz, DMSO) δ 7.87 – 7.68 (m, 3H), 7.38 (t, J = 7.7 Hz, 2H), 7.27 (t, J = 7.4 Hz, 1H), 7.04 (s, 1H), 6.81 (dd, J = 16.7, 10.5 Hz, 1H), 6.08 (dd, J = 16.7, 2.5 Hz, 1H), 5.64 (dd, J = 10.4, 2.5 Hz, 1H), 4.15 (d, J = 13.3 Hz, 1H), 3.42 – 3.32 (m, 3H), 3.04 (t, J = 9.9 Hz, 1H), 1.53 (t, J = 10.7 Hz, 4H).). LC/MS (ESI) m/z 343.67; $[M+H]^+$ calcd for $C_{18}H_{22}N_3O_2S$+: 344.14

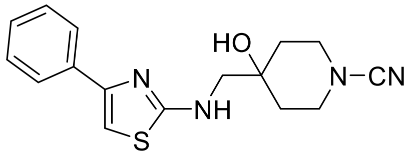

XL_10320_085C (6mg, 38%) ^{1}H NMR (500 MHz, DMSO) δ (ppm): 7.80 (m, 2H), 7.39 (m, 2H), 7.29 (m, 1H), 7.05 (s, 1H), 3.37 (m, 2H), 3.32 – 3.19 (m, 4H), 1.73 – 1.62 (m, 2H), 1.61 – 1.40 (m, 2H). LC/MS (ESI) *m/z* 314.67; $[M+H]^+$ calcd for $C_{16}H_{19}N_4OS^+$: 315.13

<u>Synthesis of XL_10320_086A, 086B, 086C:</u>

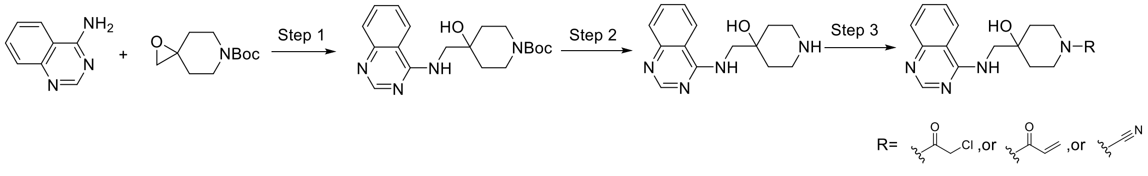

Step 1: The synthesis was performed according to General Procedure 2 with quinazolin-4-amine (0.29g, 2.0mmol) and tert-butyl 1-oxa-6-azaspiro[2.5]octane-6-carboxylate (0.42g, 2.0mmol). 0.35g desired product (tert-butyl 4-hydroxy-4-((quinazolin-4-ylamino)methyl)piperidine-1-carboxylate) was obtained (49%). LC/MS (ESI) *m/z* 359.27; $[M+H]^+$ calcd for $C_{19}H_{27}N_4O_3^+$: 359.21

Step 2: The synthesis was performed according to the General Procedure 2 with tert-butyl 4-hydroxy-4-((quinazolin-4-ylamino)methyl)piperidine-1-carboxylate (0.35g, 1.0mmol) except for using 4N HCl in 1,4-dioxane instead of TFA/DCM. 0.26g 4-((quinazolin-4-ylamino)methyl)piperidin-4-ol was obtained (quant.)

Step 3: The synthesis was performed according to the General Procedure 2 with 4-((quinazolin-4-ylamino)methyl)piperidin-4-ol (0.086g, 0.3mmol), Et_3N (0.077mL, 0.55mmol) and chloroacetyl chloride (43.0µL, 0.5mmol), or acryloyl chloride (43.0µL, 0.5mmol), or cyanogen bromide (0.57g, 0.5mmol).

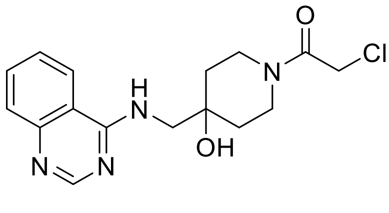

XL_10320_086A (31mg, 31%) ^{1}H NMR (500 MHz, DMSO) δ (ppm): 10.00 (t, *J* = 5.0 Hz, 1H), 8.89 (s, 1H), 8.59 (d, *J* = 8.0 Hz, 1H), 8.09 – 8.01 (m, 1H), 7.80 (m, 2H), 4.39 – 4.29 (d, *J* = 15.0, 20.0 Hz, 2H), 4.07 (d, *J* = 13.1 Hz, 1H), 3.81 (ddd, *J* = 30.7, 13.1, 6.2 Hz, 3H), 3.63 (d, *J* = 13.5 Hz, 1H), 3.41 – 3.26 (m, 1H), 2.99 (m, 1H), 1.72 – 1.43 (m, 4H). LC/MS (ESI) *m/z* 334.87; $[M+H]^+$ calcd for $C_{16}H_{20}ClN_4O_2^+$: 335.13

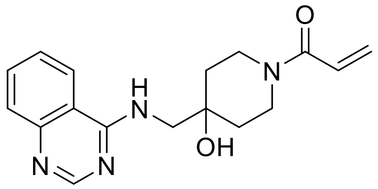

XL_10320_086B (53mg, 57%) ^{1}H NMR (500 MHz, DMSO) δ (ppm): 10.03 (t, *J* = 6.0 Hz, 1H), 8.89 (s, 1H), 8.59 (d, *J* = 7.9 Hz, 1H), 8.05 (t, *J* = 7.8 Hz, 1H), 7.80 (m, 2H), 6.79 (dd, *J* = 16.7, 10.5 Hz, 1H), 6.07 (dd, *J* = 16.7, 2.4 Hz, 1H), 5.64 (dd, *J* = 10.5, 2.4 Hz, 1H), 4.15 (d, *J* = 12.8 Hz, 1H), 3.89 – 3.74 (m, 3H), 3.34 (m, 1H), 3.02 (m, 1H), 1.54 (m, 4H). LC/MS (ESI) *m/z* 312.57; $[M+H]^+$ calcd for $C_{17}H_{21}N_4O_2^+$: 313.17

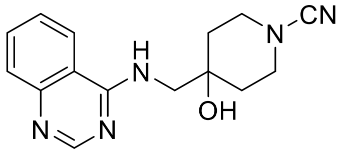

XL_10320_086C (28mg, 33%) ^{1}H NMR (500 MHz, DMSO) δ (ppm): 9.83 (s, 1H), 8.86 (s, 1H), 8.56 (d, *J* = 8.1 Hz, 1H), 8.03 (t, *J* = 7.3 Hz, 1H), 7.78 (m, 2H), 3.80 (d, *J* = 6.2 Hz, 2H), 3.29 – 3.19 (m, 5H), 1.77 – 1.64 (m, 2H), 1.58 (d, *J* = 13.4 Hz, 2H). LC/MS (ESI) *m/z* 283.77; $[M+H]^+$ calcd for $C_{15}H_{18}N_5O^+$: 284.15

Synthesis of XL_9678_159A, 159B, 159C, XL_9872_056, and AC_10180-18:

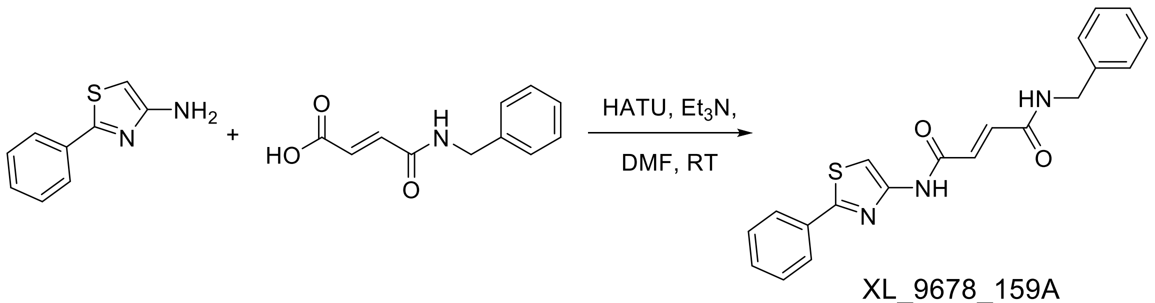

XL_9678_159A

2-phenylthiazol-4-amine (0.08g, 0.5mmol), (E)-4-(benzylamino)-4-oxobut-2-enoic acid (0.12g, 0.6mmol) Et_3N (0.35mL, 2.5mmol), and HATU (0.38g, 1.0mmol) were added sequentially to 3mL anhydrous DMF. The reaction mixture was stirred overnight at room temperature, and purified by flash chromatography (hexanes/EtOAc/MeOH) and preparative HPLC (MeOH/H_2O w/ 0.0425% TFA) to afford **XL_9678_159A** (20mg, 11%) ^1H NMR (500 MHz, DMSO) δ 12.79 (s, 1H), 9.10 (t, *J* = 5.8 Hz, 1H), 7.92 (d, *J* = 7.4 Hz, 2H), 7.71 (s, 1H), 7.44 (t, *J* = 7.6 Hz, 2H), 7.31 (m, 6H), 7.19 (d, *J* = 11.0 Hz, 2H), 4.42 (d, *J* = 5.8 Hz, 2H). LC/MS (ESI) *m/z* 363.87; $[M+H]^+$ calcd for $C_{20}H_{18}N_3O_2S^+$: 364.11

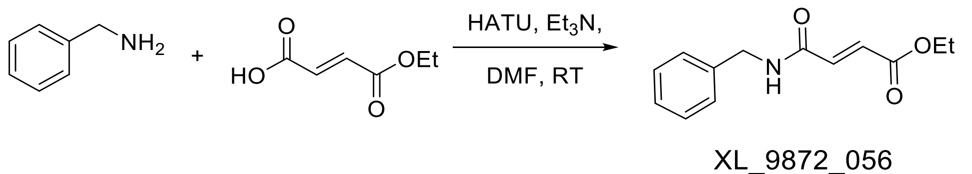

XL_9872_056

Benzylamine (0.11g, 1.0mmol), monoethyl fumarate (0.14g, 1.0mmol) Et3N (0.7mL, 5.0mmol), and HATU (0.57g, 1.5mmol) were added sequentially to 3mL anhydrous DMF. The reaction mixture was stirred overnight at room temperature, and purified by flash chromatography (hexanes/EtOAc/MeOH) and preparative HPLC (MeOH/H2O w/ 0.0425% TFA) to afford **XL_9872_056** (0.19g, 81%) 1H NMR (500 MHz, DMSO) δ 9.03 (t, J = 5.7 Hz, 1H), 7.37 – 7.31 (m, 2H), 7.31 – 7.24 (m, 3H), 7.07 (d, J = 15.5 Hz, 1H), 6.62 (d, J = 15.5 Hz, 1H), 4.40 (d, J = 5.9 Hz, 2H), 4.19 (q, J = 7.1 Hz, 2H), 1.25 (t, J = 7.1 Hz, 3H). LC/MS (ESI) m/z 234.08; [M+H]+ calcd for $C_{13}H_{16}NO_3^+$: 234.11

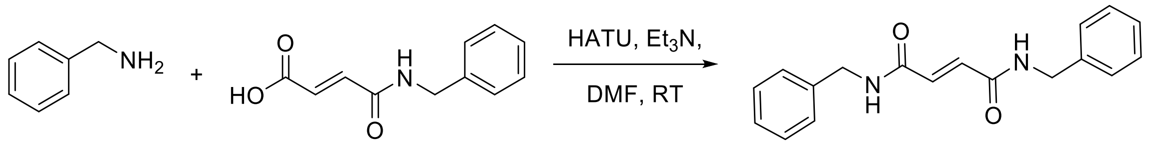

AC_10180_18

Benzylamine (0.026mL, 0.24mmol), (*E*)-4-(benzylamino)-4-oxobut-2-enoic acid (0.06g, 0.29mmol) Et_3N (0.17mL, 1.2mmol), and HATU (0.14g, 0.36mmol) were added sequentially to 1mL anhydrous DMF. The reaction mixture was stirred overnight at room temperature, and purified by flash chromatography (hexanes/EtOAc/MeOH) and trituation from diethyl ether to afford **AC-10180-18** (22mg, 31%) ^1H NMR (500 MHz, DMSO) δ 8.92 (t, *J* = 5.9 Hz, 2H), 7.39 – 7.30

(m, 4H), 7.30 – 7.20 (m, 6H), 6.93 (s, 2H), 4.38 (d, *J* = 6.0 Hz, 4H). LC/MS (ESI) *m/z* 294.80; $[M+H]^+$ calcd for $C_{18}H_{19}N_2O_2^+$: 295.14

2-

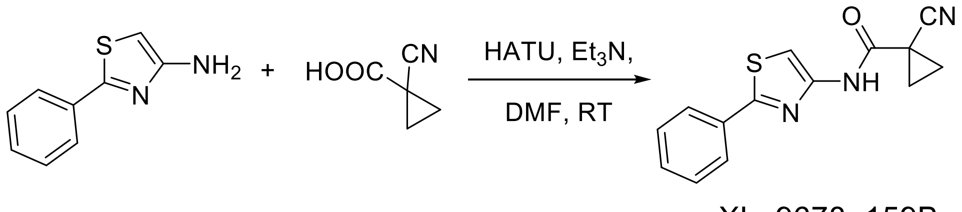

XL_9678_159B

phenylthiazol-4-amine (0.08g, 0.5mmol), 1-cyanocyclopropane-1-carboxylic acid (0.07g, 0.6mmol) Et_3N (0.35mL, 2.5mmol), and HATU (0.38g, 1.0mmol) were added sequentially to 3mL anhydrous DMF. The reaction mixture was stirred overnight at room temperature, and purified by flash chromatography (hexanes/EtOAc/MeOH) and preparative HPLC (MeOH/H_2O w/ 0.0425% TFA) to afford **XL_9678_159B** (40mg, 30%). ^1H NMR (500 MHz, DMSO) δ 12.43 (s, 1H), 7.91 (d, *J* = 7.6 Hz, 2H), 7.68 (s, 1H), 7.45 (t, *J* = 7.7 Hz, 2H), 7.35 (t, *J* = 7.3 Hz, 1H), 1.87 (br, 2H), 1.79 (br, 2H). LC/MS (ESI) *m/z* 269.87; $[M+H]^+$ calcd for $C_{14}H_{12}N_3OS^+$: 270.07

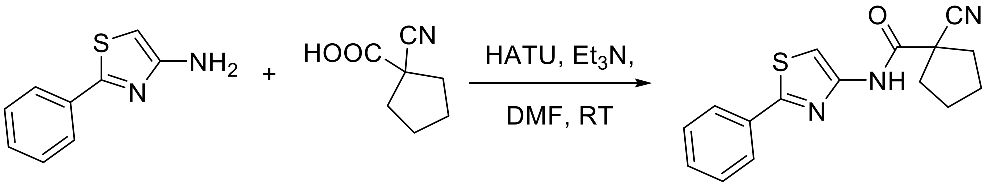

XL_9678_159C

2-phenylthiazol-4-amine (0.08g, 0.5mmol), 1-cyanocyclopentane-1-carboxylic acid (0.08g, 0.6mmol), Et_3N (0.35mL, 2.5mmol), and HATU (0.38g, 1.0mmol) were added sequentially to 3mL anhydrous DMF. The reaction mixture was stirred overnight at room temperature, and purified by flash chromatography (hexanes/EtOAc/MeOH) and preparative HPLC (MeOH/H_2O w/ 0.0425% TFA) to afford **XL_9678_159C** (56mg, 38%) ^1H NMR (500 MHz, DMSO) δ 12.92 (s, 1H), 7.93 (d, *J* = 7.5 Hz, 2H), 7.73 (s, 1H), 7.45 (t, *J* = 7.6 Hz, 2H), 7.35 (t, *J* = 7.3 Hz, 1H), 2.46 – 2.26 (m, 4H), 1.80 (m, 4H). LC/MS (ESI) *m/z* 297.87; $[M+H]^+$ calcd for $C_{16}H_{16}N_3OS^+$: 298.10

Synthesis of XL_9678_185B, 185C:

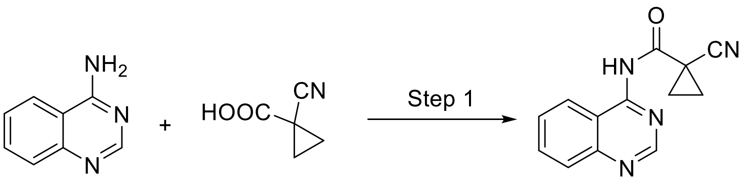

XL_9678_185B

Step 1: quinazolin-4-amine (0.043g, 0.3mmol), 1-cyanocyclopropane-1-carboxylic acid (0.044g, 0.4mmol), Et_3N (0.21mL, 1.5mL), and HATU (0.23g, 0.6mmol) were added into 3mL DMF. The reaction was stirred at room temperature overnight. The reaction mixture was stirred overnight at room temperature, and purified by flash chromatography (hexanes/EtOAc/MeOH) and preparative HPLC (MeOH/H_2O w/ 0.0425% TFA) to afford **XL_9678_185B**. ^1H NMR (500 MHz, DMSO) δ 13.60 (s, 1H), 8.48 (d, *J* = 8.0 Hz, 1H), 8.41 (s, 1H), 8.03 – 7.94 (m, 1H), 7.81 (d, *J* = 8.2 Hz, 1H), 7.75 – 7.64 (m, 1H), 1.78 – 1.67 (m, 4H). LC/MS (ESI) *m/z* 238.98; $[M+H]^+$ calcd for $C_{13}H_{11}N_4O^+$: 239.09.

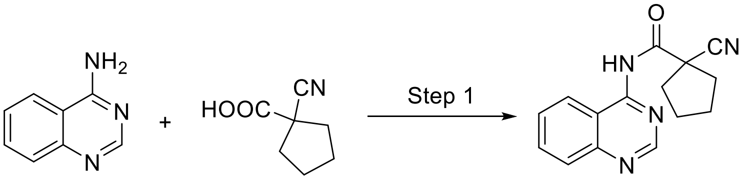

XL_9678_185C

Step 1: quinazolin-4-amine (0.043g, 0.3mmol), 1-cyanocyclopentane-1-carboxylic acid (0.055g, 0.4mmol), Et3N (0.21mL, 1.5mL), and HATU (0.23g, 0.6mmol) were added into 3mL DMF. The reaction was stirred at room temperature overnight. The reaction mixture was stirred overnight at room temperature, and purified by flash chromatography (hexanes/EtOAc/MeOH) and preparative HPLC (MeOH/H2O w/ 0.0425% TFA) to afford **XL_9678_185C** (2mg, 3%). 1H NMR (500 MHz, DMSO) δ 8.42 (br, 2H), 8.00 (t, J = 7.2 Hz, 1H), 7.85 (d, J = 6.7 Hz, 1H), 7.72 (t, J = 7.6 Hz, 1H), 2.37 (m, 2H), 2.25 (m, 2H), 1.92 – 1.78 (m, 4H). LC/MS (ESI) *m/z* 267.10; $[M+H]^+$ calcd for $C_{15}H_{15}N_4O^+$: 267.12.

<u>Synthesis of XL_9872_111B</u>:

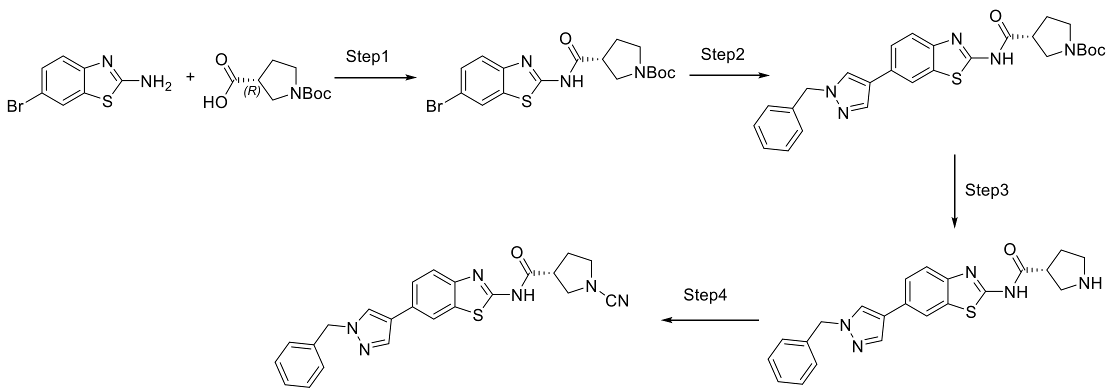

Step 1: 6-bromobenzo[d]thiazol-2-amine (0.41g, 1.8mmol), (R)-1-(tert-butoxycarbonyl)pyrrolidine-3-carboxylic acid (0.47g, 2.2mmol), Et_3N (1.2mL, 9.0mmol) and HATU (1.03g, 2.7mmol) were added sequentially in anhydrous DMF (5mL). The mixture was stirred at room temperature overnight. The mixture was then diluted with EtOAc (50mL), and washed with brine (30mL×2) to remove excess DMF. Organic layer was dried over anhydrous sodium sulfate (Na_2SO_4), filtered, and concentrated under reduced pressure. The crude material was then purified by flash column chromatography (hexanes/EtOAc/MeOH) to afford 0.72g (94%) material. LC/MS (ESI) *m/z* 426.27; $[M+H]^+$ calcd for $C_{17}H_{21}BrN_3O_3S^+$: 426.05.

Step 2: The isolated product tert-butyl (R)-3-((6-bromobenzo[d]thiazol-2-yl)carbamoyl)pyrrolidine-1-carboxylate from step 1 (0.064g, 0.15mmol) was dissolved in 1,4-dioxane and H_2O (4mL, 3:1). Into the solution were added 1-benzyl-4-(4,4,5,5-tetramethyl-1,3,2-dioxaborolan-2-yl)-1H-pyrazole (0.09g, 0.45mmol), potassium carbonate (0.062g, 0.45mmol) and $Pd(PPh_3)_4$ (0.035g, 0.03mmol). The mixture was degassed by bubbling through N_2 for 10min before heating up to 95°C and stirred at this temperature for 2-8 hours. The reaction was then cooled down to room temperature and diluted with EtOAc (50mL). The organic phase was washed with saturated ammonium chloride (30mL×2). Aqueous layer was then extracted with more EtOAc (50mL). Combined organic layers were washed with brine, dried over anhydrous sodium sulfate (Na_2SO_4), filtered, and concentrated under reduced pressure to afford crude material, which was then purified by flash chromatography (hexanes/EtOAc/MeOH) to afford 0.01g product (13%) LC/MS (ESI) *m/z* 503.88; $[M+H]^+$ calcd for $C_{27}H_{30}N_5O_3S^+$: 504.21.

Step 3: Products from last step were dissolved in DCM (1mL) and treated with TFA (1mL). The mixtures were stirred at room temperature until the tert-butyloxycarbonyl protecting group was

cleaved tracking by UPLC-MS. The mixture was concentrated and flushed by flash column chromatography (EtOAc/MeOH/0.5%Et_3N).

Step 4: Products from the last step (0.008g, 0.02mmol) were dissolved in DCM (2mL) with Et_3N (14µL, 0.1mmol) at 0°C. Cyanogen bromide 3M solution in DCM (13µL, 0.04mmol) was then added. The mixture was then stirred at 0°C for 1 hour, and directly purified by flash chromatography (hexanes/EtOAc/MeOH) followed by preparative HPLC (MeOH or CH_3CN/H_2O with 0.0425% TFA) to afford the target product.

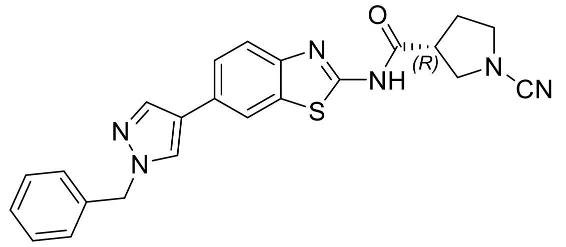

XL_9872_111B (4mg, 47%) ^1H NMR (500 MHz, DMSO) δ 12.52 (s, 1H), 8.32 (s, 1H), 8.19 (s, 1H), 7.97 (s, 1H), 7.71 (d, *J* = 8.4 Hz, 1H), 7.65 (d, *J* = 8.4 Hz, 1H), 7.37 (m, 2H), 7.30 (m, 3H), 5.36 (s, 2H), 3.69 – 3.62 (m, 1H), 3.58 (dd, *J* = 9.6, 6.1 Hz, 1H), 3.46 (m, 2H), 3.41 – 3.34 (m, 1H), 2.23 (dt, *J* = 13.1, 7.3 Hz, 1H), 2.11 (dt, *J* = 19.7, 7.0 Hz, 1H). LC/MS (ESI) *m/z* 428.87; $[M+H]^+$ calcd for $C_{23}H_{21}N_6OS^+$: 429.15

Synthesis of XL_9872_111F:

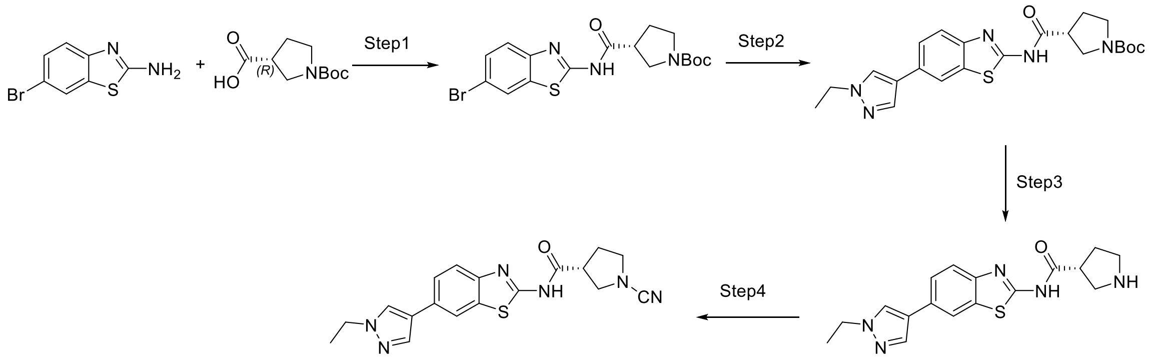

Step 1: 6-bromobenzo[d]thiazol-2-amine (0.41g, 1.8mmol), (R)-1-(tert-butoxycarbonyl)pyrrolidine-3-carboxylic acid (0.47g, 2.2mmol), Et3N (1.2mL, 9.0mmol) and HATU (1.03g, 2.7mmol) were added sequentially in anhydrous DMF (5mL). The mixture was stirred at room temperature overnight. The mixture was then diluted with EtOAc (50mL), and

washed with brine (30mL×2) to remove excess DMF. Organic layer was dried over anhydrous sodium sulfate (Na_2SO_4), filtered, and concentrated under reduced pressure. The crude material was then purified by flash column chromatography (hexanes/EtOAc/MeOH) to afford 0.72g (94%) material. LC/MS (ESI) *m/z* 426.27; $[M+H]^+$ calcd for $C_{17}H_{21}BrN_3O_3S^+$: 426.05.

Step 2: The isolated product tert-butyl (R)-3-((6-bromobenzo[d]thiazol-2-yl)carbamoyl)pyrrolidine-1-carboxylate from step 1 (0.064g, 0.15mmol) was dissolved in 1,4-dioxane and H_2O (4mL, 3:1). Into the solution were added 1-ethyl-4-(4,4,5,5-tetramethyl-1,3,2-dioxaborolan-2-yl)-1H-pyrazole (0.1g, 0.45mmol), potassium carbonate (0.062g, 0.45mmol) and $Pd(PPh_3)_4$ (0.035g, 0.03mmol). The mixture was degassed by bubbling through N_2 for 10min before heating up to 95°C and stirred at this temperature for 2-8 hours. The reaction was then cooled down to room temperature and diluted with EtOAc (50mL). The organic phase was washed with saturated ammonium chloride (30mL×2). Aqueous layer was then extracted with more EtOAc (50mL). Combined organic layers were washed with brine, dried over anhydrous sodium sulfate (Na_2SO_4), filtered, and concentrated under reduced pressure to afford crude material, which was then purified by flash chromatography (hexanes/EtOAc/MeOH) to afford 0.05g product (76%). LC/MS (ESI) *m/z* 441.88; $[M+H]^+$ calcd for $C_{22}H_{28}N_5O_3S^+$: 442.19.

Step 3: Products from last step were dissolved in DCM (1mL) and treated with TFA (1mL). The mixtures were stirred at room temperature until the tert-butyloxycarbonyl protecting group was cleaved tracking by UPLC-MS. The mixture was concentrated and flushed by flash column chromatography (EtOAc/MeOH/0.5%Et_3N).

Step 4: Products from the last step (0.041g, 0.12mmol) were dissolved in DCM (2mL) with Et_3N (84µL, 0.6mmol) at 0°C. Cyanogen bromide 3M solution in DCM (60µL, 0.24mmol) was then added. The mixture was then stirred at 0°C for 1 hour, and directly purified by flash chromatography (hexanes/EtOAc/MeOH) followed by preparative HPLC (MeOH or CH_3CN/H_2O with 0.0425% TFA) to afford the target product.

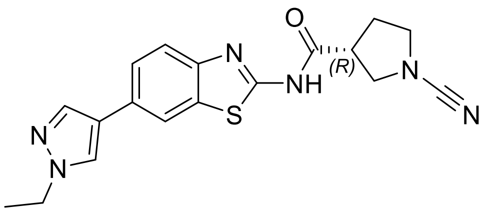

XL_9872_111F (10mg, 23%) ^{1}H NMR (500 MHz, DMSO) δ 12.53 (s, 1H), 8.23 (s, 1H), 8.19 (d, *J* = 1.5 Hz, 1H), 7.92 (d, *J* = 0.6 Hz, 1H), 7.72 (d, *J* = 8.4 Hz, 1H), 7.68 – 7.64 (dd, *J* = 1.7, 8.5 Hz, 1H), 4.21 – 4.11 (q, *J* = 7.4 Hz, 2H), 3.65 (dd, *J* = 9.6, 7.7 Hz, 1H), 3.59 (dd, *J* = 9.6, 6.0 Hz, 1H), 3.52 – 3.42 (m, 2H), 3.42 – 3.35 (m, 1H), 2.24 (dt, *J* = 13.4, 7.5 Hz, 1H), 2.11 (dt, *J* = 14.3, 6.9 Hz, 1H), 1.42 (t, *J* = 7.3 Hz, 3H). LC/MS (ESI) *m/z* 366.87; $[M+H]^+$ calcd for $C_{18}H_{19}N_6OS^+$: 367.13

Synthesis of XL_9872_106A, 106B, 106C:

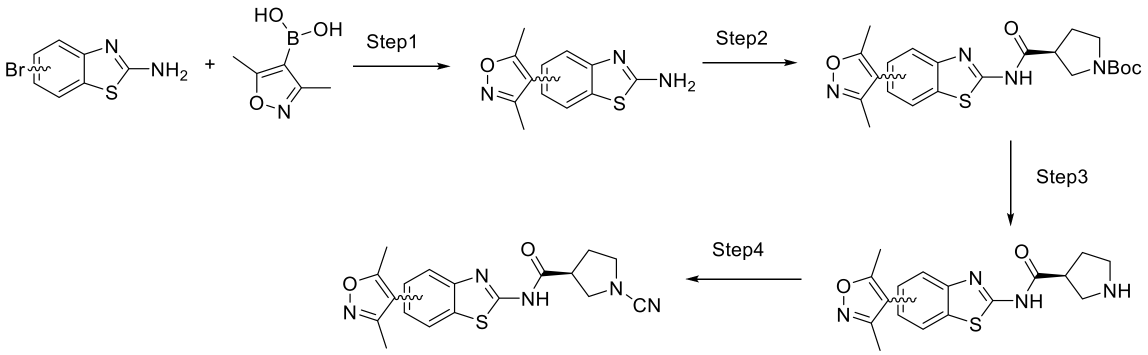

Step 1: 2-amino-4-bromobenzothiazole (0.69g, 3.0mmol), 2-amino-5-bromobenzothiazole (0.69g, 3.0mmol), or 2-amino-7-bromobenzothiazole (0.69g, 3.0mmol) were used as starting material in the synthesis described in Step 1 of General Procedure 4 to afford desired products (4-(3,5-dimethylisoxazol-4-yl)benzo[d]thiazol-2-amine: 0.6g (82%) LC/MS (ESI) *m/z* 245.98; $[M+H]^+$ calcd for $C_{12}H_{12}N_3OS^+$: 246.07; 5-(3,5-dimethylisoxazol-4-yl)benzo[d]thiazol-2-amine: 0.58g (79%) LC/MS (ESI) *m/z* 245.98; $[M+H]^+$ calcd for $C_{12}H_{12}N_3OS^+$: 246.07; 7-(3,5-dimethylisoxazol-4-yl)benzo[d]thiazol-2-amine: 0.39g (53%) LC/MS (ESI) *m/z* 245.88; $[M+H]^+$ calcd for $C_{12}H_{12}N_3OS^+$: 246.07.

Step 2: The isolated products from Step 1 (0.05g, 0.2mmol) were used in Step 2 described in General Procedure 4 to afford desired products (tert-butyl (S)-3-((4-(3,5-dimethylisoxazol-4-yl)benzo[d]thiazol-2-yl)carbamoyl)pyrrolidine-1-carboxylate: 0.092g (84%); LC/MS (ESI) m/z 443.08; [M+H]+ calcd for $C_{22}H_{27}N_4O_4S^+$: 443.17; tert-butyl (S)-3-((5-(3,5-dimethylisoxazol-4-

yl)benzo[d]thiazol-2-yl)carbamoyl)pyrrolidine-1-carboxylate: 0.19g (over 100%) LC/MS (ESI) m/z 443.08; [M+H]+ calcd for $C_{22}H_{27}N_4O_4S^+$: 443.17; tert-butyl (S)-3-((7-(3,5-dimethylisoxazol-4-yl)benzo[d]thiazol-2-yl)carbamoyl)pyrrolidine-1-carboxylate: 0.20g (over 100%) LC/MS (ESI) *m/z* 387.27 (M+H−*t*-Butyl); $[M+H]^+$ calcd for $C_{22}H_{27}N_4O_4S^+$: 443.54).

Step 3: The isolated products from Step 2 were used in Step 3 described in General Procedure 4 to afford desired products ((S)-N-(4-(3,5-dimethylisoxazol-4-yl)benzo[d]thiazol-2-yl)pyrrolidine-3-carboxamide: 0.08g (quant.); (S)-N-(5-(3,5-dimethylisoxazol-4-yl)benzo[d]thiazol-2-yl)pyrrolidine-3-carboxamide: 0.08g (quant.); (S)-N-(7-(3,5-dimethylisoxazol-4-yl)benzo[d]thiazol-2-yl)pyrrolidine-3-carboxamide: 0.07g (quant.)).

Step 4: The isolated products from Step 3 (0.072g, 0.21mmol) were used in Step 4 described in General Procedure 4 to afford desired products.

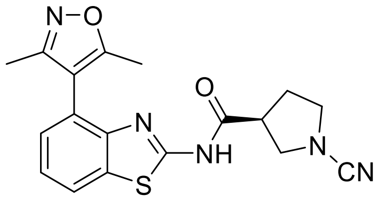

XL_9872_106A (0.026g, 34%) ^{1}H NMR (500 MHz, DMSO) δ 12.60 (s, 1H), 8.10 – 7.99 (m, 1H), 7.45 – 7.35 (m, 2H), 3.63 (dd, *J* = 9.6, 7.8 Hz, 1H), 3.57 (dd, *J* = 9.7, 6.0 Hz, 1H), 3.51 – 3.40 (m, 2H), 3.37 (dd, *J* = 13.8, 6.9 Hz, 1H), 2.31 (s, 3H), 2.27 – 2.16 (m, 1H), 2.16 – 2.03 (m, 4H). LC/MS (ESI) *m/z* 367.77; $[M+H]^+$ calcd for $C_{18}H_{18}N_5O_2S^+$: 368.12

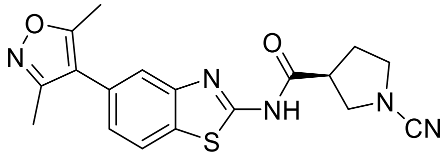

XL_9872_106B (0.022g, 29%) ^{1}H NMR (500 MHz, DMSO) δ 12.59 (s, 1H), 8.09 (d, *J* = 8.2 Hz, 1H), 7.74 (s, 1H), 7.31 (dd, *J* = 19.8, 9.9 Hz, 1H), 3.70 – 3.63 (m, 1H), 3.60 (dd, *J* = 9.6, 6.1 Hz, 1H), 3.52 – 3.37 (m, 3H), 2.43 (s, 3H), 2.28 – 2.21 (m, 4H), 2.12 (td, *J* = 14.1, 7.1 Hz, 1H). LC/MS (ESI) *m/z* 367.87; $[M+H]^+$ calcd for $C_{18}H_{18}N_5O_2S^+$: 368.12

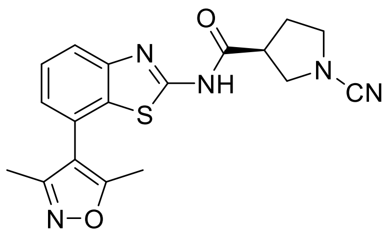

XL_9872_106C (0.029g, 40%) 1H NMR (500 MHz, DMSO) δ 7.82 (dd, J = 8.1, 0.9 Hz, 1H), 7.61 – 7.51 (m, 1H), 7.28 (dd, J = 7.4, 0.9 Hz, 1H), 3.68 – 3.60 (m, 1H), 3.56 (dd, J = 9.7, 5.9 Hz, 1H), 3.48 – 3.41 (m, 2H), 3.39 (dd, J = 13.5, 6.8 Hz, 1H), 2.31 (s, 3H), 2.21 (tt, J = 12.8, 6.4 Hz, 1H), 2.14 – 2.04 (m, 4H). LC/MS (ESI) m/z 367.97; [M+H]+ calcd for $C_{18}H_{18}N_5O_2S^+$: 368.12.

Synthesis of XL_9872_123A, 123B, 123C:

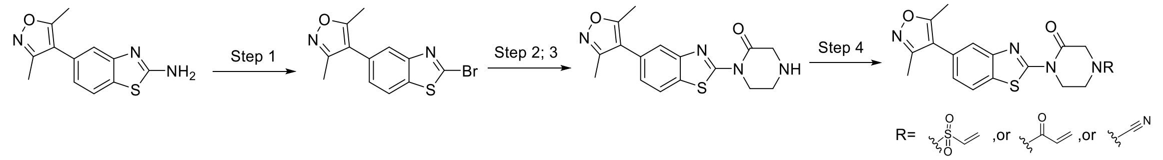

Synthesis were performed according to General Procedure 5.

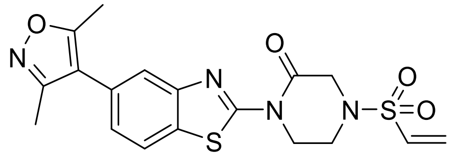

XL_9872_123A (19mg, 45%) ^{1}H NMR (500 MHz, DMSO) δ 8.13 (d, *J* = 8.2 Hz, 1H), 7.86 (d, *J* = 1.4 Hz, 1H), 7.39 (dd, *J* = 8.2, 1.6 Hz, 1H), 7.00 (dd, *J* = 16.5, 10.0 Hz, 1H), 6.28 (dd, *J* = 13.2, 3.3 Hz, 2H), 4.38 – 4.29 (m, 2H), 4.18 (s, 2H), 3.73 – 3.61 (m, 2H), 2.44 (s, 3H), 2.27 (s, 3H). ^{13}C NMR (126 MHz, DMSO) δ 165.75, 165.48, 159.16, 158.73, 148.42, 132.90, 132.27, 131.14, 128.57, 125.60, 122.66, 121.79, 116.29, 48.93, 47.24, 42.34, 11.82, 10.95. LC/MS (ESI) *m/z* 418.87; $[M+H]^+$ calcd for $C_{18}H_{19}N_4O_4S_2^+$: 419.08

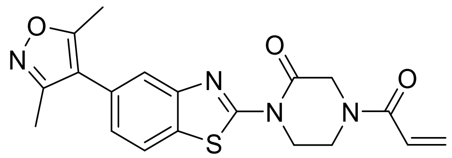

XL_9872_123B (16mg, 42%) ^{1}H NMR (500 MHz, DMSO) δ 8.12 (d, *J* = 8.2 Hz, 1H), 7.85 (s, 1H), 7.37 (dd, *J* = 8.2, 1.5 Hz, 1H), 6.98 – 6.78 (m, 1H), 6.22 (d, *J* = 16.6 Hz, 1H), 5.80 (d, *J* = 11.0 Hz,

1H), 4.68 (s, 1H), 4.50 (s, 1H), 4.32 (br, 2H), 4.04 (br, 2H), 2.44 (s, 3H), 2.27 (s, 3H). LC/MS (ESI) *m/z* 382.87; $[M+H]^+$ calcd for $C_{19}H_{19}N_4O_3S^+$: 383.12

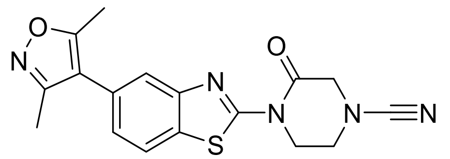

XL_9872_123C (16mg, 45%) ^1H NMR (500 MHz, DMSO) δ 8.13 (d, *J* = 8.2 Hz, 1H), 7.86 (s, 1H), 7.38 (d, *J* = 8.2 Hz, 1H), 4.36 (m, 4H), 3.85 – 3.72 (m, 2H), 2.44 (s, 3H), 2.27 (s, 3H). LC/MS (ESI) *m/z* 353.87; $[M+H]^+$ calcd for $C_{17}H_{16}N_5O_2S^+$: 354.10

Synthesis of XL_9872_126A, 126B, 128A, 128B:

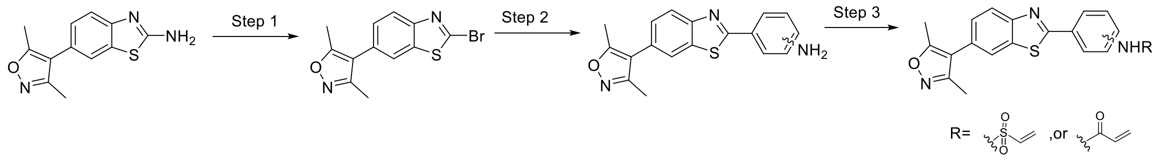

Synthesis was performed according to General Procedure 6.

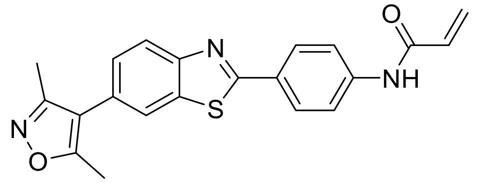

XL_9872_126A (22mg, 45%) ^1H NMR (500 MHz, DMSO) δ 10.49 (s, 1H), 8.19 (d, *J* = 1.6 Hz, 1H), 8.10 (dd, *J* = 7.1, 1.7 Hz, 3H), 7.90 (d, *J* = 8.7 Hz, 2H), 7.54 (dd, *J* = 8.4, 1.7 Hz, 1H), 6.56 – 6.44 (m, 1H), 6.33 (dd, *J* = 17.0, 1.8 Hz, 1H), 5.83 (dd, *J* = 10.1, 1.8 Hz, 1H), 2.47 (s, 3H), 2.29 (s, 3H). ^{13}C NMR (126 MHz, DMSO) δ 167.99, 165.88, 163.98, 158.69, 153.32, 142.42, 135.49, 132.04, 128.56, 128.23, 128.11, 128.03, 127.40, 123.17, 122.88, 120.09, 116.06, 11.87, 10.98. LC/MS (ESI) *m/z* 375.97; $[M+H]^+$ calcd for $C_{21}H_{18}N_3O_2S^+$: 376.11

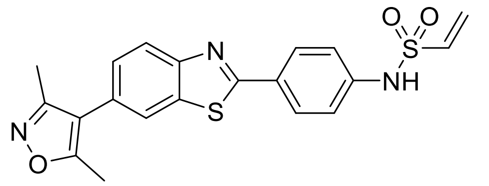

XL_9872_126B (7mg, 13%) ^{1}H NMR (500 MHz, DMSO) δ 10.53 (s, 1H), 8.20 (d, *J* = 1.5 Hz, 1H), 8.08 (dd, *J* = 16.1, 8.5 Hz, 3H), 7.54 (dd, *J* = 8.4, 1.6 Hz, 1H), 7.34 (d, *J* = 8.7 Hz, 2H), 6.89 (dd, *J* = 16.4, 9.9 Hz, 1H), 6.25 (d, *J* = 16.4 Hz, 1H), 6.13 (d, *J* = 9.9 Hz, 1H), 2.46 (s, 3H), 2.29 (s, 3H). LC/MS (ESI) *m/z* 411.87; $[M+H]^+$ calcd for $C_{20}H_{18}N_3O_3S_2^+$: 412.08

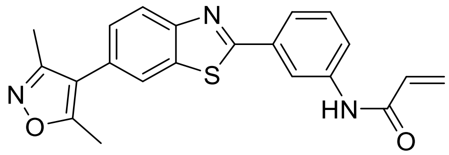

XL_9872_128A (25mg, 60%) ^{1}H NMR (500 MHz, DMSO) δ 10.44 (s, 1H), 8.59 (s, 1H), 8.23 (s, 1H), 8.15 (d, *J* = 8.4 Hz, 1H), 7.87 (d, *J* = 8.2 Hz, 1H), 7.81 (d, *J* = 7.7 Hz, 1H), 7.64 – 7.49 (m, 2H), 6.48 (dd, *J* = 17.0, 10.1 Hz, 1H), 6.33 (dd, *J* = 17.0, 1.6 Hz, 1H), 5.87 – 5.78 (m, 1H), 2.47 (s, 3H), 2.30 (s, 3H). LC/MS (ESI) *m/z* 375.97; $[M+H]^+$ calcd for $C_{21}H_{18}N_3O_2S^+$: 376.11

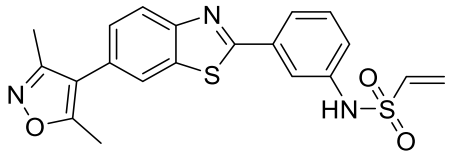

XL_9872_128B (17mg, 38%) ^{1}H NMR (500 MHz, DMSO) δ 10.35 (s, 1H), 8.23 (d, *J* = 1.5 Hz, 1H), 8.16 (d, *J* = 8.4 Hz, 1H), 7.95 (t, *J* = 1.8 Hz, 1H), 7.81 (d, *J* = 7.8 Hz, 1H), 7.65 – 7.45 (m, 2H), 7.38 (dd, *J* = 8.1, 1.6 Hz, 1H), 6.88 (dd, *J* = 16.4, 9.9 Hz, 1H), 6.20 (d, *J* = 16.4 Hz, 1H), 6.11 (d, *J* = 9.9 Hz, 1H), 2.47 (s, 3H), 2.30 (s, 3H). LC/MS (ESI) *m/z* 411.87; $[M+H]^+$ calcd for $C_{20}H_{18}N_3O_3S_2^+$: 412.08

Synthesis of XL_9872_151A, 151B, 151C:

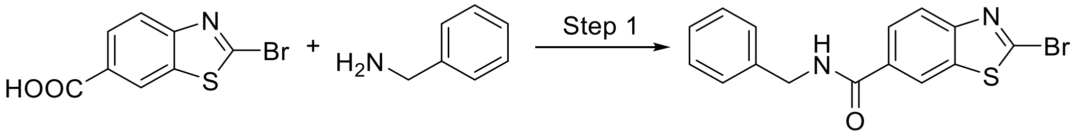

XL_9872_151A

XL_9872_151A (0.11g, 64%) ^{1}H NMR (500 MHz, DMSO) δ 9.21 (t, *J* = 5.8 Hz, 1H), 8.65 (s, 1H), 8.08 (d, *J* = 8.5 Hz, 1H), 8.04 (dd, *J* = 8.5, 1.4 Hz, 1H), 7.39 – 7.30 (m, 4H), 7.30 – 7.20 (m, 1H), 4.52 (d, *J* = 5.9 Hz, 2H). LC/MS (ESI) *m/z* 346.77; $[M+H]^+$ calcd for $C_{15}H_{12}BrN_2OS^+$: 346.98

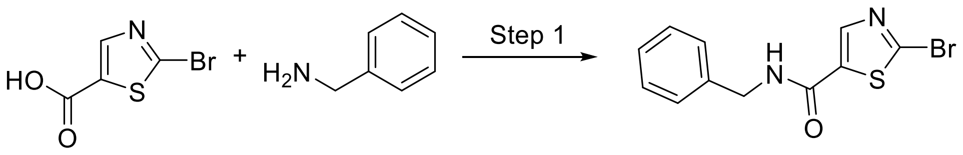

XL_9872_151B

XL_9872_151B (0.075g, 51%) ^{1}H NMR (500 MHz, DMSO) δ 9.08 (t, *J* = 6.1 Hz, 1H), 8.31 (s, 1H), 7.37 – 7.27 (m, 4H), 7.27 – 7.19 (m, 1H), 4.43 (d, *J* = 6.3 Hz, 2H). LC/MS (ESI) *m/z* 296.77; $[M+H]^+$ calcd for $C_{11}H_{10}BrN_2OS^+$: 296.97

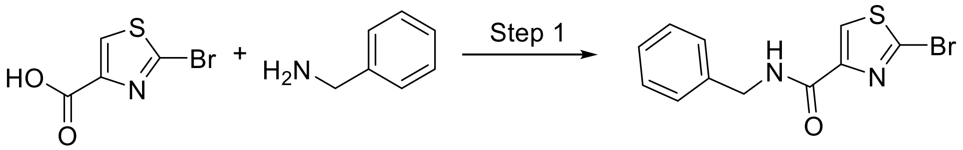

XL_9872_151C

XL_9872_151C ^{1}H NMR (500 MHz, DMSO) δ 9.32 (t, *J* = 5.8 Hz, 1H), 8.28 (d, *J* = 5.6 Hz, 1H), 7.39 – 7.30 (m, 4H), 7.30 – 7.21 (m, 1H), 4.46 (d, *J* = 5.9 Hz, 2H). LC/MS (ESI) *m/z* 296.87; $[M+H]^+$ calcd for $C_{11}H_{10}BrN_2OS^+$: 296.97

Synthesis of XL_9872_159:

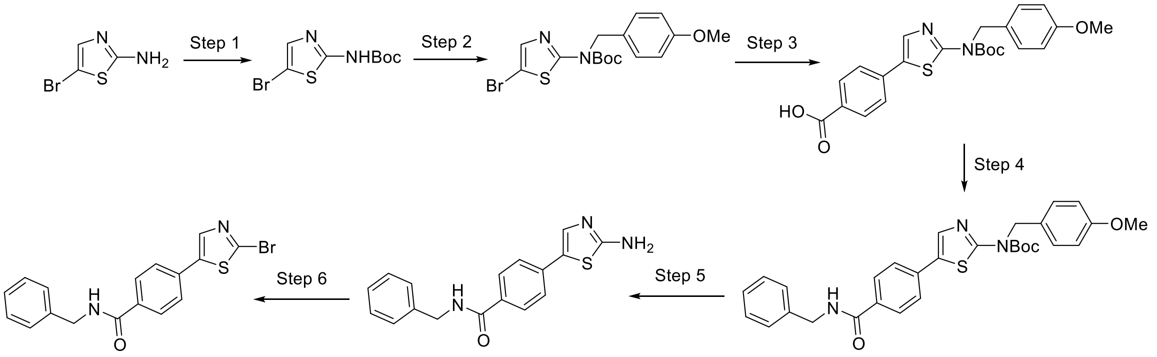

Step 1: 2-amino-5-bromothiazole hydrobromide (1.3g, 5.0mmol), di-tert-butyl decarbonate (1.3g, 6.0mmol), Et3N (1.4mL, 10.0mmol), and DMAP (0.06g, 0.5mmol) were added into 5mL THF sequentially. The mixture was stirred at room temperature overnight, and diluted with EtOAc (30mL). The solution was washed with saturated sodium bicarbonate (30mL×2). Combined aqueous layers was extracted with EtOAc (50mL). Combined organic layer was washed with brine, dried over anhydrous Na_2SO_4, filtered, and concentrated under reduced pressure to afford crude material. The crude was purified by flash chromatography

(hexanes/EtOAc) to afford desired product. LC/MS (ESI) *m/z* 222.88 (M+H−*t*-butyl); $[M+H]^+$ calcd for $C_8H_{12}BrN_2O_2S^+$: 278.98

Step 2: tert-Butyl (5-bromothiazol-2-yl)carbamate (0.57g, 2.1mmol), PPh_3 (1.18g, 4.5mmol), and p-methoxybenzyl alcohol (0.57g, 4.1mmol) were mixed up in 10mL anhydrous THF. The mixture was stirred at 0°C while DIAD (0.91g, 4.5mmol) was added dropwisely. Upon completion, the mixture was warmed to room temperature, and stirred for 2 hours. The resulting reaction mixture was diluted with EtOAc (30mL). The solution was washed with saturated sodium bicarbonate (30mL×2). Combined aqueous layers was extracted with EtOAc (50mL). Combined organic layer was washed with brine, dried over anhydrous Na_2SO_4, filtered, and concentrated under reduced pressure to afford crude material. The crude was purified by flash chromatography (hexanes/EtOAc) to afford desired product (0.60g, 74%). LC/MS (ESI) *m/z* 342.24 (M+H−*t*-butyl); $[M+H]^+$ calcd for $C_{16}H_{20}BrN_2O_3S^+$: 399.04

Step 3: tert-butyl (5-bromothiazol-2-yl)(4-methoxybenzyl)carbamate (0.2g, 0.5mmol), 4-(4,4,5,5-tetramethyl-1,3,2-dioxaborolan-2-yl)benzoic acid (0.26g, 1.0mmol), potassium carbonate (0.14g, 1.0mmol) and $Pd(dppf)Cl_2$ (0.04g, 0.05mmol) were added into 1,4-dioxane/H_2O (3mL/0.5mL). The mixture was purged with N_2 for 10min before stirring at 95°C overnight under N_2. Then the mixture was concentrated under reduced pressure, diluted with EtOAc (30mL), and washed with saturated NH_4Cl (30mL×2). Combined aqueous layer was extracted with EtOAc (50mL). Combined organic layer was washed once with brine, dried over anhydrous Na_2SO_4, filtered, and evaporated under reduced pressure to afford crude material, which was then purified by flash chromatography (hexanes/EtOAc/MeOH) to afford desired product (0.1g, 45%). LC/MS (ESI) *m/z* 384.77 (M+H−*t*-butyl); $[M+H]^+$ calcd for $C_{23}H_{25}N_2O_5S^+$: 441.15

Step 4: 4-(2-((tert-butoxycarbonyl)(4-methoxybenzyl)amino)thiazol-5-yl)benzoic acid (0.1g, 0.23mmol), benzylamine (0.03g, 0.28mmol), Et_3N (0.18mL, 1.25mmol), and HATU (0.13g, 0.35mmol) were added into 3mL DMF. The mixture was stirred at room temperature overnight, and purified by flash chromatography (hexanes/EtOAc/MeOH) to afford desired product (0.03g, 25%). LC/MS (ESI) *m/z* 473.88 (M+H−*t*-butyl); $[M+H]^+$ calcd for $C_{30}H_{32}N_3O_4S^+$: 530.21

Step 5: tert-butyl (5-(4-(benzylcarbamoyl)phenyl)thiazol-2-yl)(4-methoxybenzyl)carbamate (0.03g, 0.06mmol) were dissolved in DCM (2-3mL) and treated with TFA (2-3mL). The mixtures were stirred at room temperature until the tert-butyloxycarbonyl protecting group was cleaved tracking by UPLC-MS. The mixture was concentrated and flushed by flash column chromatography (EtOAc/MeOH/0.5%Et_3N). LC/MS (ESI) *m/z* 309.87; $[M+H]^+$ calcd for $C_{17}H_{16}N_3OS^+$: 310.10

Step 6: 4-(2-aminothiazol-5-yl)-N-benzylbenzamide (0.028g, 0.09mmol), $CuBr_2$ (0.025g, 0.18mmol), and t-butyl nitrite (0.02g, 0.18mmol) were added into 2mL anhydrous MeCN at 0°C. The mixture was then warmed up to room temperature then 65°C, and stirred for 4 hours. The reaction was cooled to room temperature, and diluted with water (30mL). The mixture was acidified with HBr (48%wt in H_2O) to pH=2 and extracted with EtOAc (30mL×2). The combined organic layer was washed with brine, dried over anhydrous Na_2SO_4, filtered, and concentrated under reduced pressure to afford the crude material. The material was purified by flash chromatography (hexanes/EtOAc/MeOH) followed by preparative HPLC (MeOH or CH_3CN/H_2O with 0.0425% TFA) to afford the target products to afford desired product (6mg, 18%).

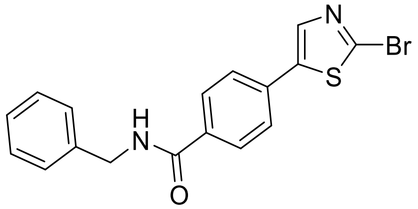

XL_9872_159 ^1H NMR (500 MHz, DMSO) δ 9.14 (t, *J* = 5.9 Hz, 1H), 8.23 (s, 1H), 7.98 (d, *J* = 8.4 Hz, 2H), 7.78 (d, *J* = 8.4 Hz, 2H), 7.34 (dd, *J* = 7.7, 5.6 Hz, 4H), 7.29 – 7.22 (m, 1H), 4.50 (d, *J* = 6.0 Hz, 2H). LC/MS (ESI) *m/z* 372.67; $[M+H]^+$ calcd for $C_{17}H_{14}BrN_2O_4S^+$: 373.00

Synthesis of XL_9872_163:

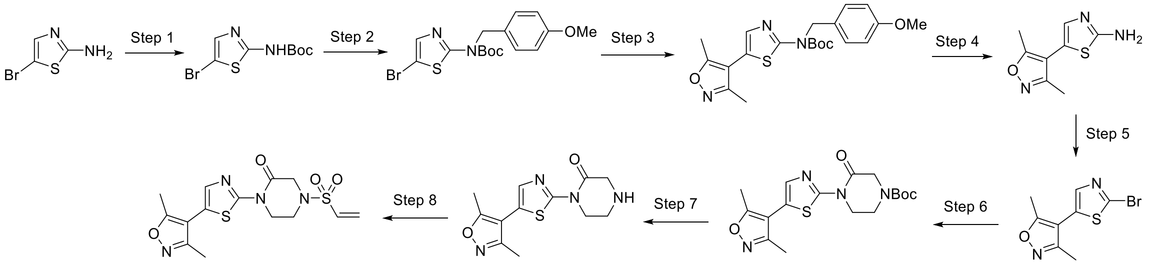

Step 1: 2-amino-5-bromothiazole hydrobromide (1.3g, 5.0mmol), di-tert-butyl decarbonate (1.3g, 6.0mmol), Et_3N (1.4mL, 10.0mmol), and DMAP (0.06g, 0.5mmol) were added into 5mL THF sequentially. The mixture was stirred at room temperature overnight, and diluted with EtOAc (30mL). The solution was washed with saturated sodium bicarbonate (30mL×2). Combined aqueous layers was extracted with EtOAc (50mL). Combined organic layer was washed with brine, dried over anhydrous Na_2SO_4, filtered, and concentrated under reduced pressure to afford crude material. The crude was purified by flash chromatography (hexanes/EtOAc) to afford desired product. LC/MS (ESI) *m/z* 222.88 (M+H−*t*-butyl); $[M+H]^+$ calcd for $C_8H_{12}BrN_2O_2S^+$: 278.98

Step 2: tert-Butyl (5-bromothiazol-2-yl)carbamate (0.57g, 2.1mmol), PPh_3 (1.18g, 4.5mmol), and p-methoxybenzyl alcohol (0.57g, 4.1mmol) were mixed up in 10mL anhydrous THF. The mixture was stirred at 0°C while DIAD (0.91g, 4.5mmol) was added dropwisely. Upon completion, the mixture was warmed to room temperature, and stirred for 2 hours. The resulting reaction mixture was diluted with EtOAc (30mL). The solution was washed with saturated sodium bicarbonate (30mL×2). Combined aqueous layers was extracted with EtOAc (50mL). Combined organic layer was washed with brine, dried over anhydrous Na_2SO_4, filtered, and concentrated under reduced pressure to afford crude material. The crude was purified by flash chromatography (hexanes/EtOAc) to afford desired product (0.60g, 74%). LC/MS (ESI) *m/z* 342.24 (M+H−*t*-butyl); $[M+H]^+$ calcd for $C_{16}H_{20}BrN_2O_3S^+$: 399.04

Step 3: tert-butyl (5-bromothiazol-2-yl)(4-methoxybenzyl)carbamate (0.2g, 0.5mmol), (3,5-dimethylisoxazol-4-yl)boronic acid (0.14g, 1.0mmol), potassium carbonate (0.14g, 1.0mmol) and $Pd(dppf)Cl_2$ (0.04g, 0.05mmol) were added into 1,4-dioxane/H_2O (3mL/0.5mL). The mixture was purged with N_2 for 10min before stirring at 95°C overnight under N_2. Then the mixture was concentrated under reduced pressure, diluted with EtOAc (30mL), and washed with saturated NH_4Cl (30mL×2). Combined aqueous layer was extracted with EtOAc (50mL). Combined organic layer was washed once with brine, dried over anhydrous Na_2SO_4, filtered, and evaporated under reduced pressure to afford crude material, which was then purified by flash chromatography (hexanes/EtOAc/MeOH) to afford desired product (0.19g, 91%). LC/MS (ESI) *m/z* 359.77 (M+H−*t*-butyl); $[M+H]^+$ calcd for $C_{21}H_{26}N_3O_4S^+$: 416.16

Step 4: tert-butyl (5-(3,5-dimethylisoxazol-4-yl)thiazol-2-yl)(4-methoxybenzyl)carbamate (0.19g, 0.45mmol) was dissolved in 3mL DCM. Into the solution was added 3mL TFA. The mixture was stirred at 80°C for 5 hours, then concentrated under reduced pressure, and re-dissolved in DCM (50mL). The organic solution was basified using saturated $NaHCO_3$ (50mL×2). Combined aqueous layer was extracted with DCM (50mL). Combined organic layer was washed with brine, dried over anhydrous Na_2SO_4, filtered, and concentrated under reduced pressure to afford crude material (0.13g, quant.) which was used directly in the following step without further purification. LC/MS (ESI) *m/z* 195.98; $[M+H]^+$ calcd for $C_8H_{10}N_3OS^+$: 196.05

Step 5: *Step 6*: 4-(2-aminothiazol-5-yl)-N-benzylbenzamide (0.078g, 0.4mmol), $CuBr_2$ (0.115g, 0.8mmol), and t-butyl nitrite (0.082g, 0.8mmol) were added into 3mL anhydrous MeCN at 0°C. The mixture was then warmed up to room temperature then 65°C, and stirred for 4 hours. The reaction was cooled to room temperature, and diluted with water (30mL). The mixture was acidified with HBr (48%wt in H_2O) to pH=2 and extracted with EtOAc (30mL×2). The combined organic layer was washed with brine, dried over anhydrous Na_2SO_4, filtered, and concentrated under reduced pressure to afford the crude material. The material was purified by flash chromatography (hexanes/EtOAc) to afford the target product (0.076g, 75%). LC/MS (ESI) *m/z* 258.77; $[M+H]^+$ calcd for $C_8H_8BrN_2OS^+$: 258.95

Step 6: 4-(2-bromothiazol-5-yl)-3,5-dimethylisoxazole (0.076g, 0.3mmol), 1-Boc-3-oxopiperazine (0.12g, 0.6mmol), cesium carbonate (0.39g, 1.2mmol), $Pd_2(dba)_3$ (0.028g, 0.03mmol), and Xantphos (0.035g, 0.06mmol) were added into 4mL 1,4-dioxane. The mixture was degassed by bubbling in N_2 for 10-15min before heated at 95°C overnight. Then the mixture was cooled to room temperature before diluted with EtOAc (30mL). Organic layer was washed with 20% citric acid (20mL×2). Combined aqueous layer was extracted with EtOAc (30mL). Combined organic layer was washed with brine, dried over anhydrous Na_2SO_4, filtered and concentrated under reduced pressure to afford crude material. The crude material was purified by flash chromatography (hexanes/EtOAc/MeOH) to afford desired product (0.035g, 31%). LC/MS (ESI) *m/z* 378.87; $[M+H]^+$ calcd for $C_{17}H_{23}N_4O_4S^+$: 379.14

Step 7: tert-butyl 4-(5-(3,5-dimethylisoxazol-4-yl)thiazol-2-yl)-3-oxopiperazine-1-carboxylate (0.035g, 0.09mmol) were dissolved in DCM (1mL) and treated with 4M HCl in 1,4-dioxane (1mL).

The mixtures were stirred at room temperature until the tert-butyloxycarbonyl protecting group was cleaved tracking by UPLC-MS. The mixture was concentrated and flushed by flash column chromatography (EtOAc/MeOH/0.5%Et_3N) to afford desired product (0.025g, 97%).

Step 8: 1-(5-(3,5-dimethylisoxazol-4-yl)thiazol-2-yl)piperazin-2-one (0.025g, 0.087mmol), Et_3N (0.1mL, 0.7mmol) were added in to 2mL DCM at 0°C. Into the solution was added 2-chloroethane sulfonyl chloride (0.023g, 0.14mmol). The mixture was stirred at 0°C for 1 hour before purified by flash chromatography (hexanes/EtOAc/MeOH) followed by preparative HPLC (MeOH or CH_3CN/H_2O with 0.0425% TFA) to afford the target products to afford desired product (7mg, 22%).

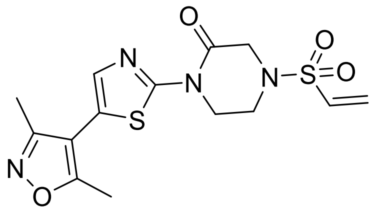

XL_9872_163 ^{1}H NMR (500 MHz, DMSO) δ 7.69 (s, 1H), 6.99 (dd, *J* = 16.5, 10.0 Hz, 1H), 6.41 – 6.12 (m, 2H), 4.28 – 4.18 (m, 2H), 4.12 (s, 2H), 3.68 – 3.59 (m, 2H), 2.48 (s, 3H), 2.29 (s, 3H). LC/MS (ESI) *m/z* 368.87; $[M+H]^+$ calcd for $C_{14}H_{17}N_4O_4S_2^+$: 369.07

<u>Synthesis of XL_11478_092A, 092B, 092C, 092D, 092E, 093A, 093B, 093C, 093D, 093E, 093F</u>:

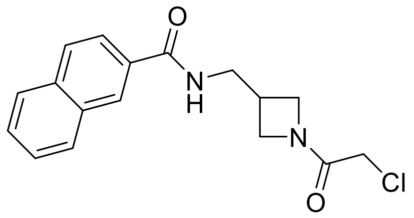

Synthesis of XL_11478_092A followed the General Procedure 1 by using 2-naphthoic acid (0.10g, 0.6mmol) and t-butyl-3-(aminoethyl)acetidine-1-carboxylate (0.09g, 0.5mmol) to afford XL_11478_092A (0.027g, 17%) ^{1}H NMR (500 MHz, DMSO) δ 8.82 (t, *J* = 5.6 Hz, 1H), 8.44 (s, 1H), 8.01 (m,3H), 7.93 (dd, *J* = 8.6, 1.6 Hz, 1H), 7.68 – 7.54 (m, 2H), 4.28 (t, *J* = 8.6 Hz, 1H), 4.16 – 4.05 (s, 2H), 4.00 (dd, *J* = 16.6, 7.8 Hz, 2H), 3.74 (dd, *J* = 9.8, 5.4 Hz, 1H), 3.59 – 3.49 (m, 2H), 3.00 – 2.80 (m, 1H). ^{13}C NMR (126 MHz, DMSO) δ 167.31, 166.18, 134.62, 132.60, 132.19, 129.28,

128.36, 128.09, 128.07, 127.93, 127.22, 124.64, 53.93, 51.94, 42.67, 40.44, 29.04. LC/MS (ESI) *m/z* 316.67; $[M+H]^+$ calcd for $C_{17}H_{18}ClN_2O_2^+$: 317.11

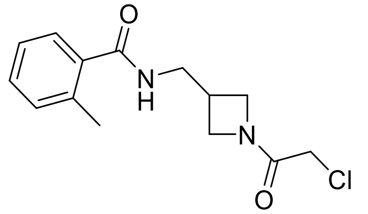

Synthesis of XL_11478_092B followed the General Procedure 1 by using 2-methylbenzoic acid (0.08g, 0.6mmol) and t-butyl-3-(aminoethyl)acetidine-1-carboxylate (0.09g, 0.5mmol) to afford XL_11478_092B (0.042g, 30%) ^{1}H NMR (500 MHz, DMSO) δ 8.44 (m, 1H), 7.41 – 7.28 (m, 2H), 7.28 – 7.18 (m, 2H), 4.32 – 4.21 (m, 1H), 4.15 – 4.05 (m, 2H), 4.02 – 3.91 (m, 2H), 3.76 – 3.63 (m, 1H), 3.44 (m, 2H), 2.91 – 2.77 (m, 1H), 2.33 (s, 3H). LC/MS (ESI) *m/z* 280.77; $[M+H]^+$ calcd for $C_{14}H_{18}ClN_2O_2^+$: 281.11

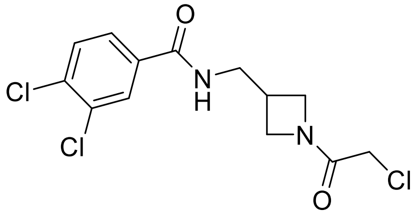

Synthesis of XL_11478_092C followed the General Procedure 1 by using 3,4-dichlorobenzoic acid (0.12g, 0.6mmol) and t-butyl-3-(aminoethyl)acetidine-1-carboxylate (0.09g, 0.5mmol) to afford XL_11478_092C (0.058g, 35%) ^{1}H NMR (500 MHz, DMSO) δ 8.80 (t, *J* = 5.6 Hz, 1H), 8.07 (d, *J* = 2.0 Hz, 1H), 7.82 (dd, *J* = 8.4, 2.0 Hz, 1H), 7.80 – 7.69 (m, 1H), 4.32 – 4.20 (m, 1H), 4.16 – 4.04 (s, 2H), 3.99 – 3.89 (m, 2H), 3.68 (dd, *J* = 9.9, 5.4 Hz, 1H), 3.49 (dd, *J* = 6.8, 5.9 Hz, 2H), 2.91 – 2.78 (m, 1H). LC/MS (ESI) *m/z* 334.87; $[M+H]^+$ calcd for $C_{13}H_{14}Cl_3N_2O_2^+$: 335.01

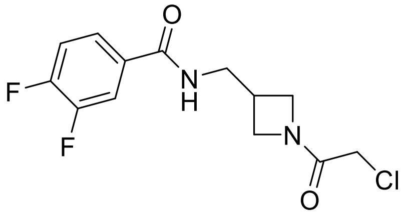

Synthesis of XL_11478_092D followed the General Procedure 1 by using 3,4-difluorobenzoic acid (0.095g, 0.6mmol) and t-butyl-3-(aminoethyl)acetidine-1-carboxylate (0.09g, 0.5mmol) to afford XL_11478_092D (0.037g, 25%) ^{1}H NMR (500 MHz, DMSO) δ 8.77 (m, 1H), 8.00 – 7.85 (m, 1H), 7.80 – 7.68 (m, 1H), 7.61 – 7.49 (m, 1H), 4.29 – 4.22 (m, 1H), 4.14 – 4.04 (m, 2H), 3.99 – 3.91 (m, 2H), 3.73 – 3.64 (m, 1H), 3.48 (dd, *J* = 11.7, 5.4 Hz, 2H), 2.92 – 2.77 (m, 1H). LC/MS (ESI) *m/z* 302.97; $[M+H]^+$ calcd for $C_{13}H_{14}ClF_2N_2O_2^+$: 303.07

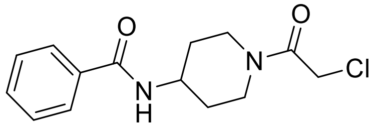

Synthesis of XL_11478_093A followed the General Procedure 1 by using benzoic acid (0.07g, 0.6mmol), t-butyl-4-amino piperidine-1-carboxylate (0.10g, 0.5mmol) to afford XL_11478_093A (0.07g, 52%) ^{1}H NMR (500 MHz, DMSO) δ 8.32 (d, *J* = 7.7 Hz, 1H), 7.85 (d, *J* = 7.4 Hz, 2H), 7.52 (t, *J* = 7.2 Hz, 1H), 7.46 (t, *J* = 7.5 Hz, 2H), 4.44 (d, *J* = 12.8 Hz, 1H), 4.35 (d, *J* = 12.9 Hz, 1H), 4.30 (d, *J* = 13.2 Hz, 1H), 4.14 – 4.00 (m, 1H), 3.87 (d, *J* = 13.6 Hz, 1H), 3.19 (t, *J* = 11.8 Hz, 1H), 2.81 (t, *J* = 11.5 Hz, 1H), 1.96 – 1.74 (m, 2H), 1.57 (qd, *J* = 12.2, 4.0 Hz, 1H), 1.42 (qd, *J* = 12.3, 4.2 Hz, 1H). ^{13}C NMR (126 MHz, DMSO) δ 166.25, 164.92, 135.05, 131.56, 128.63, 127.77, 46.83, 45.00, 42.38, 41.32, 32.24, 31.45. LC/MS (ESI) *m/z* 280.77; $[M+H]^+$ calcd for $C_{14}H_{18}ClN_2O_2^+$: 281.11

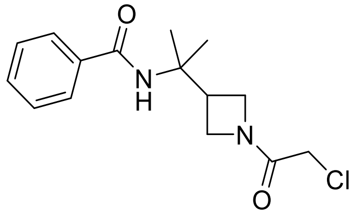

Synthesis of XL_11478_093B followed the General Procedure 1 by using benzoic acid (0.07g, 0.6mmol), t-butyl-3-(2-aminopropan-2-yl)azetidine-1-carboxylate (0.10g, 0.5mmol) to afford XL_11478_093B (5mg, 3.4%) LC/MS (ESI) *m/z* 295.18; $[M+H]^+$ calcd for $C_{15}H_{20}ClN_2O_2^+$: 295.12

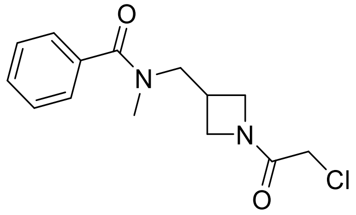

Synthesis of XL_11478_093C followed the General Procedure 1 by using benzoic acid (0.07g, 0.6mmol), t-butyl-ethylaminoazetidine-1-carboxylate (0.10g, 0.5mmol) to afford XL_11478_093C (0.032g, 23%) 1H NMR (500 MHz, CDCl3) δ 7.49 – 7.35 (m, 5H), 4.42 (br, 1H), 4.27 (br, 2H), 4.08 (br, 1H), 3.91 (br, 3H), 3.60 (dd, J = 13.5, 6.6 Hz, 1H), 3.02 (s, 4H). LC/MS (ESI) m/z 281.08; [M+H]+ calcd for C14H18ClN2O2+: 281.11

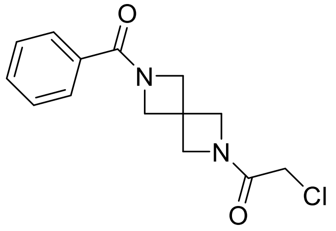

Synthesis of XL_11478_093D followed the General Procedure 1 by using benzoic acid (0.07g, 0.6mmol), t-butyl-2,6-diazaspiro[3,3]heptane-2-carboxylate (0.10g, 0.5mmol) to afford XL_11478_093D (0.054g, 39%) ^{1}H NMR (500 MHz, DMSO) δ 7.67 – 7.56 (m, 2H), 7.50 (m, 3H), 4.46 (br, 2H), 4.39 – 4.29 (m, 2H), 4.18 (m, 2H), 4.15 – 4.00 (m, 4H). LC/MS (ESI) *m/z* 278.67; $[M+H]^+$ calcd for $C_{14}H_{16}ClN_2O_2^+$: 279.09

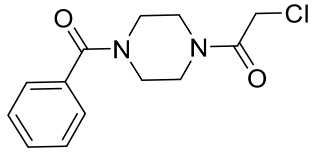

Synthesis of XL_11478_093E followed the General Procedure 1 by using benzoic acid (0.07g, 0.6mmol), t-butylpiperazine-1-carboxylate (0.093g, 0.5mmol) to afford XL_11478_093E (0.11g, 83%) ^{1}H NMR (500 MHz, DMSO) δ 7.51 – 7.45 (m, 3H), 7.45 – 7.40 (m, 2H), 4.37 (s, 2H), 3.54 (br, 8H). LC/MS (ESI) *m/z* 266.77; $[M+H]^+$ calcd for $C_{13}H_{16}ClN_2O_2^+$: 267.09

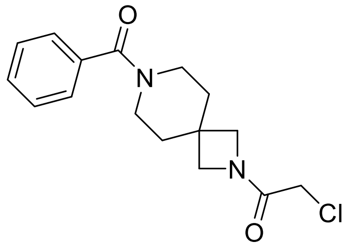

Synthesis of XL_11478_093F followed the General Procedure 1 by using benzoic acid (0.07g, 0.6mmol), t-butyl-2,7-diazaspiro[3,5]nonane-2-carboxylate (0.11g, 0.5mmol) to afford

XL_11478_093F (0.053g, 35%) 1H NMR (500 MHz, CDCl3) δ 7.51 – 7.35 (m, 5H), 4.05 (br, 2H), 3.93 (s, 2H), 3.86 (br, 2H), 3.68 (br, 1H), 3.41 (br, 2H), 1.85 (br, 4H). LC/MS (ESI) m/z 307.08; [M+H]+ calcd for $C_{16}H_{20}ClN_2O_2^+$: 307.12.

Synthesis of XL_10320_012A, 012B:

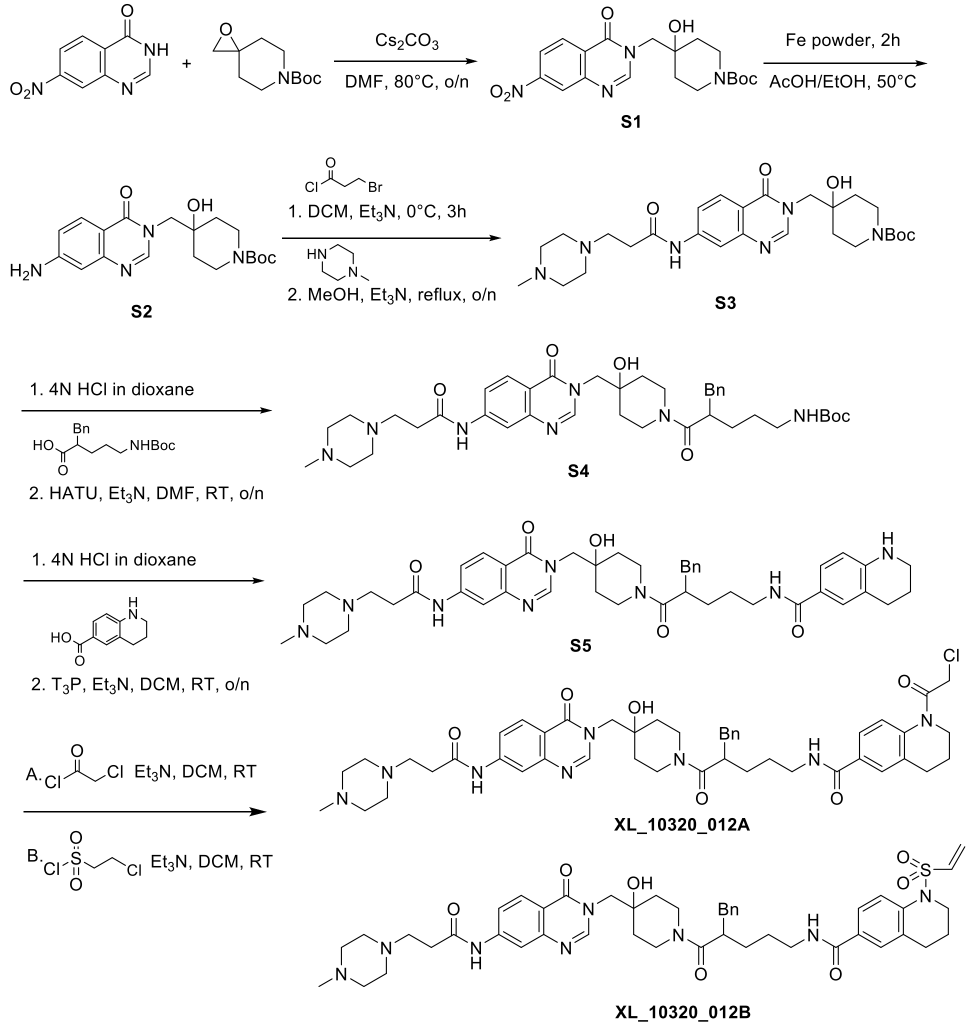

Step 1 (Synthesis of **S1**): 7-nitroquinazolin-4(*3H*)-one (1.55g, 8.1mmol) and *tert*-butyl-1-oxa-6-azaspiro[2.5]octane-6-carboxylate (1.90g, 8.9mmol) were added into 20mL DMF. Cesium carbonate (7.82g, 24.0mmol) was added in one portion. The mixture was heated at 80°C overnight. The mixture was diluted with EtOAc, then washed with sat. NaCl. Combined organic layer was concentrated under reduced pressure. The crude product was purified by flash chromatography (EtOAc: hexanes: 50%-70%) to afford 2.42g **S1** (75%). ^{1}H NMR (500 MHz, $CDCl_3$) δ 8.54 (d, *J* = 2.1 Hz, 1H), 8.44 (t, *J* = 8.6 Hz, 1H), 8.26 (dd, *J* = 8.8, 2.2 Hz, 1H), 8.20 (s, 1H), 4.10 (s,

2H), 3.88 (s, 2H), 3.14 (t, *J* = 11.6 Hz, 2H), 1.73 – 1.48 (m, 5H), 1.44 (s, 9H). LCMS (ESI) *m/z* 304.97 (M + H - Boc) [$(M+H)^+$ $C_{19}H_{25}N_4O_6^+$ calcd for 405.18]

Step 2 (Synthesis of **S2**): Compound **S1**(2.4g, 6.0mmol) was suspended in 20mL solvent (EtOH/AcOH=1:1). 4 eq. of Fe powder was added in portions. The mixture was stirred for 1 hour at 55°C. Then the reaction was cooled down to room temperature, and filtered through a pad of Celite. The filtrate was concentrated under reduced pressure to afford the crude product, which was then purified by flash chromatography (10%MeOH in EtOAc) to afford 2.1g product **S2** (93%) ^{1}H NMR (500 MHz, DMSO) δ 8.04 (s, 1H), 7.79 (d, *J* = 8.7 Hz, 1H), 6.72 (dd, *J* = 8.7, 1.9 Hz, 1H), 6.61 (d, *J* = 2.0 Hz, 1H), 6.09 (s, 2H), 4.87 (s, 1H), 3.89 (s, 2H), 3.64 (d, *J* = 12.0 Hz, 2H), 3.05 (s, 2H), 1.54 – 1.24 (m, 13H).LCMS (ESI) *m/z* 374.97 [$(M+H)^+$ $C_{19}H_{27}N_4O_4^+$ calcd for 375.20]

Step 3 (Synthesis of **S3**): Compound **S2** (2.1g, 5.6mmol) was dissolved in anhydrous 10mL dichloromethane under N_2 at 0°C. 3.0 eq. of Et_3N was added. Then 3-bromopropionyl chloride (1.15g, 6.7mmol) was added dropwisely. The mixture was stirred at 0°C for 1 hour, then quenched with MeOH, and concentrated under reduced pressure. The solid residue was directly used for the following step without further purification. The crude product from last step was dissolved in 10mL MeOH, then 3.0eq of Et_3N was added. Into the stirred mixture was added 1-methylpiperazine (0.67g, 6.7mmol) dropwisely. After the addition completed, the mixture was stirred for 1 hour at 50°C. Then the reaction mixture was cooled down to room temperature and concentrated under reduced pressure, then directly subjected to HPLC purification (MeOH/H_2O with 4‰ TFA) to afford 2.1g product **S3** (73% in two steps) ^{1}H NMR (500 MHz, CD_3OD) δ 8.28 (s, 1H), 8.20 (d, *J* = 8.8 Hz, 1H), 8.12 (d, *J* = 1.9 Hz, 1H), 7.69 (dd, *J* = 8.7, 2.0 Hz, 1H), 4.11 (s, 2H), 3.82 (d, *J* = 13.4 Hz, 2H), 3.23 (m, 2H), 2.85 (t, *J* = 7.0 Hz, 2H), 2.79 – 2.50 (m, 10H), 2.37 (s, 3H), 1.72 – 1.62 (m, 2H), 1.50 (d, *J* = 17.4 Hz, 11H). LCMS (ESI) *m/z* 529.08 [$(M+H)^+$ $C_{27}H_{41}N_6O_5^+$ calcd for 529.31].

Step 4 (Synthesis of **S4**): **S3** (0.53g, 1.0mmol) was dissolved in 3mL DCM, then 5mL 4M HCl in 1,4-dioxane was added in portions. The solution was stirred for 1 hour at room temperature. Then the mixture was concentrated under reduced pressure, and left on high vacuum overnight to remove residual acid. Then the product (0.11g, 0.25mmol) was dissolved in 3mL DMF, and basified by adding 10 eq of Et_3N. Into the solution 2-benzyl-5-((tert-

butoxycarbonyl)amino)pentanoic acid (0.11g, 0.35mmol) and HATU (0.16g, 0.4mmol) sequentially The resultant solution was stirred overnight. Then the mixture was directly subjected to HPLC purification (MeOH/H_2O with 4‰ TFA) to afford 183mg **S4** (quantitative) ^{1}H NMR (500 MHz, DMSO) δ 10.51 (s, 1H), 8.16-8.04 (m, 2H, conformer), 8.01 (dd, *J* = 5.0, 1.9 Hz, 1H), 7.61 (ddd, *J* = 9.8, 8.3, 2.0 Hz, 1H), 7.29 – 7.19 (m, 2H), 7.19 – 7.07 (m, 3H), 6.76 (m, 1H), 4.84 (s, 1H), 4.11 (m, 1H), 4.05 – 3.87 (m, 2H), 3.80 (d, *J* = 13.7 Hz, 1H), 3.67 – 3.49 (m, 3H), 3.16 – 3.03 (m, 2H), 2.98 (s, 1H), 2.85 (m, 3H), 2.77 – 2.58 (m, 5H), 2.55 (m, 3H), 2.22 (s, 3H), 1.61 – 1.44 (m, 1H), 1.43 – 1.33 (m, 9H, conformer), 1.33 – 1.22 (m, 3H), 1.22 – 1.02 (m, 4H), 0.39 (m, 1H). LCMS (ESI) *m/z* 718.00 [(M+H)$^+$ 718.43 calcd for $C_{39}H_{56}N_7O_6^+$]

Step 5 (Synthesis of S5): **S4** (0.15g, 0.2mmol) was dissolved in 2mL DCM, then 2mL 4M HCl in 1,4-dioxane was added in portions. The solution was stirred for 1 hour at room temperature. Then the mixture was concentrated under reduced pressure, and left on high vacuum overnight to remove residual acid. Then the product (0.12g, 0.2mmol) was dissolved in 3mL DMF, and basified by adding 10 eq of Et_3N. Into the solution 1,2,3,4-tetrahydroquinoline carboxylic acid (0.064g, 0.3mmol) and HATU (0.12g, 0.3mmol) sequentially. The resultant solution was stirred overnight. Then the mixture was directly subjected to HPLC purification (MeOH/H_2O with 4‰ TFA) to afford 108mg **S5** (56%). LCMS (ESI) *m/z* 776.91 [(M+H)$^+$ 777.44 calcd for $C_{44}H_{57}N_8O_5^+$]

Step 6 (Synthesis of XL_10320_012A, 012B): S5 (0.054g, 0.07mmol) was dissolved in anhydrous DCM/DMF co-solvent (2mL, v/v=1:1). Et_3N (0.05mL, 0.34mmol) was added into the solution, followed by addition of 2-chloroacetyl chloride (9.4mg, 0.08mmol) or 2-chloroethane-1-sulfonyl chloride (22mg, 0.14mmol). The mixture was stirred at 0°C for 1 hour before purified by flash chromatography (hexanes/EtOAc/MeOH) followed by preparative HPLC (MeOH or CH_3CN/H_2O with 0.0425% TFA) to afford the target products.

XL_10320_012A (31mg, 53%, mixture of rotamers) ^{1}H NMR (500 MHz, DMSO) δ 10.66 (s, 1H), 8.41 (dt, *J* = 10.9, 5.4 Hz, 1H), 8.22 – 8.00 (m, 3H), 7.66 (m, 4H), 7.30 – 7.17 (m, 2H), 7.17 – 7.05 (m, 3H), 4.57 (s, 2H), 4.12 (d, *J* = 12.4 Hz, 0.5H), 3.94 (m, 1H), 3.81 (d, *J* = 13.6 Hz, 0.5H), 3.71 (t, *J* = 6.2 Hz, 2H), 3.66 – 3.54 (m, 2H), 3.36 (m, 3H), 3.32 – 3.15 (m, 5.5H), 3.05 (m, 3H), 2.80 (m, 5.5H), 2.78 – 2.57 (m, 5.5H), 1.98 – 1.81 (m, 2H), 1.59 (m, 1H), 1.51 – 1.25 (m, 5H), 1.13 (m, 3H), 0.39 (m, 1H). LCMS (ESI) *m/z* 853.02 [(M+H)$^+$ 853.42 calcd for $C_{46}H_{58}ClN_8O_6^+$]

XL_10320_012B (34mg, 57%, mixture of rotamers) ^{1}H NMR (500 MHz, DMSO) δ 10.67 (s, 1H), 8.37 (dt, *J* = 11.0, 5.5 Hz, 1H), 8.24 – 8.00 (m, 3H), 7.59 (m, 4H), 7.21 (m, 2H), 7.16 – 7.04 (m, 3H), 6.90 (dd, *J* = 16.3, 9.9 Hz,

1H), 6.14 (d, *J* = 15.0 Hz, 1H), 6.10 (d, *J* = 10.0 Hz, 1H), 4.12 (d, *J* = 12.1 Hz, 0.5H), 4.05 – 3.86 (m, 1H), 3.81 (d, *J* = 13.6 Hz, 0.5H), 3.75 – 3.59 (m, 3.5H), 3.48 (m, 3.5H), 3.28 (m, 4H), 3.19 (m, 2.5H), 3.10 (m, 2.5H), 2.93 – 2.76 (m, 7H), 2.67 (m, 3H), 1.99 – 1.82 (m, 2H), 1.56 (m, 1H), 1.51 – 1.23 (m, 5H), 1.23 – 0.99 (m, 3H), 0.39 (t, *J* = 10.5 Hz, 1H). LCMS (ESI) *m/z* 866.82 [(M+H)$^+$ 867.42 calcd for $C_{46}H_{59}N_8O_7S^+$]

Synthesis of XL_10320_197:

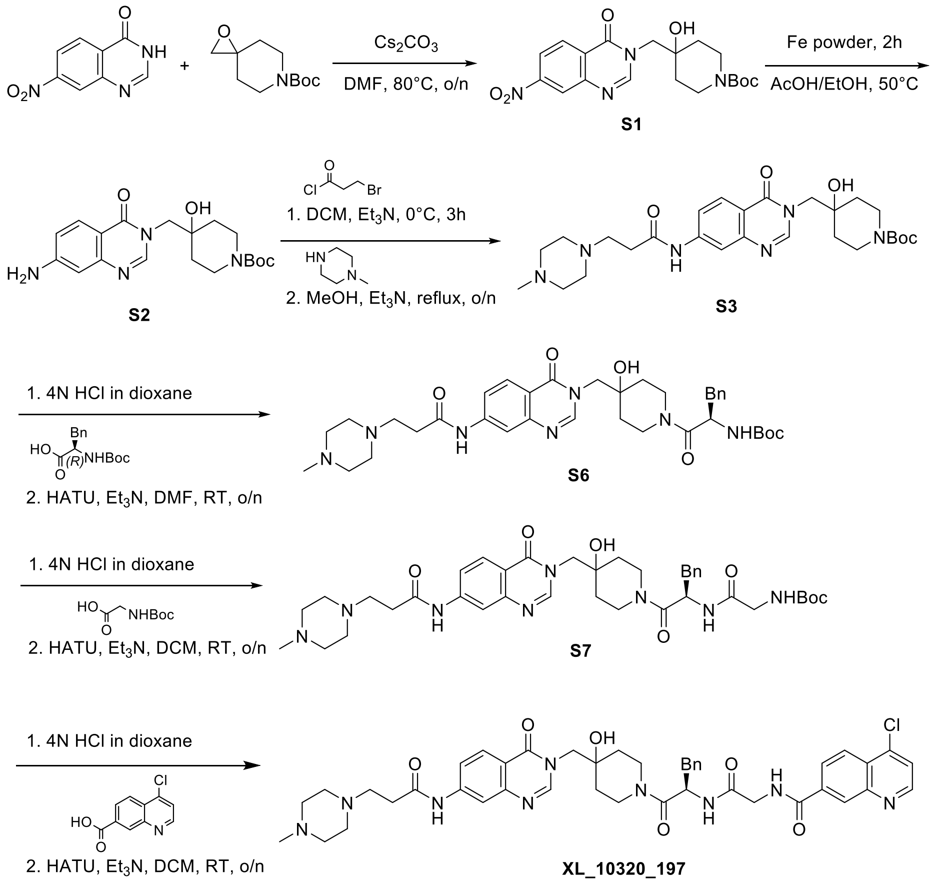

Step 1-3 (Synthesis of S1-S3): the same as the steps in synthetic routes toward XL_10320_012A, B.

Step 4: (Synthesis of **S6**): **S3** (0.20g, 0.4mmol) was dissolved in 2mL DCM, then 2mL 4M HCl in 1,4-dioxane was added in portions. The solution was stirred for 1 hour at room temperature. Then the mixture was concentrated under reduced pressure, and left on high vacuum overnight to remove residual acid. Then the product (0.16g, 0.4mmol) was dissolved in 5mL DMF, and basified by adding 10 eq of Et_3N. Into the solution (tert-butoxycarbonyl)-D-phenylalanine (0.15g, 0.56mmol) and HATU (0.29g, 0.76mmol) sequentially. The resultant solution was stirred

overnight. Then the mixture was directly subjected to HPLC purification (MeOH/H_2O with 4‰ TFA) to afford 110mg **S6** (43%). LCMS (ESI) *m/z* 676.00 [$(M+H)^+$ 676.38 calcd for $C_{36}H_{50}N_7O_6^+$]

Step 5: (Synthesis of **S7**): **S6** (0.11g, 0.16mmol) was dissolved in 2mL DCM, then 2mL 4M HCl in 1,4-dioxane was added in portions. The solution was stirred for 1 hour at room temperature. Then the mixture was concentrated under reduced pressure, and left on high vacuum overnight to remove residual acid. Then the product (0.092g, 0.16mmol) was dissolved in 3mL DMF, and basified by adding 10 eq of Et_3N. Into the solution *N*-Boc glycine (0.053g, 0.3mmol) and HATU (0.15g, 0.4mmol) sequentially. The resultant solution was stirred overnight. Then the mixture was directly subjected to HPLC purification (MeOH/H_2O with 4‰ TFA) to afford 87mg **S7** (63%). LCMS (ESI) *m/z* 732.81 [$(M+H)^+$ 733.40 calcd for $C_{38}H_{53}N_8O_7^+$]

Step 6 (Synthesis of XL_10320_197): **S7** (0.087g, 0.12mmol) was dissolved in 2mL DCM, then 2mL 4M HCl in 1,4-dioxane was added in portions. The solution was stirred for 1 hour at room temperature. Then the mixture was concentrated under reduced pressure, and left on high vacuum overnight to remove residual acid. Then the product (0.076g, 0.12mmol) was dissolved in 3mL anhydrous DCM, and basified by adding 10 eq of Et_3N. Into the solution 4-chloroquinoline-7-carboxylic acid (0.03g, 0.14mmol) and T3P (0.23g, 0.7mmol) sequentially. The resultant solution was stirred overnight. Then the mixture was directly subjected to flash chromatography (EtOAc/MeOH) and HPLC purification (MeOH/H_2O with 4‰ TFA) to afford 25mg **XL_10320_197** (25%). ^{1}H NMR (500 MHz, MeOD) δ 8.87 (dd, *J* = 4.7, 2.5 Hz, 1H), 8.60 (dd, *J* = 4.1, 1.6 Hz, 1H), 8.38 (dd, *J* = 8.7, 6.7 Hz, 1H), 8.27 – 8.14 (m, 3H), 8.14 – 8.03 (m, 1H), 7.78 (dd, *J* = 4.8, 2.4 Hz, 1H), 7.65 (ddd, *J* = 34.7, 8.7, 2.0 Hz, 1H), 7.36 (t, *J* = 7.6 Hz, 1H), 7.32 – 7.16 (m, 4H), 5.25 – 5.08 (m, 1H), 4.27 – 4.06 (m, 3H), 4.00 (dd, *J* = 19.8, 13.9 Hz, 1H), 3.74 (m, 2H), 3.15 – 2.91 (m, 4H), 2.86 – 2.77 (m, 2H), 2.74 – 2.40 (m, 7H), 2.32 (s, 3H), 1.83 – 1.55 (m, 2H), 1.54 – 1.21 (m, 3H), 0.71 – 0.49 (m, 1H). LCMS (ESI) *m/z* 821.69 [$(M+H)^+$ 822.35 calcd for $C_{43}H_{49}ClN_9O_6^+$]

Synthesis of AC-10180-19:

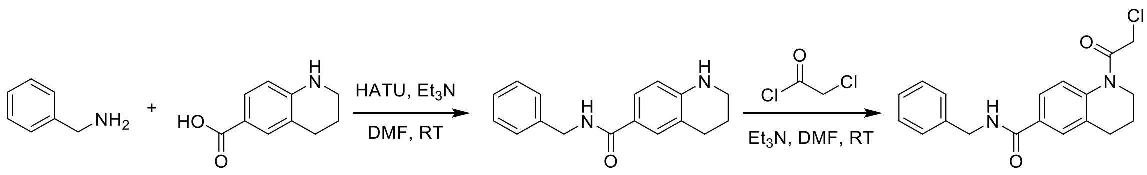

Synthesis of AC-10180-19 followed the General Procedure 1 by using benzylamine (0.29mL, 2.7mmol), 1,2,3,4-tetrahydroquinoline-6-carboxylic acid (0.52g, 2.9mmol), HATU (1.12g,

2.9mmol), and Et_3N (1.88mL, 13.4mmol). AC-10180-19 (53mg, 75%) 1H NMR (500 MHz, DMSO) δ 8.99 (t, J = 6.0 Hz, 1H), 7.76 (s, 1H), 7.71 (m, 2H), 7.36 – 7.29 (m, 4H), 7.26 – 7.22 (m, 1H), 4.59 (s, 2H), 4.48 (d, J = 6.0 Hz, 2H), 3.78 – 3.69 (t, J = 10.0 Hz, 2H), 2.78 (t, J = 10.0 Hz, 2H), 1.98 – 1.87 (m, 2H). LC/MS (ESI) m/z 342.79; $[M+H]^+$ calcd for $C_{19}H_{20}ClN_2O_2^+$: 343.12

Synthesis of AV-9606-12:

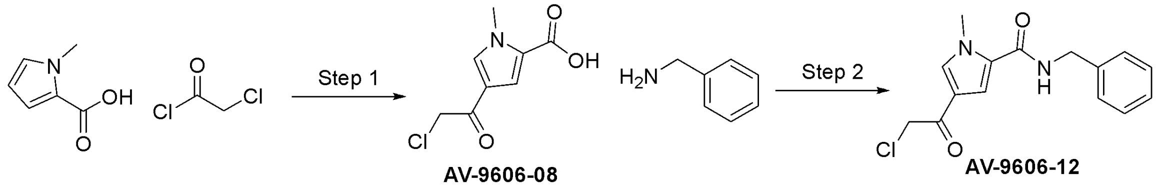

Step 1: 1-methyl-1H-pyrrole-2-carboxylic acid (125.1 mg, 1.0 mmol) was added to a heat dried pressure vial flushed with nitrogen and dissolved in DCM (5 mL). The vial was then placed in an ice bath and $AlCl_3$ (288.0 mg, 2.5 mmol) was then added as a solid to the reaction and the reaction was stirred for 30 minutes on ice. After 30 minutes, chloroacetyl chloride (87.6 µL, 1.1 mmol) was then added and the reaction was heated to 45°C for 18 hours. The reaction was quenched with saturated sodium bicarbonate solution until pH was greater than 7. The reaction was washed with DCM. The aqueous layer was then acidified with concentrated hydrochloric acid until pH 0-2 at which point the desired product as a white precipitate crashed out. The reaction was filtered to isolate the desired product (126.7 mg, 63% yield). ^1H NMR (500 MHz, DMSO-d_6) δ 7.90 (d, J = 2.0 Hz, 1H), 7.24 (d, J = 2.0 Hz, 1H), 4.81 (s, 2H), 3.89 (s, 3H). LC/MS (ESI) *m/z* 202.08 [M+H]+; calcd for $C_8H_9ClNO_3^+$: 202.03.

Step 2: AV-9606-08 (60.4 mg, 0.30 mmol) and HATU (137.2 mg, 0.36 mmol) were combined and suspended in THF (2 mL). Et_3N (83.5 µL, 0.6 mmol) was then added and the reaction was stirred under nitrogen. To the reaction was then added a solution of benzylamine (39.2 µL, 0.36 mmol) in THF (1 mL). The reaction was stirred at room temperature for 2 hours. The reaction was diluted with EtOAc and washed with water, saturated sodium bicarbonate, and brine. The organics were collected, dried over $MgSO_4$, filtered, and concentrated under reduced pressure. The crude material was purified flash chromatography (DCM/MeOH) to afford the desired product (56.6 mg, 65% yield). ^1H NMR (500 MHz, DMSO-d6) δ 8.84 (t, J = 6.1 Hz, 1H), 7.86 (d, J = 1.9 Hz, 1H), 7.36 –

7.28 (m, 5H), 7.27 – 7.21 (m, 1H), 4.76 (s, 2H), 4.40 (d, J = 6.0 Hz, 2H), 3.89 (s, 3H). LC/MS (ESI) *m/z* 291.18 [M+H]+; calcd for $C_{15}H_{16}ClN_2O_2^+$: 291.09.

<u>Synthesis of AV-9606-16:</u>

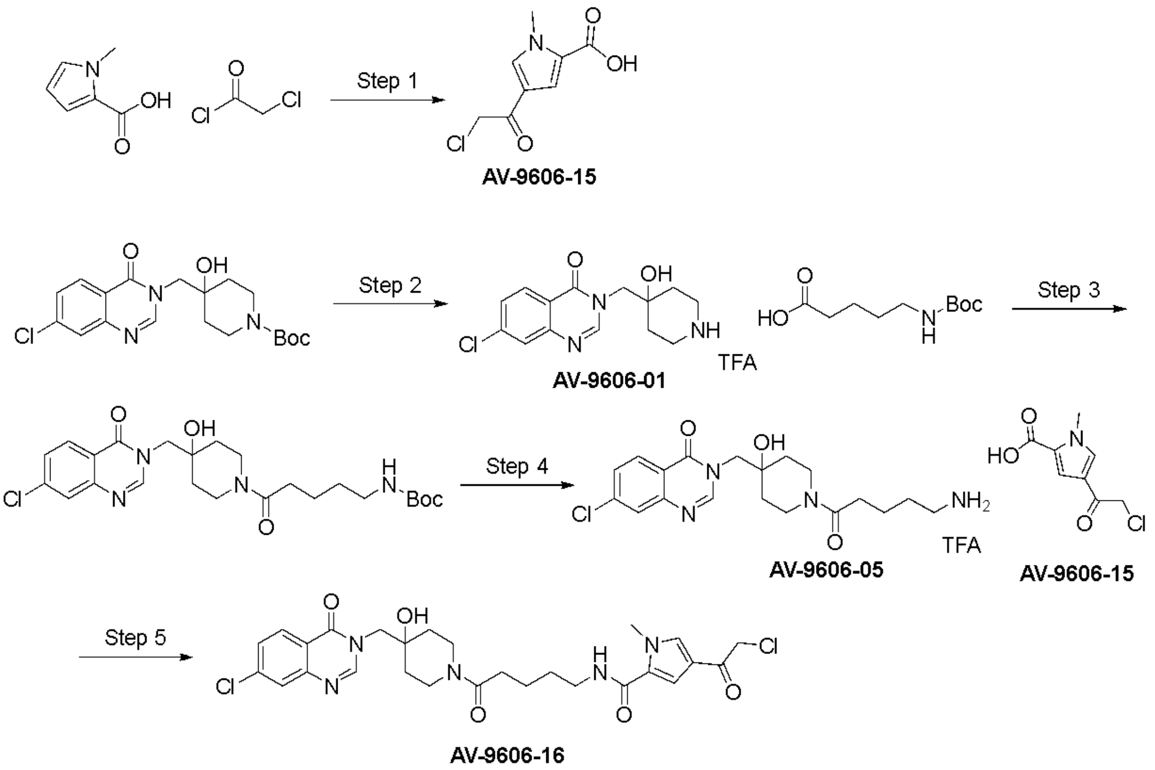

Step 1: 1-methyl-1H-pyrrole-2-carboxylic acid (376.7 mg, 3.0 mmol) was dissolved in DCM (15 mL) and the reaction was placed in an ice bath. To the reaction was then added $AlCl_3$ (822.6 mg, 6.2 mmol) and the reaction was stirred for 30 minutes on ice. After 30 minutes, chloroacetyl chloride (263.0 µL, 3.3 mmol) was then added and the reaction was heated to 45°C for 22 hours. The reaction was quenched with saturated sodium bicarbonate solution until pH was greater than 7. The reaction was washed with DCM. The aqueous layer was then acidified with concentrated hydrochloric acid until pH 0-2 at which point the desired product as a white precipitate crashed out. Filter to isolate the desired product (371.5 mg, 61% yield). LC/MS (ESI) *m/z* 202.18 [M+H]+; calcd for $C_8H_9ClNO_3^+$: 202.03.

Step 2: tert-butyl 4-((7-chloro-4-oxoquinazolin-3(4H)-yl)methyl)-4-hydroxypiperidine-1-carboxylate (1004.3 mg, 2.55 mmol) was suspended in TFA. The reaction was stirred under nitrogen gas at room temperature for 2 hours. The reaction was concentrated under reduced

pressure and used in the subsequent step without further purification. LC/MS (ESI) *m/z* 294.08 [M+H]+; calcd for $C_{14}H_{17}ClN_3O_2^+$: 294.10.

Step 3: 5-(Boc-amino)pentanoic acid (223.7 mg, 1.03 mmol) and HATU (653.2 mg, 2.0 mmol) were combined and suspended in DMF (1 mL). Et_3N (829.2 uL, 5.95 mmol) was added and the reaction was stirred at room temperature for 10 minutes. A solution of AV-9606-01 (249.7 mg, 0.85 mmol) in DMF (1 mL) was then added to the reaction. The reaction was stirred at room temperature under nitrogen for 3 hours. The reaction was diluted with EtOAc and washed with water and brine. The organics were combined, dried over Na_2SO_4, filtered, and concentrated under reduced pressure. The crude material was purified via flash chromatography (hexanes/EtOAc/MeOH) to afford the desired product (370 mg, 88%). LC/MS (ESI) *m/z* 493.20 [M+H]+; calcd for $C_{24}H_{34}ClN_4O_5^+$: 493.22.

Step 4: AV-9606-02 (370.0 mg, 0.75 mmol) was dissolved in TFA (3 mL). The reaction was stirred at room temperature for 2 hours. The reaction was concentrated under reduced pressure and the crude material was purified via flash chromatography (EtOAc/MeOH/20%NH_4OH) to afford the desired product (261.3 mg, 89%). LC/MS (ESI) *m/z* 393.29 $[M+H]^+$; calcd for $C_{19}H_{26}ClN_4O_3^+$: 393.17.

Step 5: AV-9606-15 (65.2 mg, 0.32 mmol) was suspended in DCM (1 mL). Thionyl chloride (1 mL) was added and the reaction was stirred at room temperature for 1 hour. The reaction was concentrated under reduced pressure. The crude material was suspended in DCM (1 mL) and the reaction was placed in an ice bath. To the reaction was then added Et_3N (198.7 µL, 1.43 mmol) followed by a solution of AV-9606-05 in DCM (1 mL). The reaction was warmed to room temperature and stirred for 1 hour. The reaction was quenched with water (0.5 mL). The crude material was purified via silica gel chromatography (DCM/MeOH) and fractions containing desired product were collected and concentrated under reduced pressure. The material was washed with hexanes, sonicated, and then filtered and dried under vacuum to afford the desired product (8.4 mg, 4.6%). ^1H NMR (500 MHz, Methanol-d_4) δ 8.81 (s, 1H), 8.35 – 8.23 (m, 1H), 7.76

(d, *J* = 2.0 Hz, 1H), 7.73 – 7.62 (m, 2H), 7.21 (d, *J* = 1.9 Hz, 1H), 4.62 (s, 2H), 4.25 – 4.10 (m, 3H), 3.78 (d, *J* = 13.8 Hz, 1H), 3.49 – 3.39 (m, 1H), 3.34 (d, *J* = 6.2 Hz, 2H), 3.21 (q, *J* = 7.3 Hz, 1H), 3.17 – 3.08 (m, 1H), 2.47 (q, *J* = 7.0 Hz, 2H), 2.01 (s, 8H), 1.75 – 1.61 (m, 6H), 1.61 – 1.51 (m, 2H), 1.35 – 1.27 (m, 2H). LC/MS (ESI) *m/z* 576.22 $[M+H]^+$; calcd for $C_{27}H_{32}Cl_2N_5O_5^+$: 576.18.

Synthesis of AV-11324-46:

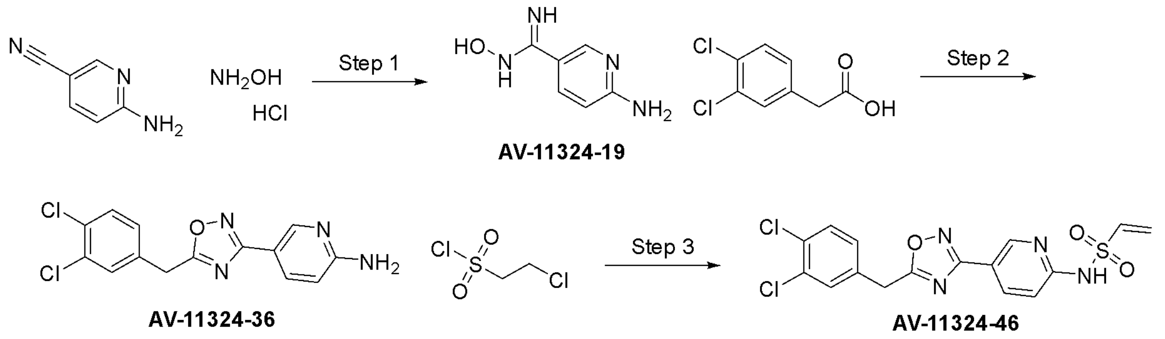

Step 1: 2-amino-5-cyanopyridine (503.7 mg, 4.20 mmol) and hydroxylamine HCl (354.1 mg, 5.10 mmol) were combined and suspended in EtOH (8 mL). Et_3N (2926 µL, 21.0 mmol) was added and the reaction was stirred at 65°C for 6 hours. The reaction was then removed from heat and stirred at room temperature for 12 hours. The desired product precipitated out of the reaction and was isolated via filtration (316.4 mg, 49.5%). The material was used in the next step without further purification. LC/MS (ESI) *m/z* 153.00 $[M+H]^+$; calcd for $C_6H_9N_4O^+$: 153.08.

Step 2: 3,4-dichlorophenylacetic acid (390.6 mg, 1.90 mmol) and CDI (462.1 mg, 2.85 mmol) were combined and suspended in MeCN (5 mL). The reaction was stirred at room temperature for 2 hours, at which point AV-11324-19 (316.4 mg, 2.08 mmol) was added to the reaction. The reaction was stirred at room temperature for 30 minutes. DBU was then added (568.3 µL, 3.80 mmol) and the reaction was heated to 60°C for 40 minutes. The reaction was diluted with EtOAc ad washed with water, saturated sodium bicarbonate solution, and brine. The organics were collected, dried over Na_2SO_4, filtered, and concentrated under reduced pressure. The crude material was purified via flash chromatography (DCM/MeOH) followed by preparative HPLC

(MeOH or CH_3CN/H_2O with 0.0425% TFA) to afford the desired product (32.5 mg, 5.3%). LC/MS (ESI) *m/z* 320.77 $[M+H]^+$; calcd for $C_{14}H_{11}Cl_2N_4O^+$: 321.03.

Step 3: AV-11324-36 (32.5 mg, 0.10 mmol) was suspended in DCM (2 mL) and placed in an ice bath. To the reaction was then added 2-chloroethanesulfonyl chloride (26.4 µL, 0.25 mmol) followed by Et_3N (69.7 µL, 0.50 mmol). The reaction was stirred at 0°C for 3 hours. The reaction was quenched with water, extracted with DCM, and then washed with brine. The organics were collected, dried over Na_2SO_4, filtered, and concentrated under reduced pressure. The crude material was purified via flash chromatography (DCM/MeOH) followed by preparative HPLC (MeOH or CH_3CN/H_2O with 0.0425% TFA) to afford the desired product (0.6 mg, 1.5%) LC/MS (ESI) *m/z* 410.87 [M+H]+; calcd for $C_{16}H_{12}Cl_2N_4O_3S^+$: 410.00. ^1H NMR (500 MHz, DMSO-d6) δ 8.38 (d, J = 2.2 Hz, 1H), 7.96 (dd, J = 9.3, 2.1 Hz, 1H), 7.72 (d, J = 2.1 Hz, 1H), 7.66 (d, J = 8.3 Hz, 1H), 7.41 (dd, J = 8.3, 2.1 Hz, 1H), 6.72 (d, J = 9.4 Hz, 1H), 4.76 – 4.65 (m, 2H), 4.49 (s, 2H), 3.55 – 3.43 (m, 2H).

Synthetic Procedure for AXA-01-045:

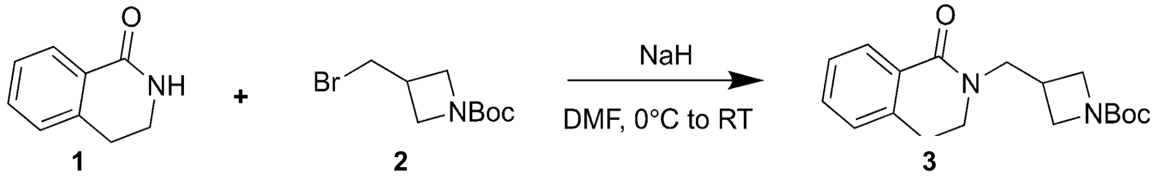

3,4-dihydroisoquinolin-1(2H)-one (1, 73.6 mg, 0.5 mmol) was taken up in DMF (2mL) and cooled to 0℃ and NaH (22 mg, 0.55 mmol) was added. The mixture was stirred at 0℃ for 30 minutes. Tert-butyl 3-(bromomethyl)azetidine-1-carboxylate (2,149.5 mg, 0.60 mmol) was added to the mixture, and the reaction was warmed to RT and stirred for 2.5 hrs. The reaction was diluted with water (25mL) and extracted with ethyl acetate (25mL x 2). The combined organics were washed with brine (1 x 50mL), dried over anhydrous Na_2SO_4, filtered, concentrated and purified by column chromatography on silica gel (0% to 100% Hexanes/ EtOAc) to afford 3 as a pale-yellow solid (89.9 mg, yield 56.7%). LCMS (m/z): 317.80 $[M + H]^+$; calcd for $C_{18}H_{25}N_2O_3^+$: 317.19.

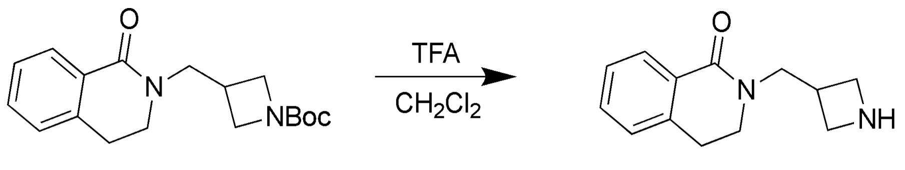

3 **4**

A mixture of 3 (89.9 mg, 0.284 mmol), CH_2Cl_2 (1.8 mL), and TFA (0.2 mL) was stirred at rt for 3 hrs, the reaction was concentrated in vacuum to leave the crude 4 as a white solid (quantitative yield). LCMS (m/z): 217.80 $[M + H]^+$; calcd for $C_{13}H_{17}N_2O^+$: 217.13.

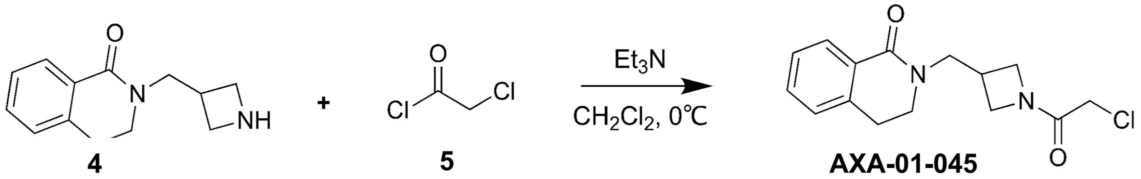

4 **5** **AXA-01-045**

A mixture of 4 (50.0 mg, 0.151 mmol) and Et_3N (46.4 µL, 0.333 mmol) was taken up in CH_2Cl_2 (4 mL) and cooled to 0℃. 2-chloroacetyl chloride (5, 24 µL, 0.303 mmol) was added to the reaction mixture. The reaction was warmed to rt and stirred overnight. The reaction was concentrated in vacuum. The mixture was purified by preparative HPLC (MeCN/H2O with 0.0425% TFA) to afford AXA-01-045 as a white solid (17.3 mg, yield 28.1%). LCMS ESI (m/z): 292.67 $[M + H]^+$; calcd for $C_{15}H_{17}Cl_2N_2O_2^+$: 292.76. ^1H NMR (500 MHz, DMSO-d6) δ 7.92 (dd, J = 8.1, 1.2 Hz, 1H), 7.68 (td, J = 7.5, 1.2 Hz, 1H), 7.58 – 7.25 (m, 2H), 4.45 (s, 2H), 4.36 – 4.15 (m, 2H), 3.76 (ddd, J = 13.7, 4.8, 1.9 Hz, 1H), 3.56 (dd, J = 13.7, 8.0 Hz, 1H), 3.40 – 3.29 (m, 2H), 3.07 (t, J = 6.8 Hz, 2H), 2.69 – 2.55 (m, 1H).

Synthetic Procedure for AXA-01-055:

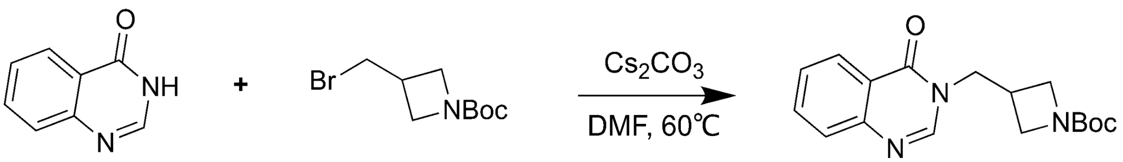

6 **2** **8**

Quinazolin-4(3H)-one (6, 73.1 mg, 0.5 mmol), tert-butyl 3-(bromomethyl)azetidine-1-carboxylate (2, 150.0 mg, 0.6 mmol), and Cs_2CO_3 (325.82 mg, 1.0 mmol) were taken up in DMF (2 mL) and stirred at 60℃ for 3 hours. The reaction was diluted with water (25 mL) and extracted with ethyl

acetate (25 mL x 2). The combined organics were washed with brine (50 mL x 1), dried over anhydrous Na_2SO_4, filtered, concentrated and purified by flash chromatography (50% to 100% EtOAc /hexanes) to afford 152.6 mg product 8 (96.8%). LCMS (m/z): 316.37 $[M + H]^+$; calcd for $C_{17}H_{22}N_3O_3^+$: 316.17.

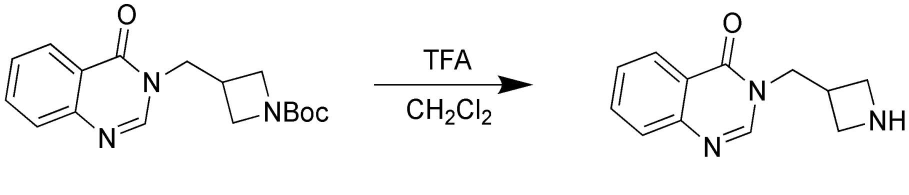

8 **9**

A mixture of tert-butyl-3-((4-oxoquinazolin-3(4H)-yl)methyl)azetidine-1-carboxylate (8, 152.6 mg, 0.484 mmol), CH_2Cl_2 (1.8 mL), and TFA (0.2 mL) was stirred at rt for 3 hours. The reaction was concentrated in vacuum to leave the crude 9 as a white solid (quantitative yield). LCMS (m/z): 216.37 $[M + H]^+$. calcd for $C_{12}H_{14}N_3O^+$: 216.11.

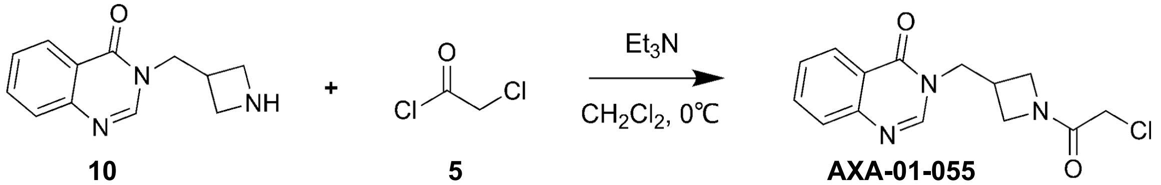

10 **5** **AXA-01-055**

A mixture of 10 (25.0 mg, 0.116 mmol) and Et_3N (81.0 µL, 0.580 mmol) was taken up in CH_2Cl_2 (2 mL) and cooled to 0℃. 2-chloroacetyl chloride (5, 18.5 µL, 0.232 mmol) was added to the reaction mixture. The reaction was warmed to rt and stirred for 2 hours. The mixture was purified by flash chromatography (5% to 20% MeOH/EtOAc), followed by preparative HPLC (MeCN/H2O with 0.0425% TFA) to afford 12.6 mg product (37.8%). LCMS ESI (m/z): 292.74 $[M + H]^+$. ; calcd for $C_{15}H_{17}Cl_2N_3O_2^+$: 292.76. ^{1}H NMR (500 MHz, DMSO-d6) δ 8.48 (s, 1H), 8.17 (dd, J = 8.1, 1.5 Hz, 1H), 7.85 (ddd, J = 8.4, 7.1, 1.6 Hz, 1H), 7.69 (dd, J = 8.2, 1.1 Hz, 1H), 7.57 (ddd, J = 8.2, 7.1, 1.2 Hz, 1H), 4.32 – 4.21 (m, 3H), 4.07 (dd, J = 8.9, 5.6 Hz, 2H), 3.97 (t, J = 9.2 Hz, 1H), 3.80 (dd, J = 9.9, 5.7 Hz, 1H), 3.17 – 3.07 (m, 1H).

<u>Synthesis of the scaffold:</u> <u>benzo[d]thiazole-2-carboxylic acid</u>

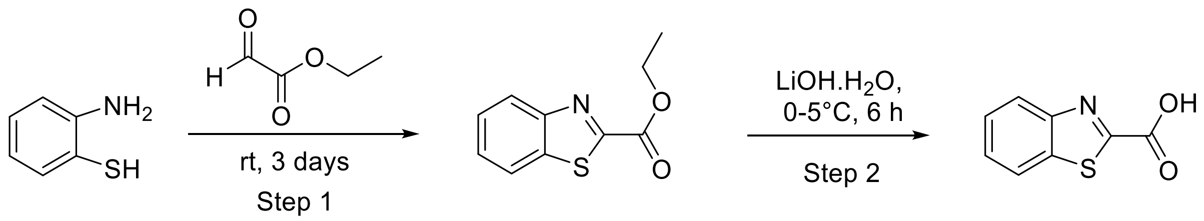

Step 1: A mixture of 2-aminobenzenethiol (2.76mL, 25.6mmol) and ethyl 2-oxoacetate (50% in toluene) (6.28mL, 30.7mmol) was stirred at room temperature for 3 days. The mixture was diluted with EtOAc, and washed with H_2O three times. The organic layer was then washed with brine and dried over Na_2SO_4, filtered, and concentrated under reduced pressure. The crude material was purified by flash column chromatography (EtOAc in hexanes, 20% to 60%) and preparative HPLC (MeCN/H_2O with 0.0425% TFA) to afford desired product (1.8g, 34%). LCMS ESI (*m/z*): 208.18; $[M+H]^+$ calcd for $C_{10}H_{10}NO_2S^+$: 208.04

Step 2: To a solution of ethyl benzo[d]thiazole-2-carboxylate (0.80g, 3.8mmol) in H_2O (16mL) and THF (12mL) was added a solution of lithium hydroxide monohydrate (0.16g, 3.8mmol) in water at 0~5°C, then stirred at 0~5°C for 6 hours. The mixture was diluted with H2O (~50mL), and adjusted to pH=4~5 with 2N HCl. The precipitate was collected by filtration, washed with water, and dried in vacuo to afford off-white solid product (0.52g). The filtrate was extracted with DCM 3 times. Combined organic layers were washed with brine and dried over Na_2SO_4, filtered and concentrated to afford desired product as yellowish solid (0.13g). LCMS ESI (*m/z*): 180.01; $[M+H]^+$ calcd for $C_8H_6NO_2S^+$: 180.01

Synthesis of WH-9943-094, 098A, 098B:

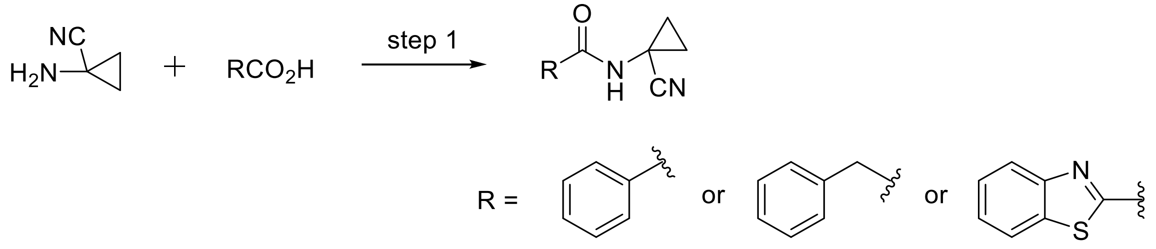

1-aminocyclopropane-1-carbonitrile (1.0 eq.), carboxylic acids (1.2 eq.) Et_3N (5.0 eq.) and HATU (1.5 eq.) were added into DMF (3-5mL). The mixture was stirred at room temperature overnight. If necessary, the mixture was diluted with EtOAc (50mL), and washed with brine (30mL×2) to

remove excess DMF. Organic layer was dried over anhydrous sodium sulfate (Na_2SO_4), filtered, and concentrated under reduced pressure. The crude material was then purified by flash column chromatography (hexanes/EtOAc/MeOH) and preparative HPLC (MeCN/H_2O with 0.0425% TFA) to afford desired products.

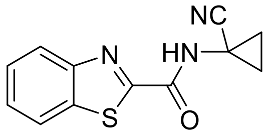

WH-9943-094 (N-(1-cyanocyclopropyl)benzo[d]thiazole-2-carboxamide (0.03g, 55%)). 1H NMR (500 MHz, DMSO-d6) δ 10.20 (s, 1H), 8.27 (dd, J = 8.2, 1.4 Hz, 1H), 8.16 (dd, J = 8.0, 1.6 Hz, 1H), 7.75 – 7.55 (m, 2H), 1.60 (q, 2H), 1.42 (q, 2H). LCMS ESI (*m/z*): 243.98; $[M+H]^+$ calcd for $C_{12}H_{10}N_3OS^+$: 244.05

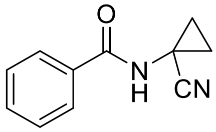

WH9943-098A (N-(1-cyanocyclopropyl)benzamide (0.067g, 85%)). 1H NMR (500 MHz, DMSO-d6) δ 9.32 (s, 1H), 7.89 – 7.80 (m, 2H), 7.63 – 7.54 (m, 1H), 7.54 – 7.45 (m, 2H), 1.57 (q, 2H), 1.29 (q, 2H). LCMS ESI (*m/z*): 187.19; $[M+H]^+$ calcd for $C_{11}H_{11}N_2O^+$: 187.09

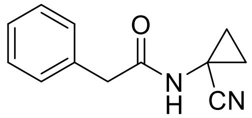

WH-9943-098B (N-(1-cyanocyclopropyl)-2-phenylacetamide (0.087g, 100%)). 1H NMR (500 MHz, DMSO-d6) δ 9.00 (s, 1H), 7.37 – 7.28 (m, 2H), 7.28 – 7.20 (m, 3H), 3.42 (s, 2H), 1.47 (q, 2H), 1.12 (q, 2H). LCMS ESI (*m/z*): 201.08; $[M+H]^+$ calcd for $C_{12}H_{13}N_2O^+$: 201.09

<u>Synthesis of WH-9943-102A, 103B, 103C, 104B:</u>

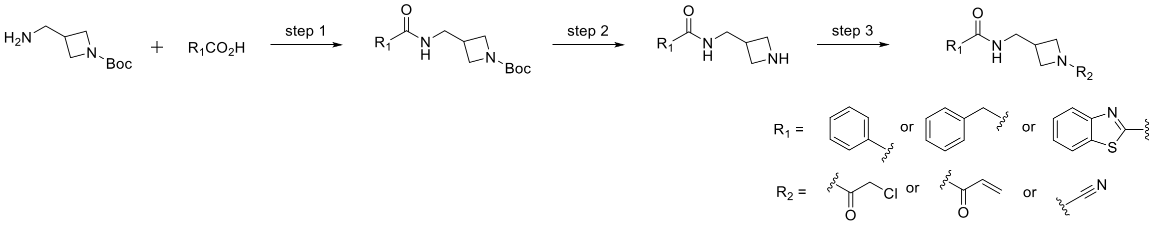

Step 1: The synthesis was preformed according to General Procedure 1 with tert-butyl 3-(aminomethyl)azetidine-1-carboxylate (1.2eq.), carboxylic acids (1.0eq.), Et_3N (3.0eq.), HATU (1.5eq.). Desired compounds were obtained (when R=phenyl, 0.34g (71%), LCMS ESI (*m/z*): 235.21 (m−*t*-butyl); $[M+H]^+$ calcd for $C_{16}H_{23}N_2O_3^+$: 291.17, when R=benzyl, 0.44g (98%), LCMS ESI (*m/z*): 249.08 (m−*t*-butyl); $[M+H]^+$ calcd for $C_{17}H_{25}N_2O_3^+$: 305.19, when R=benzylthiazol, 0.27g (42%) LCMS ESI (*m/z*): 292.02 (m−*t*-butyl); $[M+H]^+$ calcd for $C_{17}H_{22}N_3O_3S^+$: 348.14

Step 2: The synthesis was performed according to the General Procedure 1 with Boc-protected azetidine derivatives using 4N HCl in dioxane. Reaction mixtures were concentrated under reduced pressure to afford crude material which were used directly without any further purification.

Step 3: The synthesis was performed according to the General Procedure 2 with those azetidines (1.0eq.) and chloroacetyl chloride (1.5eq.), or acryloyl chloride (1.5eq.), or cyanogen bromide (1.5eq.)

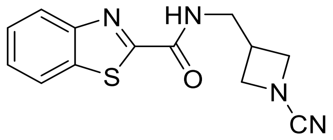

WH-9943-102A (N-((1-cyanoazetidin-3-yl)methyl)benzo[d]thiazole-2-carboxamide (3mg, 8%)). 1H NMR (500 MHz, DMSO-d6) δ 9.44 (t, J = 6.1 Hz, 1H), 8.24 (d, J = 7.9, 1.2 Hz, 1H), 8.15 (d, 1H), 7.71 – 7.54 (m, 2H), 4.22 (t, J = 8.0 Hz, 2H), 3.97 (dd, J = 7.7, 5.7 Hz, 2H), 3.55 (t, J = 6.5 Hz, 2H), 3.02 – 2.89 (m, 1H). LCMS ESI (*m/z*): 272.97; $[M+H]^+$ calcd for $C_{13}H_{13}N_4OS^+$: 273.08

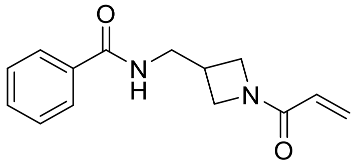

WH-9943-103B (N-((1-acryloylazetidin-3-yl)methyl)benzamide (6mg, 9%)). 1H NMR (500 MHz, DMSO-d6) δ 8.31 (t, J = 5.7 Hz, 1H), 7.93 – 7.80 (m, 2H), 7.52 – 7.46 (m, 1H), 7.46 – 7.40 (m, 2H), 6.28 (dd, J = 17.1, 10.1 Hz, 1H), 6.14 (dd, J = 17.1, 2.2 Hz, 1H), 5.64 (dd, J = 10.1, 2.2 Hz, 1H), 4.49 – 4.34 (m, 1H), 4.11 – 4.04 (m, 1H), 3.66 – 3.57 (m, 1H), 3.30 (dd, J = 16.7, 7.4 Hz, 1H), 3.25 – 3.19 (m, 2H), 2.24 – 2.08 (m, 1H). LCMS ESI (*m/z*): 245.28; $[M+H]^+$ calcd for $C_{14}H_{17}N_2O_2^+$: 245.13

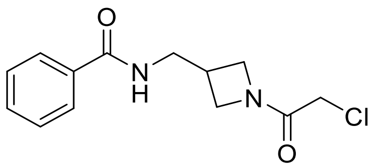

WH-9943-103C (N-((1-(2-chloroacetyl)azetidin-3-yl)methyl)benzamide (18mg, 26%)). 1H NMR (500 MHz, DMSO-d6) δ 8.42 (t, J = 5.9 Hz, 1H), 7.91 – 7.78 (m, 2H), 7.49 – 7.44 (m, 1H), 7.44 – 7.37 (m, 2H), 4.40 – 4.32 (m, 1H), 4.09 (s, 2H), 4.03 (dd, J = 10.6, 8.2 Hz, 1H), 3.62 – 3.53 (m, 1H), 3.26 (dd, J = 16.7, 7.8 Hz, 1H), 3.16 (t, J = 6.7 Hz, 2H), 2.21 – 2.01 (m, 1H). ^{13}C NMR (126 MHz, DMSO) δ 167.20, 166.14, 134.82, 131.69, 128.74, 127.68, 53.86, 51.86, 42.48, 40.42, 28.98. LCMS ESI (*m/z*): 267.27; $[M+H]^+$ calcd for $C_{13}H_{16}ClN_2O_2^+$: 267.09

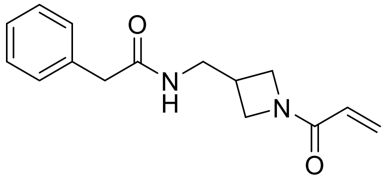

WH-9943-104B N-((1-acryloylazetidin-3-yl)methyl)-2-phenylacetamide (20mg, 27%). 1H NMR (500 MHz, DMSO-d6) δ 8.25 (t, J = 5.9 Hz, 1H), 7.37 – 7.16 (m, 5H), 6.26 (dd, J = 16.9, 10.3 Hz, 1H), 6.08 (dd, J = 16.9, 2.3 Hz, 1H), 5.65 (dd, J = 10.3, 2.3 Hz, 1H), 4.20 (t, J = 8.5 Hz, 1H), 3.90 (t, J = 10.1, 8.5 Hz, 1H), 3.84 (dd, J = 8.7, 5.4 Hz, 1H), 3.61 (dd, J = 10.1, 5.5 Hz, 1H), 3.42 (s, 2H), 3.32 – 3.22 (m, 2H), 2.80 – 2.64 (m, 1H). LCMS ESI (*m/z*): 259.27; $[M+H]^+$ calcd for $C_{15}H_{19}N_2O_2^+$: 259.14

Synthesis of WH-9943-105A, 105B, 105C:

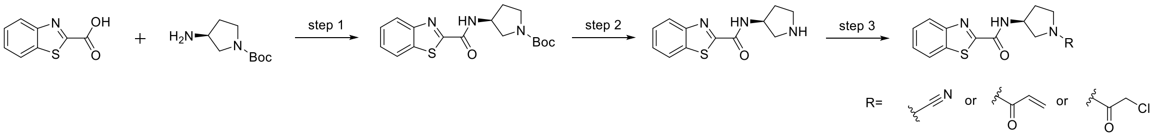

Step 1: The synthesis was preformed according to General Procedure 1 with benzo[d]thiazole-2-carboxylic acid (0.2g, 1.1mmol) and tert-butyl (S)-3-aminopyrrolidine-1-carboxylate (0.25g, 1.3mmol). 0.3g desired compound tert-butyl (S)-3-(benzo[d]thiazole-2-carboxamido)pyrrolidine-1-carboxylate was obtained (77%). LCMS ESI (*m/z*): 292.12 (m−*t*-butyl); $[M+H]^+$ calcd for $C_{17}H_{22}N_3O_3S^+$: 348.14

Step 2: The synthesis was performed according to the General Procedure 1 with tert-butyl (S)-3-(benzo[d]thiazole-2-carboxamido)pyrrolidine-1-carboxylate (0.3g, mmol). 0.29g (S)-N-(pyrrolidin-3-yl)benzo[d]thiazole-2-carboxamide (quant.). LCMS ESI (*m/z*): 247.88; $[M+H]^+$ calcd for $C_{12}H_{14}N_3OS^+$: 248.09

Step 3: The synthesis was performed according to the General Procedure 1 with (S)-N-(pyrrolidin-3-yl)benzo[d]thiazole-2-carboxamide (0.05g, 0.2mmol) and cyanogen bromide (35mg, 0.3mmol) , or acryloyl chloride (30mg, 0.3mmol), or chloroacetyl chloride (37mg, 0.3mmol).

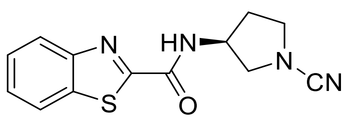

WH-9943-105A ((*S*)-N-(1-cyanopyrrolidin-3-yl)benzo[d]thiazole-2-carboxamide (34mg, 57%)). 1H NMR (500 MHz, DMSO-d6) δ 9.52 (s, 1H), 8.24 (d, J = 8.3, 1.3 Hz, 1H), 8.16 (d, J = 8.1, 1.2 Hz, 1H), 7.73 – 7.51 (m, 2H), 4.60 – 4.49 (m, 1H), 3.65 (dd, J = 9.7, 6.7 Hz, 1H), 3.63 – 3.56 (m, 1H), 3.49 – 3.42 (m, 2H), 2.22 – 2.11 (m, 1H), 2.11 – 2.02 (m, 1H). LCMS ESI (*m/z*): 273.27; $[M+H]^+$ calcd for $C_{13}H_{13}N_4OS^+$: 273.08

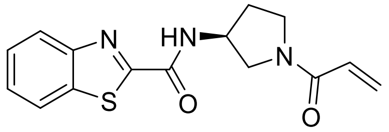

WH-9943-105B ((S)-N-(1-acryloylpyrrolidin-3-yl)benzo[d]thiazole-2-carboxamide (12mg, 18%)). 1H NMR (500 MHz, DMSO-d6) δ 8.42 (t, J = 5.9 Hz, 1H), 7.91 – 7.78 (m, 2H), 7.49 – 7.44 (m, 1H), 7.44 – 7.37 (m, 2H), 4.40 – 4.32 (m, 1H), 4.09 (s, 2H), 4.03 (dd, J = 10.6, 8.2 Hz, 1H), 3.62 – 3.53 (m, 1H), 3.26 (dd, J = 16.7, 7.8 Hz, 1H), 3.16 (t, J = 6.7 Hz, 2H), 2.21 – 2.01 (m, 1H). LCMS ESI (*m/z*): 302.27; $[M+H]^+$ calcd for $C_{15}H_{16}N_3O_2S^+$: 302.10

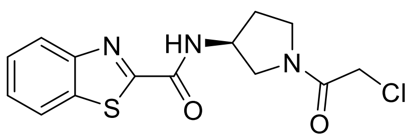

WH-9943-105C ((S)-N-(1-(2-chloroacetyl)pyrrolidin-3-yl)benzo[d]thiazole-2-carboxamide (26mg, 36%)) 1H NMR (500 MHz, DMSO-d6) δ 9.48 (dd, J = 14.3, 7.1 Hz, 1H), 8.24 (d, 1H), 8.15 (d, J = 8.1 Hz, 1H), 7.63 (t, 1H), 7.59 (t, 1H), 4.63 – 4.48 (m, 1H), 4.39 – 4.28 (m, 2H), 3.80 (dd, J = 10.6, 6.5

Hz, 0.5H), 3.75 – 3.61 (m, 1H), 3.61 – 3.49 (m, 1.5H), 3.50 – 3.37 (m, 1H), 2.33 – 2.03 (m, 2H). LCMS ESI (*m/z*): 324.17; $[M+H]^+$ calcd for $C_{14}H_{15}ClN_3O_2S^+$: 324.06

Synthesis of WH-9943-107B, 107C:

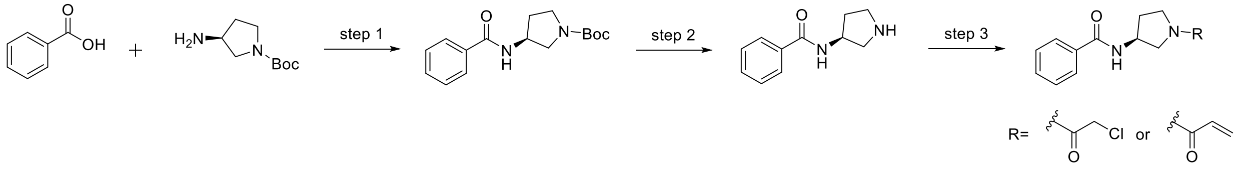

Step 1: The synthesis was preformed according to General Procedure 1 with benzoic acid (0.2g, 1.6mmol) and tert-butyl (S)-3-aminopyrrolidine-1-carboxylate (0.37g, 2.0mmol). 0.45g desired compound tert-butyl tert-butyl (S)-3-benzamidopyrrolidine-1-carboxylate was obtained (95%). LCMS ESI (*m/z*): 235.21 (m−*t*-butyl); $[M+H]^+$ calcd for $C_{16}H_{23}N_2O_3^+$: 291.17

Step 2: The synthesis was performed according to the General Procedure 1 with tert-butyl (S)-3-benzamidopyrrolidine-1-carboxylate (0.45g, 1.5mmol). 0.34g (S)-N-(pyrrolidin-3-yl)benzamide (quant.). LCMS ESI (*m/z*): 191.09; $[M+H]^+$ calcd for $C_{11}H_{15}N_2O^+$: 191.12

Step 3: The synthesis was performed according to the General Procedure 1 with (S)-N-(pyrrolidin-3-yl)benzamide (50mg, 0.2mmol) and chloroacetyl chloride (37mg, 0.3mmol), or acryloyl chloride (30mg, 0.3mmol).

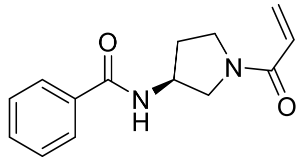

WH-9943-107B ((S)-N-(1-acryloylpyrrolidin-3-yl)benzamide (29mg, 54%). 1H NMR (500 MHz, DMSO-d6) δ 8.66 (dd, J = 18.9, 6.7 Hz, 1H), 8.05 – 7.88 (m, 2H), 7.68 – 7.58 (m, 1H), 7.55 (td, J = 7.5, 2.2 Hz, 2H), 6.78 – 6.54 (m, 1H), 6.32 – 6.13 (m, 1H), 5.75 (td, J = 10.7, 2.4 Hz, 1H), 4.71 – 4.46 (m, 1H), 3.95 (dd, J = 10.6, 6.6 Hz, 0.5H), 3.85 – 3.47 (m, 3.5H), 2.37 – 2.14 (m, 1H), 2.15 – 1.95 (m, 1H). LCMS ESI (*m/z*): 245.28; $[M+H]^+$ calcd for $C_{14}H_{17}N_2O_2^+$: 245.13

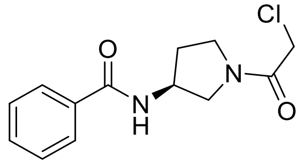

WH-9943-107C ((S)-N-(1-(2-chloroacetyl)pyrrolidin-3-yl)benzamide (30mg, 51%)). 1H NMR (500 MHz, DMSO-d6) δ 8.56 (dd, J = 20.4, 6.6 Hz, 1H), 7.91 – 7.80 (m, 2H), 7.58 – 7.51 (m, 1H), 7.50 – 7.42 (m, 2H), 4.60 – 4.40 (m, 1H), 4.38 – 4.24 (m, 2H), 3.81 (dd, J = 10.6, 6.5 Hz, 0.5H), 3.72 – 3.49 (m, 2H), 3.48 – 3.35 (m, 1.5H), 2.26 – 2.06 (m, 1H), 2.07 – 1.88 (m, 1H). LCMS ESI (*m/z*): 267.17; $[M+H]^+$ calcd for $C_{13}H_{16}ClN_2O_2^+$: 267.09

<u>Synthesis of WH-9943-108A, 108B, 108C:</u>

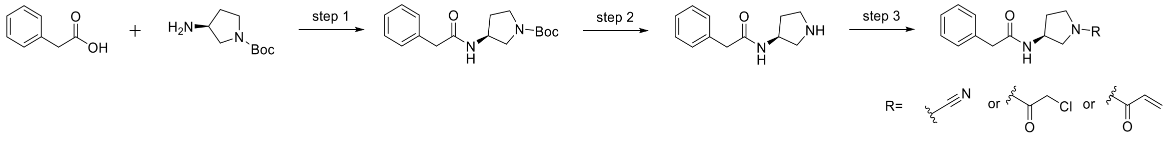

Step 1: The synthesis was preformed according to General Procedure 1 with 2-phenylacetic acid (0.20g, 1.5mmol) and tert-butyl (S)-3-aminopyrrolidine-1-carboxylate (0.33g, 1.8mmol). 0.42g desired compound tert-butyl (S)-3-(2-phenylacetamido)pyrrolidine-1-carboxylate was obtained (94%). LCMS ESI (*m/z*): 249.08 (m−*t*-butyl); $[M+H]^+$ calcd for $C_{17}H_{25}N_2O_3^+$: 305.19

Step 2: The synthesis was performed according to the General Procedure 1 with tert-butyl (S)-3-(2-phenylacetamido)pyrrolidine-1-carboxylate (0.42g, 1.4mmol). 0.39g (S)-2-phenyl-N-(pyrrolidin-3-yl)acetamide (quant.) . LCMS ESI (*m/z*): 204.98; $[M+H]^+$ calcd for $C_{12}H_{17}N_2O^+$: 205.13

Step 3: The synthesis was performed according to the General Procedure 1 (S)-2-phenyl-N-(pyrrolidin-3-yl)acetamide (0.06g, 0.25mmol) and cyanogen bromide (0.04g, 0.37mmol), or chloroacetyl chloride (0.04g, 0.37mmol), or acryloyl chloride (0.03g,0.37 mmol).

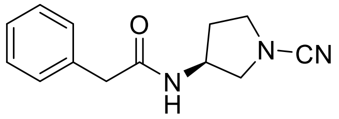

WH-9943-108A ((S)-N-(1-cyanopyrrolidin-3-yl)-2-phenylacetamide (31mg, 54%)). 1H NMR (500 MHz, DMSO-d6) δ 8.41 (d, J = 6.7 Hz, 1H), 7.40 – 7.04 (m, 5H), 4.27 – 4.16 (m, 1H), 3.54 (dd, J = 9.7, 6.1 Hz, 1H), 3.51 – 3.46 (m, 1H), 3.46 – 3.39 (m, 3H), 3.14 (dd, J = 9.7, 3.9 Hz, 1H), 2.11 – 1.97 (m, 1H), 1.84 – 1.70 (m, 1H). LCMS ESI (*m/z*): 230.38; $[M+H]^+$ calcd for $C_{13}H_{16}N_3O^+$: 230.13

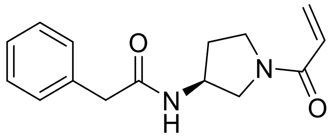

WH-9943-108B ((S)-N-(1-acryloylpyrrolidin-3-yl)-2-phenylacetamide (36mg, 56%)) 1H NMR (500 MHz, DMSO-d6) δ 8.38 (dd, J = 17.3, 6.7 Hz, 1H), 7.41 – 7.02 (m, 5H), 6.73 – 6.41 (m, 1H), 6.14 (ddd, J = 16.7, 10.6, 2.4 Hz, 1H), 5.67 (ddd, J = 15.8, 10.3, 2.4 Hz, 1H), 4.40 – 4.11 (m, 1H), 3.75 (dd, J = 10.6, 6.1 Hz, 0.5H), 3.68 – 3.15 (m, 3.5H), 2.20 – 1.93 (m, 1H), 1.92 – 1.63 (m, 1H). LCMS ESI (*m/z*): 259.27; $[M+H]^+$ calcd for $C_{15}H_{19}N_2O_2^+$: 259.14

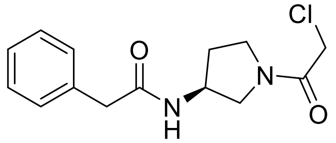

WH-9943-108C ((S)-N-(1-(2-chloroacetyl)pyrrolidin-3-yl)-2-phenylacetamide (26mg, 37%)) 1H NMR (500 MHz, DMSO-d6) δ 8.37 (dd, J = 13.4, 6.7 Hz, 1H), 7.42 – 7.11 (m, 5H), 4.36 – 4.14 (m, 3H), 3.70 (dd, J = 10.5, 6.2 Hz, 0.5H), 3.61 – 3.34 (m, 4.5H), 3.31 – 3.18 (m, 1H), 2.19 – 1.92 (m, 1H), 1.92 – 1.65 (m, 1H). LCMS ESI (*m/z*): 281.27; $[M+H]^+$ calcd for $C_{14}H_{18}ClN_2O_2^+$: 281.11

<u>Synthesis of WH-9943-118A, 118B,118C:</u>

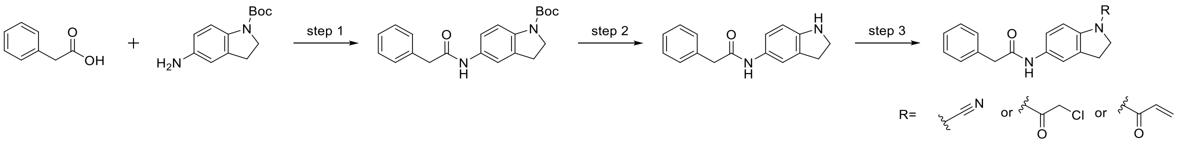

Step 1: The synthesis was preformed according to General Procedure 1 with 2-phenylacetic acid (0.20g, 1.5mmol) and tert-butyl 5-aminoindoline-1-carboxylate (0.42g, 1.7mmol). 0.52g desired compound tert-butyl 5-(2-phenylacetamido)indoline-1-carboxylate was obtained (quant.). LCMS ESI (*m/z*): 296.87 (m−*t*-butyl); $[M+H]^+$ calcd for $C_{21}H_{25}N_2O_3^+$: 353.19

Step 2: The synthesis was performed according to the General Procedure 1 with tert-butyl 5-(2-phenylacetamido)indoline-1-carboxylate (0.52g, 1.47mmol). 0.37g N-(indolin-5-yl)-2-phenylacetamide (quant.). LCMS ESI (*m/z*): 252.87; $[M+H]^+$ calcd for $C_{16}H_{17}N_2O^+$: 253.13

Step 3: The synthesis was performed according to the General Procedure 1 N-(indolin-5-yl)-2-phenylacetamide (0.07g, 0.24mmol) and cyanogen bromide (0.04g, 0.37mmol), or chloroacetyl chloride (0.033g, 0.37mmol), or acryloyl chloride (0.04g, 0.37mmol).

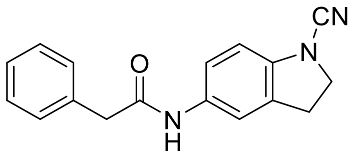

WH-9943-118A (N-(1-cyanoindolin-5-yl)-2-phenylacetamide (36mg, 53%)) 1H NMR (500 MHz, DMSO-d6) δ 10.14 (s, 1H), 7.58 (d, J = 1.9 Hz, 1H), 7.42 (dd, J = 8.5, 2.1 Hz, 1H), 7.38 – 7.30 (m, 4H), 7.29 – 7.23 (m, 1H), 6.87 (d, J = 8.5 Hz, 1H), 4.08 (t, J = 8.5 Hz, 2H), 3.63 (s, 2H), 3.15 (t, J = 8.5 Hz, 2H). LCMS ESI (*m/z*): 277.87; $[M+H]^+$ calcd for $C_{17}H_{16}N_3O^+$: 278.13

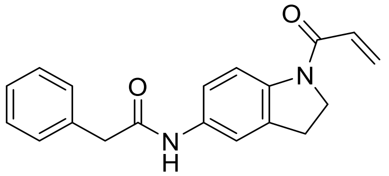

WH-9943-118B (N-(1-acryloylindolin-5-yl)-2-phenylacetamide (49mg, 66%)) 1H NMR (500 MHz, DMSO-d6) δ 10.12 (s, 1H), 8.07 (d, J = 8.8 Hz, 1H), 7.60 (d, J = 2.1 Hz, 1H), 7.38 – 7.28 (m, 5H), 7.28 – 7.21 (m, 1H), 6.72 (dd, J = 16.7, 10.3 Hz, 1H), 6.28 (dd, J = 16.7, 2.3 Hz, 1H), 5.79 (dd, J = 10.4, 2.2 Hz, 1H), 4.19 (t, J = 8.4 Hz, 2H), 3.62 (s, 2H), 3.13 (t, J = 8.4 Hz, 2H). LCMS ESI (*m/z*): 306.87; $[M+H]^+$ calcd for $C_{19}H_{19}N_2O_2^+$: 307.14

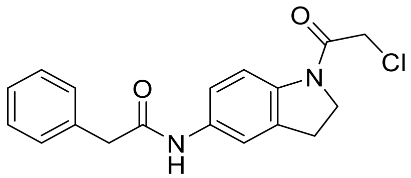

WH-9943-118C (N-(1-(2-chloroacetyl)indolin-5-yl)-2-phenylacetamide (33mg, 41%)) 1H NMR (500 MHz, DMSO-d6) δ 10.14 (s, 1H), 7.96 (d, J = 8.7 Hz, 1H), 7.61 (d, J = 2.0 Hz, 1H), 7.38 – 7.29 (m, 5H), 7.28 – 7.20 (m, 1H), 4.50 (s, 2H), 4.12 (t, J = 8.4 Hz, 2H), 3.62 (s, 2H), 3.14 (t, J = 8.4 Hz, 2H). LCMS ESI (*m/z*): 328.87; $[M+H]^+$ calcd for $C_{18}H_{18}ClN_2O_2^+$: 329.11

Synthesis of WH-9943-119A, 119B, 119C:

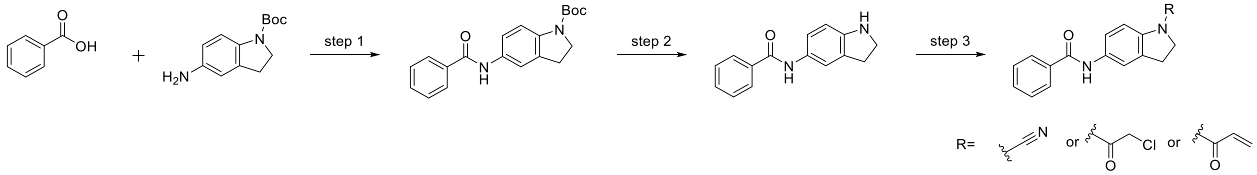

Step 1: The synthesis was preformed according to General Procedure 1 with benzoic acid (0.20g, 1.6mmol) and tert-butyl 5-aminoindoline-1-carboxylate (0.38g, 1.6mmol). 0.36g desired compound tert-butyl 5-benzamidoindoline-1-carboxylate was obtained (67%). LCMS ESI (*m/z*): **241.11** (m−*t*-butyl); $[M+H]^+$ calcd for $C_{20}H_{23}N_2O^+$: 339.17

Step 2: The synthesis was performed according to the General Procedure 1 with tert-butyl 5-benzamidoindoline-1-carboxylate (0.34g, 1.0mmol). 0.24g N-(indolin-5-yl)benzamide (quant.). LCMS ESI (*m/z*): 238.98; $[M+H]^+$ calcd for $C_{15}H_{15}N_2O^+$: 239.12

Step 3: The synthesis was performed according to the General Procedure 1 N-(indolin-5-yl)benzamide (0.05g, 0.18mmol) and cyanogen bromide (0.058g, 0.5mmol), or chloroacetyl chloride (0.031g, 0.27mmol), or acryloyl chloride (0.025g, 0.27mmol).

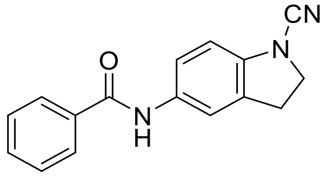

WH-9943-119A (N-(1-cyanoindolin-5-yl)benzamide (34mg, 71%) 1H NMR (500 MHz, DMSO-d6) δ 10.22 (s, 1H), 8.01 – 7.87 (m, 2H), 7.73 (d, J = 1.9 Hz, 1H), 7.65 – 7.56 (m, 2H), 7.57 – 7.48 (m, 2H), 6.92 (d, J = 8.5 Hz, 1H), 4.11 (t, J = 8.5 Hz, 2H), 3.19 (t, J = 8.5 Hz, 2H). LCMS ESI (*m/z*): 263.87; $[M+H]^+$ calcd for $C_{16}H_{14}N_3O^+$: 264.11

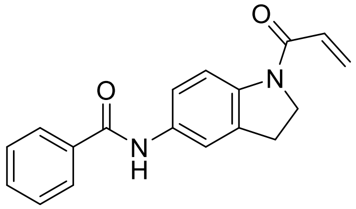

WH-9943-119B (N-(1-acryloylindolin-5-yl)benzamide (39mg, 73%)) 1H NMR (500 MHz, DMSO-d6) δ 10.22 (s, 1H), 8.13 (d, J = 8.7 Hz, 1H), 8.03 – 7.87 (m, 2H), 7.76 (d, J = 2.1 Hz, 1H), 7.63 – 7.56 (m, 1H), 7.56 – 7.48 (m, 3H), 6.75 (dd, J = 16.6, 10.3 Hz, 1H), 6.31 (dd, J = 16.6, 2.4 Hz, 1H), 5.81 (dd, J = 10.2, 2.3 Hz, 1H), 4.23 (t, J = 8.4 Hz, 2H), 3.19 (t, J = 9.4, 7.0 Hz, 2H). LCMS ESI (*m/z*): 292.97; $[M+H]^+$ calcd for $C_{18}H_{17}N_2O_2^+$: 293.13

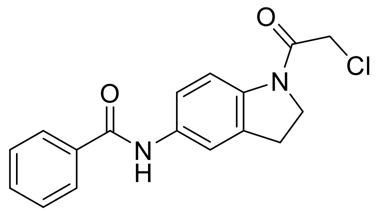

WH-9943-119C (N-(1-(2-chloroacetyl)indolin-5-yl)benzamide (43mg, 75%)) 1H NMR (500 MHz, DMSO-d6) δ 10.23 (s, 1H), 8.01 (d, J = 8.8 Hz, 1H), 7.98 – 7.91 (m, 2H), 7.78 (d, J = 2.1 Hz, 1H), 7.62 – 7.57 (m, 1H), 7.53 (dd, J = 8.2, 6.6 Hz, 3H), 4.53 (s, 2H), 4.16 (t, J = 8.4 Hz, 2H), 3.20 (t, J = 8.5 Hz, 2H). ^{13}C NMR (126 MHz, DMSO) δ 165.79, 164.12, 138.99, 135.77, 135.43, 132.68, 131.94, 128.83, 128.06, 119.60, 117.82, 116.30, 47.64, 44.20, 28.14. LCMS ESI (*m/z*): 314.87; $[M+H]^+$ calcd for $C_{17}H_{16}ClN_2O_2^+$: 315.09

<u>Synthesis of WH-9943-120A, 120B, 120C:</u>

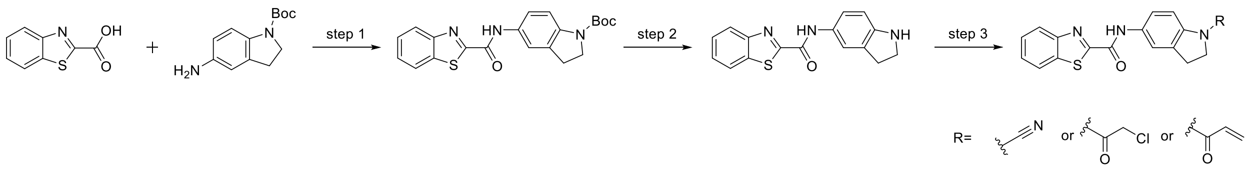

Step 1: The synthesis was preformed according to General Procedure 1 with benzo[d]thiazole-2-carboxylic acid (0.20g, 1.1mmol) and tert-butyl 5-aminoindoline-1-carboxylate (0.31g, 1.3mmol). 0.32g desired compound tert-butyl 5-(benzo[d]thiazole-2-carboxamido)indoline-1-carboxylate was obtained (73%). LCMS ESI (*m/z*): 340.20 (m−*t*-butyl); $[M+H]^+$ calcd for $C_{21}H_{22}N_3O_3S^+$: 396.14

Step 2: The synthesis was performed according to the General Procedure 2 with tert-butyl 5-(benzo[d]thiazole-2-carboxamido)indoline-1-carboxylate (0.32g, mmol). 0.24g N-(indolin-5-yl)benzo[d]thiazole-2-carboxamide (quant.). LCMS ESI (*m/z*): 295.87; $[M+H]^+$ calcd for $C_{16}H_{14}N_3OS^+$: 296.09

Step 3: The synthesis was performed according to the General Procedure N-(indolin-5-yl)benzo[d]thiazole-2-carboxamide (0.05g, 0.15mmol) and cyanogen bromide (0.048g, 0.45mmol), or chloroacetyl chloride (0.026g, 0.23mmol), or acryloyl chloride (0.021g, 0.23mmol).

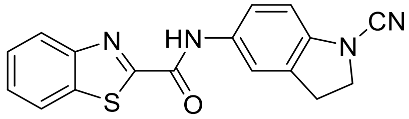

WH-9943-120A (N-(1-cyanoindolin-5-yl)benzo[d]thiazole-2-carboxamide (24mg, 50%))1H NMR (500 MHz, DMSO-d6) δ 11.01 (s, 1H), 8.17 (dd, 2H), 7.76 (d, J = 2.1 Hz, 1H), 7.70 (dd, J = 8.5, 2.1 Hz, 1H), 7.64 – 7.57 (m, 1H), 7.57 – 7.50 (m, 1H), 6.86 (d, J = 8.3 Hz, 1H), 4.05 (t, J = 8.5 Hz, 2H), 3.13 (t, J = 8.5 Hz, 2H). LCMS ESI (*m/z*): 320.87; $[M+H]^+$ calcd for $C_{17}H_{13}N_4OS^+$: 321.08

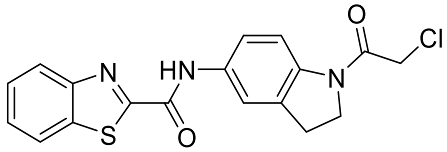

WH-9943-120C (N-(1-(2-chloroacetyl)indolin-5-yl)benzo[d]thiazole-2-carboxamide (35mg, 67%)). 1H NMR (500 MHz, DMSO-d6) δ 11.05 (s, 1H), 8.25 (dd, J = 8.0, 1.3 Hz, 1H), 8.21 (d, 1H), 8.14 (d, J = 8.7 Hz, 1H), 7.85 (d, J = 2.1 Hz, 1H), 7.71 (dd, J = 8.6, 2.2 Hz, 1H), 7.69 – 7.63 (m, 1H), 7.63 – 7.57 (m, 1H), 6.74 (dd, J = 16.6, 10.2 Hz, 1H), 6.31 (d, J = 16.6, 2.2 Hz, 1H), 5.81 (dd, J = 10.3, 2.2 Hz, 1H), 4.23 (t, J = 8.5 Hz, 2H), 3.19 (t, 2H). LCMS ESI (*m/z*): 371.77; $[M+H]^+$ calcd for $C_{18}H_{15}ClN_3O_2S^+$: 372.06

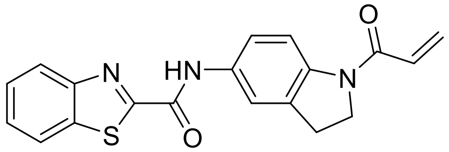

WH-9943-120B (N-(1-acryloylindolin-5-yl)benzo[d]thiazole-2-carboxamide (34mg, 61%)). 1H NMR (500 MHz, DMSO-d6) δ 11.07 (s, 1H), 8.26 (d, J = 8.0, 1.3 Hz, 1H), 8.22 (d, 1H), 8.03 (d, J = 8.7 Hz, 1H), 7.87 (d, J = 2.4 Hz, 1H), 7.72 (dd, J = 8.7, 2.2 Hz, 1H), 7.70 – 7.64 (m, 1H), 7.64 – 7.57 (m, 1H), 4.54 (s, 2H), 4.16 (t, J = 8.4 Hz, 2H), 3.20 (t, J = 8.2 Hz, 2H). LCMS ESI (*m/z*): 349.87; $[M+H]^+$ calcd for $C_{19}H_{16}N_3O_2S^+$: 350.10

<u>Synthesis of the scaffold: (E)-3-(phenylsulfonyl)prop-2-en-1-amine</u>

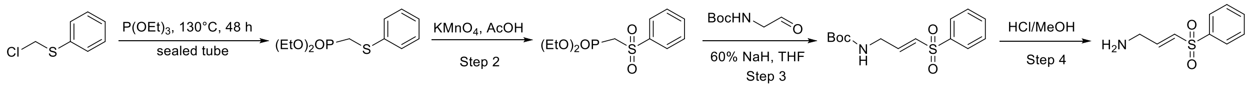

Step 1 and 2: A mixture of (chloromethyl)(phenyl)sulfane (1.26mL, 12.5mmol) and (2.14mL 12.3mmol) in a sealed tube was stirred at 130°C for 48 hours. The mixture was cooled to room temperature and dissolved in AcOH (10mL), $KMnO_4$ (3.74g, 23.6mmol) in H_2O (20mL) was added dropwisely to the solution slowly at 5 to 15°C. Then stirred at room temperature for 1 hour. Saturated $NaHCO_3$ aqueous solution was added until the mixture became colorless (below 15°C). The mixture was extracted with EtOAc twice. Combined organic layers were washed with water and brine, dried over Na2SO4, filtered and concentrated to afford crude material as colorless oil, which was purified by flash chromatography (EtOAc in hexanes, 0 to 100%) to afford product as colorless oil (XX) LCMS ESI (*m/z*): 292.77; $[M+H]^+$ calcd for $C_{11}H_{18}O_5PS^+$: 293.06

Step 3: Under N_2 atomsphere, to the solution of diethyl ((phenylsulfonyl)methyl)phosphonate (0.60g, 2.0mmol) in THF was added 60% NaH (0.099g, 2.5mmol) at at 0 to 5°C and the solution was stirred for 40min. N-Boc-2-aminoacetaldehyde (0.33g, 2.0mmol) was then added. The reaction was stirred for 15min at 5°C. The mixture was then quenched with saturated NH_4Cl and extracted with EtOAc. The combined organic layers were washed with brine and dried over Na_2SO_4, then filtered and concentrated to afford crude material which was purified by flash chromatography (EtOAc in hexanes, 0 to 100%) to afford product (0.27g, 45%). LCMS ESI (*m/z*): 197.87 (m−Boc); $[M+H]^+$ calcd for $C_{14}H_{20}NO_4S^+$: 298.11

Step 4: A solution of tert-butyl (E)-(3-(phenylsulfonyl)allyl)carbamate (0.27g, 0.9mmol) was dissolved in 2N HCl in MeOH. The solution was stirred at room temperature for 2 hours. The mixture was then concentrated, and the residue was triturated with acetonitrile. Precipitate was collected by filtration, washed with more acetonitrile and dried in vacuo to afford off-white solid as product (0.21g, quant.), LCMS ESI (*m/z*): 197.98; $[M+H]^+$ calcd for $C_9H_{12}NO_2S^+$: 198.06

Synthesis of WH-9943-127A, 127B:

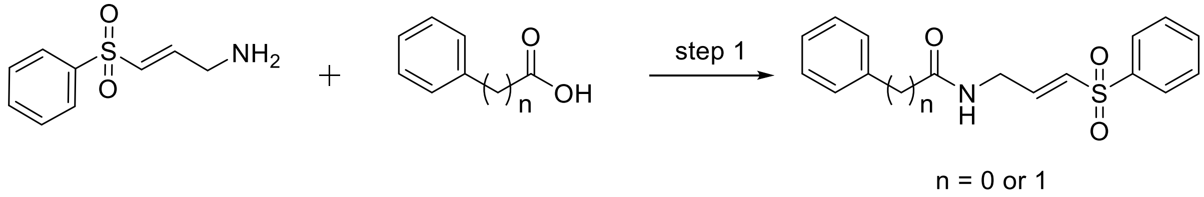

Step 1: (E)-3-(phenylsulfonyl)prop-2-en-1-amine (0.07g, 0.3mmol, 1.0 eq.), carboxylic acids (1.5 eq.) Et_3N (5.0 eq.) and HATU (1.5 eq.) were added into DMF (3-5mL). The mixture was stirred at room temperature overnight. If necessary, the mixture was diluted with EtOAc (50mL), and washed with brine (30mL×2) to remove excess DMF. Organic layer was dried over anhydrous sodium sulfate (Na_2SO_4), filtered, and concentrated under reduced pressure. The crude material was then purified by flash column chromatography (hexanes/EtOAc) and preparative HPLC (preparative HPLC (MeCN/H_2O with 0.0425% TFA)

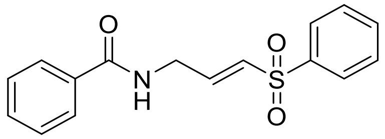

WH-9943-127A ((E)-N-(3-(phenylsulfonyl)allyl)benzamide.(10mg, 11%)) ^{1}H NMR (500 MHz, DMSO-d_6) δ 9.72 (d, *J* = 10.7 Hz, 1H), 7.96 – 7.90 (m, 2H), 7.77 – 7.72 (m, 2H), 7.71 – 7.66 (m, 1H), 7.66 – 7.57 (m, 3H), 7.52 (t, *J* = 8.2, 6.9 Hz, 2H), 7.01 (t, *J* = 10.7, 9.1 Hz, 1H), 4.66 (q, *J* = 8.5 Hz, 1H), 4.49 (d, *J* = 8.2 Hz, 2H). LCMS ESI (*m/z*): 301.87; $[M+H]^+$ calcd for $C_{16}H_{16}NO_3S^+$: 302.08

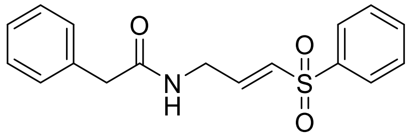

WH-9943-127B ((E)-2-phenyl-N-(3-(phenylsulfonyl)allyl)acetamide (12mg, 13%)) ^{1}H NMR (500 MHz, DMSO-d_6) δ 9.69 (d, *J* = 11.1 Hz, 1H), 7.93 – 7.84 (m, 2H), 7.77 – 7.68 (m, 1H), 7.65 – 7.59 (m, 2H), 7.36 – 7.28 (m, 2H), 7.27 – 7.18 (m, 3H), 6.81 – 6.68 (m, 1H), 4.51 (q, *J* = 8.5 Hz, 1H), 4.33 (dd, *J* = 8.1, 1.0 Hz, 2H), 3.48 (s, 2H). LCMS ESI (*m/z*): 315.97; $[M+H]^+$ calcd for $C_{17}H_{18}NO_3S^+$: 316.10

Synthesis of WH-9943-157A, 157B and WH-10417-038A:

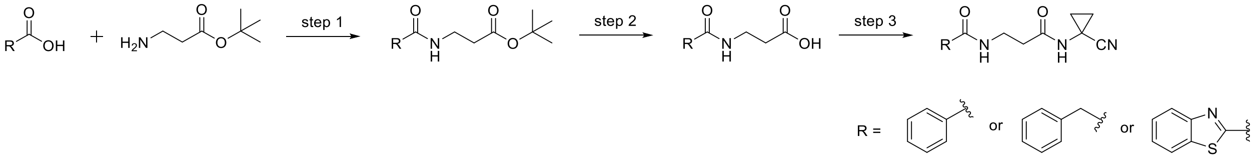

Step 1: The synthesis was preformed according to General Procedure 1 with tert-butyl 3-aminopropanoate (0.98g, 5.4mmol) and acids (1.0 eq.) and desired compounds were obtained

(when R=phenyl, 1.3g (96%), LCMS ESI (*m/z*): 193.99 (m−*t*-butyl); $[M+H]^+$ calcd for $C_{14}H_{20}NO_3^+$: 250.14, when R=benzyl, 0.64g (45%), LCMS ESI (*m/z*): 208.08 (m−*t*-butyl); $[M+H]^+$ calcd for $C_{15}H_{22}NO_3^+$: 264.16, when R=benzylthiazol, 0.25g (42%), LCMS ESI (*m/z*): 250.88 (m−*t*-butyl); $[M+H]^+$ calcd for $C_{15}H_{19}N_2O_3S^+$: 307.11)

Step 2: The synthesis was performed according to the General Procedure 1 with t-Butyl ester intermediates synthesized in Step 1. Free acid products were obtained by flash column chromatography (EtOAc in hexanes 0 to 100%) (when R=phenyl, 0.70g (70%), LCMS ESI (*m/z*): 193.99; $[M+H]^+$ calcd for $C_{10}H_{12}NO_3^+$: 194.08, when R=benzyl, 0.44g (82%), LCMS ESI (*m/z*): 208.08; $[M+H]^+$ calcd for $C_{11}H_{14}NO_3^+$: 208.10, when R=benzylthiazol, 0.2g (quant.), LCMS ESI (*m/z*): 250.98; $[M+H]^+$ calcd for $C_{11}H_{11}N_2O_3S^+$: 251.05)

Step 3: The synthesis was performed according to the General Procedure 1 with 1-aminocyclopropane-1-carbonitrile (0.04g, 0.34mmol) and acids (1.0eq.) synthesized in step 2, Et_3N (5.0eq.) and HATU (1.5eq.) The crude materials were purified by preparative HPLC (($MeCN/H_2O$ with 0.0425% TFA) to afford the products after concentration under reduced pressure, which were then triturated with DCM and collected by filtration to obtain the products as white solid.

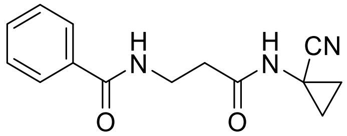

WH-9943-157A N-(3-((1-cyanocyclopropyl)amino)-3-oxopropyl)benzamide (42mg, 48%). ^1H NMR (500 MHz, DMSO-d_6) δ 8.82 (s, 1H), 8.53 (t, *J* = 5.7 Hz, 1H), 7.90 – 7.73 (m, 2H), 7.55 – 7.49 (m, 1H), 7.46 (dd, *J* = 8.2, 6.7 Hz, 2H), 3.46 (td, *J* = 7.0, 5.5 Hz, 2H), 2.39 (t, *J* = 7.0 Hz, 2H), 1.44 (q, 2H), 1.12 (q, 2H). LCMS ESI (*m/z*): 257.97; $[M+H]^+$ calcd for $C_{14}H_{16}N_3O_2^+$: 258.12

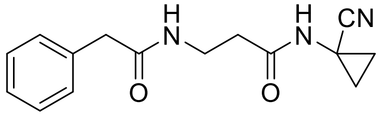

WH-9943-157B N-(1-cyanocyclopropyl)-3-(2-phenylacetamido)propenamide (22mg, 24%). ^1H NMR (500 MHz, DMSO-d_6) δ 8.77 (s, 1H), 8.12 (t, *J* = 5.8 Hz, 1H), 7.33 – 7.26 (m, 2H), 7.25 – 7.17

(m, 3H), 3.38 (s, 2H), 3.35 (s, 3H), 3.24 (q, *J* = 6.7 Hz, 2H), 2.25 (t, *J* = 6.9 Hz, 2H), 1.43 (q, 2H), 1.08 (q, 2H). LCMS ESI (*m/z*): 271.97; $[M+H]^+$ calcd for $C_{15}H_{18}N_3O_2^+$: 272.14

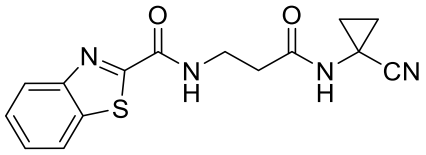

WH-10417-038A N-(3-((1-cyanocyclopropyl)amino)-3-oxopropyl)benzo[d]thiazole-2-carboxamide (31mg, 49%). 1H NMR (500 MHz, DMSO-d6) δ 9.14 (t, J = 5.9 Hz, 1H), 8.86 (s, 1H), 8.23 (d, J = 7.8 Hz, 1H), 8.14 (d, J = 8.0 Hz, 1H), 7.64 (t, 1H), 7.59 (t, J = 7.5 Hz, 1H), 3.53 (q, J = 6.9 Hz, 2H), 2.45 (t, J = 7.1 Hz, 2H), 1.45 (q, 2H), 1.13 (q, 2H). LCMS ESI (*m/z*): 315.17; $[M+H]^+$ calcd for $C_{15}H_{15}N_4O_2S^+$: 315.09

Synthesis of WH-9943-186:

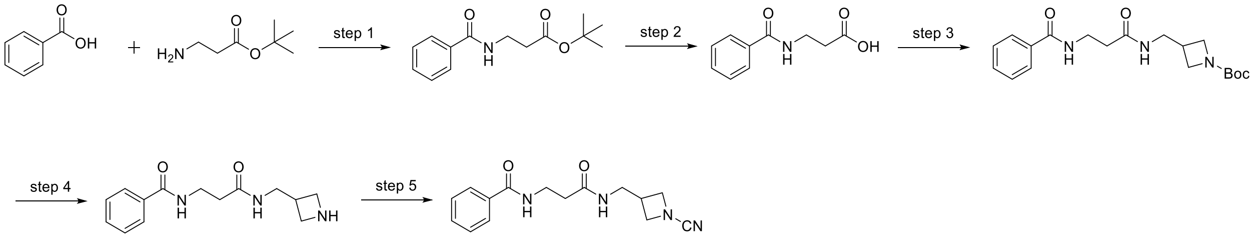

Step 1: The synthesis was preformed according to General Procedure 1 with tert-butyl 3-aminopropanoate (0.98g, 5.4mmol) and benzyl (1.0 eq.) and desired compounds were obtained (1.3g, 96%). LCMS ESI (*m/z*): 193.99 (m−*t*-butyl) ; $[M+H]^+$ calcd for $C_{14}H_{20}NO_3^+$: 250.14

Step 2: The synthesis was performed according to the General Procedure 1 with t-Butyl ester intermediates synthesized in Step 1. Free acid product was obtained by flash column chromatography (EtOAc in hexanes 0 to 100%): 0.70g (70%). LCMS ESI (*m/z*): 193.99 (m−*t*-butyl); $[M+H]^+$ calcd for $C_{10}H_{12}NO_3^+$: 194.08

Step 3: The synthesis was preformed according to General Procedure 1 with 3-benzamidopropanoic acid (0.07g, 0.36mmol) and tert-butyl 3-(aminomethyl)azetidine-1-carboxylate (0.08g, 0.44mmol), Et3N (0.15mL, 1.1mmol) and HATU (0.21g, 0.54mmol) 0.14g desired compound tert-butyl 3-((3-benzamidopropanamido)methyl)azetidine-1-carboxylate was obtained (quant.). LCMS ESI (*m/z*): 361.97; $[M+H]^+$ calcd for $C_{19}H_{28}N_3O^+$: 362.21

Step 4: The synthesis was performed according to the General Procedure 1 with tert-butyl 3-((3-benzamidopropanamido)methyl)azetidine-1-carboxylate (0.14g, 0.38mmol). 0.1g N-(3-((azetidin-3-ylmethyl)amino)-3-oxopropyl)benzamide (quant.). LCMS ESI (*m/z*): 261.97; $[M+H]^+$ calcd for $C_{14}H_{20}N_3O_2^+$: 262.16

Step 5: The synthesis was performed according to the General Procedure 1 N-(3-((azetidin-3-ylmethyl)amino)-3-oxopropyl)benzamide (0.23g, 0.6mmol) and cyanogen bromide (0.06g, 0.6mmol).

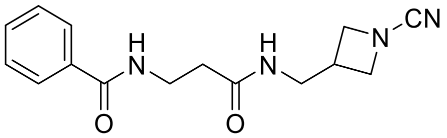

WH-9943-186 N-(3-(((1-cyanoazetidin-3-yl)methyl)amino)-3-oxopropyl)benzamide (57mg, 33%). 1H NMR (500 MHz, DMSO-d6) δ 8.44 (t, J = 5.6 Hz, 1H), 8.01 (t, J = 5.8 Hz, 1H), 7.81 – 7.70 (m, 2H), 7.48 – 7.41 (m, 1H), 7.41 – 7.33 (m, 2H), 4.04 (t, J = 7.9 Hz, 2H), 3.74 (dd, J = 7.6, 5.8 Hz, 2H), 3.39 (q, J = 7.1, 5.6 Hz, 2H), 3.19 (t, J = 6.3 Hz, 2H), 2.77 – 2.61 (m, 1H), 2.32 (t, J = 7.2 Hz, 2H). LCMS ESI (*m/z*): 286.97; $[M+H]^+$ calcd for $C_{15}H_{19}N_4O_2^+$: 287.15

Synthesis of WH-9943-188, 189 and WH-10417-046A:

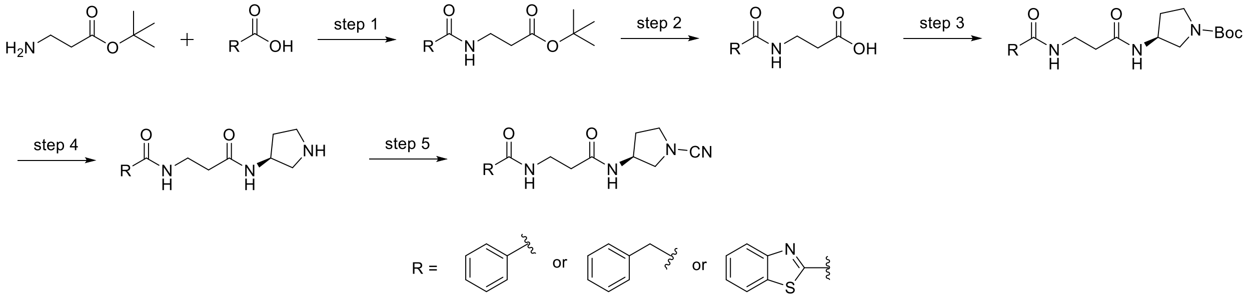

Step 1: The synthesis was preformed according to General Procedure 1 with tert-butyl 3-aminopropanoate (0.98g, 5.4mmol) and acids (1.0 eq.) and desired compounds were obtained (when R=phenyl, 1.3g (96%), LCMS ESI (*m/z*): 193.99 (m−*t*-butyl); $[M+H]^+$ calcd for $C_{14}H_{20}NO_3^+$: 250.14, when R=benzyl, 0.64g (45%), LCMS ESI (*m/z*): 208.08 (m−*t*-butyl); $[M+H]^+$ calcd for $C_{15}H_{22}NO_3^+$: 264.16, when R=benzylthiazol, 0.25g (42%), LCMS ESI (*m/z*): 250.88 (m-t-butyl); $[M+H]^+$ calcd for $C_{15}H_{19}N_2O_3S^+$: 307.11)

Step 2: The synthesis was performed according to the General Procedure 1 with t-Butyl ester intermediates synthesized in Step 1. Free acid products were obtained by flash column chromatography (EtOAc in hexanes 0 to 100%) (when R=phenyl, 0.70g (70%), LCMS ESI (*m/z*): 193.99; $[M+H]^+$ calcd for $C_{10}H_{12}NO_3^+$: 194.08, when R=benzyl, 0.44g (82%), LCMS ESI (*m/z*): 208.08; $[M+H]^+$ calcd for $C_{11}H_{14}NO_3^+$: 208.10, when R=benzylthiazol, 0.2g (quant.), LCMS ESI (*m/z*): 250.98; $[M+H]^+$ calcd for $C_{11}H_{11}N_2O_3S^+$: 251.05)

Step 3: The synthesis was preformed according to General Procedure 1 with tert-butyl (S)-3-aminopyrrolidine-1-carboxylate (0.08g, 0.44mmol) and acids (0.8eq.). Desired compounds were obtained (when R=phenyl, 0.14g (quant.), LCMS ESI (*m/z*): 261.87 (m−*Boc*); $[M+H]^+$ calcd for $C_{19}H_{28}N_3O_4^+$: 362.21, when R=benzyl, 0.15g (quant.), LCMS ESI (*m/z*): 275.97 (m−*Boc*); $[M+H]^+$ calcd for $C_{20}H_{30}N_3O_4^+$: 376.22, when R=benzylthiazol, 0.09g (quant.) LCMS ESI (*m/z*): 319.17 (m−*Boc*); $[M+H]^+$ calcd for $C_{20}H_{27}N_4O_4S^+$: 419.17).

Step 4: The synthesis was performed according to the General Procedure 1 with Boc protected intermediate (0.4mmol). The amines (TFA salt) were obtained (quant.)

Step 5: The synthesis was performed according to the General Procedure 1 with amines (1 eq.) and cyanogen bromide (1 eq.), Et_3N (10.0 eq.) in DMSO.

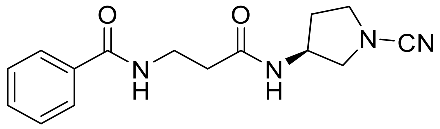

WH-9943-188 (S)-N-(3-((1-cyanopyrrolidin-3-yl)amino)-3-oxopropyl)benzamide (31mg, 40%). 1H NMR (500 MHz, DMSO-d6) δ 8.52 (t, J = 5.6 Hz, 1H), 8.24 (d, J = 6.7 Hz, 1H), 7.91 – 7.76 (m, 2H), 7.55 – 7.49 (m, 1H), 7.49 – 7.42 (m, 2H), 4.35 – 4.19 (m, 1H), 3.53 (dd, J = 9.7, 6.1 Hz, 1H), 3.51 – 3.36 (m, 4H), 3.20 – 3.12 (m, 1H), 2.38 (t, J = 7.1 Hz, 2H), 2.11 – 1.95 (m, 1H), 1.84 – 1.68 (m, 1H). ^{13}C NMR (126 MHz, DMSO) δ 171.03, 166.78, 134.97, 131.57, 128.72, 127.59, 117.74, 55.44, 49.26, 48.90, 36.55, 35.81, 31.28. LCMS ESI (*m/z*): 286.87; $[M+H]^+$ calcd for $C_{15}H_{19}N_4O_2^+$: 287.14

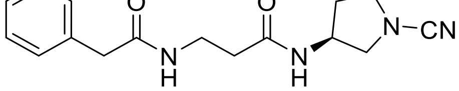

WH-9943-189 (S)-N-(1-cyanopyrrolidin-3-yl)-3-(2-phenylacetamido)propenamide (19mg, 25%). 1H NMR (500 MHz, DMSO-d6) δ 8.18 (d, J = 6.6 Hz, 1H), 8.09 (t, J = 5.8 Hz, 1H), 7.44 – 7.10 (m, 5H), 4.30 – 4.18 (m, 1H), 3.52 (dd, J = 9.7, 6.1 Hz, 1H), 3.48 – 3.36 (m, 4H), 3.25 (q, J = 6.7 Hz, 2H), 3.11 (dd, J = 9.7, 3.9 Hz, 1H), 2.25 (t, J = 6.9 Hz, 2H), 2.14 – 1.90 (m, 1H), 1.82 – 1.63 (m, 1H). LCMS ESI (*m/z*): 300.97; $[M+H]^+$ calcd for $C_{16}H_{21}N_4O_2^+$: 301.17

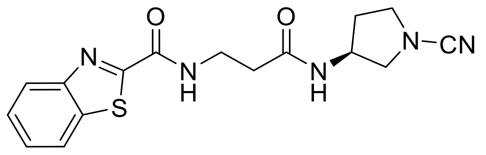

WH-10417-046A (S)-N-(3-((1-cyanopyrrolidin-3-yl)amino)-3-oxopropyl)benzo[d]thiazole-2-carboxamide (43mg, 48%). 1H NMR (500 MHz, DMSO-d6) δ 9.11 (t, J = 6.0 Hz, 1H), 8.27 (d, J = 6.7 Hz, 1H), 8.23 (d, J = 7.9 Hz, 1H), 8.14 (d, J = 8.0 Hz, 1H), 7.64 (t, 1H), 7.59 (t, 1H), 4.33 – 4.19 (m, 1H), 3.59 – 3.49 (m, 3H), 3.49 – 3.36 (m, 2H), 3.15 (dd, J = 9.7, 3.9 Hz, 1H), 2.44 (t, J = 7.2 Hz, 2H), 2.10 – 1.92 (m, 1H), 1.86 – 1.67 (m, 1H). LCMS ESI (*m/z*): 344.17; $[M+H]^+$ calcd for $C_{16}H_{18}N_5O_2S^+$: 344.12

Synthesis of WH-10417-053:

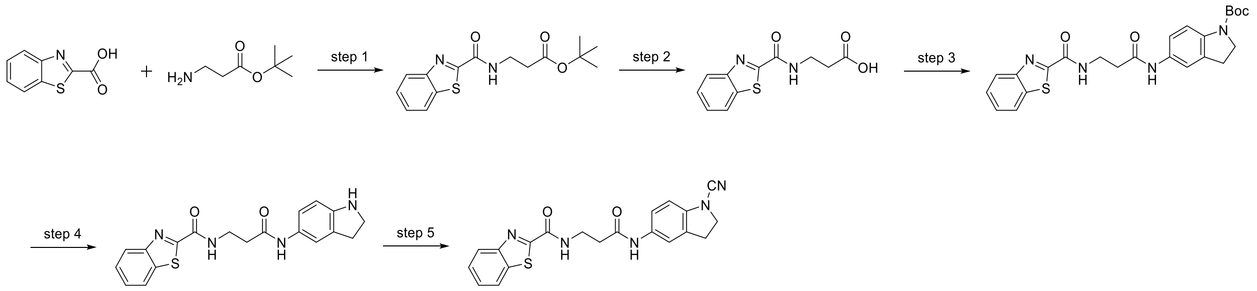

Step 1: The synthesis was preformed according to General Procedure 1 with tert-butyl 3-aminopropanoate (0.46g, 2.5mmol) and benzo[d]thiazole-2-carboxylic acid (0.35g, 1.9mmol.) and desired compounds were obtained (0.25g, 42%). LCMS ESI (*m/z*): 250.88 (m−*t*-butyl); $[M+H]^+$ calcd for $C_{15}H_{19}N_2O_3S^+$: 307.11

Step 2: The synthesis was performed according to the General Procedure 1 with t-Butyl ester intermediates synthesized in Step 1. Free acid products were obtained by flash column

chromatography (EtOAc in hexanes 0 to 100%) (0.2g, quant.) LCMS ESI (*m/z*): 250.98; $[M+H]^+$ calcd for $C_{11}H_{11}N_2O_3S^+$: 251.05

Step 3: The synthesis was preformed according to General Procedure 1 with 3-(benzo[d]thiazole-2-carboxamido)propanoic acid (0.05g, 0.2mmol) and tert-butyl 5-aminoindoline-1-carboxylate (0.05g, 0.2mmol). 0.06g desired compound tert-butyl 5-(3-(benzo[d]thiazole-2-carboxamido)propanamido)indoline-1-carboxylate was obtained (61%). LCMS ESI (*m/z*): 467.28; $[M+H]^+$ calcd for $C_{24}H_{27}N_4O_4S^+$: 467.17

Step 4: The synthesis was performed according to the General Procedure 1 with tert-butyl 5-(3-(benzo[d]thiazole-2-carboxamido)propanamido)indoline-1-carboxylate (0.06g, 0.13mmol) in 2mL 4N HCl in dioxane. 0.04g N-(3-(indolin-5-ylamino)-3-oxopropyl)benzo[d]thiazole-2-carboxamide (85%). LCMS ESI (*m/z*): 367.17; $[M+H]^+$ calcd for $C_{19}H_{19}N_4O_2S^+$: 367.12

Step 5: The synthesis was performed according to the General Procedure 3 N-(3-(indolin-5-ylamino)-3-oxopropyl)benzo[d]thiazole-2-carboxamide (0.04g, 0.1mmol) and cyanogen bromide (0.023g, 0.2mmol).

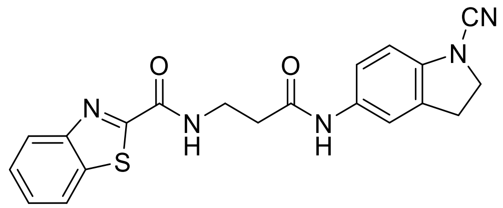

WH-10417-053 N-(3-((1-cyanoindolin-5-yl)amino)-3-oxopropyl)benzo[d]thiazole-2-carboxamide (15mg, 35%). 1H NMR (500 MHz, DMSO-d6) δ 9.73 (s, 1H), 8.24 (t, J = 5.7 Hz, 1H), 7.82 (d, J = 8.6 Hz, 1H), 7.47 (s, 1H), 7.38 – 7.24 (m, 3H), 7.22 (d, J = 8.7, 2.1 Hz, 1H), 3.87 (t, J = 8.8 Hz, 2H), 3.54 – 3.45 (m, 2H), 2.99 (t, J = 8.8 Hz, 2H), 2.51 (t, J = 6.8 Hz, 2H). LCMS ESI (*m/z*): 392.17; $[M+H]^+$ calcd for $C_{20}H_{18}N_5O_2S^+$: 392.12

<u>Synthesis of 3-(2-phenylacetamido)propanoic acid</u>

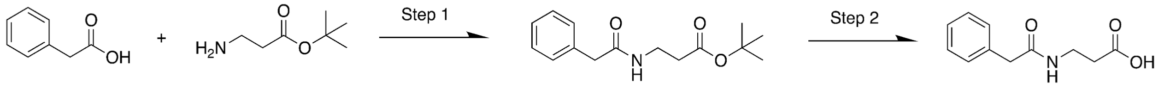

Step 1: The synthesis was preformed according to General Procedure 1 with *tert*-butyl 3-aminopropanoate (0.75 g, 4.13mmol) and 2-phenylacetic acid (1.12 g, 8.26mmol). 1.02 g desired compound (*tert*-butyl 3-(2-phenylacetamido)propanoate was obtained (94%).

Step 2: The synthesis was performed according to the General Procedure 1 with (*tert*-butyl 3-(2-phenylacetamido)propanoate (1.02 g, 3.87mmol) except for using 4N HCl in 1,4-dioxane instead of TFA/DCM. 0.802 g 3-(2-phenylacetamido)propanoic acid was obtained (quant.)

Synthesis of *tert*-butyl (*S*)-3-(3-aminopropanamido)pyrrolidine-1-carboxylate

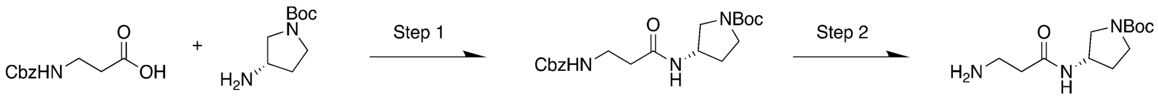

Step 1: The synthesis was preformed according to General Procedure 1 with *tert*-butyl (*S*)-3-aminopyrrolidine-1-carboxylate (0.625g, 3.36mmol) and 3-(((benzyloxy)carbonyl)amino)propanoic acid, (0.75g, 3.36mmol). 1.3g desired compound *tert*-butyl (*S*)-3-(3-(((benzyloxy)carbonyl)amino)propanamido)pyrrolidine-1-carboxylate was obtained (99%).

Step 2: The synthesis was performed according to the General Procedure 1 with *tert*-butyl (*S*)-3-(3-(((benzyloxy)carbonyl)amino)propanamido)pyrrolidine-1-carboxylate (1.3g, 0.357mmol) except for using activated palladium on carbon and methanol instead of TFA/DCM. 0.918g *tert*-butyl (*S*)-3-(3-aminopropanamido)pyrrolidine-1-carboxylate was obtained (quant.)

Synthesis of AF_11010_64, 112:

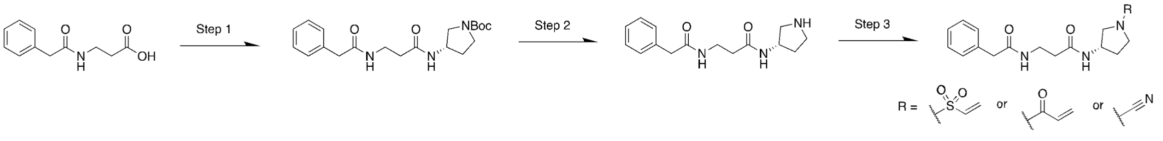

Step 1: The synthesis was performed according to the General Procedure 1 with 3-(2-phenylacetamido)propanoic acid (0.075g, 0.36mmol) and *tert*-butyl (*S*)-3-aminopyrrolidine-1-carboxylate (0.066mL, 0.36 mmol). 0.096g desired compound *tert*-butyl (*S*)-3-(3-(2-phenylacetamido)propanamido)pyrrolidine-1-carboxylate was obtained (71%).

Step 2: The synthesis was performed according to the General Procedure 1 with *tert*-butyl (*S*)-3-(3-(2-phenylacetamido)propanamido)pyrrolidine-1-carboxylate (0.096 g, 0.255 mmol) except for using 4N HCl in 1,4-dioxane instead of TFA/DCM. 0.070 g (*S*)-3-(2-phenylacetamido)-*N*-(pyrrolidin-3-yl)propanamide was obtained (quant.)

Step 3: The synthesis was performed according to the General Procedure 1 with *S*)-3-(2-phenylacetamido)-*N*-(pyrrolidin-3-yl)propanamide (0.05 g, 0.18 mmol) and ethenesulfonyl chloride (0.046mL, 0.44mmol), or acryloyl chloride (0.036mL, 0.44mmol).

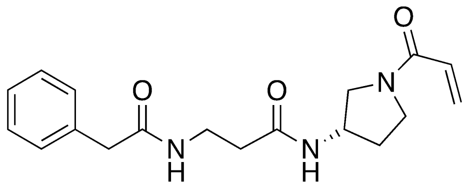

AF_11010_64 ^{1}H NMR (500 MHz, Methanol-*d*$_4$) δ 7.33 – 7.17 (m, 5H), 6.56 (ddd, *J* = 39.0, 16.8, 10.4 Hz, 1H), 6.27 (ddd, *J* = 16.8, 6.2, 2.0 Hz, 1H), 5.74 (ddd, *J* = 10.4, 7.5, 1.9 Hz, 1H), 4.35 (dp, *J* = 15.9, 5.6 Hz, 1H), 3.73 – 3.65 (m, 2H), 3.62 – 3.52 (m, 1H), 3.49 – 3.46 (m, 2H), 3.43 (td, *J* = 6.7, 1.8 Hz, 2H), 3.41 – 3.34 (m, 1H), 2.38 (td, *J* = 6.7, 1.0 Hz, 2H), 2.26 – 2.06 (m, 1H), 1.95 – 1.79 (m, 1H). LC/MS (ESI) *m/z* 329.77; [M+H]$^+$ calcd for $C_{18}H_{23}N_3O_3^+$: 330.17

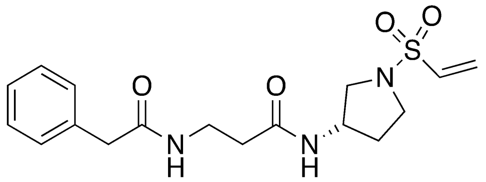

AF_11010_112 ^{1}H NMR (500 MHz, Methanol-*d*$_4$) δ 7.32 – 7.21 (m, 5H), 6.69 (dd, *J* = 16.6, 10.0 Hz, 1H), 6.19 (d, *J* = 16.6 Hz, 1H), 6.09 (d, *J* = 10.1 Hz, 1H), 4.29 – 4.24 (m, 1H), 3.48 (d, *J* = 3.3 Hz, 2H), 3.47 – 3.36 (m, 4H), 3.09 (ddd, *J* = 10.5, 4.7, 0.8 Hz, 1H), 2.38 (t, *J* = 6.7 Hz, 2H), 2.13 (ddt, *J* = 13.1, 8.1, 6.6 Hz, 1H), 1.86 – 1.77 (m, 1H). ^{13}C NMR (126 MHz, DMSO) δ 171.00, 170.66, 136.92, 132.17, 129.40, 129.03, 128.63, 126.75, 53.10, 49.21, 46.47, 42.77, 35.77, 35.59, 31.01. LC/MS (ESI) *m/z* 365.97; [M+H]$^+$ calcd for $C_{17}H_{23}N_3O_4S^+$: 366.14

<u>Synthesis of AF_11010_66:</u>

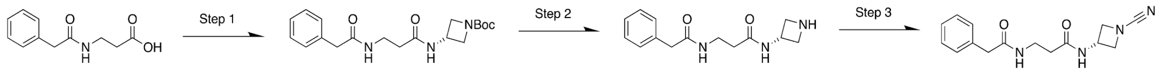

Step 1: The synthesis was performed according to the General Procedure 1 with 3-(2-phenylacetamido)propanoic acid (0.075g, 0.36mmol) and *tert*-butyl 3-aminoazetidine-1-carboxylate (0.057mL, 0.36mmol). 0.129g desired compound *tert*-butyl 3-(3-(2-phenylacetamido)propanamido)azetidine-1-carboxylate was obtained (99%).

Step 2: The synthesis was performed according to the General Procedure 1 with *tert*-butyl 3-(3-(2-phenylacetamido)propanamido)azetidine-1-carboxylate (0.129g, 0.357mmol) except for using 4N HCl in 1,4-dioxane instead of TFA/DCM. 0.093g *N*-(azetidin-3-yl)-3-(2-phenylacetamido)propanamide was obtained (quant.)

Step 3: The synthesis was performed according to the General Procedure 1 with *N*-(azetidin-3-yl)-3-(2-phenylacetamido)propanamide (0.093g, 0.357mmol) and cyanogen bromide (0.424mL, 1.27mmol).

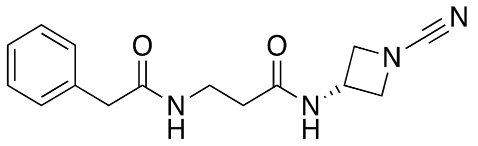

AF_11010_66 ^{1}H NMR (500 MHz, Methanol-d_4) δ 7.35 – 7.24 (m, 5H), 4.59 (tt, *J* = 7.8, 5.9 Hz, 1H), 4.37 (t, *J* = 7.9 Hz, 2H), 4.03 – 3.98 (m, 2H), 3.50 (s, 2H), 3.45 (t, *J* = 6.6 Hz, 2H), 2.41 (t, *J* = 6.6 Hz, 2H). LC/MS (ESI) *m/z* 286.97; $[M+H]^+$ calcd for $C_{15}H_{18}N_4O_2^+$: 287.14

Synthesis of AF_11010_82:

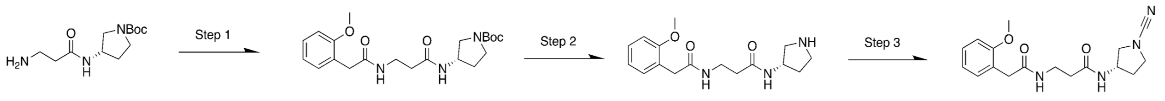

Step 1: The synthesis was performed according to the General Procedure 2-(2-methoxyphenyl)acetic acid (0.0485g, 0.291mmol) and *tert*-butyl (*S*)-3-(3-aminopropanamido)pyrrolidine-1-carboxylate (0.05g, 0.194mmol). 0.079g desired compound

tert-butyl (*S*)-3-(3-(2-(2-methoxyphenyl)acetamido)propanamido)pyrrolidine-1-carboxylate was obtained (99%).

Step 2: The synthesis was performed according to the General Procedure 1 with *tert*-butyl (*S*)-3-(3-(2-(2-methoxyphenyl)acetamido)propanamido)pyrrolidine-1-carboxylate (0.079g, 0.194mmol) except for using 4N HCl in 1,4-dioxane instead of TFA/DCM. 0.059g (*S*)-3-(2-(2-methoxyphenyl)acetamido)-*N*-(pyrrolidin-3-yl)propanamide was obtained (quant.)

Step 3: The synthesis was performed according to the General Procedure 1 (*S*)-3-(2-(2-methoxyphenyl)acetamido)-*N*-(pyrrolidin-3-yl)propanamide (0.059g, 0.194mmol) and cyanogen bromide (0.129mL, 0.388mmol).

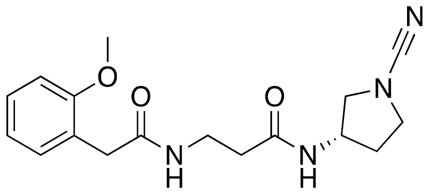

AF_11010_82 ^{1}H NMR (500 MHz, Methanol-d_4) δ 7.26 (td, *J* = 7.8, 1.7 Hz, 1H), 7.19 (dd, *J* = 7.4, 1.7 Hz, 1H), 6.96 (dd, *J* = 8.2, 1.1 Hz, 1H), 6.91 (td, *J* = 7.5, 1.1 Hz, 1H), 4.30 (ddt, *J* = 8.6, 6.1, 3.0 Hz, 1H), 3.83 (s, 3H), 3.58 (dd, *J* = 9.9, 6.0 Hz, 1H), 3.54 – 3.50 (m, 1H), 3.49 (s, 2H), 3.48 – 3.44 (m, 1H), 3.42 (t, *J* = 6.7 Hz, 2H), 3.19 (ddd, *J* = 9.9, 3.9, 0.9 Hz, 1H), 2.38 (t, *J* = 6.6 Hz, 2H), 2.13 (dtd, *J* = 13.1, 8.0, 6.2 Hz, 1H), 1.89 – 1.80 (m, 1H). LC/MS (ESI) *m/z* 330.97; $[M+H]^+$ calcd for $C_{17}H_{22}N_4O_3^+$: 331.17

Synthesis of AF_11010_83:

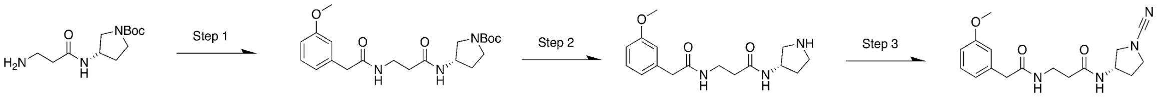

Step 1: The synthesis was performed according to the General Procedure 2-(3-methoxyphenyl)acetic acid (0.0485g, 0.291mmol) and *tert*-butyl (*S*)-3-(3-aminopropanamido)pyrrolidine-1-carboxylate (0.05g, 0.194mmol). 0.079g desired compound *tert*-butyl (*S*)-3-(3-(2-(3-methoxyphenyl)acetamido)propanamido)pyrrolidine-1-carboxylate was obtained (99%).

Step 2: The synthesis was performed according to the General Procedure 1 with *tert*-butyl (*S*)-3-(3-(2-(3-methoxyphenyl)acetamido)propanamido)pyrrolidine-1-carboxylate (0.079g, 0.194mmol) except for using 4N HCl in 1,4-dioxane instead of TFA/DCM. 0.059g (*S*)-3-(2-(3-methoxyphenyl)acetamido)-*N*-(pyrrolidin-3-yl)propanamide was obtained (quant.)

Step 3: The synthesis was performed according to the General Procedure 1 (*S*)-3-(2-(3-methoxyphenyl)acetamido)-*N*-(pyrrolidin-3-yl)propanamide (0.059g, 0.194mmol) and cyanogen bromide (0.129mL, 0.388mmol).

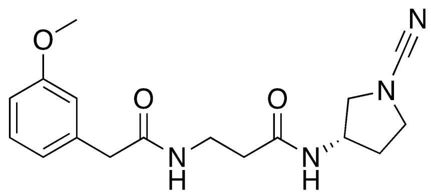

AF_11010_83 ^{1}H NMR (500 MHz, Methanol-d_4) δ 7.29 – 7.21 (m, 1H), 7.18 (dt, *J* = 7.4, 2.1 Hz, 1H), 6.96 (dd, *J* = 8.2, 1.2 Hz, 1H), 6.90 (tt, *J* = 7.5, 1.4 Hz, 1H), 4.30 (dd, *J* = 8.8, 4.3 Hz, 1H), 3.83 (d, *J* = 1.5 Hz, 3H), 3.58 (dd, *J* = 10.0, 6.0 Hz, 1H), 3.56 – 3.50 (m, 1H), 3.49 (d, *J* = 2.2 Hz, 2H), 3.48 – 3.44 (m, 1H), 3.42 (td, *J* = 6.7, 2.6 Hz, 2H), 3.22 – 3.14 (m, 1H), 2.38 (td, *J* = 6.6, 3.2 Hz, 2H), 2.19 – 2.06 (m, 1H), 1.89 – 1.79 (m, 1H). LC/MS (ESI) *m/z* 331.07; $[M+H]^+$ calcd for $C_{17}H_{22}N_4O_3^+$: 331.17

Synthesis of AF_11010_84:

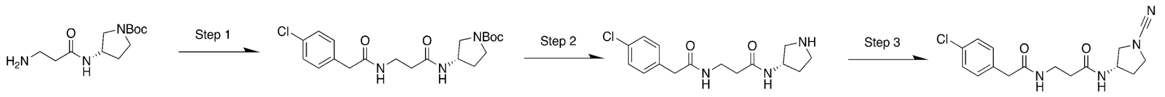

Step 1: The synthesis was performed according to the General Procedure 2-(4-chlorophenyl)acetic acid (0.0485g, 0.291mmol) and *tert*-butyl (*S*)-3-(3-aminopropanamido)pyrrolidine-1-carboxylate (0.05g, 0.194mmol). 0.079g desired compound *tert*-butyl (*S*)-3-(3-(2-(4-chlorophenyl)acetamido)propanamido)pyrrolidine-1-carboxylate was obtained (99%).

Step 2: The synthesis was performed according to the General Procedure 1 with *tert*-butyl (*S*)-3-(3-(2-(4-chlorophenyl)acetamido)propanamido)pyrrolidine-1-carboxylate (0.079g, 0.194mmol) except for using 4N HCl in 1,4-dioxane instead of TFA/DCM. 0.059g (*S*)-3-(2-(4-chlorophenyl)acetamido)-*N*-(pyrrolidin-3-yl)propanamide was obtained (quant.)

Step 3: The synthesis was performed according to the General Procedure 1 ((*S*)-3-(2-(4-chlorophenyl)acetamido)-*N*-(1-cyanopyrrolidin-3-yl)propanamide (0.059g, 0.194mmol) and cyanogen bromide (0.129mL, 0.388mmol).

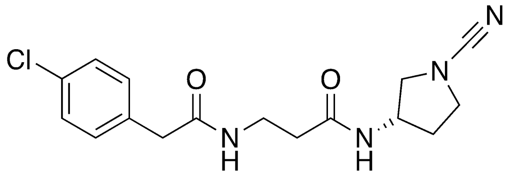

AF_11010_84 ^{1}H NMR (500 MHz, Methanol-d_4) δ 7.33 – 7.25 (m, 3H), 4.33 – 4.28 (m, 1H), 3.59 (dd, *J* = 9.9, 6.0 Hz, 1H), 3.54 – 3.48 (m, 1H), 3.48 – 3.45 (m, 2H), 3.44 (t, *J* = 6.6 Hz, 2H), 3.22 – 3.17 (m, 1H), 2.38 (t, *J* = 6.6 Hz, 2H), 2.18 – 2.09 (m, 1H), 1.84 (ddt, *J* = 12.6, 7.4, 4.9 Hz, 1H), 1.30 (s, 1H). LC/MS (ESI) *m/z* 334.97; [M+H]$^+$ calcd for $C_{16}H_{19}ClN_4O_2^+$: 335.12

Synthesis of AF_11010_124:

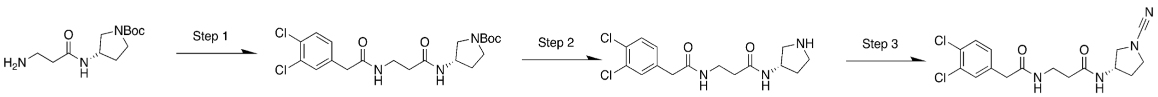

Step 1: The synthesis was performed according to the General Procedure 2-(3,4-dichlorophenyl)acetic acid (0.06g, 0.291mmol) and *tert*-butyl (*S*)-3-(3-aminopropanamido)pyrrolidine-1-carboxylate (0.05g, 1.0mmol). 0.059g desired compound *tert*-butyl (*S*)-3-(3-(2-(3,4-dichlorophenyl)acetamido)propanamido)pyrrolidine-1-carboxylate was obtained (68%).

Step 2: The synthesis was performed according to the General Procedure 1 with *tert*-butyl (*S*)-3-(3-(2-(3,4-dichlorophenyl)acetamido)propanamido)pyrrolidine-1-carboxylate (0.059g,

0.133mmol) except for using 4N HCl in 1,4-dioxane instead of TFA/DCM. 0.046g (*S*)-3-(2-(3,4-dichlorophenyl)acetamido)-*N*-(pyrrolidin-3-yl)propanamide was obtained (quant.)

Step 3: The synthesis was performed according to the General Procedure 1 (*S*)-3-(2-(3,4-dichlorophenyl)acetamido)-*N*-(pyrrolidin-3-yl)propanamide (0.045g, 0.133mmol) and cyanogen bromide (0.112mL, 0.33mmol).

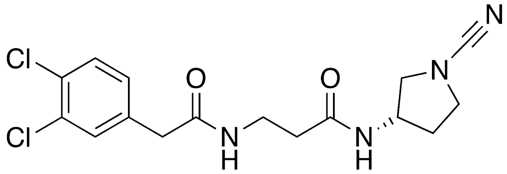

AF_11010_124 ^{1}H NMR (500 MHz, Methanol-d_4) δ 7.47 (d, *J* = 2.1 Hz, 1H), 7.46 (d, *J* = 8.2 Hz, 1H), 7.21 (dd, *J* = 8.3, 2.1 Hz, 1H), 4.31 (ddd, *J* = 10.3, 6.0, 4.3 Hz, 1H), 3.59 (dd, *J* = 9.9, 6.0 Hz, 1H), 3.54 – 3.49 (m, 1H), 3.48 (s, 2H), 3.44 (t, *J* = 6.6 Hz, 2H), 3.21 (dd, *J* = 9.9, 3.8 Hz, 1H), 2.39 (t, *J* = 6.6 Hz, 2H), 2.14 (dtd, *J* = 15.9, 7.9, 6.2 Hz, 1H), 1.84 (ddt, *J* = 12.6, 7.5, 4.9 Hz, 1H). ^{13}C NMR (126 MHz, DMSO) δ 170.86, 169.81, 138.04, 131.47, 131.10, 130.71, 129.93, 129.52, 117.75, 55.46, 49.24, 48.92, 41.47, 35.85, 35.54, 31.24. LC/MS (ESI) *m/z* 368.97; $[M+H]^+$ calcd for $C_{16}H_{18}Cl_2N_4O_2^+$: 369.08

Synthesis of AF_11010_125:

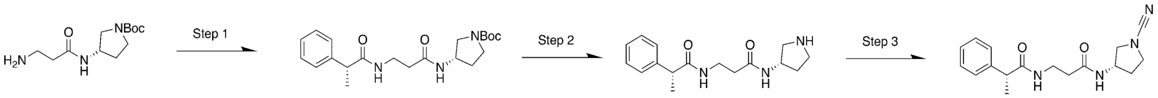

Step 1: The synthesis was performed according to the General Procedure (*R*)-2-phenylpropanoic acid (0.044g, 0.29mmol) and *tert*-butyl (*S*)-3-(3-aminopropanamido)pyrrolidine-1-carboxylate (0.050g, 0.194mmol). 0.052g desired compound *tert*-butyl (*S*)-3-(3-((*R*)-2-phenylpropanamido)propanamido)pyrrolidine-1-carboxylate was obtained (69%).

Step 2: The synthesis was performed according to the General Procedure 1 with *tert*-butyl (*S*)-3-(3-((*R*)-2-phenylpropanamido)propanamido)pyrrolidine-1-carboxylate (0.052g, 0.135mmol) except for using 4N HCl in 1,4-dioxane instead of TFA/DCM. 0.040g ((*R*)-*N*-(3-oxo-3-(((*S*)-pyrrolidin-3-yl)amino)propyl)-2-phenylpropanamide was obtained (quant.)

Step 3: The synthesis was performed according to the General Procedure 1 (*R*)-*N*-(3-oxo-3-(((*S*)-pyrrolidin-3-yl)amino)propyl)-2-phenylpropanamide (0.04g, 0.135mmol) and cyanogen bromide (0.112mL, 0.34mmol).

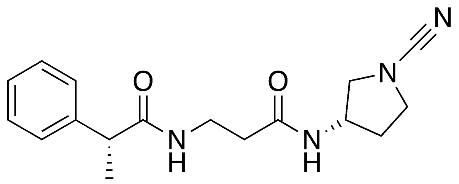

AF_11010_125 ^{1}H NMR (500 MHz, Methanol-*d*$_4$) δ 7.36 – 7.30 (m, 4H), 7.27 – 7.23 (m, 1H), 4.29 (ddd, *J* = 6.2, 4.5, 1.7 Hz, 1H), 3.63 (q, *J* = 7.1 Hz, 1H), 3.56 (dd, *J* = 9.9, 6.1 Hz, 1H), 3.53 – 3.44 (m, 3H), 3.40 (q, *J* = 6.8 Hz, 1H), 3.09 (ddd, *J* = 9.9, 4.1, 0.8 Hz, 1H), 2.38 (td, *J* = 6.7, 3.9 Hz, 2H), 2.18 – 2.09 (m, 1H), 1.88 – 1.80 (m, 1H), 1.45 (d, *J* = 7.0 Hz, 3H). LC/MS (ESI) *m/z* 314.87; $[M+H]^+$ calcd for $C_{17}H_{22}N_4O_2^+$: 315.17

Synthesis of AF_11010_131:

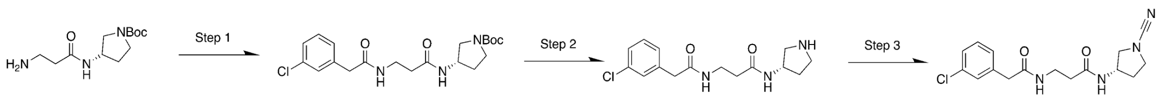

Step 1: The synthesis was performed according to the General Procedure 2-(3-chlorophenyl)acetic acid (0.044g, 0.291mmol) and *tert*-butyl (*S*)-3-(3-aminopropanamido)pyrrolidine-1-carboxylate (0.05g, 0.194mmol). 0.079g desired compound *tert*-butyl (*S*)-3-(3-(2-(3-chlorophenyl)acetamido)propanamido)pyrrolidine-1-carboxylate was obtained (99%).

Step 2: The synthesis was performed according to the General Procedure 1 with *tert*-butyl (*S*)-3-(3-(2-(3-chlorophenyl)acetamido)propanamido)pyrrolidine-1-carboxylate (0.079g, 0.194mmol) except for using 4N HCl in 1,4-dioxane instead of TFA/DCM. 0.06g (*S*)-3-(2-(3-chlorophenyl)acetamido)-*N*-(pyrrolidin-3-yl)propanamide was obtained (quant.)

Step 3: The synthesis was performed according to the General Procedure 1 ((*S*)-3-(2-(3-chlorophenyl)acetamido)-*N*-(1-cyanopyrrolidin-3-yl)propanamide (0.06g, 0.194mmol) and cyanogen bromide (0.162mL, 0.485mmol).

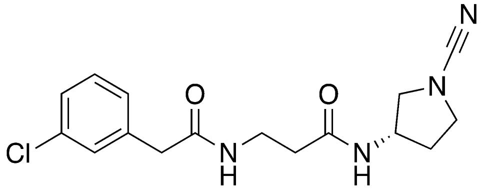

AF_11010_131 ^{1}H NMR (500 MHz, Methanol-d_4) δ 7.34 – 7.20 (m, 4H), 4.32 (tt, *J* = 6.1, 4.2 Hz, 1H), 3.59 (dd, *J* = 9.9, 6.0 Hz, 1H), 3.54 – 3.50 (m, 1H), 3.49 (s, 2H), 3.48 – 3.46 (m, 1H), 3.45 (t, *J* = 6.7 Hz, 2H), 3.20 (dd, *J* = 9.9, 3.9 Hz, 1H), 2.40 (t, *J* = 6.7 Hz, 2H), 2.19 – 2.09 (m, 1H), 1.90 – 1.81 (m, 1H). LC/MS (ESI) *m/z* 334.97; $[M+H]^+$ calcd for $C_{16}H_{19}ClN_4O_2^+$: 334.12

Synthesis of AF_11010_132:

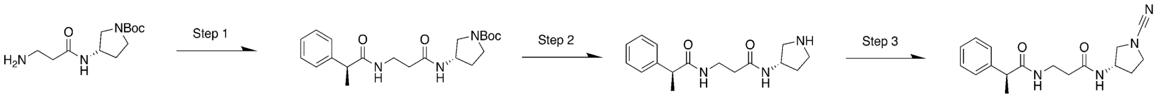

Step 1: The synthesis was performed according to the General Procedure (*S*)-2-phenylpropanoic acid (0.044g, 0.291mmol) and *tert*-butyl (*S*)-3-(3-aminopropanamido)pyrrolidine-1-carboxylate (0.05g, 0.194mmol). 0.075g desired compound *tert*-butyl (*S*)-3-(3-((*S*)-2-phenylpropanamido)propanamido)pyrrolidine-1-carboxylate was obtained (99%).

Step 2: The synthesis was performed according to the General Procedure 1 with *tert*-butyl (*S*)-3-(3-((*S*)-2-phenylpropanamido)propanamido)pyrrolidine-1-carboxylate (0.075g, 0.194mmol) except for using 4N HCl in 1,4-dioxane instead of TFA/DCM. 0.056g ((*S*)-*N*-(3-oxo-3-(((*S*)-pyrrolidin-3-yl)amino)propyl)-2-phenylpropanamide was obtained (quant.)

Step 3: The synthesis was performed according to the General Procedure 1 (*R*)-*N*-(3-oxo-3-(((*S*)-pyrrolidin-3-yl)amino)propyl)-2-phenylpropanamide (0.056g, 0.194mmol) and cyanogen bromide (0.162mL, 0.485mmol).

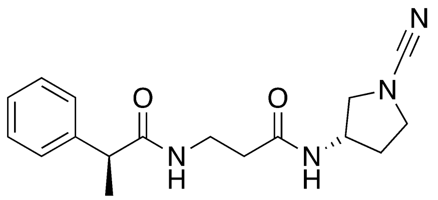

AF_11010_132 ^{1}H NMR (500 MHz, Methanol-d_4) δ 7.35 – 7.26 (m, 4H), 7.22 (dddd, *J* = 6.9, 5.7, 3.3, 1.4 Hz, 1H), 4.26 (ddd, *J* = 10.4, 6.1, 4.3 Hz, 1H), 3.63 – 3.53 (m, 2H), 3.49 – 3.34 (m, 4H), 3.17 (ddd, *J* = 9.9, 3.8, 0.9 Hz, 1H), 2.39 – 2.33 (m, 2H), 2.14 – 2.02 (m, 1H), 1.83 – 1.73 (m, 1H), 1.42 (dd, *J* = 7.2, 1.5 Hz, 3H). LC/MS (ESI) *m/z* 314.97; [M+H]$^+$ calcd for $C_{17}H_{22}N_4O_2^+$: 315.17

Synthesis of AF_11010_104:

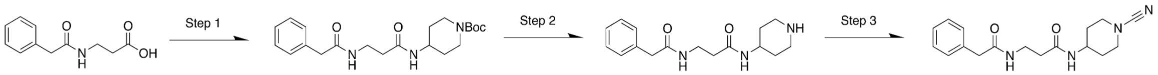

Step 1: The synthesis was performed according to the General Procedure 1 with 3-(2-phenylacetamido)propanoic acid (0.075g, 0.362mmol) and *tert*-butyl 4-aminopiperidine-1-carboxylate (0.06g, 0.3mmol). 0.116g desired compound *tert*-butyl 4-(3-(2-phenylacetamido)propanamido)piperidine-1-carboxylate was obtained (99%).

Step 2: The synthesis was performed according to the General Procedure 1 with *tert*-butyl 4-(3-(2-phenylacetamido)propanamido)piperidine-1-carboxylate (0.116g, 0.3mmol) except for using 4N HCl in 1,4-dioxane instead of TFA/DCM. 0.087g 3-(2-phenylacetamido)-*N*-(piperidin-4-yl)propanamide was obtained (quant.)

Step 3: The synthesis was performed according to the General Procedure 1 with 3-(2-phenylacetamido)-*N*-(piperidin-4-yl)propanamide (0.087g, 0.3mmol) and cyanogen bromide (0.251mL, 0.75mmol).

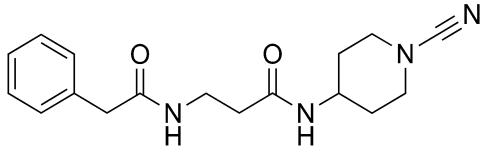

AF_11010_104 ^{1}H NMR (500 MHz, Methanol-d_4) δ 7.33 – 7.22 (m, 5H), 3.74 (tt, *J* = 10.8, 4.0 Hz, 1H), 3.48 (s, 2H), 3.43 (t, *J* = 6.7 Hz, 2H), 3.39 (td, *J* = 5.3, 4.4, 3.1 Hz, 2H), 3.13 (ddd, *J* = 13.2, 11.6, 2.8 Hz, 2H), 2.36 (t, *J* = 6.7 Hz, 2H), 1.86 – 1.79 (m, 2H), 1.55 – 1.44 (m, 2H). LC/MS (ESI) *m/z* 314.77; $[M+H]^+$ calcd for $C_{17}H_{22}N_4O_2^+$: 315.17

Synthesis of AF_11010_105:

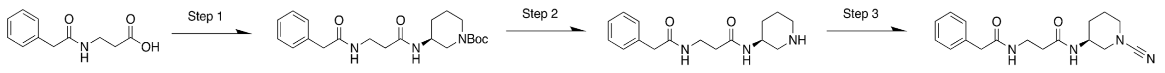

Step 1: The synthesis was performed according to the General Procedure 1 with 3-(2-phenylacetamido)propanoic acid (0.15g, 0.724mmol) and *tert*-butyl (*S*)-3-aminopiperidine-1-carboxylate (0.125g, 0.626mmol). 0.243g desired compound *tert*-butyl (*S*)-3-(3-(2-phenylacetamido)propanamido)piperidine-1-carboxylate was obtained (99%).

Step 2: The synthesis was performed according to the General Procedure 1 with *tert*-butyl (*S*)-3-(3-(2-phenylacetamido)propanamido)piperidine-1-carboxylate (0.233, 0.6mmol) except for using 4N HCl in 1,4-dioxane instead of TFA/DCM. 0.173g (*S*)-3-(2-phenylacetamido)-*N*-(piperidin-3-yl)propanamide was obtained (quant.)

Step 3: The synthesis was performed according to the General Procedure 1 with (*S*)-3-(2-phenylacetamido)-*N*-(piperidin-3-yl)propanamide (0.087g, 0.3mmol) and cyanogen bromide (0.25mL, 0.75mmol).

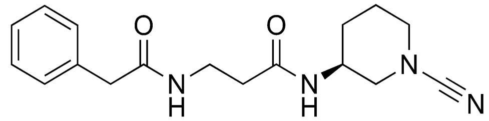

AF_11010_105 ^{1}H NMR (500 MHz, Methanol-d_4) δ 7.35 – 7.21 (m, 5H), 3.83 (ddd, *J* = 9.0, 5.5, 4.0 Hz, 1H), 3.48 (s, 2H), 3.43 (td, *J* = 6.6, 3.2 Hz, 2H), 3.35 (dd, *J* = 12.4, 4.2 Hz, 1H), 3.30 – 3.24 (m, 1H), 3.09 – 3.03 (m, 1H), 2.79 (dd, *J* = 12.4, 8.6 Hz, 1H), 2.39 (td, *J* = 6.7, 1.1 Hz, 2H), 1.86 – 1.78 (m, 2H), 1.71 – 1.61 (m, 1H), 1.44 – 1.35 (m, 1H). LC/MS (ESI) *m/z* 314.67; $[M+H]^+$ calcd for $C_{17}H_{22}N_4O_2^+$: 315.17

Synthesis of AF_11010_136

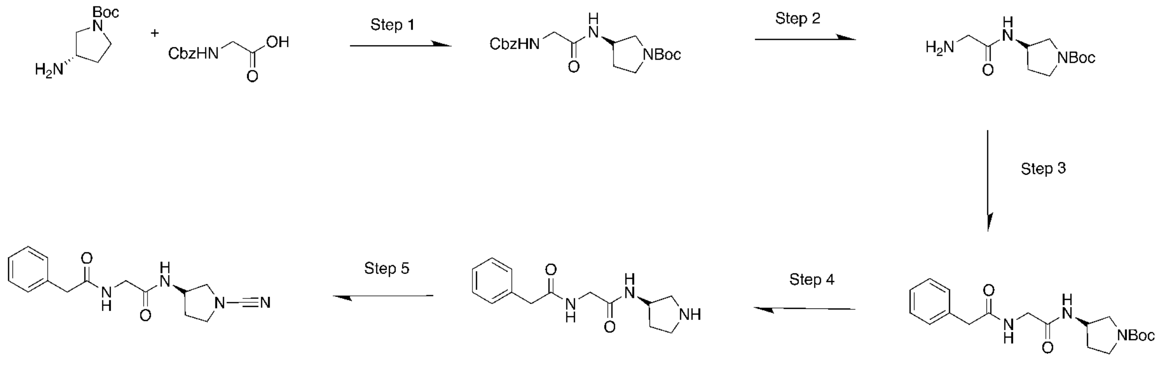

Step 1: The synthesis was preformed according to General Procedure 1 with ((benzyloxy)carbonyl)glycine (0.075g, .36mmol) and *tert*-butyl (*S*)-3-aminopyrrolidine-1-carboxylate (0.055mL, 0.3mmol). 0.103g desired compound *tert*-butyl (*S*)-3-(2-(((benzyloxy)carbonyl)amino)acetamido)pyrrolidine-1-carboxylate was obtained (91%).

Step 2: The synthesis was performed according to the General Procedure 1 with *tert*-butyl (*S*)-3-(2-(((benzyloxy)carbonyl)amino)acetamido)pyrrolidine-1-carboxylate (0.103g, 0.27mmol) except for using palladium on carbon and MeOH instead of TFA/DCM. 0.065g *tert*-butyl (*S*)-3-(2-aminoacetamido)pyrrolidine-1-carboxylate was obtained (quant.)

Step 3: The synthesis was performed according to the General Procedure 1 *tert*-butyl (*S*)-3-(2-aminoacetamido)pyrrolidine-1-carboxylate (0.065g, 0.27mmol) and *2-phenyl acetic acid* (0.043g, 0.32mmol). 0.90g desired compound *tert*-butyl (*S*)-3-(2-(2-phenylacetamido)acetamido)pyrrolidine-1-carboxylate was obtained (92%).

Step 4: The synthesis was performed according to the General Procedure 1 with *S*)-3-(2-(2-phenylacetamido)acetamido)pyrrolidine-1-carboxylate (0.09g, 0.25mmol) except for using 4N HCl in 1,4-dioxane instead of TFA/DCM. 0.065g (*S*)-*N*-(2-oxo-2-(pyrrolidin-3-ylamino)ethyl)-2-phenylacetamide was obtained (quant.)

Step 5: The synthesis was performed according to the General Procedure 1 with (*S*)-*N*-(2-oxo-2-(pyrrolidin-3-ylamino)ethyl)-2-phenylacetamide (0.065g, 0.25mmol) and cyanogen bromide (0.250mL, 0.75mmol).

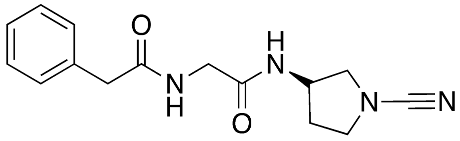

AF_11010_136 ^{1}H NMR (500 MHz, Methanol-d_4) δ 7.35 – 7.32 (m, 3H), 7.29 – 7.24 (m, 1H), 4.38 (tt, *J* = 6.1, 4.2 Hz, 1H), 3.84 (d, *J* = 2.5 Hz, 2H), 3.63 (dd, *J* = 9.9, 6.0 Hz, 1H), 3.60 (s, 2H), 3.55 – 3.46 (m, 2H), 3.26 (ddd, *J* = 10.1, 3.9, 0.9 Hz, 1H), 2.17 (dtd, *J* = 13.0, 8.0, 6.2 Hz, 1H), 1.94 – 1.87 (m, 1H). LC/MS (ESI) *m/z* 286.97; $[M+H]^+$ calcd for $C_{15}H_{18}N_4O_2^+$: 287.14

<u>Synthesis of AF_11010_137</u>

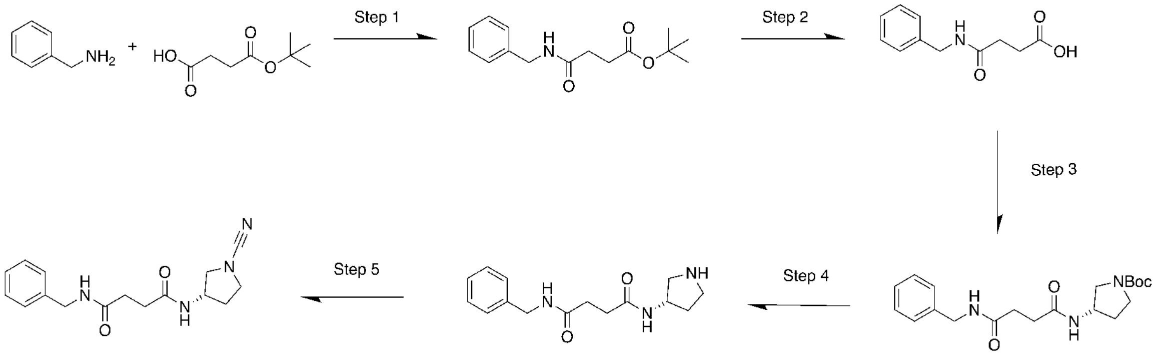

Step 1: The synthesis was preformed according to General Procedure 1 with phenylmethanamine (0.031mL, 0.287mmol) and 4-(*tert*-butoxy)-4-oxobutanoic acid (0.075g, 0.43mmol). 0.105g desired compound *tert*-butyl 4-(benzylamino)-4-oxobutanoate was obtained (93%).

Step 2: The synthesis was performed according to the General Procedure 1 with (*tert*-butyl 4-(benzylamino)-4-oxobutanoate (0.105g, 0.4mmol) except for using 4N HCl in 1,4-dioxane instead of TFA/DCM. 0.83g 4-(benzylamino)-4-oxobutanoic acid was obtained (quant.)

Step 3: The synthesis was performed according to the General Procedure 1 4-(benzylamino)-4-oxobutanoic acid (0.083g, 0.4mmol) and *tert*-butyl (*S*)-3-(3-aminopropanamido)pyrrolidine-1-carboxylate (0.088mL, 0.48mmol). 0.144g desired compound *tert*-butyl (*S*)-3-(4-(benzylamino)-4-oxobutanamido)pyrrolidine-1-carboxylate was obtained (99%).

Step 4: The synthesis was performed according to the General Procedure 1 with *tert*-butyl (*S*)-3-(4-(benzylamino)-4-oxobutanamido)pyrrolidine-1-carboxylate (0.144g, 0.4mmol) except for

using 4N HCl in 1,4-dioxane instead of TFA/DCM. 0.11g (*S*)-*N*[1]-benzyl-*N*[4]-(pyrrolidin-3-yl)succinamide was obtained (quant.)

Step 5: The synthesis was performed according to the General Procedure 1 with *S*)-*N*[1]-benzyl-*N*[4]-(pyrrolidin-3-yl)succinamide (0.11g, 0.4mmol) and cyanogen bromide (0.33mL, 1.0mmol).

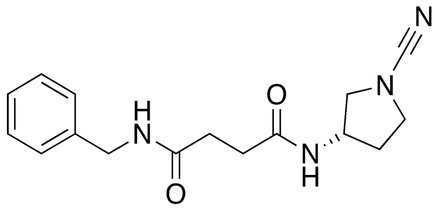

AF_11010_137 ^{1}H NMR (500 MHz, Methanol-d_4) δ 7.35 – 7.23 (m, 5H), 4.37 (s, 2H), 4.34 (dq, *J* = 6.0, 4.2, 3.1 Hz, 1H), 3.61 (dd, *J* = 9.9, 6.0 Hz, 1H), 3.58 – 3.46 (m, 2H), 3.24 (dd, *J* = 9.9, 3.9 Hz, 1H), 2.58 – 2.49 (m, 4H), 2.15 (dtd, *J* = 13.1, 8.0, 6.2 Hz, 1H), 1.90 (ddt, *J* = 12.6, 7.3, 4.8 Hz, 1H). LC/MS (ESI) *m/z* 300.97; $[M+H]^+$ calcd for $C_{16}H_{20}N_4O_2^+$: 301.16

Synthesis of AF_11010_139

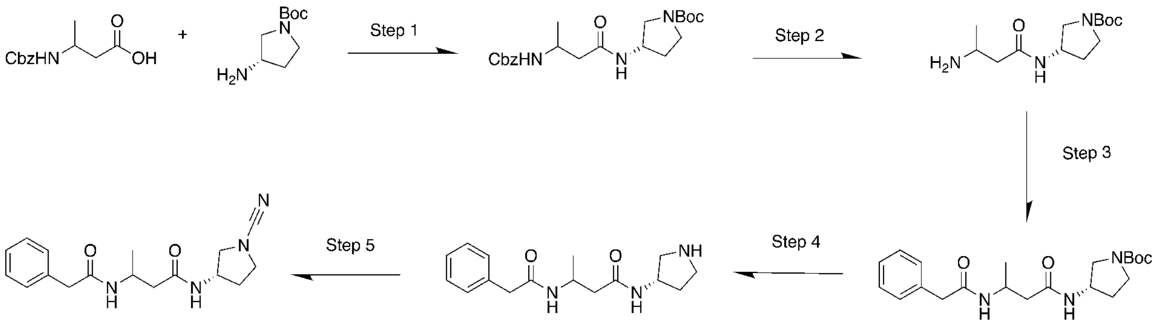

Step 1: The synthesis was preformed according to General Procedure 1 with 3-(((benzyloxy)carbonyl)amino)butanoic acid (0.072g, 0.3mmol) and *tert*-butyl (*S*)-3-aminopyrrolidine-1-carboxylate (0.046mL, 0.25mmol). 0.094g desired compound *tert*-butyl

(3*S*)-3-(3-(((benzyloxy)carbonyl)amino)butanamido)pyrrolidine-1-carboxylate was obtained (93%).

Step 2: The synthesis was performed according to the General Procedure 1 with *tert*-butyl (3*S*)-3-(3-(((benzyloxy)carbonyl)amino)butanamido)pyrrolidine-1-carboxylate (0.094g, 0.233mmol) except for using palladium on carbon and MeOH instead of TFA/DCM. 0.63g *tert*-butyl (3*S*)-3-(3-aminobutanamido)pyrrolidine-1-carboxylate was obtained (quant.)

Step 3: The synthesis was performed according to the General Procedure 1 3-(2-phenylacetamido)butanoic acid (0.038g, 0.28mmol) and *tert*-butyl (3*S*)-3-(3-aminobutanamido)pyrrolidine-1-carboxylate (0.063g, 0.233mmol). 0.09 desired compound *tert*-butyl (3*S*)-3-(3-(2-phenylacetamido)butanamido)pyrrolidine-1-carboxylate was obtained (98%).

Step 4: The synthesis was performed according to the General Procedure 1 with *tert*-butyl (3*S*)-3-(3-(2-phenylacetamido)butanamido)pyrrolidine-1-carboxylate (0.09g, 0.23mmol) except for using 4N HCl in 1,4-dioxane instead of TFA/DCM. 0.067g 3-(2-phenylacetamido)-*N*-((*S*)-pyrrolidin-3-yl)butanamide was obtained (quant.)

Step 5: The synthesis was performed according to the General Procedure 1 3-(2-phenylacetamido)-*N*-((*S*)-pyrrolidin-3-yl)butanamide (0.067g, 0.23mmol) and cyanogen bromide (0.192mL, 0.575mmol).

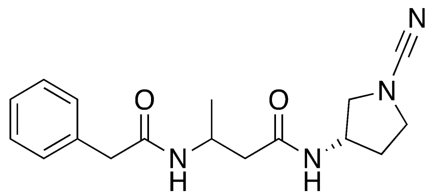

AF_11010_139 ^{1}H NMR (500 MHz, Methanol-*d*$_4$) δ 7.34 – 7.23 (m, 5H), 4.27 (qd, *J* = 6.4, 2.5 Hz, 2H), 3.57 (dd, *J* = 9.9, 6.0 Hz, 1H), 3.55 – 3.49 (m, 1H), 3.48 (d, *J* = 2.4 Hz, 2H), 3.45 (tdd, *J* = 9.3, 4.9, 3.3 Hz, 1H), 3.19 – 3.11 (m, 1H), 1.20 (d, *J* = 6.7 Hz, 3H). LC/MS (ESI) *m/z* 314.97; $[M+H]^+$ calcd for $C_{17}H_{22}N_4O_2^+$: 315.17

<u>Synthesis of CAS 11487 188, 193, 195, 199, 200, 205:</u>

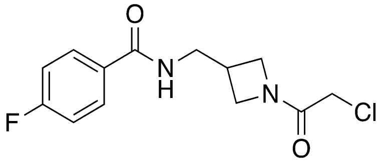

Synthesis of **CAS-11487-188** followed the General Procedure 1 by using 4-fluorobenzoic acid (0.075 g, 0.535 mmol), and *tert*-butyl 3-(aminomethyl)azetidine-1-carboxylate (0.098 mL, 0.535 mmol) to afford CAS-11487-188 (0.0353g, 51.7%) ^{1}H NMR (500 MHz, DMSO-*d*6) δ 8.67 (t, *J* = 5.7 Hz, 1H), 7.99 – 7.81 (m, 2H), 7.42 – 7.18 (m, 2H), 4.31 – 4.17 (m, 1H), 4.09 (d, *J* = 1.4 Hz, 2H), 4.01 – 3.89 (m, 2H), 3.69 (dd, *J* = 9.9, 5.5 Hz, 1H), 3.51 – 3.37 (m, 2H), 2.91 – 2.71 (m, 1H). LC/MS (ESI) *m/z* 285.67; $[M+H]^+$ calcd for $C_{13}H_{15}ClFN_2O_2^+$ 285.08

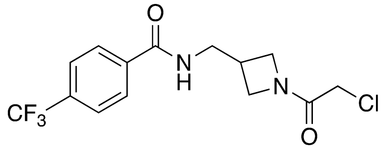

Synthesis of **CAS-11487-193** followed the General Procedure 1 by using 4-(trifluoromethyl)benzoic acid (0.200 g, 1.05 mmol), and *tert*-butyl 3-(aminomethyl)azetidine-1-carboxylate (0.192 mL, 1.05 mmol) to afford CAS-11487-193 (0.0128g, 11.7%) ^{1}H NMR (500 MHz, Chloroform-*d*) δ 7.94 (d, *J* = 8.1 Hz, 2H), 7.72 (d, *J* = 8.2 Hz, 2H), 7.07 (t, *J* = 6.0 Hz, 1H), 4.42 (t, *J* = 8.7 Hz, 1H), 4.20 – 4.08 (m, 2H), 3.96 – 3.91 (m, 1H), 3.89 (d, *J* = 2.5 Hz, 2H), 3.80 (dt, *J* = 14.0, 6.3 Hz, 1H), 3.68 (dt, *J* = 13.7, 6.5 Hz, 1H), 3.04 (dtdd, *J* = 8.3, 6.6, 4.9, 1.5 Hz, 1H). LC/MS (ESI) *m/z* 335.07; $[M+H]^+$ calcd for $C_{14}H_{15}ClF_3N_2O_2^+$: 335.08

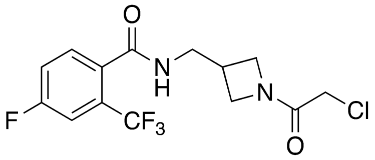

Synthesis of **CAS-11487-195** followed the General Procedure 1 by using 4-fluoro-2-(trifluoromethyl)benzoic acid (0.250 g, 1.20 mmol), and *tert*-butyl 3-(aminomethyl)azetidine-1-carboxylate (0.440 mL, 2.40 mmol) to afford CAS-11487-195 (0.0266 g, 12.6%) ^{1}H NMR (500 MHz, Chloroform-*d*) δ 7.57 (dd, *J* = 8.5, 5.2 Hz, 1H), 7.43 (dd, *J* = 8.8, 2.6 Hz, 1H), 7.32 (td, *J* = 8.1, 2.6 Hz, 1H), 6.37 (s, 1H), 4.41 (t, *J* = 8.8 Hz, 1H), 4.17 (t, *J* = 9.5 Hz, 1H), 4.10 (dd, *J* = 9.2, 5.4 Hz, 1H), 3.88 (s, 2H), 3.84 (dd, *J* = 10.6, 5.6 Hz, 1H), 3.79 – 3.60 (m, 2H), 3.08 – 2.98 (m, 1H). LC/MS (ESI) *m/z* 353.06; $[M+H]^+$ calcd for $C_{14}H_{14}ClF_4N_2O_2^+$: 353.07

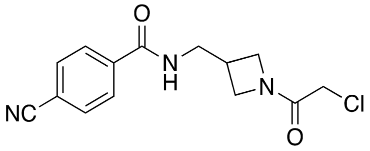

Synthesis of **CAS-11487-199** followed the General Procedure 1 by using 4-cyanobenzoic acid (1.0 g, 6.80 mmol), and *tert*-butyl 3-(aminomethyl)azetidine-1-carboxylate (1.4 mL, 7.48 mmol) to afford CAS-11487-199 (0.011 g, 11.5%) ^{1}H NMR (500 MHz, Chloroform-*d*) δ 7.97 – 7.88 (m, 2H), 7.81 – 7.73 (m, 2H), 6.82 (s, 0H), 4.43 (t, *J* = 8.7 Hz, 1H), 4.23 – 4.15 (m, 1H), 4.12 (dd, *J* = 9.2, 5.2 Hz, 1H), 3.96 – 3.90 (m, 1H), 3.90 (d, *J* = 2.0 Hz, 2H), 3.81 (dt, *J* = 13.1, 6.4 Hz, 1H), 3.69 (dt, *J* = 13.6, 6.4 Hz, 1H), 3.10 – 2.98 (m, 1H). LC/MS (ESI) *m/z* 292.38; [M+H]$^+$ calcd for $C_{14}H_{15}ClN_3O_2^+$: 292.08

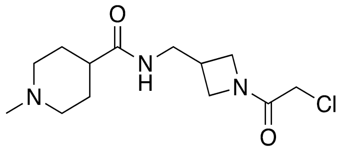

Synthesis of **CAS-11487-200** followed the General Procedure 1 by using 1-methylpiperidine-4-carboxylic acid (0.300 g, 2.10 mmol), and *tert*-butyl 3-(aminomethyl)azetidine-1-carboxylate (0.421 mL, 2.30 mmol) to afford CAS-11487-200 (0.0266 g, 12.6%). ^{1}H NMR (500 MHz, Methanol-*d*$_4$) δ 4.37 (t, *J* = 8.8 Hz, 1H), 4.13 – 4.07 (m, 1H), 4.07 – 3.98 (m, 4H), 3.76 (dd, *J* = 10.4, 5.5 Hz, 1H), 3.62 – 3.54 (m, 2H), 3.51 – 3.37 (m, 3H), 3.03 (td, *J* = 13.0, 3.1 Hz, 2H), 2.88 (s, 5H), 2.53 (tt, *J* = 12.1, 3.8 Hz, 1H), 2.15 (d, *J* = 16.0 Hz, 1H), 2.10 – 2.01 (m, 2H), 2.01 – 1.88 (m, 2H). LC/MS (ESI) *m/z* 288.07; [M+H]$^+$ calcd for $C_{13}H_{23}ClN_3O_2^+$: 288.15

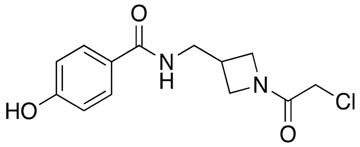

Synthesis of **CAS-11487-205** followed the General Procedure 1 by using 4-hydroxybenzoic acid (0.200 g, 1.45 mmol), and *tert*-butyl 3-(aminomethyl)azetidine-1-carboxylate (0.291 mL, 1.59 mmol) to afford CAS-11487-205 (0.001 g, 1.4%) ^{1}H NMR (500 MHz, Methanol-*d*$_4$) δ 7.76 – 7.65 (m, 2H), 7.66 – 7.51 (m, 0H), 7.05 – 6.93 (m, 0H), 6.90 – 6.78 (m, 2H), 4.46 – 4.34 (m, 1H), 4.19 – 4.06 (m, 2H), 4.02 (d, *J* = 1.1 Hz, 2H), 3.91 – 3.81 (m, 1H), 3.67 – 3.55 (m, 2H), 3.00 (dddd, *J* = 12.3, 8.4, 5.0, 2.2 Hz, 1H). LC/MS (ESI) *m/z* 283.15; [M+H]$^+$ calcd for $C_{13}H_{16}ClN_2O_3^+$: 283.08

Synthesis of CAS 12290 001

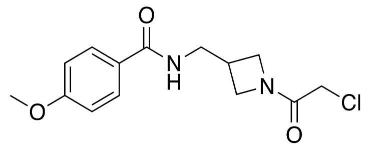

Synthesis of **CAS-12290-001** followed the General Procedure 1 by using 4-methoxybenzoic acid (0.250 g, 1.64 mmol), and *tert*-butyl 3-(aminomethyl)azetidine-1-carboxylate (0.330 mL, 1.81 mmol) to afford CAS-12290-001 (0.060 g, 24.8%) ^{1}H NMR (500 MHz, Chloroform-*d*) δ 7.83 – 7.63 (m, 2H), 7.18 (t, *J* = 6.1 Hz, 1H), 7.00 – 6.80 (m, 2H), 4.36 (t, *J* = 8.7 Hz, 1H), 4.18 – 4.04 (m, 2H), 3.89 (q, *J* = 5.4, 5.0 Hz, 1H), 3.86 (d, *J* = 7.5 Hz, 5H), 3.72 (dt, *J* = 13.1, 6.4 Hz, 1H), 3.62 (dt, *J* = 13.6, 6.4 Hz, 1H), 3.05 – 2.94 (m, 1H). LC/MS (ESI) *m/z* 297.18; $[M+H]^+$ calcd for $C_{14}H_{18}ClN_2O_3^+$: 297.10

Syntheses of CAS 12290 010, and 011

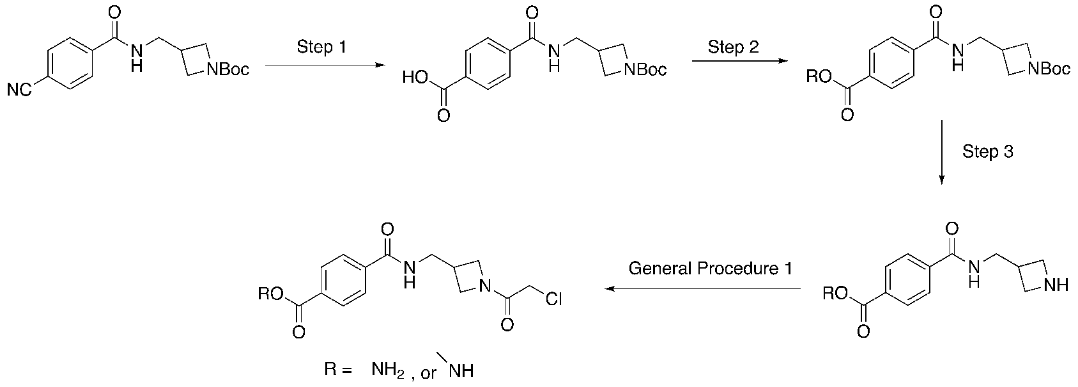

Step 1: Nitrile *tert*-butyl 3-((4-cyanobenzamido)methyl)azetidine-1-carboxylate (0.350 g, 1.10 mmol) was dissolved in 80% EtOH (8 mL), KOH (0.311 g, 5.55 mmol) was added and the reaction was heated to reflux for 16h. Crude was purified directly by flash chromatography using eluent gradient 0-40% MeOH/EtOAc. 140 mg of desired product 4-(((1-(*tert*-butoxycarbonyl)azetidin-3-yl)methyl)carbamoyl)benzoic acid was obtained (38%).

Step 2: 4-(((1-(*tert*-butoxycarbonyl)azetidin-3-yl)methyl)carbamoyl)benzoic acid (0.0703 g, .210 mmol) was dissolved in DCM (1-2 mL) with diisopropylethylamine (0.074 mL, 0.4205 mmol), and HATU (0.094 g, 0.2522 mmol) at room temperature. Ammonium chloride (0.045 g, 0.8409 mmol), or 2M Methylamine (0.030 mL, 0.6307 mmol) were added. The mixture then stirred at room temperature overnight, and was directly purified by flash chromatography 10% MeOH/ EtOAc. 34 mg of desired product, *tert*-butyl 3-((4-carbamoylbenzamido)methyl)azetidine-1-carboxylate, and 39 mg of desired product *tert*-butyl 3-((4-

(methylcarbamoyl)benzamido)methyl)azetidine-1-carboxylate were obtained (49%, 53%, respectively).

Step 3: Products from the last step were dissolved in DCM (1-2 mL) at room temperature and treated with TFA (1 mL). The mixtures stirred at room temperature until the tert-butyloxycarbonyl protecting group was cleaved tracking by UPLC-MS. The mixtures were concentrated and placed *in vacuo* for 12 hours.

Step 4: Acylations using chloroacetyl chloride were performed according to Step 3 of General Procedure 1 to yield the target compounds **CAS-12290-010** (5.65%), and **CAS-12290-011** (5.7%).

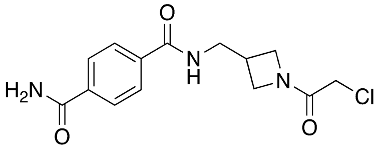

CAS-12290-010 ^{1}H NMR (500 MHz, Methanol-*d*$_4$) δ 8.82 (s, 1H), 7.99 – 7.95 (m, 1H), 7.93 – 7.89 (m, 1H), 4.42 (t, *J* = 8.8 Hz, 1H), 4.16 (d, *J* = 9.5 Hz, 1H), 4.13 (dd, *J* = 8.3, 4.6 Hz, 1H), 4.03 (d, *J* = 2.5 Hz, 2H), 3.87 (dd, *J* = 10.3, 5.5 Hz, 1H), 3.71 – 3.63 (m, 2H), 3.05 – 3.00 (m, 1H). LC/MS (ESI) *m/z* 310.07; [M+H]$^+$ calcd for $C_{14}H_{17}ClN_3O_3^+$: 310.10

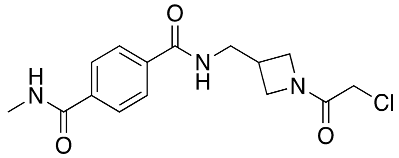

Synthesis of **CAS-12290-011** ^{1}H NMR (500 MHz, Methanol-*d*$_4$) δ 8.81 (s, 1H), 7.91 (s, 4H), 4.43 (d, *J* = 8.8 Hz, 1H), 4.17 – 4.11 (m, 2H), 4.03 (d, *J* = 2.6 Hz, 2H), 3.87 (dd, *J* = 10.3, 5.5 Hz, 2H), 3.66 (dd, *J* = 7.0, 3.2 Hz, 2H), 3.03 (d, *J* = 7.1 Hz, 1H), 2.95 (s, 3H), 2.68 (s, 2H). LC/MS (ESI) *m/z* 324.17; [M+H]$^+$ calcd for $C_{15}H_{19}ClN_3O_3^+$: 324.11

<u>Syntheses of CAS-12290 024, 039, 073, 076, and 077</u>

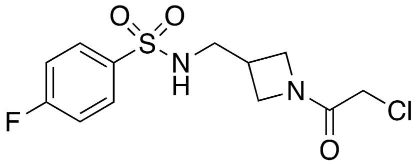

Synthesis of **CAS-12290-024** was performed by combining 4-fluorobenzenesulfonyl chloride (.200 g, 1.03 mmol), *tert*-butyl 3-(aminomethyl)azetidine-1-carboxylate (0.181 mL, 1.03 mmol), and K_2CO_3 (0.285 g, 2.06 mmol) in DCM at rt and was allowed to react overnight. Crude was then directly purified by flash chromatography 80% EtOAc / Hexanes then conc. *in vacuo* to

afford *tert*-butyl 3-(((4-fluorophenyl)sulfonamido)methyl)azetidine-1-carboxylate (92.2 %). Subsequent removal of the Boc protecting group and acylation using chloroacetyl chloride were executed following Step(s) 2, and 3 of General Procedure 1 to afford **CAS-12290-024** (2.5 %). ^{1}H NMR (500 MHz, DMSO-d_6) δ 7.96 – 7.78 (m, 1H), 7.52 – 7.40 (m, 1H), 6.57 (s, 1H), 4.16 (t, *J* = 8.6 Hz, 1H), 4.07 (s, 1H), 3.90 – 3.76 (m, 1H), 3.55 (dd, *J* = 9.9, 5.4 Hz, 1H), 3.03 – 2.90 (m, 1H), 2.67 (dddd, *J* = 13.8, 6.7, 4.2, 1.5 Hz, 1H). LC/MS (ESI) *m/z* 321.04; $[M+23]^+$ calcd for $C_{12}H_{15}ClFN_2O_3S^+$: 321.05

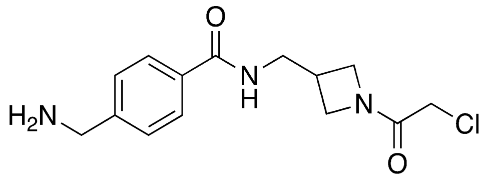

Synthesis of **CAS-12290-039** was executed by combining CAS-11487-199 (0.0263 g, 0.0902 mmol), and $NiCl_2 \cdot 6H_2O$ (0.02145 g, 0.0902 mmol) in 15:1 EtOH/DCM (1 mL) and placed on ice. $NaBH_4$ (0.0102 g, 0.2708 mmol) was added slowly, and then the mixture was warmed to room temperature and allowed to react for 2h. The crude was purified by flash chromatography (hexanes/EtOAc/MeOH) and preparative HPLC (MeOH/H_2O w/ 0.0425% TFA) to afford the target molecule (5.6 %). ^{1}H NMR (500 MHz, Methanol-d_4) δ 10.09 (s, 1H), 8.05 – 7.98 (m, 2H), 7.86 – 7.81 (m, 1H), 7.57 (dd, *J* = 18.7, 8.1 Hz, 2H), 4.45 – 4.38 (m, 2H), 4.15 (ddd, *J* = 14.2, 9.3, 5.2 Hz, 3H), 4.03 (t, *J* = 2.5 Hz, 2H), 3.87 (dt, *J* = 9.9, 4.6 Hz, 2H), 3.69 – 3.62 (m, 3H), 3.03 (q, *J* = 6.5, 5.9 Hz, 2H). LC/MS (ESI) *m/z* 296.32; $[M+H]^+$ calcd for $C_{14}H_{19}ClN_3O_2^+$: 296.12

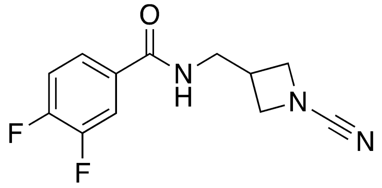

Synthesis of **CAS-12290-073** followed the General Procedure 1 by using 3,4-difluorobenzoic acid (0.500 g, 3.16 mmol), and *tert*-butyl 3-(aminomethyl)azetidine-1-carboxylate (0.636 mL, 3.48 mmol) to afford CAS-12290-073 (0.0062 g, 11.6%) ^{1}H NMR (500 MHz, Chloroform-*d*) δ 7.67 (ddd, *J* = 10.6, 7.5, 2.2 Hz, 1H), 7.54 (dddd, *J* = 8.5, 3.9, 2.2, 1.4 Hz, 1H), 7.28 – 7.22 (m, 1H), 6.46 (s, 1H), 4.30 (t, *J* = 8.0 Hz, 2H), 3.98 (dd, *J* = 7.8, 5.6 Hz, 2H), 3.69 (t, *J* = 6.4 Hz, 2H), 3.05 (ttt, *J* = 8.2, 6.7, 5.6 Hz, 1H). LC/MS (ESI) *m/z* 252.19; $[M+H]^+$ calcd for $C_{12}H_{12}F_2N_3O^+$: 252.09

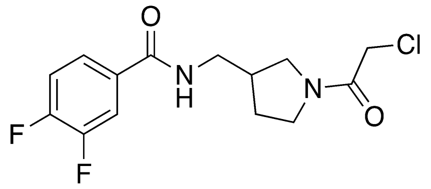

Synthesis of **CAS-12290-076** followed the General Procedure 1 by using 3,4-difluorobenzoic acid (0.150 g, 0.9487 mmol), and *tert*-butyl 3-(aminomethyl)pyrrolidine-1-carboxylate (0.200 mL, 1.04 mmol) to afford CAS-12290-076 (0.0151 g, 14.8%) ^{1}H NMR (500 MHz, Chloroform-*d*) δ 7.71 (dddd, *J* = 16.4, 10.7, 7.5, 2.2 Hz, 1H), 7.64 – 7.51 (m, 1H), 7.28 – 7.20 (m, 1H), 6.83 (d, *J* = 141.9 Hz, 1H), 4.08 – 4.03 (m, 3H), 3.71 (pd, *J* = 6.0, 4.5, 1.7 Hz, 3H), 3.67 – 3.54 (m, 1H), 3.51 (ddd, *J* = 8.9, 5.5, 3.3 Hz, 1H), 3.47 – 3.28 (m, 2H), 2.77 – 2.60 (m, 1H), 2.26 – 2.08 (m, 1H), 1.83 (ddq, *J* = 55.3, 12.8, 7.7 Hz, 1H). LC/MS (ESI) *m/z* 317.38; [M+H]$^+$ calcd for $C_{14}H_{16}ClF_2N_2O_2^+$: 317.09

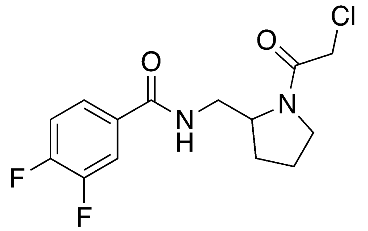

Synthesis of **CAS-12290-077** followed the General Procedure 1 by using 3,4-difluorobenzoic acid (0.150 g, 0.9487 mmol), and *tert*-butyl 2-(aminomethyl)pyrrolidine-1-carboxylate (0.200 mL, 1.04 mmol) to afford CAS-12290-077 (0.0291 g, 27.3%) ^{1}H NMR (500 MHz, Chloroform-*d*) δ 8.40 (s, 1H), 7.75 (ddd, *J* = 11.0, 7.6, 2.2 Hz, 1H), 7.60 (dddd, *J* = 8.6, 3.9, 2.2, 1.4 Hz, 1H), 7.23 (ddd, *J* = 9.9, 8.6, 7.7 Hz, 1H), 5.90 (s, 1H), 4.48 (ddt, *J* = 10.7, 7.4, 3.3 Hz, 1H), 4.10 (d, *J* = 1.9 Hz, 2H), 3.76 – 3.61 (m, 3H), 3.44 (ddd, *J* = 14.0, 10.4, 3.7 Hz, 1H), 2.21 – 1.97 (m, 3H), 1.93 – 1.70 (m, 1H). LC/MS (ESI) *m/z* 317.08; [M+H]$^+$ calcd for $C_{14}H_{16}ClF_2N_2O_2^+$: 317.09

Synthesis of CAS 12290 092, 087, 089, 091

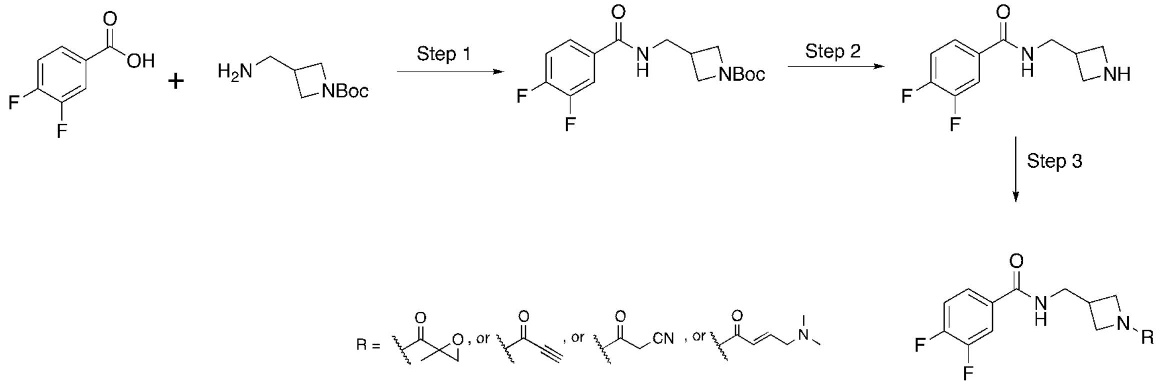

Step 1: Following methods described in Step 1 of General Procedure 1, 3,4-difluorobenzoic acid (0.500 g, 3.16 mmol), and *tert*-butyl 3-(aminomethyl)azetidine-1-carboxylate (0.636 mL, 3.48 mmol) were combined to form *tert*-butyl 3-((3,4-difluorobenzamido)methyl)azetidine-1-carboxylate (57.3%).

Step 2: Synthesis was performed adhering to methods describes in Step 2 of General Procedure 1 to afford *N*-(azetidin-3-ylmethyl)-3,4-difluorobenzamide (quant.)

Step 3: The synthesis was performed according to Step 1 of General Procedure 3 with *N*-(azetidin-3-ylmethyl)-3,4-difluorobenzamide (0.100 g, 0.3064 mmol), and 2-methyloxirane-2-carboxylic acid (0.045 mL, 0.4864 mmol), or propiolic acid (0.022 mL, 0.337 mmol), or 2-cyanoacetic acid (0.050 g, 0.574 mmol), or (*E*)-4-(dimethylamino)but-2-enoic acid (0.062 g, 0.4862 mmol). Combined organic layer was dried over Na_2SO_4, filtered, and concentrated under reduced pressure. The crude material was then purified by flash column chromatography (hexanes/EtOAc/MeOH) followed by preparative HPLC (MeOH or CH_3CN/H_2O with 0.0425% TFA) to afford the target products **CAS-12290-092** (7%), **CAS-12290-087**(15.3%), **CAS-12290-089** (17.8%), and **CAS-12290-091**(13.8%).

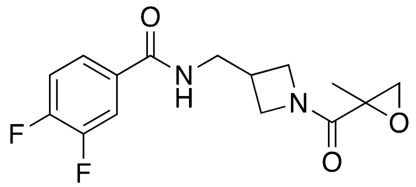

CAS-12290-092 ^{1}H NMR (500 MHz, Chloroform-*d*) δ 7.67 (dddt, *J* = 11.4, 7.5, 4.1, 2.0 Hz, 1H), 7.56 (ddh, *J* = 8.7, 6.6, 2.2 Hz, 1H), 7.24 (dddt, *J* = 11.7, 10.2, 8.0, 2.1 Hz, 1H), 7.04 – 6.55 (m, 1H), 4.65 – 4.46 (m, 1H), 4.46 – 4.24 (m, 1H), 4.16 (td, *J* = 9.3, 8.6, 4.7 Hz, 1H), 3.97 – 3.81 (m, 1H), 3.80 – 3.61 (m, 3H), 3.05 – 2.89 (m, 1H), 2.89 – 2.74 (m, 1H), 1.54 (d, *J* = 8.1 Hz, 1H), 1.51 – 1.37 (m, 1H), 1.31 (s, 1H). LC/MS (ESI) *m/z* 311.11; $[M+H]^+$ calcd for $C_{15}H_{17}F_2N_2O_3^+$: 311.12

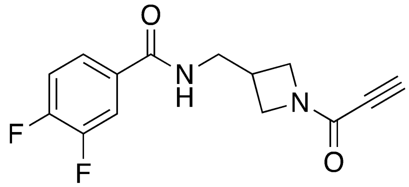

CAS-12290-087 ^{1}H NMR (500 MHz, Chloroform-*d*) δ 7.73 (ddd, *J* = 10.8, 7.5, 2.2 Hz, 1H), 7.61 (dddd, *J* = 8.7, 3.9, 2.2, 1.4 Hz, 1H), 7.24 (ddd, *J* = 9.9, 8.6, 7.6 Hz, 1H), 7.13 (d, *J* = 6.2 Hz, 1H), 4.35 (dd, *J* = 9.7, 8.2 Hz, 1H), 4.14 (dd, *J* = 10.7, 8.4 Hz, 1H), 4.02 (ddd, *J* = 9.8, 5.2, 1.2 Hz, 1H),

3.91 (ddd, *J* = 10.8, 5.3, 1.2 Hz, 1H), 3.78 (dt, *J* = 13.9, 6.3 Hz, 1H), 3.61 (ddd, *J* = 13.6, 7.5, 5.8 Hz, 1H), 3.02 (ddd, *J* = 14.0, 7.4, 3.3 Hz, 1H), 2.71 (s, 1H). LC/MS (ESI) *m/z* 279.09; $[M+H]^+$ calcd for $C_{14}H_{13}F_2N_2O_2^+$: 279.09

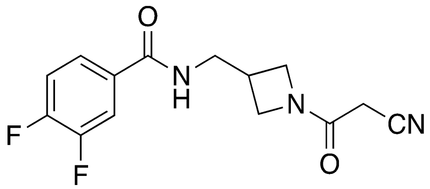

CAS-12290-089 ^{1}H NMR (500 MHz, Chloroform-*d*) δ 7.67 (ddd, *J* = 10.1, 7.4, 2.1 Hz, 1H), 7.53 (d, *J* = 8.8 Hz, 1H), 7.30 – 7.23 (m, 4H), 4.42 (t, *J* = 8.6 Hz, 1H), 4.21 (t, *J* = 9.4 Hz, 1H), 4.14 (dd, *J* = 8.8, 5.2 Hz, 1H), 3.88 (dd, *J* = 10.3, 5.4 Hz, 1H), 3.79 (dt, *J* = 13.6, 6.7 Hz, 1H), 3.68 (dt, *J* = 13.6, 6.2 Hz, 1H), 3.29 (s, 2H), 3.05 (s, 1H).). LC/MS (ESI) *m/z* 294.18; $[M+H]^+$ calcd for $C_{14}H_{14}F_2N_3O_2^+$: 294.10

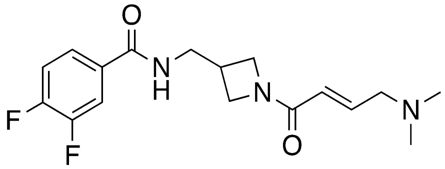

CAS-12290-091 ^{1}H NMR (500 MHz, DMSO-*d*$_6$) δ 10.05 (s, 1H), 8.78 (t, *J* = 5.7 Hz, 1H), 7.88 (ddd, *J* = 11.5, 7.8, 2.2 Hz, 1H), 7.77 – 7.65 (m, 1H), 7.57 (dt, *J* = 10.5, 8.3 Hz, 1H), 6.57 (dt, *J* = 15.2, 7.1 Hz, 2H), 6.42 – 6.34 (m, 1H), 4.28 (t, *J* = 8.5 Hz, 1H), 4.02 – 3.93 (m, 2H), 3.87 (dd, *J* = 7.1, 1.2 Hz, 2H), 3.72 (dd, *J* = 10.3, 5.4 Hz, 1H), 3.56 – 3.43 (m, 2H), 2.91 – 2.81 (m, 1H), 2.76 (s, 6H). LC/MS (ESI) *m/z* 338.16; $[M+H]^+$ calcd for $C_{17}H_{22}F_2N_3O_2^+$: 338.17

Synthesis of CAS-12290-094

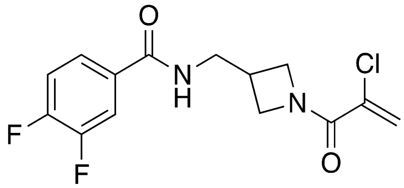

Synthesis of **CAS-12290-094** followed Step 3 of General Procedure 1 and used previously synthesized intermediate *N*-(azetidin-3-ylmethyl)-3,4-difluorobenzamide (0.050 g, 0.221 mmol), and 2-chloroacryloyl chloride (0.0226 mL, 0.243 mmol) to afford CAS-12290-094 (0.0124 g,17.9 %) ^{1}H NMR (500 MHz, Chloroform-*d*) δ 7.69 (ddd, *J* = 10.7, 7.5, 2.3 Hz, 1H), 7.57 (ddt, *J* = 8.0, 4.1, 1.8 Hz, 1H), 7.28 – 7.19 (m, 1H), 6.86 (s, 1H), 6.11 (d, *J* = 1.8 Hz, 1H), 5.80 (d, *J* = 1.7 Hz, 1H), 4.55 (t, *J* = 9.1 Hz, 1H), 4.21 (d, *J* = 9.8 Hz, 2H), 3.96 (dd, *J* = 11.1, 5.5 Hz, 1H), 3.85 – 3.54 (m,

2H), 3.00 (dddd, *J* = 13.5, 6.8, 3.0, 1.5 Hz, 1H). LC/MS (ESI) *m/z* 315.07; $[M+H]^+$ calcd for $C_{14}H_{14}ClF_2N_2O_2^+$: 315.06

Synthesis of ED-11370-017, 018:

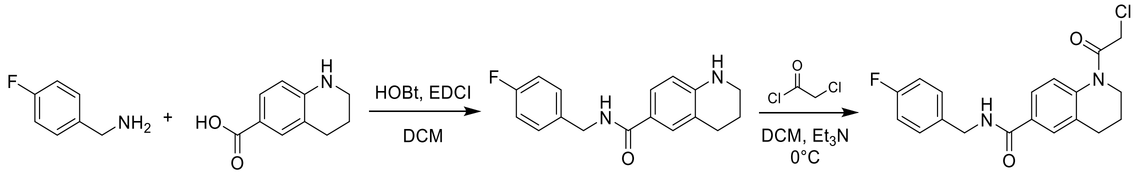

ED_11370_017

Step 1: 1,2,3,4-tetrahydroquinoline-6-carboxylic acid (0.088g, 0.5mmol), HOBt (80%, 0.12g, 0.6mmol), and EDCl (0.14g, 0.75mmol) were added sequentially into 3mL anhydrous DCM. Into the solution was added 4-fluorobenzylamine (0.063g, 0.5mmol). The mixture was stirred at room temperature overnight, and purified directly by flash column chromatography (hexanes/EtOAc/MeOH) to afford 0.15g product 4-fluorobenzyl 1,2,3,4-tetrahydroquinoline-6-carboxylate (quant.) LC/MS (ESI) *m/z* 284.67; $[M+H]^+$ calcd for $C_{17}H_{18}FN_2O^+$: 285.14

Step 2: 4-fluorobenzyl 1,2,3,4-tetrahydroquinoline-6-carboxylate (0.075g, 0.25mmol) was dissolved in 2.5mL anhydrous DCM. Into the solution was added Et_3N (0.18mL, 1.3mmol), and 2-chloroacetyl chloride (0.036g, 0.32mmol) at 0°C. The mixture was stirred for 10min before direct purification by flash column chromatography (hexanes/EtOAc/MeOH) and followed by preparative HPLC (CH_3CN/H_2O with 0.0425% TFA) to afford desired product ED_11370_017 (28mg, 30%). 1H NMR (500 MHz, DMSO) δ 9.00 (t, J = 5.8 Hz, 1H), 7.79 – 7.62 (m, 3H), 7.35 (dd, J = 8.5, 5.7 Hz, 2H), 7.21 – 7.10 (m, 2H), 4.59 (s, 2H), 4.45 (d, J = 5.9 Hz, 2H), 3.78 – 3.68 (t, J = 6.3 Hz, 2H), 2.78 (t, J = 6.6 Hz, 2H), 1.96 – 1.88 (m, 2H). LC/MS (ESI) *m/z* 360.77; $[M+H]^+$ calcd for $C_{19}H_{19}ClFN_2O_2^+$: 361.11

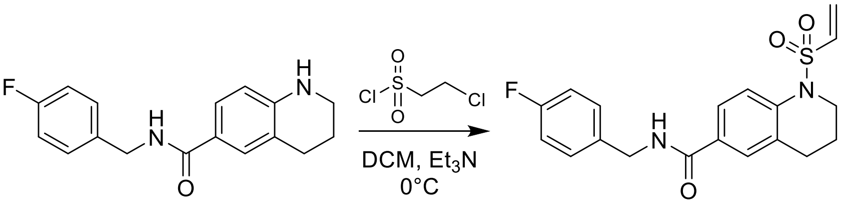

ED_11370_018

Step 1: 4-fluorobenzyl 1,2,3,4-tetrahydroquinoline-6-carboxylate (0.075g, 0.25mmol) was dissolved in 2.5mL anhydrous DCM. Into the solution was added Et_3N (0.18mL, 1.3mmol), and 2-chloroethanesulfonyl chloride (0.052g, 0.32mmol) at 0°C. The mixture was stirred for 10min

before direct purification by flash column chromatography (hexanes/EtOAc/MeOH) and followed by preparative HPLC (CH_3CN/H_2O with 0.0425% TFA) to afford desired product ED_11370_018 (10mg, 10%). 1H NMR (500 MHz, DMSO) δ 8.95 (t, J = 5.9 Hz, 1H), 7.73 – 7.63 (m, 2H), 7.56 (d, J = 8.7 Hz, 1H), 7.34 (dd, J = 8.8, 5.6 Hz, 2H), 7.15 (t, J = 8.9 Hz, 2H), 6.92 (dd, J = 16.4, 9.9 Hz, 1H), 6.14 (d, J = 16.4 Hz, 9.9 Hz, 2H), 4.44 (d, J = 6.1 Hz, 2H), 3.80 – 3.69 (m, 2H), 2.82 (t, J = 6.6 Hz, 2H), 1.93 (dd, J = 12.1, 6.1 Hz, 2H). LC/MS (ESI) *m/z* 374.97; $[M+H]^+$ calcd for $C_{19}H_{20}FN_2O_3S^+$: 375.12

Synthesis of ED_11370_027:

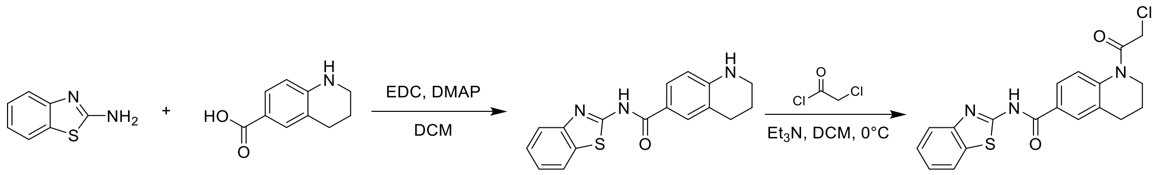

Step 1: 1,2,3,4-tetrahydroquinoline-6-carboxylic acid (0.26g, 1.5mmol), benzothiazole-2-amine (0.12g, 0.75mmol) were added into 3mL anhydrous DCM. Into the solution was added EDC (0.29g, 1.5mmol), and DMAP(0.37g, 3.0mmol) sequentially. The mixture was stirred at room temperature overnight, and purified directly by flash column chromatography (hexanes/EtOAc/MeOH) to afford 0.026g product N-(benzo[d]thiazol-2-yl)-1,2,3,4-tetrahydroquinoline-6-carboxamide (11%). LC/MS (ESI) *m/z* 309.87; $[M+H]^+$ calcd for $C_{17}H_{16}N_3OS^+$: 310.10

Step 2: N-(benzo[d]thiazol-2-yl)-1,2,3,4-tetrahydroquinoline-6-carboxamide (0.026g, 0.08mmol) was dissolved in 5mL anhydrous DCM. Into the solution was added Et_3N (0.085mL, 0.6mmol), and 2-chloroacetyl chloride (0.01uL, 0.14mmol) at 0°C. The mixture was stirred for 10min before direct purification by flash column chromatography (hexanes/EtOAc/MeOH) and followed by preparative HPLC (CH_3CN/H_2O with 0.0425% TFA) to afford desired product ED_11370_027 (27mg, 88%). 1H NMR (500 MHz, DMSO) δ 12.80 (s, 1H), 8.06 – 8.00 (m, 2H), 7.98 (dd, J = 8.6, 2.1 Hz, 1H), 7.81 (dd, J = 16.2, 8.1 Hz, 2H), 7.48 (t, J = 7.7 Hz, 1H), 7.35 (t, J = 7.6 Hz, 1H), 4.65 (s, 2H), 3.81 – 3.72 (t, J = 5.8 Hz, 2H), 2.84 (t, J = 6.5 Hz, 2H), 2.03 – 1.87 (m, 2H). LC/MS (ESI) *m/z* 385.97; $[M+H]^+$ calcd for $C_{19}H_{17}ClN_3O_2S^+$: 386.07

Synthesis of ED_11370_030:

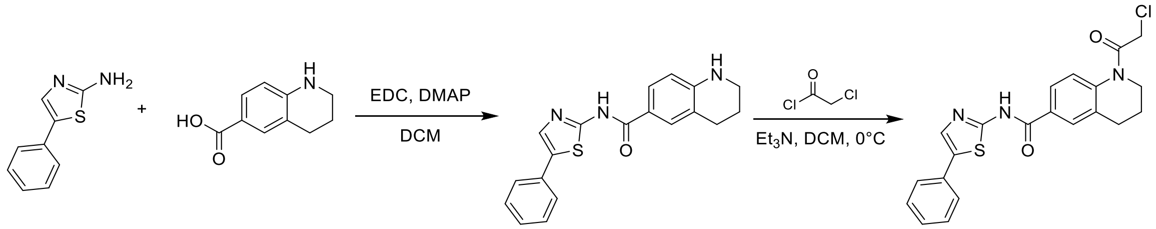

Step 1: 1,2,3,4-tetrahydroquinoline-6-carboxylic acid (0.26g, 1.5mmol), 5-phenylthiazol-2-amine (0.13g, 0.75mmol) were added into 3mL anhydrous DCM. Into the solution was added EDC (0.29g, 1.5mmol), and DMAP(0.28g, 2.3mmol) sequentially. The mixture was stirred at room temperature overnight, and purified directly by flash column chromatography (hexanes/EtOAc/MeOH) to afford 0.064g N-(5-phenylthiazol-2-yl)-1,2,3,4-tetrahydroquinoline-6-carboxamide (25%). LC/MS (ESI) *m/z* 335.87; $[M+H]^+$ calcd for $C_{19}H_{18}ClN_3OS^+$: 336.12

Step 2: N-(5-phenylthiazol-2-yl)-1,2,3,4-tetrahydroquinoline-6-carboxamide (0.064g, 0.2mmol) was dissolved in 5mL anhydrous DCM. Into the solution was added Et_3N (0.13mL, 0.9mmol), and 2-chloroacetyl chloride (0.017uL, 0.23mmol) at 0°C. The mixture was stirred for 10min before direct purification by flash column chromatography (hexanes/EtOAc/MeOH) and followed by preparative HPLC (CH_3CN/H_2O with 0.0425% TFA) to afford desired product ED_11370_030 (12mg, 15%). 1H NMR (500 MHz, DMSO) δ 12.63 (s, 1H), 8.01 (s, 1H), 7.99 – 7.92 (m, 2H), 7.80 (d, J = 8.0 Hz, 1H), 7.66 (d, J = 7.6 Hz, 2H), 7.44 (t, J = 7.7 Hz, 2H), 7.33 (t, J = 7.4 Hz, 1H), 4.64 (s, 2H), 3.82 – 3.70 (t, J = 6.1 Hz, 2H), 2.84 (t, J = 6.4 Hz, 2H), 2.04 – 1.88 (m, 2H). LC/MS (ESI) *m/z* 411.97; $[M+H]^+$ calcd for $C_{21}H_{19}ClN_3O_2S^+$: 412.09

Synthesis of CAS-12290-201

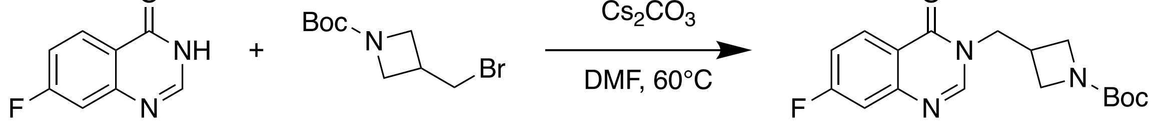

7-Fluoroquinazolin-4(3H)-one (82.02 mg, 0.5 mmol), tert-butyl 3-(bromomethyl)azetidine-1-carboxylate (150.0 mg, 0.6 mmol), and Cs_2CO_3 (325.6 mg, 1.0 mmol) were combined in DMF (1.5 mL) and stirred at 60℃ overnight. The reaction was diluted with water (25 mL) and extracted with ethyl acetate (25 mL x 2). The combined organics were washed with brine (50

mL x 1), dried over anhydrous Na_2SO_4, filtered, concentrated, and purified by flash chromatography (50% to 100% EtOAc / hexanes) to afford 129.2 mg the product (77.5%). LCMS (m/z): 334.14 $[M + H]^+$; calculated for $C_{17}H_{21}FN_3O_3^+$: 334.16.

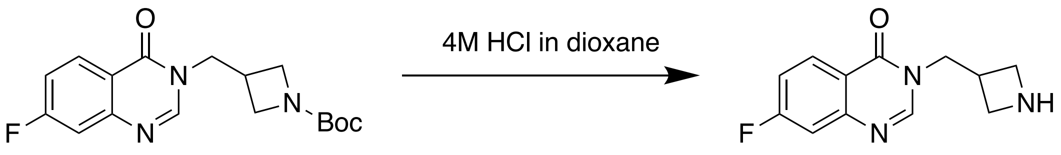

Tert-butyl 3-((7-fluoro-4-oxoquinazolin-3(4*H*)-yl)methyl)azetidine-1-carboxylate (110.1 mg, 0.330 mmol) was combined with 4M HCl in dioxane (2 mL) and then stirred at rt for 30 minutes. Reaction mixture was concentrated under reduced pressure to afford crude material which was used directly without any further purification. LCMS (m/z): 234.21 $[M + H]^+$. calcd for $C_{12}H_{13}FN_3O^+$: 234.10.

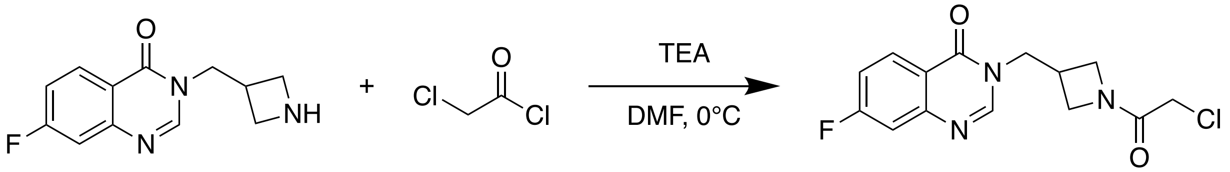

3-(azetidin-3-ylmethyl)-7-fluoroquinazolin-4(3*H*)-one (65.2 mg, 0.280 mmol) and Et_3N (97.40 µL, 0.699 mmol) was taken up in DMF (0.5 mL) and cooled to 0℃. 2-chloroacetyl chloride (26.74 µL, 0.335 mmol) was added dropwise to the reaction mixture at 0℃. The reaction was warmed to rt and stirred for 2 hours. The mixture was purified by flash chromatography (0% to 100% EtOAc / hexanes), followed by preparative HPLC (MeCN/H2O with 0.0425% TFA) to afford 32.1 mg product (37.1%). LCMS ESI (m/z): 309.85 $[M + H]^+$. ; calculated for $C_{14}H_{13}ClFN_3O_2^+$: 309.07. ^1H NMR (500 MHz, DMSO-d6) δ 8.50 (s, 1H), 8.20 (dd, J = 8.9, 6.3 Hz, 1H), 7.45 (dd, J = 10.0, 2.6 Hz, 1H), 7.40 (td, J = 8.7, 2.6 Hz, 1H), 4.29 – 4.21 (m, 3H), 4.12 (s, 2H), 4.07 (dd, J = 8.9, 5.6 Hz, 1H), 3.98 (t, J = 9.2 Hz, 1H), 3.80 (dd, J = 9.9, 5.6 Hz, 1H), 3.15 – 3.05 (m, 1H). ^{13}C NMR (126 MHz, DMSO-d_6) δ 166.21, 166.03 (d, *J* = 251.3 Hz), 160.32, 150.51 (d, *J* = 13.3 Hz), 149.72, 129.71 (d, *J* = 10.9 Hz), 119.09 (d, *J* = 1.9 Hz), 116.12 (d, *J* = 23.6 Hz), 112.72 (d, *J* = 21.7 Hz), 53.68, 51.82, 49.12, 40.45, 28.53.

Supplementary References

1. Lamberto, I. *et al.* Structure-Guided Development of a Potent and Selective Non-covalent Active-Site Inhibitor of USP7. *Cell Chem Biol* **24**, 1490-1500.e11 (2017).

2. Turnbull, A. P. *et al.* Molecular basis of USP7 inhibition by selective small molecule inhibitors. *Nature* **550**, 481–486 (2017).

3. Zhang, Z. & Marshall, A. G. A universal algorithm for fast and automated charge state deconvolution of electrospray mass-to-charge ratio spectra. *Journal of the American Society for Mass Spectrometry* **9**, 225–233 (1998).

4. Hughes, C. S. *et al.* Ultrasensitive proteome analysis using paramagnetic bead technology. *Mol. Syst. Biol.* **10**, 757 (2014).

5. Alexander, W. M., Ficarro, S. B., Adelmant, G. & Marto, J. A. multiplierz v2.0: A Python-based ecosystem for shared access and analysis of native mass spectrometry data. *PROTEOMICS* **17**, 1700091 (2017).

6. Askenazi, M., Parikh, J. R. & Marto, J. A. mzAPI: a new strategy for efficiently sharing mass spectrometry data. *Nature Methods* **6**, 240–241 (2009).

7. Ficarro, S. B., Alexander, W. M. & Marto, J. A. mzStudio: A Dynamic Digital Canvas for User-Driven Interrogation of Mass Spectrometry Data. *Proteomes* **5**, 20 (2017).

8. Ficarro, S. B., Max Alexander, W., Tavares, I. & Marto, J. A. Open source fraction collector/MALDI spotter for proteomics. *HardwareX* **11**, e00305 (2022).

9. Ficarro, S. B. *et al.* Improved Electrospray Ionization Efficiency Compensates for Diminished Chromatographic Resolution and Enables Proteomics Analysis of Tyrosine Signaling in Embryonic Stem Cells. *Anal. Chem.* **81**, 3440–3447 (2009).

10. Zhu, H. *et al.* PRM-LIVE with Trapped Ion Mobility Spectrometry and Its Application in Selectivity Profiling of Kinase Inhibitors. *Anal. Chem.* **93**, 13791–13799 (2021).

11. Yu, F. *et al.* Identification of modified peptides using localization-aware open search. *Nat Commun* **11**, 4065 (2020).

12. Zhang, Y. *et al.* A Robust Error Model for iTRAQ Quantification Reveals Divergent Signaling between Oncogenic FLT3 Mutants in Acute Myeloid Leukemia. *Mol Cell Proteomics* **9**, 780–790 (2010).

13. Benjamini, Y. & Hochberg, Y. Controlling the False Discovery Rate: A Practical and Powerful Approach to Multiple Testing. *Journal of the Royal Statistical Society: Series B (Methodological)* **57**, 289–300 (1995).
