# Supplementary material for: Accelerating inhibitor discovery for deubiquitinating enzymes
Source: Nat Commun. 2023 Feb 8;14:686. doi: 10.1038/s41467-023-36246-0 (PMC9908924; doi:10.1038/s41467-023-36246-0)
Supplement: Supplementary file 2 — Description of Additional Supplementary Files [file 41467_2023_36246_MOESM2_ESM.docx]

**Description of Additional Supplementary Files**

File Name: Supplementary Data 1

Description: Primary screening compound structures with primary screening results, as shown in figure 3a

File Name: Supplementary Data 2

Description: List of compounds which gave competition values >50% against each DUB

File Name: Supplementary Data 3

Description: List of hits shown in figure 4b with primary screening data and structures

File Name: Supplementary Data 4

Description: F-70/dtb-F-70 competition values

File Name: Supplementary Data 5

Description: Cysteine profiling experiment for WH-9943-103C, with XL177A as a positive control, as in figure 5f
