# Supplementary material for: Accelerating inhibitor discovery for deubiquitinating enzymes
Source: Nat Commun. 2023 Feb 8;14:686. doi: 10.1038/s41467-023-36246-0 (PMC9908924; doi:10.1038/s41467-023-36246-0)
Supplement: Supplementary file 8 — Reporting Summary [file 41467_2023_36246_MOESM8_ESM.pdf]

nature portfolio

Corresponding author(s): Jarrod A. Marto, Sara J. Buhrlage

Last updated by author(s): 01/08/2023

Reporting Summary

Nature Portfolio wishes to improve the reproducibility of the work that we publish. This form provides structure for consistency and transparency in reporting. For further information on Nature Portfolio policies, see our Editorial Policies and the Editorial Policy Checklist.

Statistics

For all statistical analyses, confirm that the following items are present in the figure legend, table legend, main text, or Methods section.

n/a	Confirmed	
☐	☒	The exact sample size (n) for each experimental group/condition, given as a discrete number and unit of measurement
☐	☒	A statement on whether measurements were taken from distinct samples or whether the same sample was measured repeatedly
☐	☒	The statistical test(s) used AND whether they are one- or two-sided<br>*Only common tests should be described solely by name; describe more complex techniques in the Methods section.*
☒	☐	A description of all covariates tested
☒	☐	A description of any assumptions or corrections, such as tests of normality and adjustment for multiple comparisons
☐	☒	A full description of the statistical parameters including central tendency (e.g. means) or other basic estimates (e.g. regression coefficient) AND variation (e.g. standard deviation) or associated estimates of uncertainty (e.g. confidence intervals)
☐	☒	For null hypothesis testing, the test statistic (e.g. F, t, r) with confidence intervals, effect sizes, degrees of freedom and P value noted<br>*Give P values as exact values whenever suitable.*
☒	☐	For Bayesian analysis, information on the choice of priors and Markov chain Monte Carlo settings
☒	☐	For hierarchical and complex designs, identification of the appropriate level for tests and full reporting of outcomes
☒	☐	Estimates of effect sizes (e.g. Cohen's d, Pearson's r), indicating how they were calculated

Our web collection on statistics for biologists contains articles on many of the points above.

Software and code

Policy information about availability of computer code

Data collection	No specialty software was used to collect the data.
Data analysis	Mulitplierz 2.2.0 scripts are available on github. Mascot ver 2.6, MSfragger ver 3.5 were used to search proteomics data.

For manuscripts utilizing custom algorithms or software that are central to the research but not yet described in published literature, software must be made available to editors and reviewers. We strongly encourage code deposition in a community repository (e.g. GitHub). See the Nature Portfolio guidelines for submitting code & software for further information.

Data

Policy information about availability of data

All manuscripts must include a data availability statement. This statement should provide the following information, where applicable:
- Accession codes, unique identifiers, or web links for publicly available datasets
- A description of any restrictions on data availability
- For clinical datasets or third party data, please ensure that the statement adheres to our policy

The native mass spectrometry data files associated with this study are available for free download from the MassIVE public repository using the accession: MSV000088637. Refseq can be accessed from www.ncbi.nlm.nih.gov/refseq/. Additional source data are provided with this paper.

Human research participants

Policy information about studies involving human research participants and Sex and Gender in Research.

Reporting on sex and gender	No human research participants
Population characteristics	No human research participants
Recruitment	No human research participants
Ethics oversight	No human research participants

Note that full information on the approval of the study protocol must also be provided in the manuscript.

Field-specific reporting

Please select the one below that is the best fit for your research. If you are not sure, read the appropriate sections before making your selection.

☒ Life sciences ☐ Behavioural & social sciences ☐ Ecological, evolutionary & environmental sciences

For a reference copy of the document with all sections, see nature.com/documents/nr-reporting-summary-flat.pdf

Life sciences study design

All studies must disclose on these points even when the disclosure is negative.

Sample size	For cell viability experiments, n>3 biologically independent experiments were carried out to enable calculation of standard deviations. A total of n=25 ABPP primary screening assay runs were carried out so as to screen the entire library, each compound was screened once in the primary screen.
Data exclusions	Data was not excluded
Replication	Attempts at replication with 3-point dose response of 24 primary screen hits (n=1 biologically independent experiment), carried out independently of the original primary screen were successful.
Randomization	Randomization was not applicable to this study, as no studies on advanced organisms were performed.
Blinding	No blinding techniques were used in this study, experiments were carried out with labelled compounds.

Reporting for specific materials, systems and methods

We require information from authors about some types of materials, experimental systems and methods used in many studies. Here, indicate whether each material, system or method listed is relevant to your study. If you are not sure if a list item applies to your research, read the appropriate section before selecting a response.

Materials & experimental systems

n/a	Involved in the study	
☐	☒	Antibodies
☐	☒	Eukaryotic cell lines
☒	☐	Palaeontology and archaeology
☒	☐	Animals and other organisms
☒	☐	Clinical data
☒	☐	Dual use research of concern

Methods

n/a	Involved in the study	
☒	☐	ChIP-seq
☒	☐	Flow cytometry
☒	☐	MRI-based neuroimaging

Antibodies

Antibodies used	Described in SI 1: USP25 (ab187156) and USP28 (ab188240) antibodies were obtained from abcam. GAPDH (2118s, D4C6R), UCHL1 (13179S), UCHL3 (3525S), USP28 (4217S), USP7 (4833s) antibodies were obtained from Cell Signaling Technology. VCPIP1 (A302-933) and USP48 (A301-190A-M) antibodies were obtained from Bethyl Laboratories. Antibodies were used at 1:1000 dilution for primary blotting as described in the "DUB ABP Labeling for Western blot target engagement" section in Methods.
Validation	Statement from vendors:

ab187156: Rabbit monoclonal [EPR15019] to USP25, reacts with mouse, rat, human
ab188240: Rabbit polyclonal to USP28, reacts with mouse, rat, human
GAPDH Cell Signaling Technology antibodies: GAPDH (D4C6R) Mouse mAb recognizes endogenous levels of total GAPDH protein.S pecies Reactivity: Human, Mouse, Rat, Monkey
13179S: UCHL1 (D3T2E) XP® Rabbit mAb recognizes endogenous levels of total UCHL1 protein. This antibody does not cross-react with other UCH family members. Species Reactivity: Human, Mouse, Rat, Monkey.
3525S: UCHL3 Antibody detects endogenous levels of total UCHL3 protein. Species Reactivity: Human, Mouse, Rat, Monkey.
4217S: USP28 Antibody detects transfected levels of USP28 protein. Species Reactivity: Human, Mouse
4833S: detects endogenous levels of total HAUSP protein. Species Reactivity: Human, Mouse, Rat, Monkey
A302-933: Antibody was affinity purified using an epitope specific to VCIP135 immobilized on solid support. The epitope recognized by A302-933A maps to a region between residue 1100 and 1150 of human Valosin-Containing Protein (p97)/p47 Complex-Interacting Protein p135 using the numbering given in entry NP_079330.2 (GeneID 80124). Antibody concentration was determined by extinction coefficient: absorbance at 280 nm of 1.4 equals 1.0 mg of IgG. Verified Reactivity: Human
A301-190A-M: Antibody was affinity purified using an epitope specific to USP48 immobilized on solid support. The epitope recognized by A301-190A maps to a region between residue 985 and 1035 of human ubiquitin specific peptidase 48 using the numbering given in entry NP_115612.4 (GeneID 84196). Immunoglobulin concentration was determined by extinction coefficient: absorbance at 280 nm of 1.4 equals 1.0 mg of IgG. Verified Reactivity: Mouse, Human

Eukaryotic cell lines

Policy information about cell lines and Sex and Gender in Research

Cell line source(s)	ATCC: HEK293T (CRL-3216), MCF7 (HTB-22)
Authentication	Cell lines were not separately authenticated
Mycoplasma contamination	Not tested
Commonly misidentified lines (See ICLAC register)	Commonly misidentified cell lines were not used in this study.
