# Supplementary material for: Accelerating inhibitor discovery for deubiquitinating enzymes
Source: Nat Commun. 2023 Feb 8;14:686. doi: 10.1038/s41467-023-36246-0 (PMC9908924; doi:10.1038/s41467-023-36246-0)
Supplement: Supplementary file 9 — Source Data [file 41467_2023_36246_MOESM9_ESM.zip › Source data_230119/Note_on_mass_spectrometry_data.rtf]

The native mass spectrometry data files associated with this study are available for free download from the Mass spectrometry Interactive Virtual Environment (MassIVE) public repository using the accession: MSV000088637. Alternatively, please click on the link below: http://secure-web.cisco.com/1T6MRItClHGTVb0xKUc8j9mcuKsUJOQYXUrvmTYbh38fUtnOiWcq3rfAfWOvKLRrn_eJE4LCyHRXl6HSj3tloH_9oY9xCdzTQohHfVUPYequwcfQ41AblxJrbGyI3QFHG0PcmZAQO5CyFn-ERQzopC1f9CM94FlTA2F_36bXBqolz2PtoQoCLh_q2VfrQudG_clQeVQoLYFerv3x5tLwWKQr-TXBEudeFJWGB2_S46xZ5qIBrDbjjapw5q1Z7mCXeRoczzSz9YzWCh2i5LgsM8soLdQAJh0A3uWIOGoMXzm3tWhWx5d1f54CvFgBGMdE3DLTHRnpe0n23tJJgDqtijSEAxLpf3oRsuHr737raQwo/http%3A%2F%2Fmassive.ucsd.edu%2FProteoSAFe%2Fstatus.jsp%3Ftask%3D622c60ccd7b841aba3fc1c356278dc06
