# Supplementary material for: Accelerating inhibitor discovery for deubiquitinating enzymes
Source: Nat Commun. 2023 Feb 8;14:686. doi: 10.1038/s41467-023-36246-0 (PMC9908924; doi:10.1038/s41467-023-36246-0)
Supplement: Supplementary file 9 — Source Data [file 41467_2023_36246_MOESM9_ESM.zip › Source data_230119/Uncropped gel images/Supplementary Figure 4.pptx]

## Slide 1
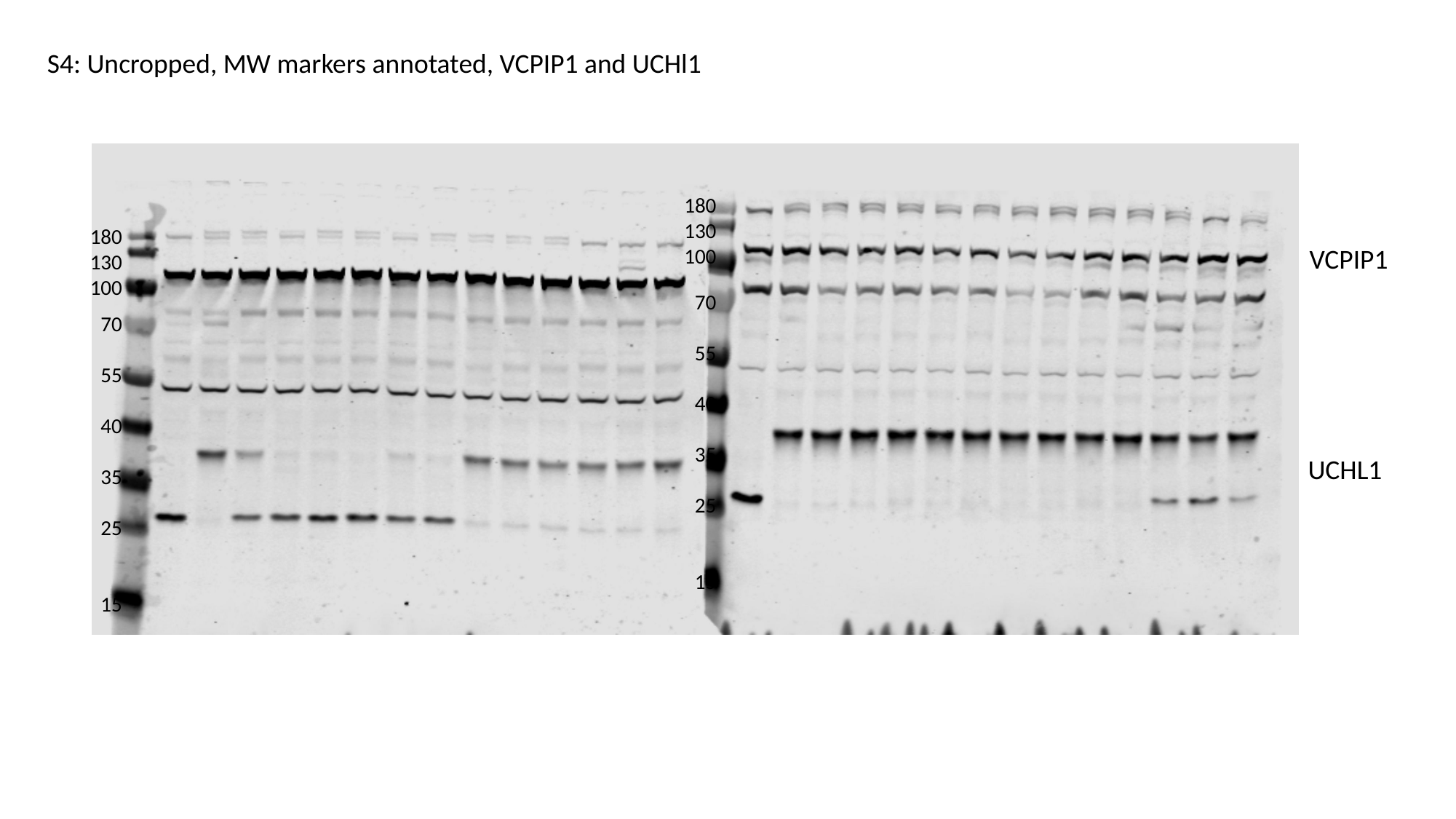

S4: Uncropped, MW markers annotated, VCPIP1 and UCHl1
180
130
100
70
55
40
35
25
15
180
130
100
70
55
40
35
25
15
VCPIP1
UCHL1

## Slide 2
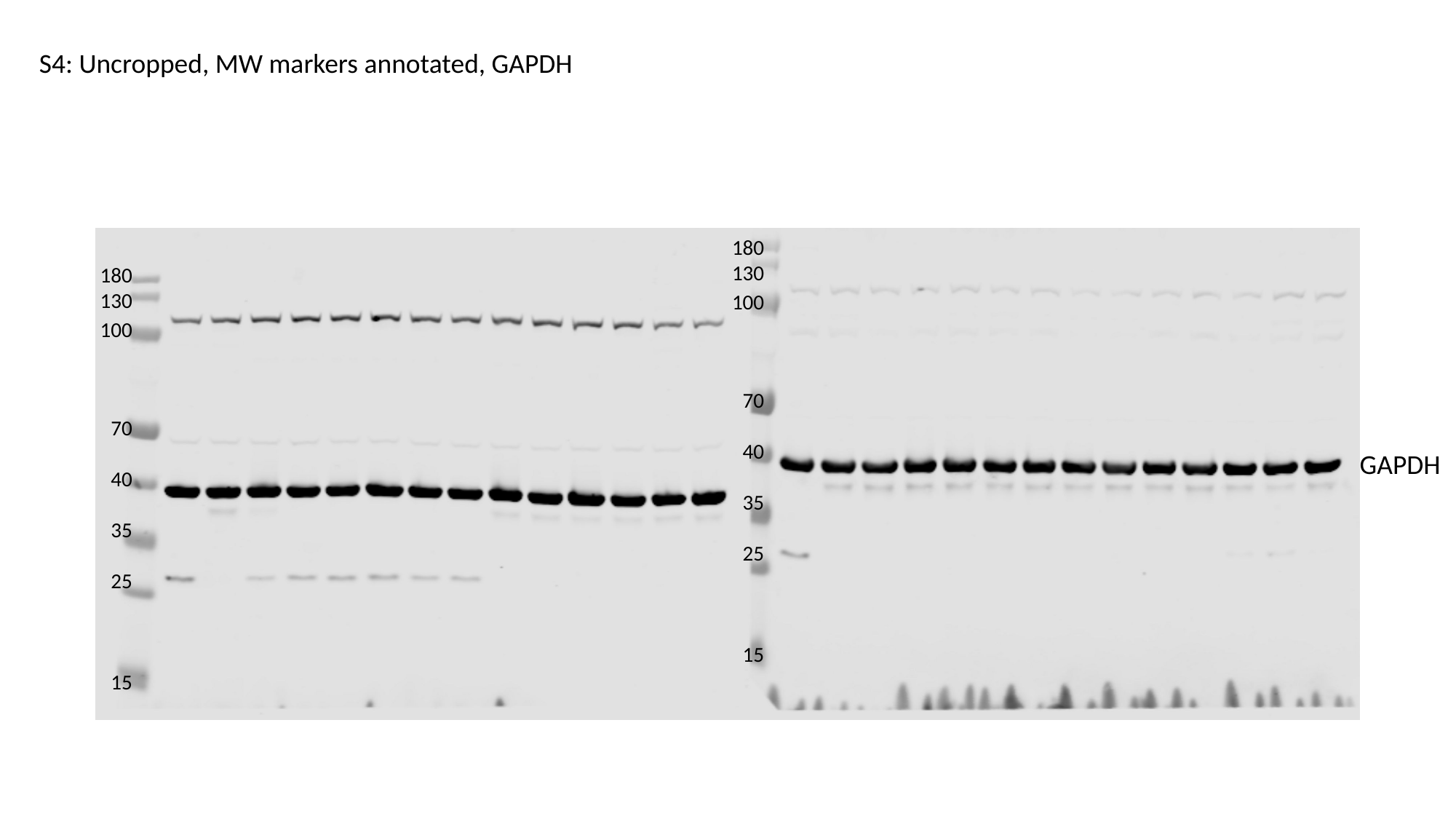

S4: Uncropped, MW markers annotated, GAPDH
180
130
100
70
40
35
25
15
180
130
100
70
40
35
25
15
GAPDH

## Slide 3
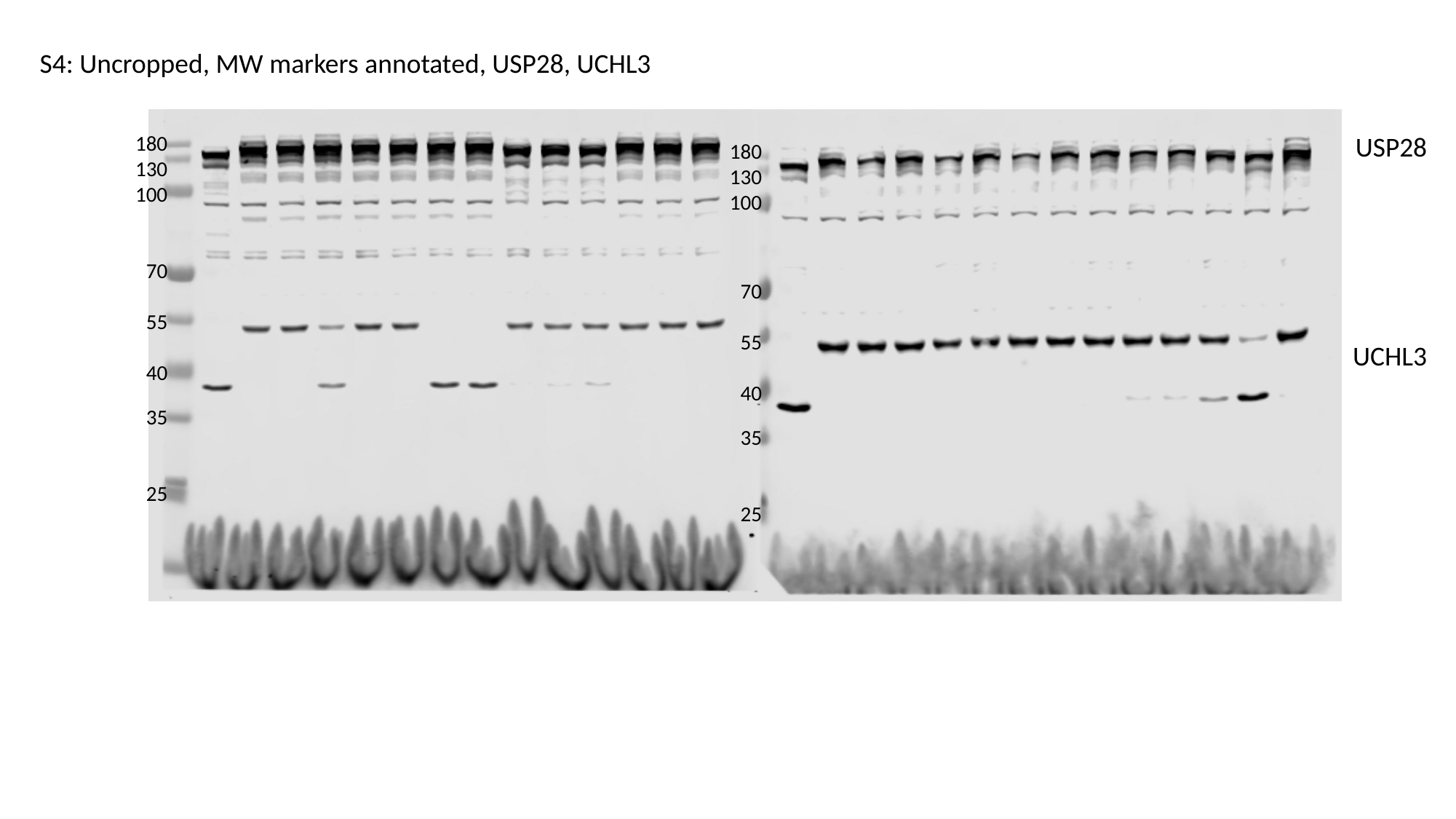

S4: Uncropped, MW markers annotated, USP28, UCHL3
180
130
100
70
55
40
35
25
USP28
180
130
100
70
55
40
35
25
UCHL3

## Slide 4
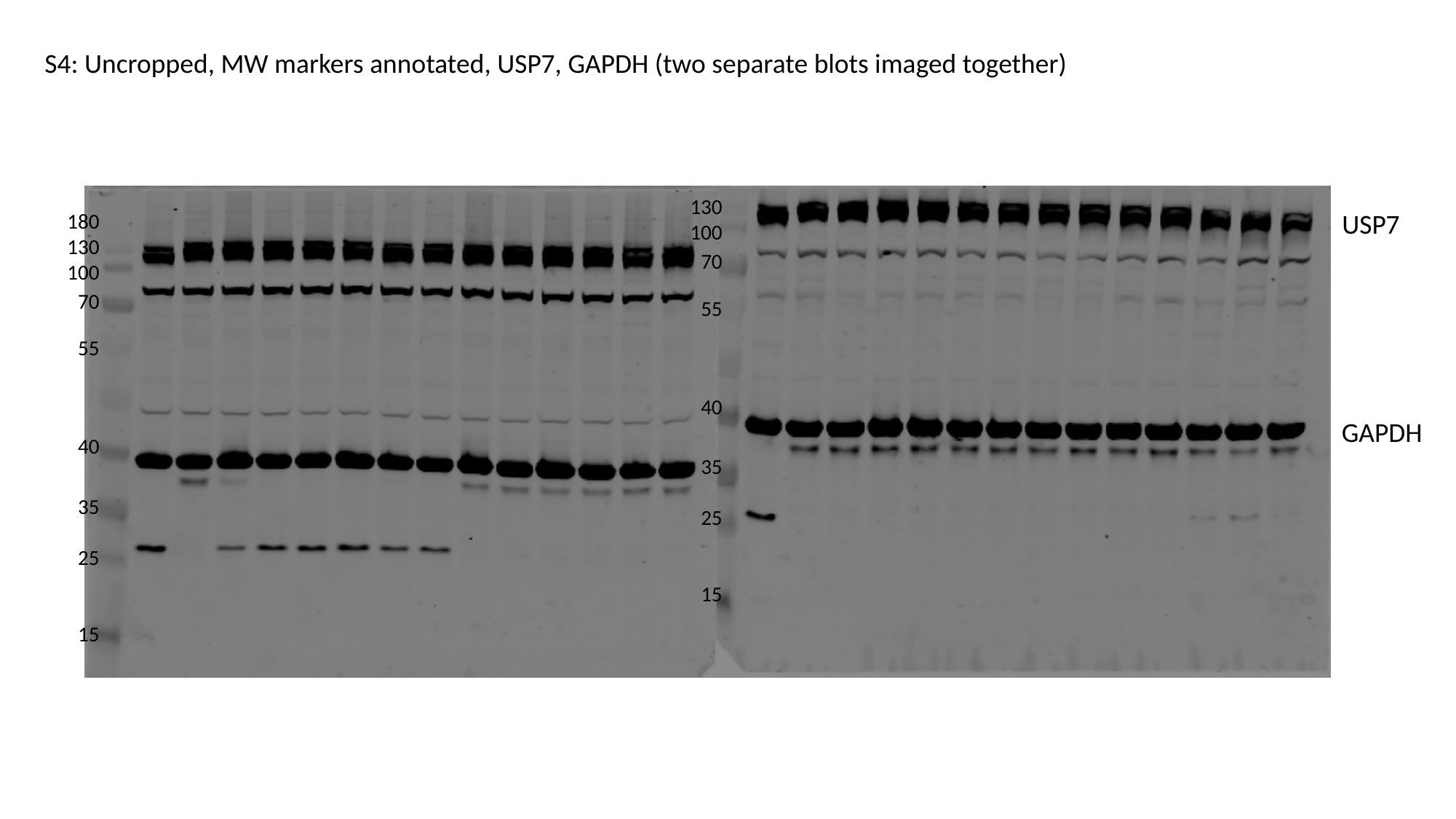

S4: Uncropped, MW markers annotated, USP7, GAPDH (two separate blots imaged together)
130
100
70
55
40
35
25
15
USP7
180
130
100
70
55
40
35
25
15
GAPDH

## Slide 5
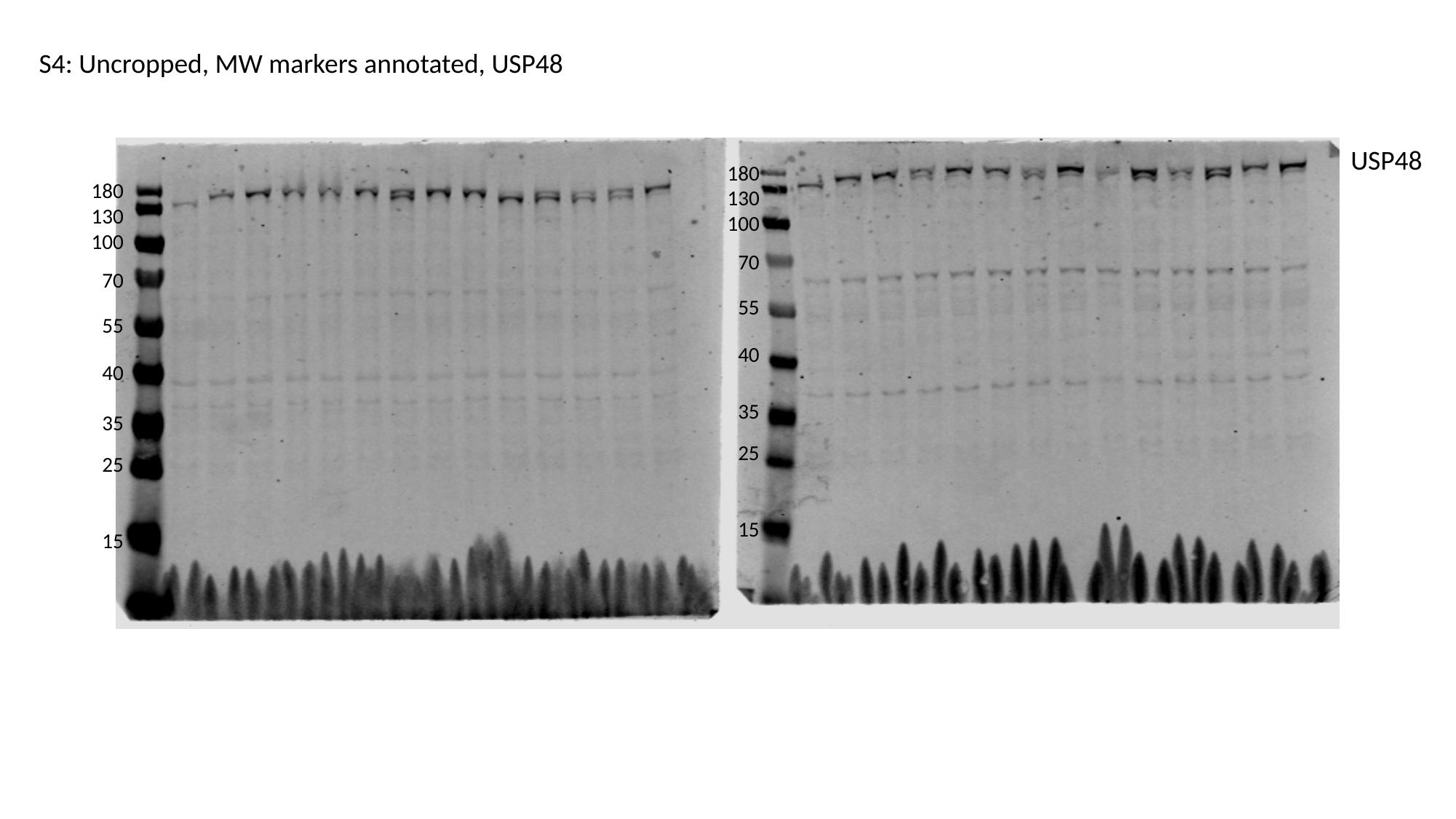

S4: Uncropped, MW markers annotated, USP48
USP48
180
130
100
70
55
40
35
25
15
180
130
100
70
55
40
35
25
15
