# Supplementary figures and images for: Accelerating inhibitor discovery for deubiquitinating enzymes
Source: Nat Commun. 2023 Feb 8;14:686. doi: 10.1038/s41467-023-36246-0 (PMC9908924; doi:10.1038/s41467-023-36246-0)

## Slide 1
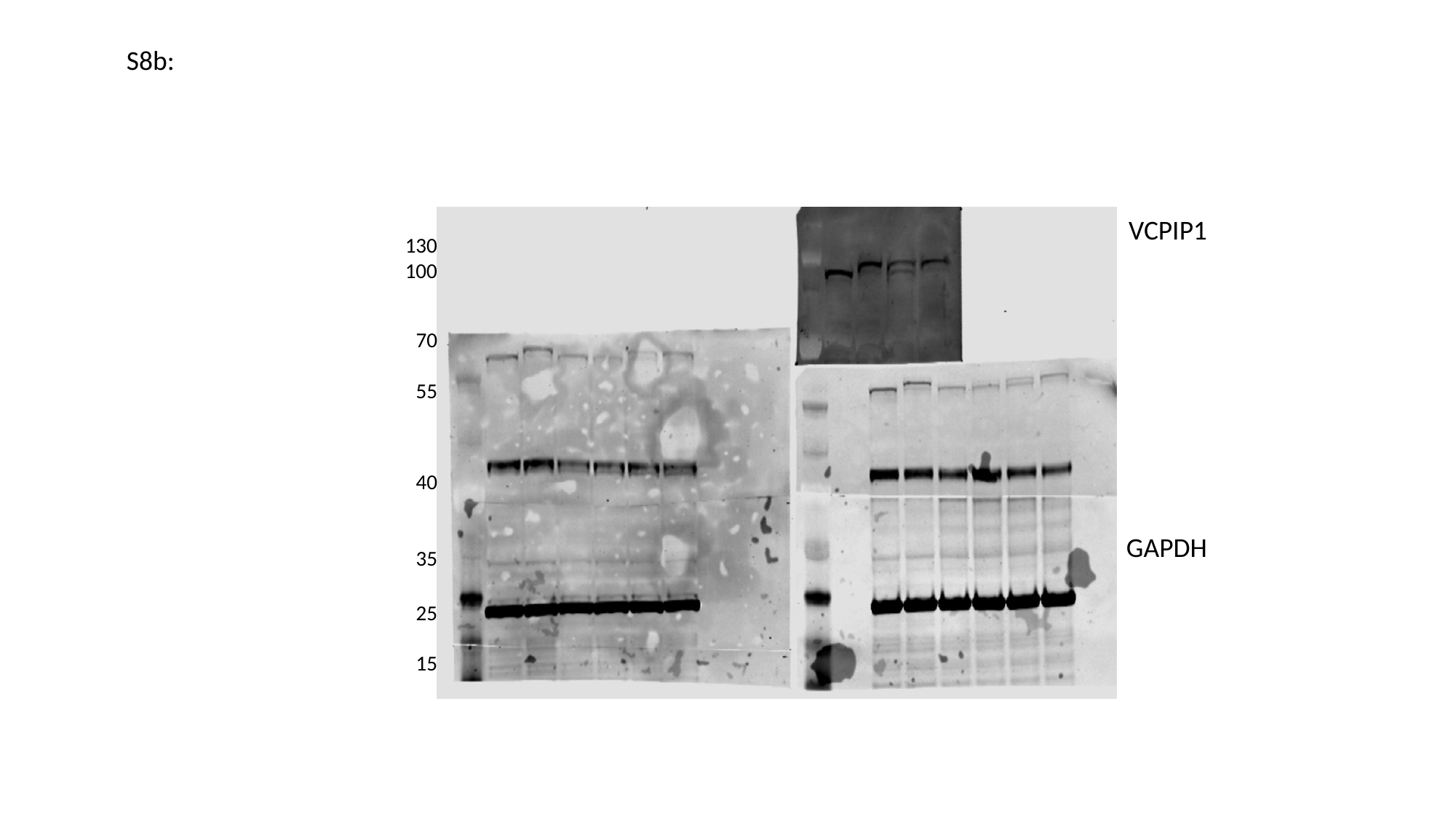

S8b:
130
100
70
55
40
35
25
15
VCPIP1
GAPDH

Supplement: Supplementary file 9 — Source Data [file 41467_2023_36246_MOESM9_ESM.zip › Source data_230119/Uncropped gel images/Supplementary Figure 8b.pptx]

## Slide 1
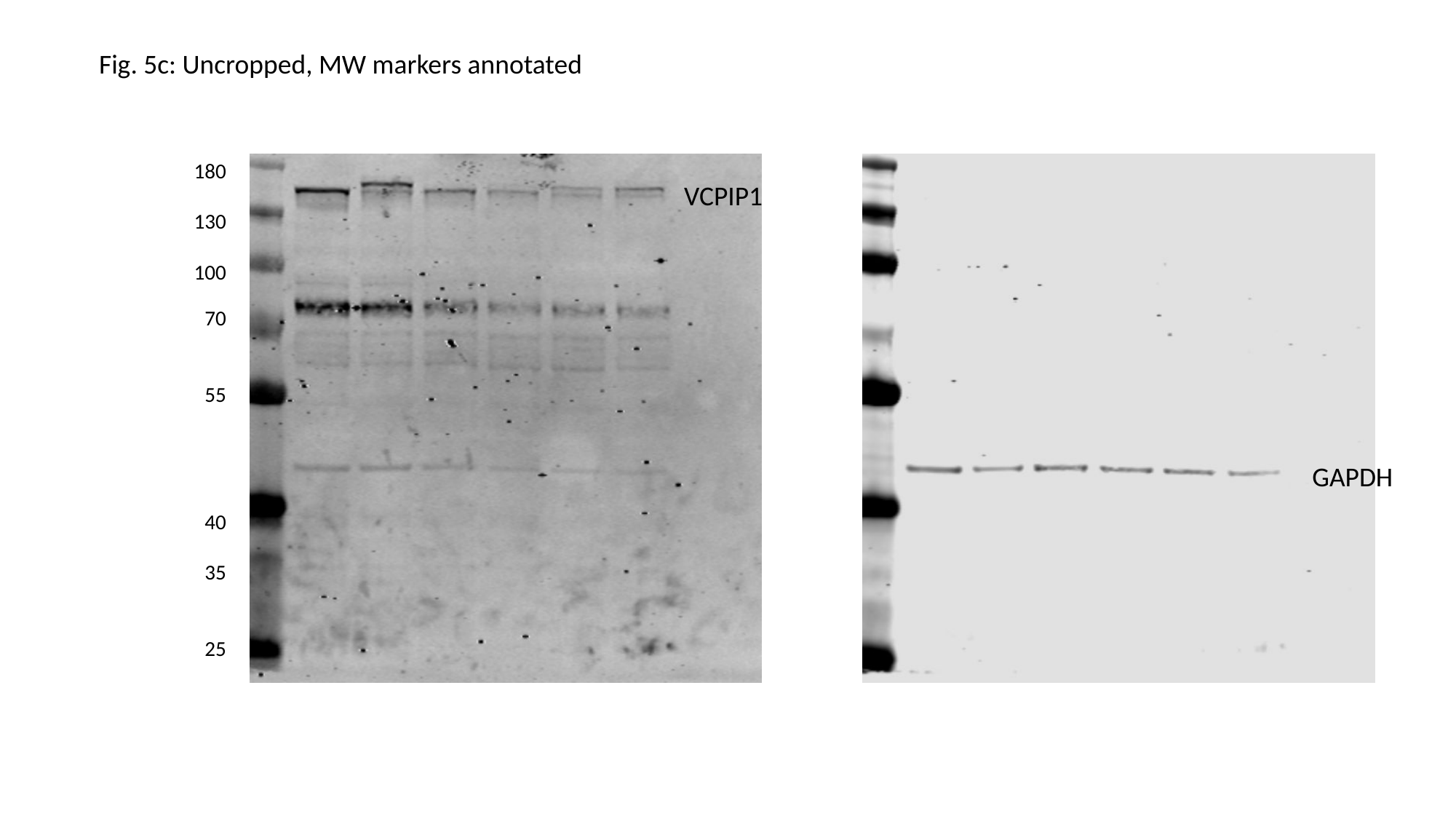

Fig. 5c: Uncropped, MW markers annotated
180
130
100
70
55
40
35
25
VCPIP1
GAPDH

Supplement: Supplementary file 9 — Source Data [file 41467_2023_36246_MOESM9_ESM.zip › Source data_230119/Uncropped gel images/Figure 5c.pptx]

## Slide 1
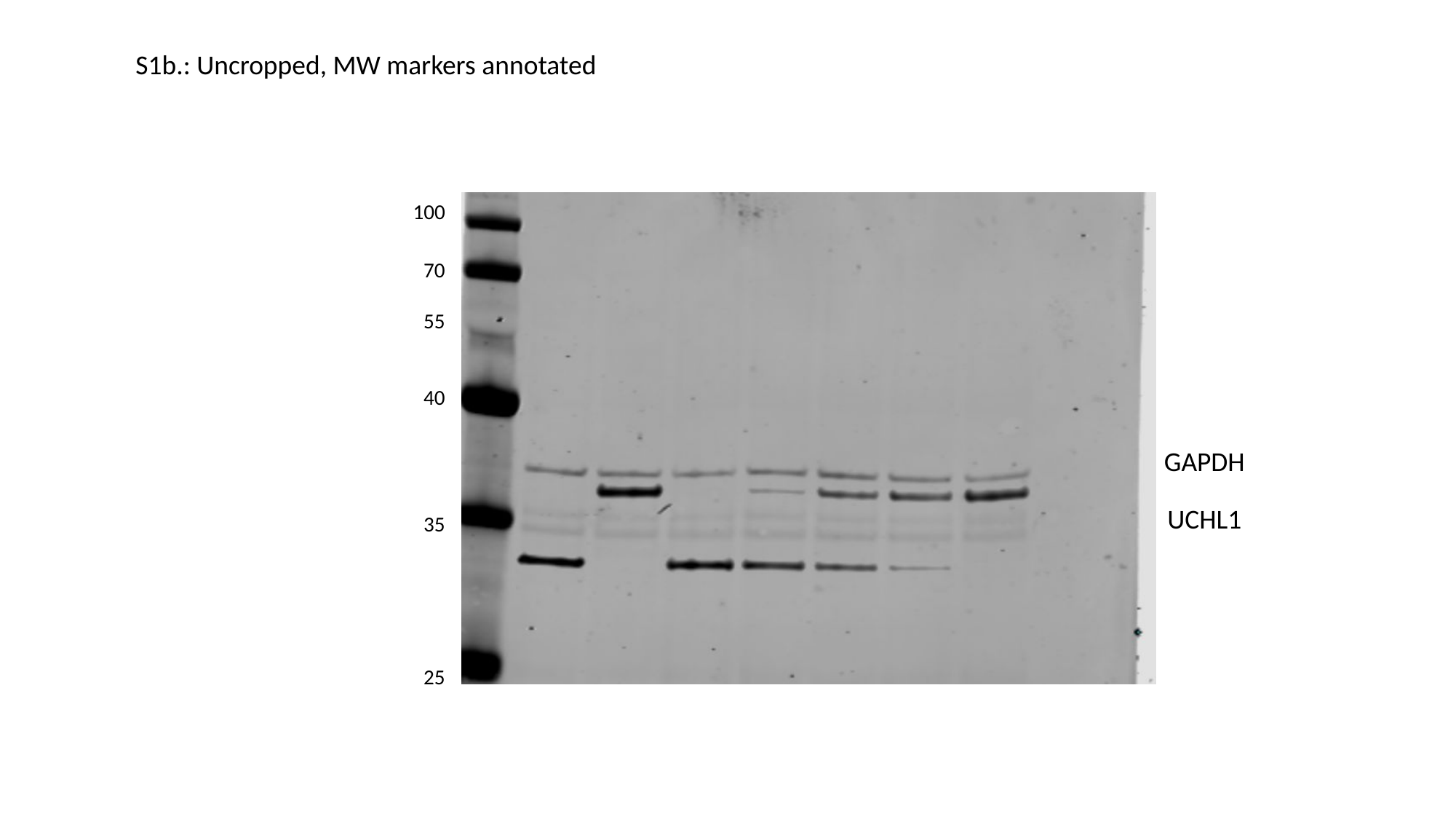

S1b.: Uncropped, MW markers annotated
100
70
55
40
35
25
GAPDH
UCHL1

Supplement: Supplementary file 9 — Source Data [file 41467_2023_36246_MOESM9_ESM.zip › Source data_230119/Uncropped gel images/Supplementary Figure 1b.pptx]

## Slide 1
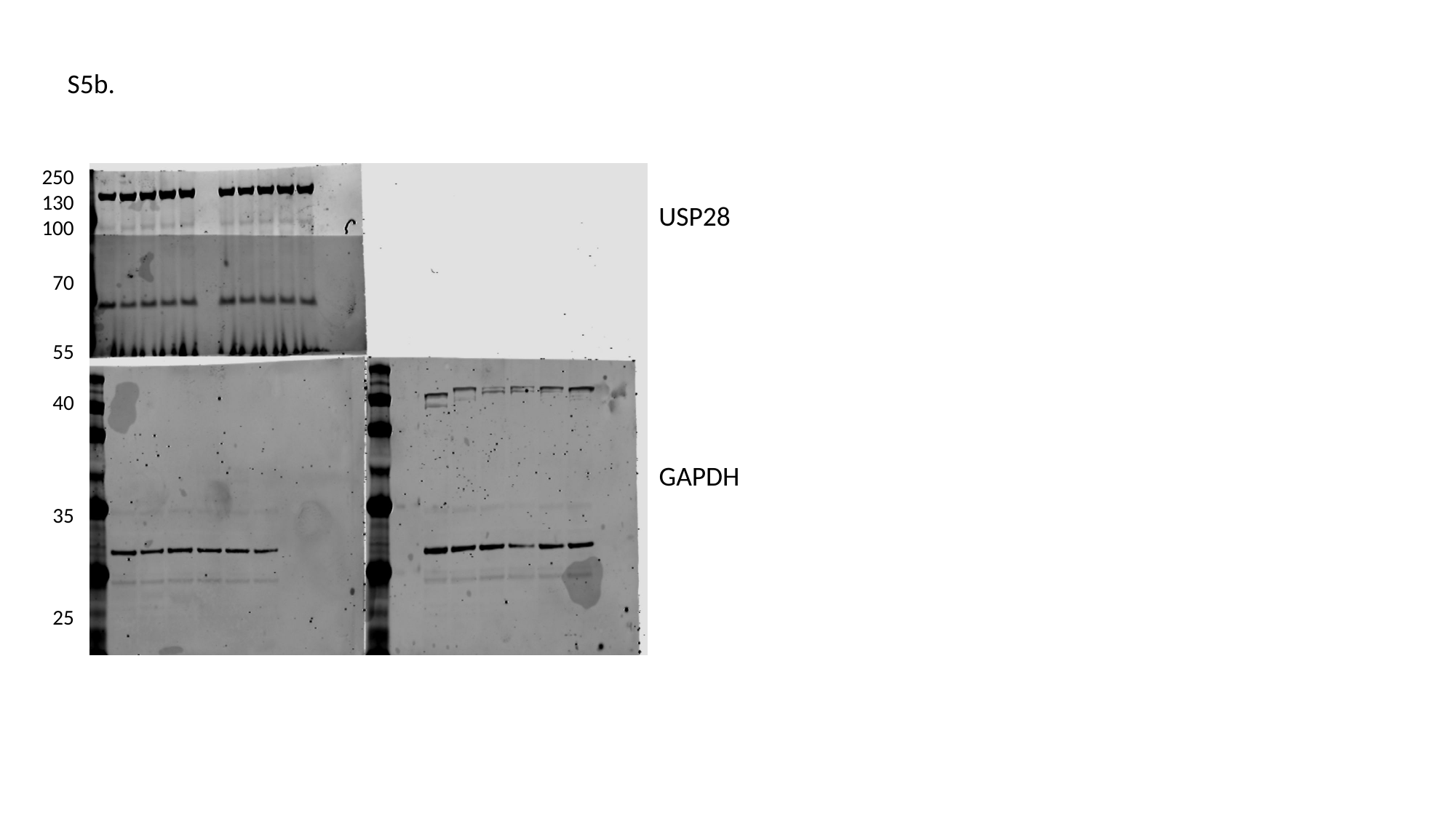

S5b.
250
130
100
70
55
40
35
25
USP28
GAPDH

## Slide 2
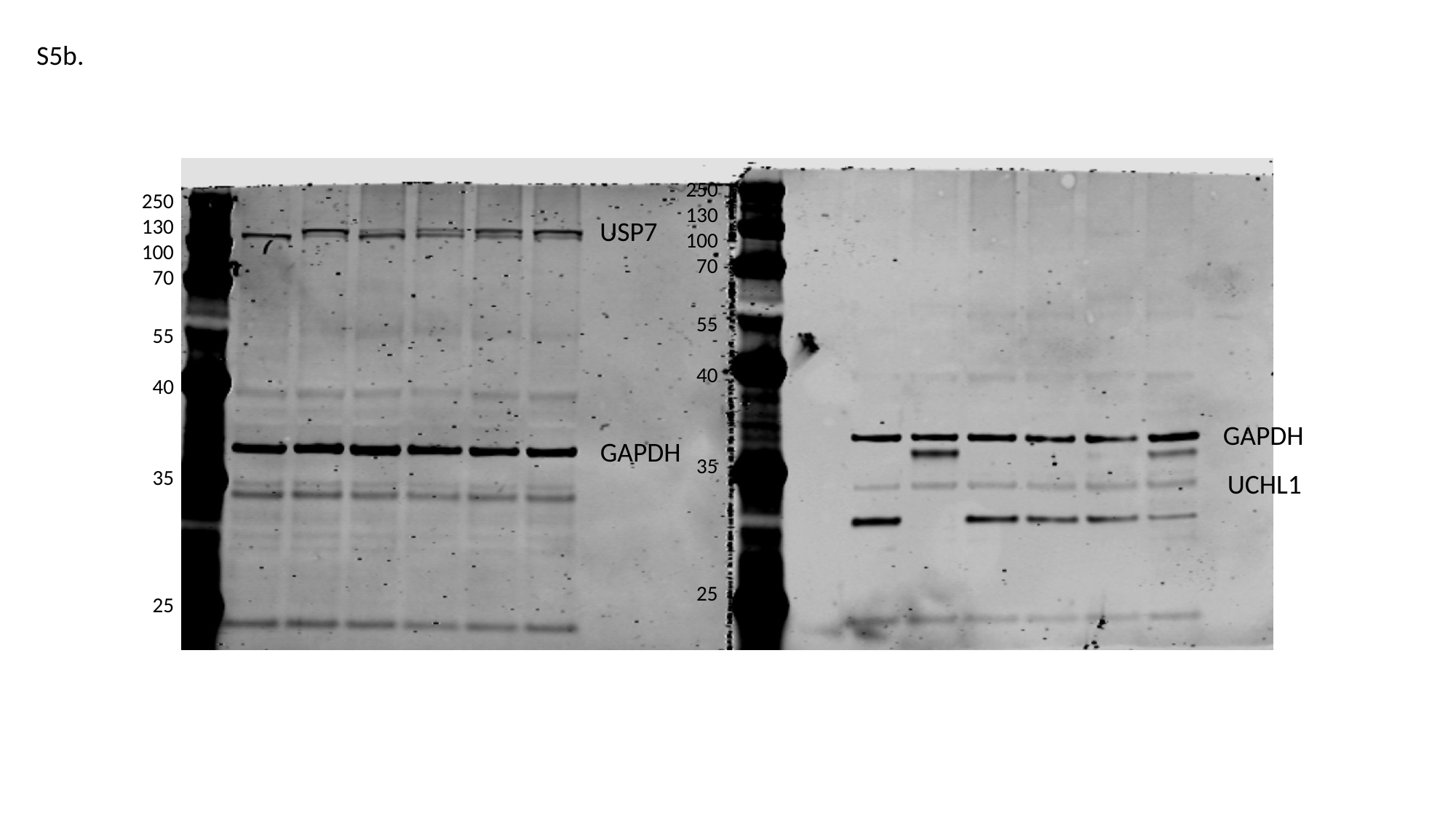

S5b.
250
130
100
70
55
40
35
25
250
130
100
70
55
40
35
25
USP7
GAPDH
GAPDH
UCHL1

Supplement: Supplementary file 9 — Source Data [file 41467_2023_36246_MOESM9_ESM.zip › Source data_230119/Uncropped gel images/Supplementary Figure 5b.pptx]
